# Supplementary material for: Redox-powered autonomous directional C–C bond rotation under enzyme control
Source: Nature. 2025 Jul 16;644(8075):96–101. doi: 10.1038/s41586-025-09291-6 (PMC12328200; doi:10.1038/s41586-025-09291-6)

nature portfolio

https://doi.org/10.1038/s41586-025-09291-6

Supplementary information

Redox-powered autonomous directional C–C bond rotation under enzyme control

In the format provided by the authors and unedited

Redox-powered autonomous unidirectional rotation about a C–C bond under enzymatic control

Supplementary Information

Jordan Berreur,[1] Olivia F. B. Watts,[1] Theo H. N. Bulless,[1] Nicholas T. O'Donoghue,[1] Marc Del Olmo,[1] Ashley J. Winter,[1] Jonathan Clayden[*1] & Beatrice S. L. Collins[*1]

[1]School of Chemistry, University of Bristol, Cantock's Close, Bristol, BS8 1TS, UK.

Contents

1) General Experimental Details 4
2) Experimental details 7
2.1) Synthesis of **1a** 7
2.2) Oxidation Products of **1a** 13
2.3) Synthesis of **3a** 15
2.4) Oxidation Products of **3a** 19
2.5) Mosher's Ester derivative of **4a**, **6a** 24
2.6) Synthesis of Deuterium-Labelled **3a**, D_2-**3a** 25
2.7) Synthesis of Deuterium-Labelled D_2-**S8e** 34
3) General considerations for biocatalytic reactions 42
4) HPLC Conditions and calibration curves 44
4.1) Calibration for the deracemization of **1a** 44
4.2) Normal phase calibration (operation of **3a**) 45
4.3) Reverse phase calibration (operation of **3a**) 46
5) Deracemization of **1a** 49
6) Oxidation of **3a** 53
7) Kinetic analysis 54
7.1) Determination of the rate of oxidation $r_{ox(Ra,Sa)}$ 54
7.2) Determination of the rate of reduction r_{red} 56
7.3) Determination of the rate of enantiomerization r_{enant} 60
8) Autonomous operation 67
8.1) Reverse phase calibration (operation of **3a**) 67
8.2) Operation of **3a** under the redox cyclic reaction network 70
8.3) Evolution of deuterium content over time 72
8.4) Pulsed $H_3N{\cdot}BH_3$ experiment 75
8.5) Analysis of the Fuel-to-Waste Reaction 78
9) Determining the directionality of the rotation 82

9.1) Biocatalytic oxidation of deuterated and enantioenriched (S_a)-D_2-**S8e**, (R_a)-D_2-**S8e** and unlabelled **S8e** 82

9.2) Biocatalytic oxidation of deuterated and enantioenriched (S_a)-D_2-**3a**, (R_a)-D_2-**3a** and unlabelled **3a** 88

10) Mean number of 360° rotations after 48 hours of operation 93

11) Code for simulating rotations 95

12) References 100

13) NMR spectra 102

1) General Experimental Details

Reagents and solvents. Reactions with air- or moisture-sensitive materials were carried out in oven- or flame-dried glassware under a nitrogen atmosphere using standard Schlenk line techniques. Bulk solutions were evaporated under reduced pressure using a Büchi or IKA rotary evaporator. Unless otherwise stated, all reagents, non-enzymatic catalysts, and solvents were obtained from commercial sources and were used without further purification. All anhydrous solvents were commercially supplied or, in the case of toluene, CH_2Cl_2, Et_2O, THF, MeCN or hexane, provided by the communal stills of the School of Chemistry, University of Bristol (Anhydrous Engineering Ltd. modified Grubbs system of double alumina and alumina-copper catalysed drying columns). Petroleum ether is a mixture with the boiling point range of 40–60 °C. H_2O used is deionised using a Veolia laboratory water purification system.

Chromatography. Flash column chromatography was carried out using VWR silica gel 40–63 μm or using a Biotage Isolera Four, Biotage Sfär Silica D Duo 60 μM columns. Thin-layer chromatography (TLC) was performed using Merck aluminium-backed plates (Silica gel 60 F_{254}). Compounds were visualized under UV light, at either 254 nm or 365 nm, by staining with aqueous basic potassium permanganate, aqueous acidic 2,4-dinitrophenylhydrazine (DNP) solution, ethanolic phosphomolybdic acid (PMA), ceric ammonium molybdate (CAM), or ethanolic acidic *p*-anisaldehyde. Normal phase preparatory High Performance Liquid Chromatography (HPLC) was performed on an ACCQPrep HP125 system. Preparatory Column: Kromasil 60-5SIL 250 × 21.2 mm. Analytical and semi-preparative normal phase High Performance Liquid Chromatography (HPLC) was performed on an Agilent Technologies 1260 Infinity.

Nuclear magnetic resonance spectroscopy. ^{1}H, ^{13}C{^{1}H}, ^{19}F, and ^{11}B{^{1}H} nuclear magnetic resonance (NMR) spectra were recorded using Jeol ECP (Eclipse) 300 MHz, Jeol ECS 400 MHz, Jeol JNM-ECZ 400 MHz, Varian CNMR 400 MHz, Bruker Ascend^{TM} DPX 400 MHz, Bruker Avance III HD 500 MHz (TCI Prodigy probe), Bruker Avance III HD 500 MHz (equipped with Cryo Probe), and Bruker Neo 600 MHz (equipped with Cryo Probe) spectrometers. ^{1}H and ^{13}C positive chemical shifts (δ) are referenced to $CHCl_3$ (^{1}H: 7.26 ppm, ^{13}C: 77.16 ppm), CD_3OD (^{1}H: 3.31 ppm, ^{13}C: 49.00 ppm), $(CD_3)_2CO$ (^{1}H: 2.05 ppm, ^{13}C: 29.84 ppm), $(CD_3)_2SO$ (^{1}H: 2.50 ppm, ^{13}C: 39.52 ppm), or THF-d_8 (^{1}H: 3.58, 1.73 ppm, ^{13}C: 67.57, 25.37 ppm) and are given in parts per million (ppm). Coupling constants (*J*) are given in Hertz (Hz) and reported as observed. ^{11}B{^{1}H} NMR spectra are obtained using thin wall quartz NMR tubes sourced from Wilmad, unless otherwise stated. The ^{1}H NMR data are reported as follows: chemical shift (multiplicity, coupling constants, integral). The ^{11}B{^{1}H}, ^{13}C{^{1}H} and ^{19}F NMR

data are reported as follows: chemical shift (multiplicity, coupling constants). Splitting patterns are abbreviated to: singlet (s), doublet (d), triplet (t), quartet (q), multiplet (m), broad (br.) or some combination thereof.

Infrared spectroscopy. Infrared (IR) spectra were recorded on a Perkin Elmer Spectrum Two FT-IR spectrophotometer as a thin film with selected absorption maxima (*ν*) reported in wavenumbers (cm^{-1}).

Mass spectrometry. High resolution mass spectrometry (HRMS) spectra were recorded on a Thermo Scientific QExactive by Electron Ionisation (EI), on a Bruker Daltonics micrOTOF II by Electrospray Ionisation (ESI), on a Waters Synapt G2-Si fitted with a Triversa Nanomate by nanospray ESI, or on a Thermo Scientific Orbitrap Elite by Atmospheric Pressure Chemical Ionisation (APCI). In the cases where the reported compound contains boron, silicon, chlorine or bromine the masses are given for the ^{11}B, ^{28}Si ^{35}Cl or ^{79}Br isotopes respectively.

Melting points. Melting points were recorded in degrees Celsius (°C), using a Gallenkamp or a Cole-Parmer SMP50 melting point apparatus and are reported uncorrected.

UV-Vis. UV-Vis spectroscopy was performed using transparent 1 cm plastic cuvettes which were placed in the cuvette port of a Denovix DS-11+ UV-Vis spectrometer pre-heated to 40 °C. The temperature was allowed to equilibrate for at least 5 min before any measurement.

Circular dichroism (CD). Measurements recorded on a JASCO J-815 Circular Dichroism Spectrometer. The recorded wavelengths were between 200–290 nm, with the sample in MeOH. The data reported is baseline corrected and smoothed.

Enantiomeric ratios. Enantiomeric ratios were determined by HPLC on Agilent 1100 series or Agilent Technologies 1260 Infinity with UV detection at 220, 254 or 270 nm as indicated. Hexane:isopropanol (IPA) mixtures were used as the eluent for all separations. Separations were performed at room temperature using the indicated column.

Biocatalytic reaction quantification: Yield quantification of biocatalytic reactions was performed using reverse phase HPLC, on either an Agilent 1260 Infinity II LCMS System, equipped with an Agilent Single Quadrupole ESI spectrometer in positive and negative modes, with detection between 150 and 1200 m/z units, or an Agilent 1260 Infinity II HPLC system, with detection carried out at 270 nm. H_2O:MeCN (with 0.1% formic acid) mixtures were used as eluents for all separations. Separations were performed at room temperature.

Naming of Compounds. Compound names are those generated by ChemBioDraw 20.0 software (PerkinElmer), following IUPAC nomenclature.

Isotopically Labelled Compounds. Full characterisation data is provided for the parent unlabelled compounds. ^{1}H NMR spectroscopy data is provided for isotopically labelled compounds.

Microwave reactor. Microwave reactions were performed in a Biotage Initiator+ using Biotage microwave vials.

Error analysis. Errors are derived from triplicate reactions where the error given is the population standard deviation between the triplicates, unless otherwise stated.

2) Experimental details

2.1) Synthesis of 1a

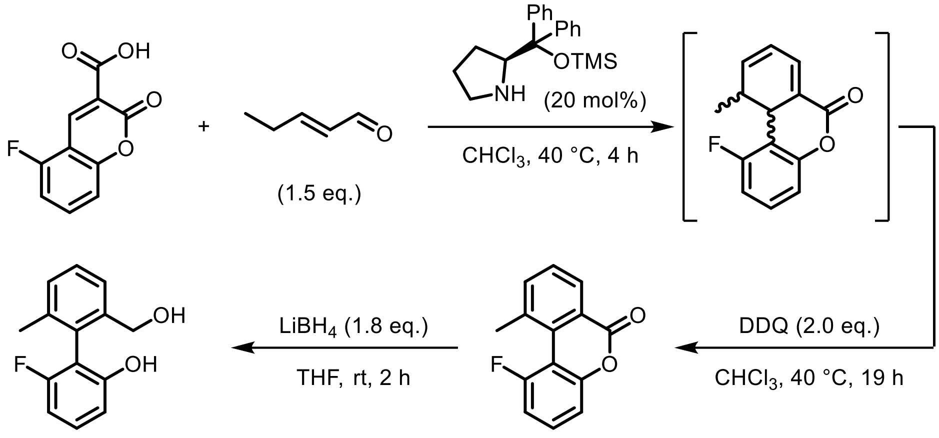

Scheme S1 General synthetic route for the preparation of **1a**.

1-Fluoro-10-methyl-6H-benzo[c]chromen-6-one (S1a)

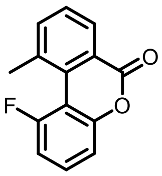

Prepared according to a modified literature procedure.[1] To a solution of 5-fluoro-2-oxo-2H-chromene-3-carboxylic acid (1.0 eq., 300 mg, 1.4 mmol) in $CHCl_3$ (5.7 mL) was added *trans*-2-pentenal (1.5 eq., 210 µL, 2.1 mmol) and (*S*)-(–)-α,α-diphenyl-2-pyrrolidinemethanol trimethylsilyl ether (0.20 eq., 93 mg, 0.29 mmol). The solution was stirred at 40 °C for 4 h, at which point TLC monitoring (R_f (hexane:EtOAc, 89:11): 0.27, UV active spot at 365 nm) showed no remaining starting material. DDQ (2.0 eq., 650 mg, 2.9 mmol) was added to the reaction mixture in one portion and the reaction mixture was stirred at 40 °C for a further 19 h, after which time the reaction mixture was allowed to cool to room temperature, filtered through a plug of silica gel (hexane:EtOAc, 89:11) and concentrated under reduced pressure. The resulting residue was purified by flash column chromatography on silica gel (hexane:EtOAc, 89:11) to give the title compound (250 mg, 1.1 mmol, 76%) as an off-white solid.

***R*$_f$** (hexane:EtOAc, 89:11): 0.27. **^{1}H NMR** (400 MHz, $CDCl_3$) δ 8.25 (dd, *J* = 7.8, 1.4 Hz, 1H), 7.69 (dd, *J* = 7.7, 1.4 Hz, 1H), 7.53 (t, *J* = 7.7 Hz, 1H), 7.44 (td, *J* = 8.2, 5.7 Hz, 1H), 7.21 (dd, *J* = 8.2, 1.2 Hz, 1H), 7.05 (ddd, *J* = 11.4, 8.2, 1.2 Hz, 1H), 2.55 (d, *J* = 11.6 Hz, 3H). **^{13}C NMR** (101 MHz, $CDCl_3$) δ 161.4, 158.6 (d, *J* = 254.2 Hz), 151.9 (d, *J* = 6.8 Hz), 138.2, 136.8, 131.0, 130.2 (d, *J* = 10.8 Hz),

128.8, 128.0, 123.6, 113.2 (d, J = 3.4 Hz), 112.1 (d, J = 24.5 Hz), 109.1 (d, J = 17.5 Hz), 22.8 (d, J = 22.5 Hz). **^{19}F NMR** (376 MHz, $CDCl_3$) δ −100.96 (pd, J = 11.6, 5.7 Hz). **IR** ν (cm^{-1}): 1733, 1620, 1590, 1479, 1437, 1256, 1225. **HRMS** (EI)$^+$ *m/z*: Calculated for $C_{14}H_9O_2F$ 228.0581; Found [M]$^+$: 228.0575. **mp** 109–110 °C.

6-Fluoro-2'-(hydroxymethyl)-6'-methyl-[1,1'-biphenyl]-2-ol (1a)

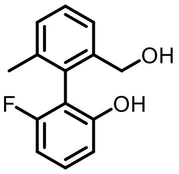

To a solution of 1-fluoro-10-methyl-6H-benzo[c]chromen-6-one **S1a** (1.0 eq., 160 mg, 0.71 mmol) in THF (5.1 mL), $LiBH_4$ (1.8 eq., 4 M in THF, 0.33 mL, 1.30 mmol) was added dropwise and the reaction mixture was stirred for 2 h. The reaction mixture was then cooled to 0 °C and H_2O (2 mL) was added dropwise, followed by HCl (1 M), until the pH of the solution was 3. The reaction mixture was then concentrated under reduced pressure and the remaining aqueous solution was made up to 25 mL with H_2O and extracted with EtOAc (3 × 25 mL). The organic phases were combined and washed with H_2O (2 × 50 mL), brine (50 mL), dried over Na_2SO_4, filtered, and concentrated under reduced pressure, to provide the title compound (160 mg, 0.690 mmol, 97%) as a pale yellow solid.

***R*$_f$** (hexane:EtOAc, 60:40): 0.16. **^{1}H NMR** (400 MHz, CD_3OD) δ 7.42 (d, J = 7.7 Hz, 1H), 7.29 (t, J = 7.7 Hz, 1H), 7.26 – 7.16 (m, 2H), 6.74 (dd, J = 8.3, 1.0 Hz, 1H), 6.73 – 6.61 (m, 1H), 4.41 – 4.27 (ABq, $\Delta\delta_{AB}$ = 0.04, J_{AB} = 17.2 Hz, 2H), 2.03 (s, 3H). **^{13}C NMR** (101 MHz, CD_3OD) δ 161.7 (d, J = 241.0 Hz), 157.1 (d, J = 7.4 Hz), 141.6, 138.5, 130.9, 130.4 (d, J = 10.6 Hz), 129.4 C-11, 128.9, 124.9, 115.6 (d, J = 20.7 Hz), 112.4 (d, J = 2.9 Hz), 107.4 (d, J = 23.1 Hz), 63.2, 20.0. **^{19}F NMR** (376 MHz, CD_3OD) δ −115.7 (t, J = 7.9 Hz). **IR** ν (cm^{-1}): 3287, 2924, 1620, 1457, 1292, 1001. **HRMS** (nanospray ESI)$^-$ *m/z*: Calculated for $C_{14}H_{12}O_2F$ 231.0821; Found [M-H]$^-$ 231.0822. **mp**: 101–103 °C.

(*R*a)-6-Fluoro-2'-(hydroxymethyl)-6'-methyl-[1,1'-biphenyl]-2-ol ((*R*a)-1a)

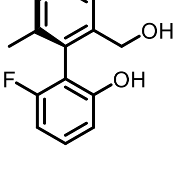

To a solution of borane tetrahydrofuran complex [1.0 M in THF] (4.0 eq., 0.88 mL, 0.88 mmol) in toluene (3.0 mL) at 0 °C was added a solution of (*R*)-(+)-2-methyl-CBS-oxazaborolidine (3.0 eq.,

180 mg, 0.66 mmol) in toluene (3.3 mL) dropwise over 10 min. After the addition, the cooling bath was removed, and the mixture was stirred at 30 °C for 30 min. With careful temperature control, a solution of 1-fluoro-10-methyl-6H-benzo[c]chromen-6-one **S1a** (1.0 eq., 50 mg, 0.22 mmol) was then added dropwise at 30 °C over 30 min. The mixture was stirred at 30 °C for 2 h, where another portion of the borane tetrahydrofuran complex [1.0 M in THF] (4.0 eq, 0.88 mL, 0.88 mmol) was added dropwise. The mixture was stirred at 30 °C for a further 18 hours. The reaction was quenched by the addition of HCl (2 M, 1 mL). H_2O (14 mL) and EtOAc (15 mL) were added, the organic and aqueous phases were separated, and the aqueous layer was extracted with EtOAc (15 mL). The combined organic phases were washed with H_2O (2 × 25 mL), brine (25 mL), dried over $MgSO_4$, filtered, and concentrated under reduced pressure. The resulting mixture was purified by flash column chromatography on silica gel (petroleum ether:EtOAc, gradient 90:10–20:80) to provide the title compound (43 mg, 1.8 μmol, 84%) as a colourless solid.

Data in accordance with racemic sample prepared above. **HPLC** CHIRALPAK® IK-3, 3 μm, 4.6 × 250 mm, eluent composition and gradient indicated in table S1, λ = 270 nm, t_R = 7.8 and 8.1 min.

Table S1 Gradient for separation of enantiomers of **1a**, 1 mL/min, 20 min run.

Time (min)	MeCN:H_2O (0.5% Formic Acid)
0	30:70
1	30:70
16	95:5
17	95:5
18	30:70

Enantiomeric ratio: 22.1:77.9 (ee = 56%). Sample solvent composition: 800 μL MeCN:H_2O:AcOH:NaOAc (6.00:3.94:0.0600, NaOAc (1.40 mg/mL))

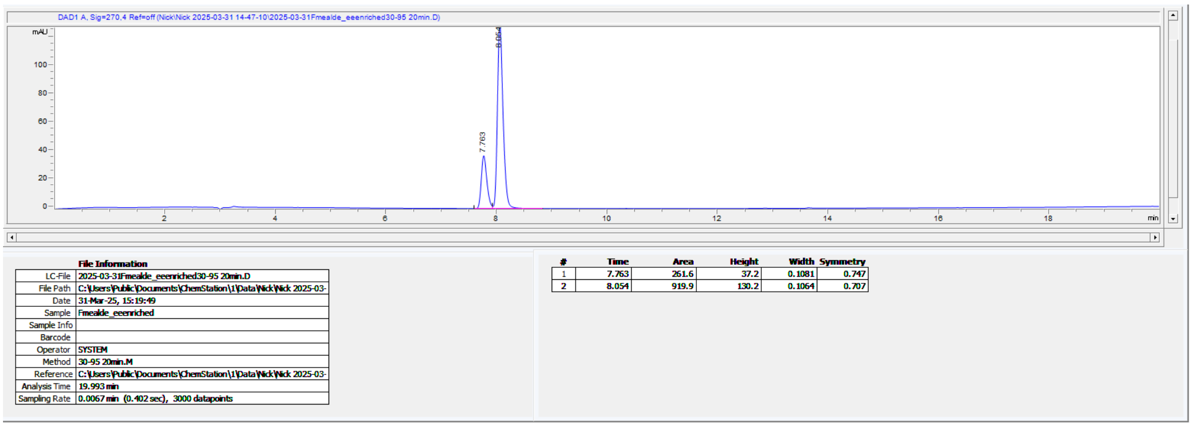

Figure S1 HPLC trace of reduction of **S1a** with (*R*)-(+)-2-methyl-CBS-oxazaborolidine catalyst; CHIRALPAK® IK-3, 3 μm, 4.6 × 250 mm, eluent composition and gradient indicated in table S1, λ = 270 nm, t_R = 7.8 and 8.1 min. Enantiomeric ratio: 22.1:77.9.

We assign the major enantiomer (t_R = 8.1 min) as (R_a)-**1a** based on the established sense of enantioselectivity with which Bringmann's 'lactone method' with (*R*)-(+)-2-methyl-CBS-oxazaborolidine provides enantioenriched biaryl diols.[2,3] We assign the minor enantiomer (t_R = 7.8 min) as (S_a)-**1a**. Establishing the order of elution of the enantiomers of **1a** on the chiral stationary phase IK-3 as described here, allows us to assign the sense of the stereochemical oxidation of **1a** with the oxidation biocatalysts (see Supplementary Information Section 5).

In support of the above stereochemical assignment of **1a**, we have compared the experimental circular dichroism data for the enantiomer that elutes at t_R = 8.1 min (assigned as (R_a)-**1a**) to the CD spectrum of (R_a)-**1a** predicted by time-dependent DFT calculations, as detailed below. The shapes of the experimental and modelled spectra are congruent (Figure S3), supporting our assignment of the absolute configuration of (R_a)-**1a** and providing further confirmation of the validity of the Bringmann 'lactone method' for assigning absolute configuration.

*Assignment of the absolute configuration of **1a** by circular dichroism.* Computational characterizations of the ground- and excited-state properties of **1a** were performed using Gaussian 16 software.[4] Calculations were performed on the three lowest energy conformers of the molecule. The ground state geometry optimisation and excited state transitions of the molecule were computed using density functional theory (DFT) and time-dependent density functional theory (TD-DFT). The ωB97X functional was used with the 6-31+G(d,p) basis set, observing singlet excitations up to n = 15. Solvent effects for MeOH were included by implicit simulation of solvation as a conductor-like polarizable continuum medium. The free energies of the major conformers of the biaryl were determined using an OPT+FREQ calculation using the same functional and basis set in order to allow the relative Boltzmann distribution across the three conformers to be determined.

Table S2 Computed relative populations of the three lowest-energy conformers of **1a**.

Conformer	Structure	Relative energy ($kJ.mol^{-1}$)	Relative population at 298 K
**1** (Benzylic OH perpendicular to ring, towards F)	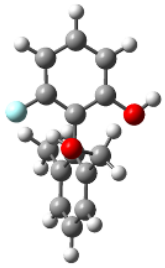	0	0.65
**2** (Benzylic OH in the plane of the ring)	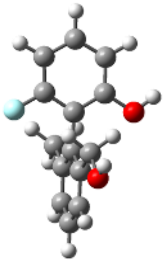	2.8	0.21
**3** (Benzylic OH perpendicular to ring, towards phenolic OH)	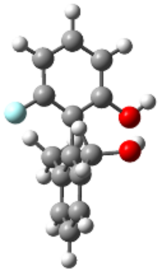	3.7	0.14

The Boltzmann populations at 298 K of the different conformers of (R_a)-**1a** afforded the following Boltzmann-weighted calculated CD spectrum shown in Figure S2.

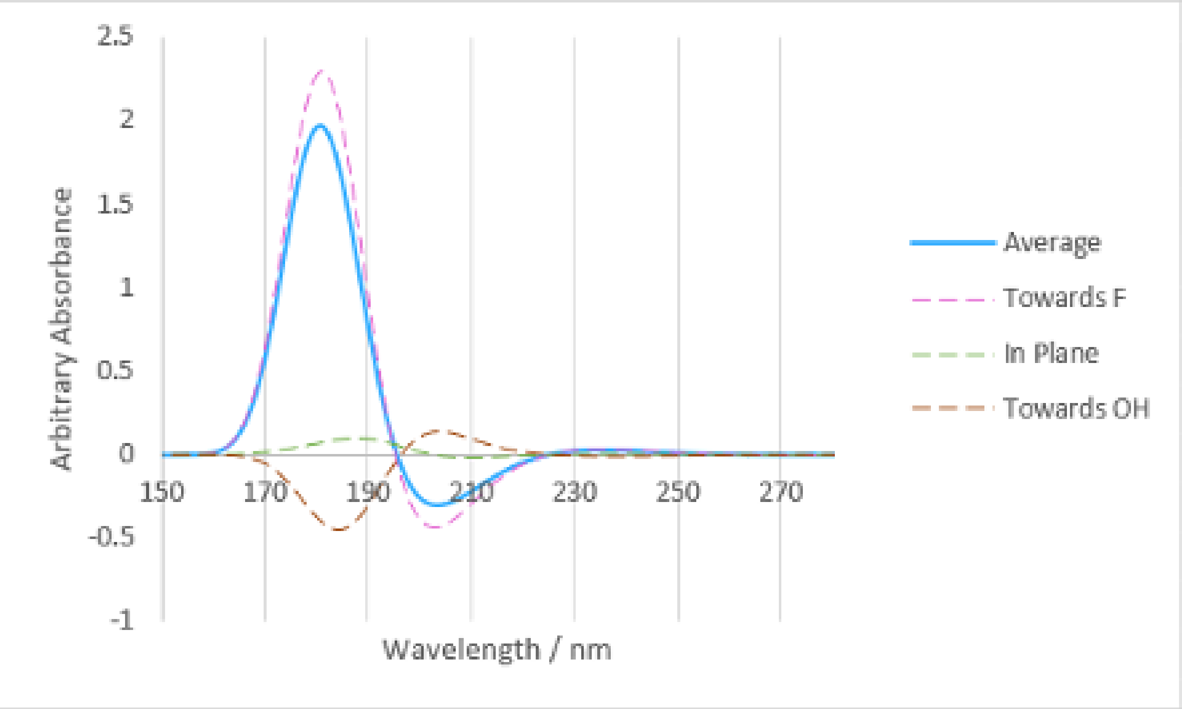

Figure S2 Calculated CD spectra of the three lowest-energy conformers of (R_a)-**1a,** (dashed lines) and their Boltzmann-weighted average (solid blue line).

The calculated CD spectrum was compared with the experimental CD spectrum of a sample of (R_a)-**1a** (60 µM in MeOH, ee = 79%, 298 K, Figure S3). The calculated and experimental CD spectra showed a Cotton effect of the same sign, with the maximum ellipticity of the calculated spectra shifted ~25 nm to shorter wavelength.

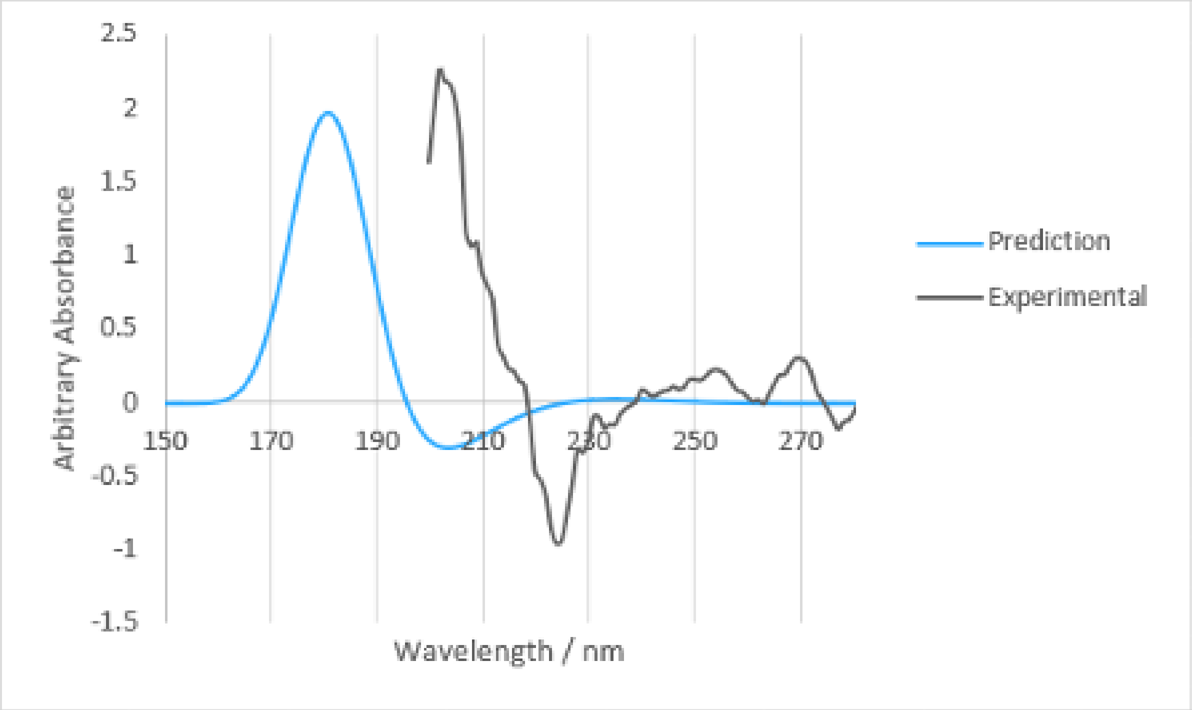

Figure S3 A comparison of the calculated and experimental CD spectra of (R_a)-**1a**.

2.2) Oxidation Products of 1a

2'-Fluoro-6'-hydroxy-6-methyl-[1,1'-biphenyl]-2-carbaldehyde (2a)

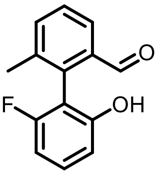

To a solution of 6-fluoro-2'-(hydroxymethyl)-6'-methyl-[1,1'-biphenyl]-2-ol **1a** (1.0 eq., 49 mg, 0.21 mmol) in THF (2.1 mL) at −10 °C was added pyridinium chlorochromate (1.1 eq., 50 mg, 0.23 mmol) portion wise. The mixture was stirred at −10 °C for 5 min, and at room temperature for a further 2 h. Another portion of pyridinium chlorochromate (0.1 eq., 5.0 mg, 20 µmol) was added, and stirring was continued for 30 min. Silica gel was added to the mixture and the volatiles were removed under reduced pressure. Flash column chromatography on silica gel (hexane:EtOAc, 80:20) afforded the title compound (24 mg, 0.10 mmol, 49%) as a yellow oil.

$\boldsymbol{R_f}$ (hexane:EtOAc, 60:40): 0.53. **^{1}H NMR** (400 MHz, CD_3OD) δ 9.69 (t, *J* = 1.0 Hz, 1H), 7.80 (dd, *J* = 7.7, 1.4 Hz, 1H), 7.60 (dt, *J* = 7.2, 1.2 Hz, 1H), 7.44 (t, *J* = 7.6 Hz, 1H), 7.29 (td, *J* = 8.3, 6.7 Hz, 1H), 6.78 (dt, *J* = 8.3, 0.9 Hz, 1H), 6.73 (ddd, *J* = 9.2, 8.3, 1.0 Hz, 1H), 2.14 (s, 3H). **^{13}C NMR** (101 MHz, CD_3OD) δ 193.9, 162.0 (d, *J* = 241.8 Hz), 157.6 (d, *J* = 7.0 Hz), 140.2, 137.1 (d, *J* = 1.1 Hz), 136.7, 135.9, 131.4 (d, *J* = 10.7 Hz), 129.2, 125.6, 113.0 (d, *J* = 19.9 Hz), 112.3 (d, *J* = 3.0 Hz), 107.2 (d, *J* = 22.8 Hz), 19.5. **^{19}F NMR** (377 MHz, CD_3OD) δ −115.34 (dd, *J* = 9.0, 6.8 Hz). **IR** *ν* (cm^{-1}): 3333, 1672, 1619, 1590, 1456, 1249, 1001, 784. **HRMS** $(ESI)^+$ *m/z*: Calculated for $C_{14}H_{11}O_2F$ 230.0738; Found $[M]^+$ 230.0734.

2'-Fluoro-6'-hydroxy-6-methyl-[1,1'-biphenyl]-2-carboxylic acid (S1b)

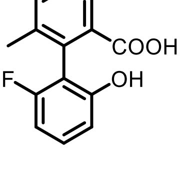

A solution of 1-fluoro-10-methyl-6H-benzo[c]chromen-6-one **S1a** (1.0 eq., 40.2 mg, 0.176 mmol) and LiOH (23 eq., 102 mg, 4.26 mmol) in THF (1.4 mL) and H_2O (4.9 mL) was stirred at 70 °C for 24 h. The reaction mixture was concentrated under reduced pressure, and the remaining aqueous solution was made up to 25 mL with NaOH (2 M) and washed with EtOAc (2 × 25 mL). The mixture was acidified with HCl (2 M) to pH 1 and extracted with EtOAc (3 × 50 mL). The combined organic phases were

then washed with brine (50 mL), dried over $MgSO_4$, filtered, and concentrated under reduced pressure. The crude mixture was purified by flash column chromatography on silica gel (petroleum ether:EtOAc, gradient 80:20–40:60) to provide the title compound (26.5 mg, 0.108 mmol, 61%) as a colourless powder.

***R*f** (EtOAc): 0.51. **^{1}H NMR** (600 MHz, CD_3OD) δ 7.81 (d, *J* = 7.7 Hz, 1H), 7.47 (d, *J* = 7.6 Hz, 1H), 7.35 (t, *J* = 7.7 Hz, 1H), 7.14 (td, *J* = 8.2, 6.6 Hz, 1H), 6.67 (d, *J* = 8.2 Hz, 1H), 6.61 (t, *J* = 8.7 Hz, 1H), 2.09 (s, 3H). **^{13}C NMR** (151 MHz, CD_3OD) δ 171.0, 161.6 (d, *J* = 240.5 Hz), 156.8 (d, *J* = 7.8 Hz), 139.9, 134.3, 133.9, 133.6, 129.7 (d, *J* = 10.7 Hz), 128.7, 128.6, 117.1 (d, *J* = 20.0 Hz), 111.9 (d, *J* = 2.6 Hz), 107.0 (d, *J* = 23.2 Hz), 20.1. **^{19}F NMR** (565 MHz, CD_3OD) δ −(116.65–116.68) (m). **IR** *ν* (cm^{-1}): 3226, 1697, 1624, 1597, 1462, 1292, 1250, 1182, 1002. **HRMS** (nanospray ESI)$^{-}$ *m/z*: Calculated for $C_{14}H_{10}O_3F$ 245.0614; Found $[M-H]^{-}$ 245.0618. **mp**: 129–131 °C.

2.3) Synthesis of 3a

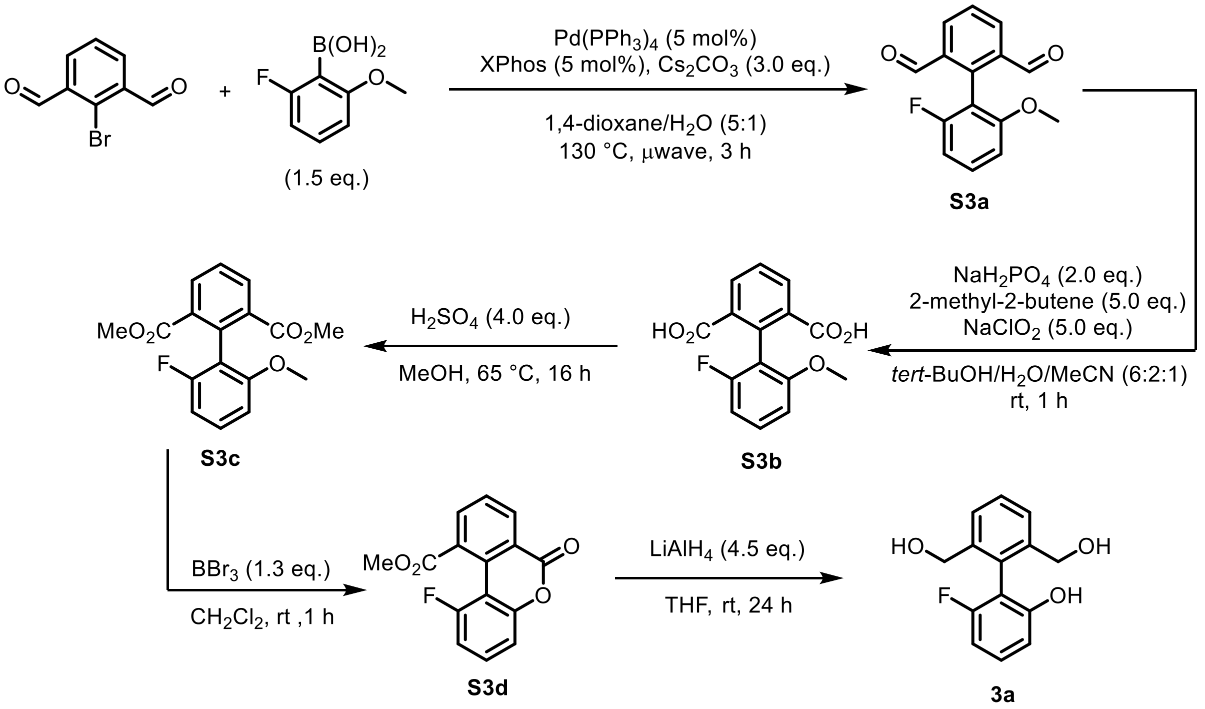

Scheme S2 General synthetic route for the preparation of **3a**.

2'-Fluoro-6'-methoxy-[1,1'-biphenyl]-2,6-dicarbaldehyde (S3a)

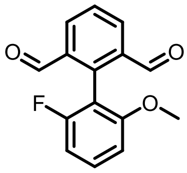

A microwave vial (10–20 mL) was charged with 2-bromoisophthalaldehyde (1.0 eq., 100 mg, 0.47 mmol), (2-fluoro-6-methoxyphenyl)boronic acid (1.5 eq., 120 mg, 0.70 mmol), Cs_2CO_3 (3.0 eq., 46 mg, 1.4 mmol) and XPhos (5 mol%, 11 mg, 20 µmol) followed by 1,4-dioxane (5 mL) and H_2O (1 mL) and N_2 was bubbled through the suspension for 5 min. $Pd(PPh_3)_4$ (5 mol%, 27 mg, 20 µmol) was added and N_2 was bubbled through the suspension for a further 5 min, after which time the vial sealed and heated to 130 °C in the microwave. After 3 h, the vial was cooled to room temperature, opened, and H_2O (10 mL) was added. The aqueous phase was extracted with EtOAc (3 × 10 mL) and the organic phases were combined, washed with brine (20 mL), dried over $MgSO_4$, filtered and the solvent was removed under reduced pressure. The resulting residue was purified by flash column chromatography on silica gel (petroleum ether: EtOAc, gradient 100:0–25:75) to provide the title compound (73 mg, 0.29 mmol, 61%) as a colourless oil.

***R*$_f$** (hexane:EtOAc, 80:20): 0.25. **^{1}H NMR** (400 MHz, $CDCl_3$) δ 9.78 (dd, *J* = 1.5, 0.8 Hz, 2H), 8.28 (d, *J* = 7.7 Hz, 2H), 7.69 (td, *J* = 7.7, 0.9 Hz, 1H), 7.48 (td, *J* = 8.5, 6.7 Hz, 1H), 6.92 – 6.82 (m, 2H), 3.75 (s, 3H). **^{13}C NMR** (101 MHz, $CDCl_3$) 190.9, 160.7 (d, *J* = 245.9 Hz), 158.4 (d, *J* = 6.6 Hz), 137.7, 135.0, 133.0, 131.9 (d, *J* = 10.4 Hz), 129.1, 109.4 (d, *J* = 19.2 Hz), 108.5 (d, *J* = 22.5 Hz), 106.7 (d, *J* = 2.8 Hz), 56.3. **^{19}F NMR** (377 MHz, $CDCl_3$) −111.33 (dd, *J* = 6.8, 6.3 Hz). **mp**: 87–89 °C.

Data are consistent with those reported in the literature.[5]

2'-Fluoro-6'-methoxy-[1,1'-biphenyl]-2,6-dicarboxylic acid (S3b)

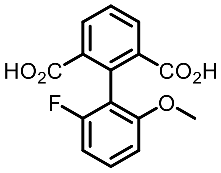

Prepared according to a modified literature procedure.[6] To a solution of 2’-fluoro-6’-methoxy-[1,1’-biphenyl]-2,6-dicarbaldehyde **S3a** (1.0 eq., 671 mg, 2.60 mmol), NaH_2PO_4 (2.0 eq., 624 mg, 5.20 mmol), and 2-methyl-2-butene (5.0 eq., 1.38 mL, 13.0 mmol) in a *tert*-BuOH/H_2O/MeCN mixture (13 mL, 6:2:1 v/v) was added $NaClO_2$ (5.0 eq., 80% (pur. w/w), 1.47 g, 13.0 mmol), and the mixture was stirred at room temperature for 1 h. The mixture was quenched with Na_2SO_3 (aq. sat., 15 mL) and treated with HCl (1 M) until the reaction mixture pH was 1. The reaction mixture was extracted with EtOAc (3 × 15 mL), and the organic layers washed with H_2O (15 mL), dried over $MgSO_4$, filtered, and concentrated under reduced pressure to afford the crude product. The crude product was triturated between EtOAc and hexane to afford the title compound (612 mg, 2.11 mmol 81% yield) as a colourless solid.

^{1}H NMR (400 MHz, $(CD_3)_2CO$) δ 8.12 (d, *J* = 7.8 Hz, 2H), 7.62 (t, *J* = 7.8 Hz, 1H), 7.28 (td, *J* = 8.4, 6.7 Hz, 1H), 6.80 (dt, *J* = 8.4, 0.9 Hz, 1H), 6.69 (ddd, *J* = 9.3, 8.4, 0.9 Hz, 1H), 3.68 (s, 3H). **^{13}C NMR** (126 MHz, $(CD_3)_2CO$) δ 168.0, 161.1 (d, *J* = 240.5 Hz), 159.0 (d, *J* = 7.6 Hz), 134.8, 133.6, 133.5, 130.0 (d, *J* = 10.6 Hz), 128.7, 117.6 (d, *J* = 19.4 Hz), 108.1 (d, *J* = 22.9 Hz), 107.4 (d, *J* = 2.6 Hz), 56.4. **^{19}F NMR** (377 MHz, $(CD_3)_2CO$) δ −116.3 (s). **IR** *ν* (cm^{-1}): 2922, 1695, 1472, 1271, 1240, 1084, 781. **HRMS** (nanospray ESI)$^-$ *m*/*z*: Calculated for $C_{15}H_{10}O_5F$ 289.0512; Found $[M]^-$ 289.0505. **mp**: 243–245 °C.

Dimethyl 2'-fluoro-6'-methoxy-[1,1'-biphenyl]-2,6-dicarboxylate (S3c)

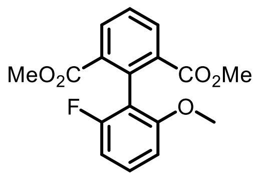

Triturated 2'-fluoro-6'-methoxy-[1,1'-biphenyl]-2,6-dicarboxylic acid **S3b** (1.0 eq., 1.58 g, 5.44 mmol) was dissolved in MeOH (18 mL), and sulfuric acid (>95%, 4.0 eq., 1.20 mL, 21.8 mmol) was added. The mixture was heated to reflux and stirred for 16 h. The mixture was allowed to cool to room temperature, and the solvent was removed under reduced pressure. The obtained brown residue was dissolved in EtOAc and washed with $NaHCO_3$ (aq. sat., 2 × 15 mL), and brine (15 mL). The organic phase was dried over Na_2SO_4, filtered, and concentrated under reduced pressure. The resulting residue was purified by flash column chromatography on silica gel (hexane:EtOAc, 85:15) to provide the title compound (1.55 g, 4.88 mmol, 90% over 2 steps) as a colourless solid.

$\boldsymbol{R_f}$ (hexane:EtOAc, 70:30): 0.29. **^{1}H NMR** (400 MHz, $CDCl_3$) δ 8.09 (d, *J* = 7.9 Hz, 2H), 7.52 (t, *J* = 7.8 Hz, 1H), 7.30 (td, *J* = 8.4, 6.7 Hz, 1H), 6.80 – 6.70 (m, 2H), 3.70 (s, 3H), 3.65 (s, 6H). **^{13}C NMR** (126 MHz, $CDCl_3$) δ 167.3, 159.9 (d, *J* = 242.6 Hz), 157.6 (d, *J* = 7.4 Hz), 133.3, 133.1, 132.9, 129.4 (d, *J* = 10.4 Hz), 128.0, 116.4 (d, *J* = 19.2 Hz), 107.8 (d, *J* = 22.6 Hz), 106.2 (d, *J* = 2.7 Hz), 56.2, 52.3. **^{19}F NMR** (377 MHz, $CDCl_3$) δ −(103.50 – 120.11) (m). **IR** *ν* (cm^{-1}): 2952, 1728, 1472, 1256, 1205, 1083, 765. **HRMS** $(ESI)^+$ *m/z*: Calculated for $C_{14}H_9O_5FNa$ 341.0796; Found $[M+Na]^+$ 341.0801. **mp**: 93–95 °C.

Methyl 1-fluoro-6-oxo-6H-benzo[c]chromene-10-carboxylate (S3d)

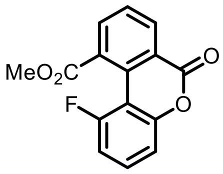

To a solution of dimethyl 2'-fluoro-6'-methoxy-[1,1'-biphenyl]-2,6-dicarboxylate **S3c** (1.0 eq., 1.49 g, 4.68 mmol) in CH_2Cl_2 (17.3 mL) at −78 °C was added boron tribromide (1.3 eq., 1.0 M in heptane, 6.1 mL, 6.10 mmol) dropwise over 15 min. After the addition, the cooling bath was removed, and the mixture was stirred at room temperature for 1 h or until TLC monitoring (hexane:EtOAc, 70:30) indicated reaction completion. The reaction was quenched by the dropwise addition of $NaHCO_3$ (aq. sat., 10 mL). The organic and aqueous phases were separated, and the aqueous phase was extracted with CH_2Cl_2 (3 × 20 mL). The combined organic phases were washed with brine (20 mL), dried over Na_2SO_4,

filtered, and concentrated under reduced pressure. The resulting residue was purified by flash column chromatography on silica gel (CH_2Cl_2) to provide the title compound (1.05 g, 3.88 mmol, 83%) as a colourless solid.

***R*$_f$** (hexane:EtOAc, 70:30): 0.37. **^{1}H NMR** (400 MHz, $CDCl_3$) δ 8.54 (dd, *J* = 7.9, 1.5 Hz, 1H), 8.16 (dd, *J* = 7.6, 1.5 Hz, 1H), 7.67 (t, *J* = 7.7 Hz, 1H), 7.47 (td, *J* = 8.3, 5.9 Hz, 1H), 7.24 (dt, *J* = 8.3, 1.1 Hz, 1H), 7.03 (ddd, *J* = 11.8, 8.3, 1.2 Hz, 1H), 3.90 (s, 3H). **^{13}C NMR** (151 MHz, $(CD_3)_2CO$) δ 168.6 (d, *J* = 3.0 Hz), 160.3 (d, *J* = 251.1 Hz), 160.2, 152.9 (d, *J* = 6.2 Hz), 136.7, 133.2, 132.6 (d, *J* = 4.9 Hz), 132.4 (d, *J* = 11.3 Hz), 130.4 (d, *J* = 2.5 Hz), 130.1, 124.5, 114.2 (d, *J* = 3.1 Hz), 112.3 (d, *J* = 24.2 Hz), 108.8 (d, *J* = 16.1 Hz), 52.7. **^{19}F NMR** (377 MHz, $(CD_3)_2CO$) δ −109.2 (s). **IR** *ν* (cm^{-1}): 2954, 1733, 1621, 1300, 1254, 1084, 1040, 793, 718. **HRMS** $(ESI)^+$ *m/z*: Calculated for $C_{15}H_{10}FO_4$ 273.0558; Found $[M+H]^+$ 273.0550. **mp**: 149–152 °C.

(2'-Fluoro-6'-hydroxy-[1,1'-biphenyl]-2,6-diyl)dimethanol (3a)

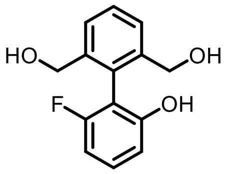

$LiAlH_4$ (4.5 eq., 47 mg, 1.2 mmol) was added to a stirred solution of methyl 1-fluoro-6-oxo-6*H*-benzo[c]chromene-10-carboxylate **S3d** (1.0 eq., 75 mg, 0.28 mmol) in THF (5 mL) at 0 °C. After 5 min, the reaction mixture was allowed to warm to room temperature and stirred for a further 24 h. The reaction mixture was then cooled to 0 °C and H_2O (10 mL) was added dropwise, after which the pH was adjusted to ~3 using HCl (1 M). The reaction mixture was then extracted with EtOAc (3 × 10 mL) and the organic phases were combined, washed with brine (30 mL), dried over $MgSO_4$, filtered, and concentrated under reduced pressure. The resulting residue was purified by flash column chromatography on silica gel (petroleum ether:EtOAc, gradient 70:30–20:80), to provide the title compound (50 mg, 0.20 mmol, 73%) as a colourless solid.

^{1}H NMR (600 MHz, $(CD_3)_2CO$) δ 8.72 (s, 1H), 7.55 (d, *J* = 7.7 Hz, 2H, H-2), 7.41 (t, *J* = 7.7 Hz, 1H), 7.29 – 7.22 (m, 1H), 6.83 (dt, *J* = 8.2, 0.9 Hz, 1H), 6.71 (ddd, *J* = 9.2, 8.3, 1.0 Hz, 1H), 4.36 (s, 4H), 4.13 (s, 2H) **^{13}C NMR** (151 MHz, $(CD_3)_2CO$) δ 160.9 (d, *J* = 240.81 Hz), 156.6 (d, *J* = 7.33 Hz), 141.7, 130.2 (d, *J* = 10.65 Hz), 128.5, 127.7, 125.5, 114.0 (d, *J* = 20.69 Hz), 112.5 (d, *J* = 2.82 Hz), 107.1 (d, *J* = 22.95 Hz), 62.3. **^{19}F NMR** (376 MHz, $(CD_3)_2CO$) δ −114.6 (d, *J* = 2.2 Hz). **IR** *ν* (cm^{-1}): 3346, 1620, 1466, 1234, 1050, 1002, 790. **HRMS** $(ESI)^-$ *m/z*: Calculated for $C_{14}H_{12}O_3F$ 247.0770; Found $[M-H]^-$ 247.0774. **mp**: 162–164 °C.

2.4) Oxidation Products of 3a

2'-Fluoro-6'-hydroxy-6-(hydroxymethyl)-[1,1'-biphenyl]-2-carbaldehyde (4a)

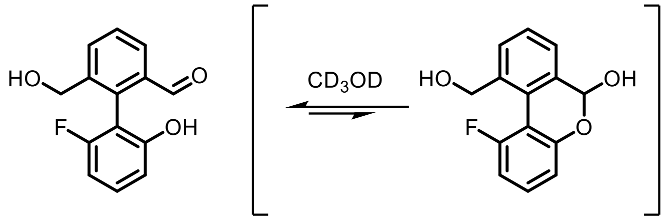

To a stirred of (2'-fluoro-6'-hydroxy-[1,1'-biphenyl]-2,6-diyl)dimethanol **3a** (1.0 eq., 110 mg, 0.44 mmol) in THF (4 mL) at 0 °C, was added pyridinium chlorochromate (1.2 eq., 110 mg, 0.52 mmol) portion wise. The reaction was stirred and allowed to warm to room temperature for 2 h, after which time the reaction was quenched with H_2O (4 mL) and diluted with EtOAc (5 mL). The organic layer was collected, and the aqueous layer was extracted with EtOAc (3 × 5 mL). The combined organic extracts were dried over $MgSO_4$, filtered, and concentrated under reduced pressure to afford the crude product, which was purified by flash column chromatography (hexane:EtOAc, gradient 80:20–70:30) to provide the title compound (30 mg, 0.12 mol, 31% yield) as a colourless solid.

***R*$_f$** (hexane:EtOAc, 40:60): 0.33. **^{1}H NMR** (400 MHz, CD_3OD) δ 9.70 (s, 1H), 7.94 (d, J = 7.7 Hz, 1H), 7.90 (d, J = 7.8 Hz, 1H), 7.58 (t, J = 7.7 Hz, 1H), 7.31 (td, J = 8.3, 6.7 Hz, 1H), 6.83 – 6.70 (m, 2H), 4.45 (d, J = 2.3 Hz, 2H). **^{13}C NMR** (101 MHz, CD_3OD) δ 193.5, 162.0 (d, *J* = 242.2 Hz), 157.6 (d, *J* = 6.8 Hz), 143.4, 135.6, 134.9, 133.2, 131.7 (d, *J* = 10.6 Hz), 129.5, 126.8, 112.3 (d, *J* = 3.0 Hz), 107.2 (d, *J* = 22.7 Hz), 105.9 (d, *J* = 22.7 Hz), 62.0. **^{19}F NMR** (376 MHz, CD_3CN) δ −114.6 (t, *J* = 7.9 Hz). **IR** *ν* (cm^{-1}): 3335, 1687, 1620, 1464, 1250, 1010, 789. **HRMS** $(ESI)^-$ *m/z*: Calculated for $C_{14}H_{10}O_3F$ 245.0614; Found $[M-H]^-$ 245.0613. **mp**: 126–128 °C (decomp.).

Also evident are some minor NMR signals, tentatively assigned to a lactol isomer or its methyl ether.

2'-Fluoro-6'-hydroxy-[1,1'-biphenyl]-2,6-dicarbaldehyde (S3e)

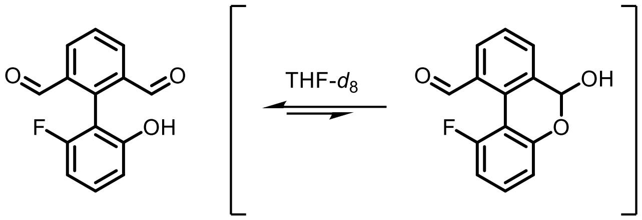

S3e was isolated during the purification of 2’-fluoro-6’-hydroxy-6-(hydroxymethyl)-[1,1’-biphenyl]-2-carbaldehyde **4a** by flash column chromatography (hexane:EtOAc, gradient 80:20–70:30). **S3e** was further purified by passing through a plug of silica (EtOAc), followed by flash column chromatography (hexane:EtOAc, gradient 88:12–0:100) to provide the title compound (12 mg, 48 μmol, 11%) as a colourless solid.

***R*f** (hexane:EtOAc, 50:50): 0.26. **^{1}H NMR** (500 MHz, THF-d_8) δ 9.99 (dd, J = 13.3, 0.7 Hz, 1H), 7.69 (dd, *J* = 7.6, 1.3 Hz, 1H), 7.55 (t, *J* = 7.6 Hz, 1H), 7.01 (d, *J* = 8.1 Hz, 1H), 6.67 (d, *J* = 6.6 Hz, 1H), 6.23 (d, *J* = 6.2 Hz, 1H). **^{13}C NMR** (126 MHz, THF-d_8) δ 189.1 (d, *J* = 13.6 Hz), 159.2 (d, *J* = 247.2 Hz), 154.4 (d, *J* = 5.6 Hz), 136.9, 132.6, 130.5 (d, *J* = 10.9 Hz), 129.5, 128.1, 127.9, 126.4, 115.1 (d, *J* = 3.0 Hz), 110.2 (d, *J* = 15.3 Hz), 109.3 (d, *J* = 23.6 Hz), 93.8. **^{19}F NMR** (471 MHz, THF-d_8) δ −117.40 (ddd, *J* = 13.3, 10.7, 6.3 Hz). **IR** *ν* (cm^{-1}): 3440, 1687, 1589, 1480, 1248, 1013, 935, 797. **HRMS** (nanospray ESI)$^+$ *m/z*: Calculated for $C_{14}H_9O_3F$ 243.0457; Found [M]$^+$ 243.0453. **mp**: 111–113 °C.

Also evident are the following peaks, tentatively assigned to the two diastereoisomers of a minor (<27%) hemiacetal isomer: **^{1}H NMR** (500 MHz, THF-d_8) δ 9.81 (s, 2H), 9.29 (s, 1H), 8.22 (d, *J* = 7.7 Hz, 2H), 7.92 (dd, *J* = 7.7, 1.5 Hz, 1H), 7.72 – 7.68 (m, 1H), 7.37 (td, *J* = 8.2, 6.4 Hz, 1H), 6.92 (ddd, *J* = 10.7, 8.3, 1.1 Hz, 1H), 6.86 – 6.73 (m, 2H); **^{13}C NMR** (126 MHz, THF-d_8) 189.4, 161.9, 135.2, 131.9, 131.2 (d, *J* = 10.8 Hz), 128.5, 111.2, 106.1 (d, *J* = 22.6 Hz). Some ^{13}C signals for this isomer were too weak to be observed. **^{19}F NMR** (471 MHz, THF-d_8) δ −113.69 (t, *J* = 7.8 Hz).

Methyl 1-fluoro-6-hydroxy-6H-benzo[c]chromene-10-carboxylate (S3i)

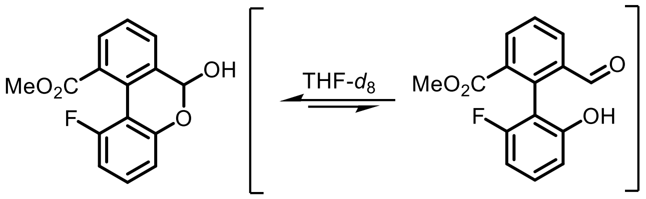

To a stirred of methyl 2'-fluoro-6'-hydroxy-6-(hydroxymethyl)-[1,1'-biphenyl]-2-carboxylate **S3f** (1.0 eq., 91 mg, 0.33 mmol) in THF (3.3 mL) at −10 °C was added pyridinium chlorochromate (1.1 eq., 79 mg, 0.36 mmol) portion wise. The reaction was stirred and allowed to warm to room temperature for 2.3 h. Another portion of pyridinium chlorochromate (0.12 eq., 8.7 mg, 0.040 mmol) was added, and stirring was continued for 45 min. Celite was added to the mixture and the volatiles were removed under reduced pressure. Flash column chromatography (petroleum ether:EtOAc, gradient 90:10–20:80) afforded the title compound (41 mg, 0.15 mol, 46% yield) as a colourless solid.

$\boldsymbol{R}_{\mathbf{f}}$ (hexane:EtOAc, 60:40): 0.27. **^{1}H NMR** (400 MHz, THF-d_8) δ 7.75 (dq, *J* = 8.1, 1.9 Hz, 1H), 7.58 (dq, *J* = 7.7, 1.7 Hz, 1H), 7.44 (tt, *J* = 7.6, 1.8 Hz, 1H), 7.25 (tdt, *J* = 8.5, 6.6, 1.9 Hz, 1H), 6.92 (dq, *J* = 8.1, 1.2 Hz, 1H), 6.78 (ddp, *J* = 10.5, 8.2, 1.3 Hz, 1H), 6.56 (dd, *J* = 6.7, 2.1 Hz, 1H), 6.17 (dt, *J* = 6.8, 1.9 Hz, 1H), 3.73 (d, *J* = 2.0 Hz, 3H). **^{13}C NMR** (101 MHz, THF-d_8) δ 169.2 (d, *J* = 2.4 Hz), 161.1 (d, *J* = 248.5 Hz), 154.9 (d, *J* = 6.2 Hz), 137.7, 131.8 (d, *J* = 3.4 Hz), 131.0, 130.5 (d, *J* = 11.3 Hz), 128.9, 128.3, 125.2, 115.6 (d, *J* = 2.9 Hz), 112.7 (d, *J* = 15.5 Hz), 109.7 (d, *J* = 23.8 Hz), 94.8, 51.9. **^{19}F NMR** (376 MHz, THF-d_8) δ −113.52 (dd, *J* = 11.2, 6.3 Hz). **IR** *ν* (cm^{-1}): 3388, 2952, 1727, 1709, 1621, 1594, 1480, 1448, 1434, 1299, 1290, 1260, 1231, 1196, 1179, 1140, 1098, 1068, 1024, 1010, 985, 961, 795, 780, 767, 755, 737, 716. **HRMS** (nanospray ESI)$^{-}$ *m/z*: Calculated for $C_{15}H_{10}O_4F$ 273.0563; Found $[M-H]^{-}$ 273.0568. **mp**: 109–114 °C.

Also evident are the following peaks, tentatively assigned to the minor (<10%) aldehyde isomer: **^{1}H NMR** (400 MHz, THF-d_8) δ 9.75 (d, *J* = 1.2 Hz, 1H), 8.90 (d, *J* = 1.9 Hz, 1H), 8.19 (dd, *J* = 7.8, 1.5 Hz, 1H), 8.12 (dd, *J* = 7.8, 1.6 Hz, 1H), 6.71 – 6.64 (m, 2H), 2.61 (s, 3H). **^{13}C NMR** (101 MHz, THF-d_8) δ 191.1, 136.0, 130.8, 94.7, 55.0, 52.1. **^{19}F NMR** (376 MHz, THF-d_8) δ –(114.88–114.98) (m). One ^{1}H environment can be observed by 2D NMR at 7.20 ppm, however this overlaps with the aromatic hemiacetal peak at 7.25 ppm. Some ^{13}C signals for this isomer were too weak to be observed.

1-Fluoro-6-hydroxy-6H-benzo[c]chromene-10-carboxylic acid (S3j)

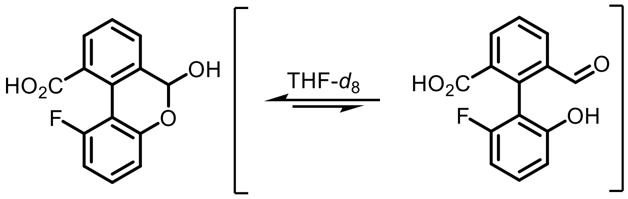

A solution of methyl 1-fluoro-6-hydroxy-6H-benzo[c]chromene-10-carboxylate **S3i** (1.0 eq., 39 mg, 0.17 mmol) and LiOH (24 eq., 99 mg, 4.1 mmol) in THF (2.6 mL) and H_2O (5.7 mL) was stirred at 70 °C for 1.5 h. The reaction mixture was concentrated under reduced pressure, and the remaining aqueous solution was made up to 25 mL with H_2O and washed with CH_2Cl_2 (2 × 25 mL). The mixture was acidified with HCl (2 M) to pH 1 and extracted with EtOAc (3 × 25 mL). The combined organic phases were then washed with brine (25 mL), dried over $MgSO_4$, filtered, and concentrated under reduced pressure. At this point 10 mg of the crude mixture was removed. The remaining crude mixture was purified by flash column chromatography on silica gel (petroleum ether:EtOAc, gradient 83:17–0:100) to provide the title compound (22 mg, 0.83 mmol, 48%) as a colourless powder.

***R*$_f$** (hexane:EtOAc, 30:70): 0.23. **^{1}H NMR** (600 MHz, THF-*d*$_8$) δ 7.79 (dd, *J* = 7.7, 1.4 Hz, 1H), 7.55 (dd, *J* = 7.5, 1.4 Hz, 1H), 7.41 (t, *J* = 7.6 Hz, 1H), 7.22 (td, *J* = 8.2, 6.1 Hz, 1H), 6.89 (d, *J* = 8.1 Hz, 1H), 6.74 (ddd, *J* = 10.9, 8.3, 1.1 Hz, 1H), 6.51 (d, *J* = 6.7 Hz, 1H), 6.15 (d, *J* = 3.2 Hz, 1H). **^{13}C NMR** (151 MHz, THF-*d*$_8$) δ 169.8 (d, *J* = 2.6 Hz), 161.5 (d, *J* = 249.4 Hz), 155.0 (d, *J* = 6.2 Hz), 137.9, 132.9 (d, *J* = 3.3 Hz), 131.0, 130.3 (d, *J* = 10.9 Hz), 128.6, 128.2, 125.3, 115.4 (d, *J* = 2.8 Hz), 113.1 (d, *J* = 15.5 Hz), 109.8 (d, *J* = 23.6 Hz), 95.0. **^{19}F NMR** (565 MHz, THF-*d*$_8$) δ −110.33 (dd, *J* = 11.0, 6.1 Hz). **IR** *v* (cm^{-1}): 3078, 2957, 1696, 1622, 1595, 1480, 1448, 1260, 1234, 1186, 1147, 1017, 972, 793, 732. **HRMS** (nanospray ESI)$^-$ *m/z*: Calculated for $C_{14}H_8O_4F$ 259.0407; Found [M−H]$^-$ 259.0409. **mp:** 112–117 °C

Also evident are the following peaks, tentatively assigned to the minor (<12%) aldehyde isomer: **^{1}H NMR** (600 MHz, THF-*d*$_8$) δ 9.74 (s, 1H), 8.25 (dd, *J* = 7.7, 1.5 Hz, 1H), 8.10 (dd, *J* = 7.8, 1.5 Hz, 1H), 7.58 (t, *J* = 7.7 Hz, 1H), 7.20 – 7.17 (m, 1H), 6.69 – 6.59 (m, 2H). **^{13}C NMR** (151 MHz, THF-*d*$_8$) δ 191.3, 167.5, 162.0 (d, *J* = 242.4 Hz), 157.4 (d, *J* = 6.8 Hz), 137.3, 136.4 (d, *J* = 7.8 Hz), 134.5, 130.6 (d, *J* = 10.4 Hz), 128.9, 111.8 (d, *J* = 2.9 Hz), 106.8 (d, *J* = 22.8 Hz). **^{19}F NMR** (565 MHz, THF-*d*$_8$) δ –(114.69–114.75) (m). Some ^{13}C signals for this isomer were too weak to be observed.

2'-Fluoro-6'-hydroxy-[1,1'-biphenyl]-2,6-dicarboxylic acid (S3k)

Reported data is for lithium 2'-fluoro-6'-oxido-[1,1'-biphenyl]-2,6-dicarboxylate (**S3k′**)

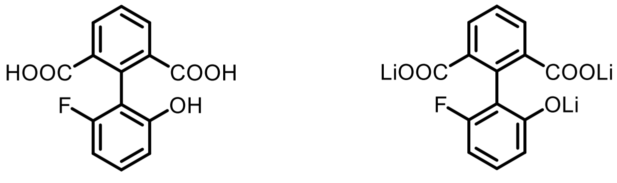

S3k **S3k'**

A solution of methyl 1-fluoro-6-oxo-6H-benzo[c]chromene-10-carboxylate **S3d** (1.0 eq., 10.9 mg, 40.0 µmol) and LiOH (24 eq., 23.0 mg, 0.960 mmol) in THF-d_8 (0.60 mL) and D_2O (1.3 mL) was stirred at 70 °C for 1.25 h. The reaction mixture was allowed to cool to rt and analysed without further purification.

***R*f** (after acidifying, EtOAc) 0.00. **^{1}H NMR** (600 MHz, D_2O/THF-d_8) δ 7.36 – 7.31 (m, 2H), 7.27 (ddd, *J* = 8.4, 6.8, 1.8 Hz, 1H), 6.92 (qd, *J* = 8.1, 1.8 Hz, 1H), 6.32 (dt, *J* = 8.4, 1.5 Hz, 1H), 6.20 – 6.14 (m, 1H), 3.24 (d, *J* = 1.7 Hz, 3H). **^{13}C NMR** (151 MHz, D_2O/THF-d_8) δ 178.7, 167.5 (d, *J* = 6.3 Hz), 161.9 (d, *J* = 239.5 Hz), 142.5, 128.6 (d, *J* = 12.4 Hz), 128.4, 127.4, 126.7, 117.6 (d, *J* = 14.2 Hz), 115.4, 100.4 (d, *J* = 23.4 Hz), 49.3. **^{19}F NMR** (565 MHz, D_2O/THF-d_8) δ −112.4 (t, *J* = 8.5 Hz). **IR** *ν* (cm^{-1}): 3669, 2970, 2918, 1578, 1406, 1257, 1063, 866. **HRMS** (nanospray ESI)$^{-}$ *m/z*: Calculated for $C_{14}H_6Li_2O_5F$ 287.0519; Found $[M-Li]^{-}$ 287.0527.

Due to the method of preparation of **S3k**/**S3k′** described above, there is one equivalent of MeOH present in the sample as observed by ^{1}H and ^{13}C NMR (^{1}H: 3.24 ppm; ^{13}C: 49.3 ppm). Evidence that the methyl ester has undergone hydrolysis to **S3k**/**S3k′** is provided by 1) the lack of a correlation by HMBC between ^{1}H: 3.24 ppm and ^{13}C: 178.7 ppm, and 2) non-equivalence of Me environments in the ^{1}H NMR spectra for **S3d** and **S3k**/**S3k′** in D_2O/THF-d_8.

2.5) Mosher's Ester derivative of 4a, 6a

(2'-Fluoro-6-formyl-6'-hydroxy-[1,1'-biphenyl]-2-yl)methyl (2*S*)-3,3,3-trifluoro-2-methoxy-2-phenylpropanoate (6a)

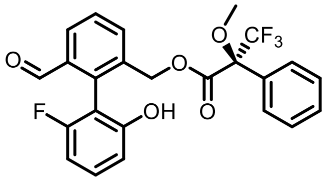

(*R*)-(−)-α-Methoxy-α-(trifluoromethyl)phenylacetyl chloride (1.5 eq., 60 µL, 0.30 mmol) was added to a stirred solution of 2'-fluoro-6'-hydroxy-6-(hydroxymethyl)-[1,1'-biphenyl]-2-carbaldehyde **4a** (1.0 eq., 50 mg, 0.20 mmol), pyridine (3.0 eq., 50 µL, 0.61 mmol) and DMAP (0.1 eq., 2.0 mg, 20 µmol) in CH_2Cl_2 (2 mL) at room temperature. After 1 h 20 min, H_2O (5 mL) and CH_2Cl_2 (5 mL) were added, the layers separated and the aqueous layer was extracted with CH_2Cl_2 (2 × 5 mL). The combined organic phases were combined, dried over $MgSO_4$, filtered, and the solvent removed under reduced pressure. The resulting residue was purified by flash column chromatography on silica gel (petroleum ether:EtOAc, gradient 100:0–60:40), followed by further purification by preparatory HPLC (hexane:EtOAc, gradient 70:30–40:60), to provide the title compound (29 mg, 60 µmol, 31%) as a colourless oil.

The NMR data show two diastereomeric conformers. **^{1}H NMR** (500 MHz, $(CD_3)_2SO$) δ 10.28 (s, 1H, single diastereomer), 10.28 (s, 1H, single diastereomer), 9.67 (br s, 1H, single diastereomer), 9.67 (br s, 1H, single diastereomer), 7.93 (dd, *J* = 7.7, 1.4 Hz, 2H, both diastereomers), 7.81 – 7.76 (m, 2H, both diastereomers), 7.64 (t, *J* = 7.7 Hz, 2H, both diastereomers), 7.50 – 7.41 (m, 6H, both diastereomers), 7.37 – 7.33 (m, 6H, both diastereomers), 6.86 (br d, *J* = 1.2 Hz, 2H, both diastereomers), 6.81 (ddd, *J* = 9.2, 8.3, 0.9 Hz, 1H, single diastereomer), 6.78 – 6.73 (m, 1H, single diastereomer), 5.30 – 5.14 (m, 4H, both diastereomers), 3.38 (br s, 3H, single diastereomer), 3.37 (br s, 3H, single diastereomer). **^{13}C NMR** (126 MHz, $(CD_3)_2SO$) δ 191.5, 165.5 (d, *J* = 2.0 Hz), 160.0 (dd, *J* = 241.6, 4.6 Hz), 156.2 (d, *J* = 6.7 Hz), 135.0 (d, *J* = 3.3 Hz), 134.8, 134.8 – 134.6 (m), 134.6, 134.3, 131.4, 131.1 (d, *J* = 2.9 Hz), 131.0 (d, *J* = 2.9 Hz), 129.9 (d, *J* = 2.9 Hz), 128.9, 128.6, 127.8, 127.1 (d, *J* = 7.8 Hz), 123.1 (q, *J* = 287.7 Hz), 111.6 (d, *J* = 2.5 Hz), 111.6 (d, *J* = 2.5 Hz), 109.3 (d, *J* = 2.6 Hz), 109.1 (d, *J* = 2.8 Hz), 105.9 (d, *J* = 22.1 Hz), 65.1, 55.2 (d, *J* = 3.9 Hz). **^{19}F NMR** (376 MHz, $(CD_3)_2SO$) δ −(71.22 – 71.37) (m), −112.86 (t, *J* = 8.3 Hz), −112.97 (t, *J* = 8.3 Hz). **IR** *ν* (cm^{-1}): 3991, 2926, 2852, 1727, 1451, 1267. **HRMS** $(ESI)^-$ *m/z*: Calculated for $C_{24}H_{17}F_4O_5$ 461.1012; Found $[M-H]^-$ 461.1014.

Also evident are the following peaks tentatively assigned to the diastereomers of a minor (<15%) hemi-acetal isomer: **^{1}H NMR** (500 MHz, $(CD_3)_2SO$) δ 6.23 (dd, *J* = 8.8, 6.0 Hz, 0.12H), 5.57 – 5.39 (m, 0.35H). **^{19}F NMR** (376 MHz, $(CD_3)_2SO$) δ −(111.05 – 111.12) (m).

2.6) Synthesis of Deuterium-Labelled 3a, D_2-3a

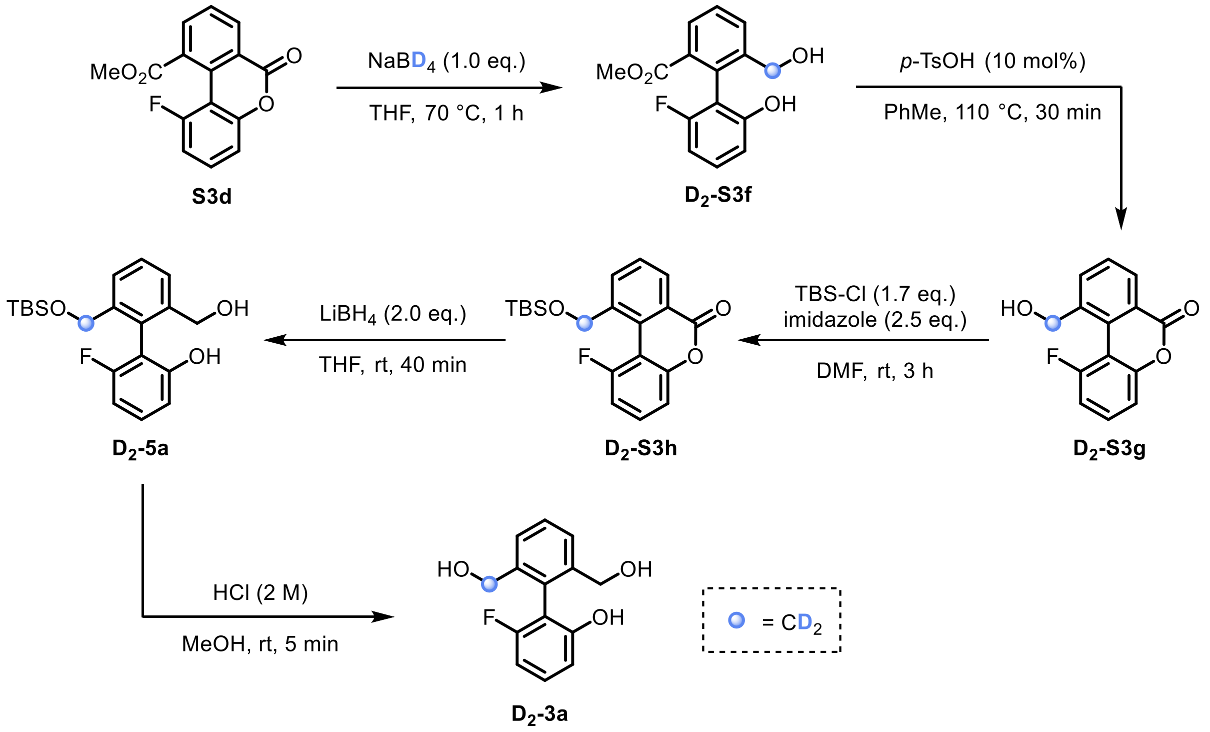

Scheme S3 General synthetic route for the preparation of deuterium labelled **3a**, D_2-**3a**. Full characterisation data is provided for the parent unlabelled compounds. ^{1}H NMR spectroscopy data is provided for isotopically labelled compounds.

Methyl 2'-fluoro-6'-hydroxy-6-(hydroxymethyl)-[1,1'-biphenyl]-2-carboxylate (S3f)

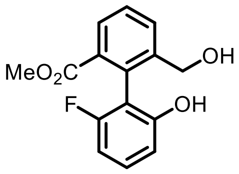

To a solution of methyl 1-fluoro-6-oxo-6H-benzo[c]chromene-10-carboxylate **S3d** (1.0 eq., 220 mg, 0.82 mmol) in THF (4.1 mL) was added sodium borohydride (1.0 eq., 31 mg, 0.82 mmol), and the resulting suspension was stirred at 70 °C for 1.5 h or until TLC monitoring (hexane:EtOAc, 70:30) indicated reaction completion. The mixture was allowed to cool to room temperature, and the reaction was quenched by the dropwise addition of H_2O (4 mL). Et_2O (5 mL) was added, followed by HCl (1 M)

until pH 1 was reached. The organic and aqueous phases were separated, and the aqueous phase was extracted Et_2O (3 × 10 mL). The combined organic phases were dried over Na_2SO_4, filtered, and concentrated under reduced pressure. The resulting residue was purified by flash column chromatography on silica gel (hexane:EtOAc, 70:30) to provide the title compound (160 mg, 0.59 mmol, 72%) as a colourless solid.

^{1}H NMR (400 MHz, CD_3OD) δ 7.88 – 7.81 (m, 2H), 7.51 (t, *J* = 7.8 Hz, 1H), 7.19 (td, *J* = 8.2, 6.6 Hz, 1H), 6.69 (d, *J* = 8.2 Hz, 1H), 6.63 (dt, *J* = 9.1, 0.9 Hz, 1H), 4.39 (ABq, $\Delta\delta_{AB}$ = 0.04, J_{AB} = 17.4 Hz, 2H), 3.59 (s, 3H). **^{13}C NMR** (101 MHz, CD_3OD) δ 169.5, 161.6 (d, *J* = 240.9 Hz), 156.9 (d, *J* = 7.3 Hz), 143.2, 132.8, 131.8, 130.8, 130.3 (d, *J* = 10.7 Hz), 129.6, 128.9, 115.4 (d, *J* = 20.1 Hz), 111.9 (d, *J* = 2.9 Hz), 107.0 (d, *J* = 22.8 Hz), 62.4, 52.3. **^{19}F NMR** (376 MHz, CD_3OD) δ −116.20 (dd, *J* = 9.1, 6.6 Hz). **IR** *ν* (cm^{-1}): 3367, 1708, 1622, 1461, 1298, 1139, 1004, 756. **HRMS** $(MALDI)^-$ *m/z*: Calculated for $C_{15}H_{12}O_4F$ 275.0725; Found $[M-H]^-$ 275.0726. **mp**: 110–111 °C.

Methyl 2'-fluoro-6'-hydroxy-6-(hydroxymethyl-d$_2$)-[1,1'-biphenyl]-2-carboxylate (D_2-S3f)

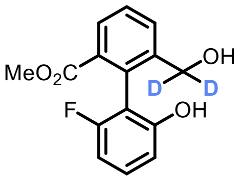

To a solution of methyl 1-fluoro-6-oxo-6H-benzo[c]chromene-10-carboxylate **S3d** (1.0 eq., 487 mg, 1.79 mmol) in THF (8.9 mL) was added sodium borodeuteride (1.0 eq., 75.0 mg, 1.79 mmol), and the resulting suspension was stirred at 70 °C for 1 h or until TLC monitoring (hexane:EtOAc, 70:30) indicated reaction completion. The mixture was allowed to cool to room temperature, and the reaction was quenched by the dropwise addition of H_2O (2 mL). Et_2O (10 mL) was added, followed by HCl (1 M) until pH 1 was reached. The organic and aqueous phases were separated, and the aqueous phase was extracted Et_2O (3 × 15 mL). The combined organic phases were dried over Na_2SO_4, filtered, and concentrated under reduced pressure. The resulting residue was purified by flash column chromatography on silica gel (hexane:EtOAc, 70:30) to provide the title compound (370 mg, 1.33 mmol, 74%, 96% D-incorporation) as a colourless solid.

^{1}H NMR (400 MHz, CD_3OD) δ 7.84 (ddd, *J* = 7.9, 6.6, 1.4 Hz, 2H), 7.50 (t, *J* = 7.8 Hz, 1H), 7.18 (td, *J* = 8.3, 6.7 Hz, 1H), 6.69 (dt, *J* = 8.3, 0.9 Hz, 1H), 6.63 (ddd, *J* = 9.2, 8.2, 1.0 Hz, 1H), 4.37 (d, *J* = 17.2 Hz, 0.09H), 3.59 (s, 3H).

1-Fluoro-10-(hydroxymethyl)-6H-benzo[c]chromen-6-one (S3g)

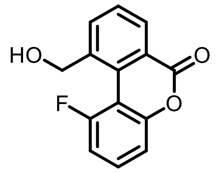

A solution of methyl 2'-fluoro-6'-hydroxy-6-(hydroxymethyl)-[1,1'-biphenyl]-2-carboxylate **S3f** (1.0 eq., 150 mg, 0.53 mmol) and *p*-toluenesulfonic acid monohydrate (10 mol%, 10 mg, 53 µmol) in toluene (106 mL) was heated to reflux and stirred for 30 min or until TLC monitoring (hexane:EtOAc, 60:40) indicated reaction completion. The mixture was allowed to cool to room temperature, and the solvent was removed under reduced pressure. The resulting residue was purified by flash column chromatography on silica gel (hexane:EtOAc, 75:25) to provide the title compound (110 mg, 0.47 mmol, 88%) as a colourless solid.

$\boldsymbol{R_f}$ (hexane:EtOAc, 7:3): 0.18. **^{1}H NMR** (400 MHz, $CDCl_3$) δ 8.33 (dd, *J* = 7.8, 1.5 Hz, 1H), 8.17 (ddt, *J* = 7.8, 1.5, 0.7 Hz, 1H), 7.67 (t, *J* = 7.7 Hz, 1H), 7.46 (td, *J* = 8.3, 5.9 Hz, 1H), 7.21 (dt, *J* = 8.3, 1.1 Hz, 1H), 7.06 (ddd, *J* = 11.5, 8.3, 1.2 Hz, 1H), 4.91 (t, *J* = 6.1 Hz, 2H), 1.92 (td, *J* = 6.2, 1.4 Hz, 1H). **^{13}C NMR** (101 MHz, $CDCl_3$) δ 161.0, 158.6 (d, *J* = 251.3 Hz), 151.8 (d, *J* = 6.3 Hz), 139.7, 135.8, 130.7 (d, *J* = 11.2 Hz), 129.6, 129.4, 129.1, 122.9, 113.5 (d, *J* = 3.2 Hz), 111.9 (d, *J* = 24.8 Hz), 108.4 (d, *J* = 17.6 Hz), 63.6 (d, *J* = 29.6 Hz). **^{19}F NMR** (377 MHz, $CDCl_3$) δ −107.73 (dq, *J* = 11.8, 6.0 Hz). **IR** *ν* (cm^{-1}): 3445, 2922, 1732, 1619, 1593, 1478, 1434, 1256, 1220, 1045, 791, 727. **HRMS** $(ESI)^+$ *m/z*: Calculated for $C_{14}H_{10}O_3F$ 245.0608; Found $[M+H]^+$ 245.0603. **mp**: 147–149 °C.

1-Fluoro-10-(hydroxymethyl-d$_2$)-6H-benzo[c]chromen-6-one (D$_2$-S3g)

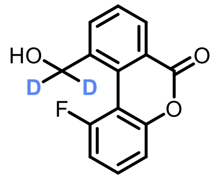

A solution of methyl 2'-fluoro-6'-hydroxy-6-(hydroxymethyl-d$_2$)-[1,1'-biphenyl]-2-carboxylate D_2-**S3f** (1.0 eq., 359 mg, 1.29 mmol) and *p*-toluenesulfonic acid monohydrate (10 mol%, 25.0 mg, 131 µmol) in toluene (260 mL) was heated to reflux and stirred for 30 min or until TLC monitoring (hexane:EtOAc, 60:40) indicated reaction completion. The mixture was allowed to cool to room temperature, and the solvent was removed under reduced pressure. The resulting residue was purified by flash column chromatography on silica gel (hexane:EtOAc, 75:25) to provide the title compound (282 mg, 1.15 mmol, 89%, 96% D-incorporation) as a colourless solid.

^{1}H NMR (400 MHz, $CDCl_3$) δ 8.34 (dd, *J* = 7.8, 1.4 Hz, 1H), 8.17 (dd, *J* = 7.8, 1.5 Hz, 1H), 7.68 (t, *J* = 7.7 Hz, 1H), 7.46 (td, *J* = 8.3, 5.9 Hz, 1H), 7.22 (dt, *J* = 8.3, 1.0 Hz, 1H), 7.07 (ddd, *J* = 11.5, 8.3, 1.2 Hz, 1H), 4.90 (s, 0.08H), 1.87 (s, 1H).

10-(((*tert*-Butyldimethylsilyl)oxy)methyl)-1-fluoro-6H-benzo[c]chromen-6-one (S3h)

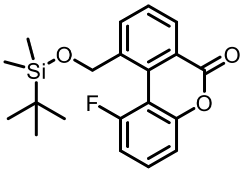

A solution of 1-fluoro-10-(hydroxymethyl)-6H-benzo[c]chromen-6-one **S3g** (1.0 eq., 100 mg, 0.42 mmol), imidazole (2.5 eq., 72 mg, 0.64 mmol), and TBS-Cl (1.5 eq., 96 mg, 1.1 mmol) in DMF (0.4 mL) was stirred at room temperature for 2 h or until TLC monitoring (hexane:EtOAc, 70:30) indicated reaction completion. The reaction was quenched by addition of NH_4Cl (aq. sat., 2 mL). H_2O (10 mL) and Et_2O (15 mL) were added, the aqueous and organic phases were separated, and the aqueous layer was extracted with Et_2O (3 × 15 mL). The combined organic phases were washed with H_2O (20 mL), dried over Na_2SO_4, filtered, and concentrated under reduced pressure. The resulting residue was purified by flash column chromatography on silica gel (hexane:EtOAc, gradient 100:0–98:2) to provide the title compound (150 mg, 0.40 mmol, 95%) as a colourless solid.

***R*f** (hexane:EtOAc, 70:30): 0.69. **^{1}H NMR** (400 MHz, $CDCl_3$) δ 8.34 (dd, *J* = 7.7, 1.5 Hz, 1H), 8.12 (ddt, *J* = 7.8, 1.7, 0.9 Hz, 1H), 7.65 (t, *J* = 7.7 Hz, 1H), 7.46 (td, *J* = 8.3, 5.8 Hz, 1H), 7.23 (dt, *J* = 8.3, 1.1 Hz, 1H), 7.06 (ddd, *J* = 11.5, 8.3, 1.2 Hz, 1H), 4.91 (d, *J* = 6.1 Hz, 2H), 0.86 (s, 9H), 0.00 (s, 6H). **^{13}C NMR** (101 MHz, $CDCl_3$) δ 161.2, 158.6 (d, *J* = 252.0 Hz), 151.7 (d, *J* = 6.4 Hz), 140.2, 135.3, 130.5 (d, *J* = 11.0 Hz), 129.2, 129.1, 128.8, 122.7, 113.3 (d, *J* = 3.2 Hz), 111.8 (d, *J* = 24.9 Hz), 108.6 (d, *J* = 18.0 Hz), 63.7 (d, *J* = 32.6 Hz), 25.9, 18.3, −5.3. **^{19}F NMR** (377 MHz, $CDCl_3$) δ −106.61 (dq, *J* = 12.0, 6.0 Hz). **IR** *ν* (cm^{-1}): 2929, 2857, 1747, 1619, 1593, 1478, 1434, 1255, 1221, 1116, 1087, 1037, 838, 790, 777, 725. **HRMS** $(ESI)^+$ *m*/*z*: Calculated for $C_{20}H_{24}O_3FSi$ 359.1473; Found $[M+H]^+$ 359.1465. **mp**: 60–61 °C.

10-(((*tert*-Butyldimethylsilyl)oxy)methyl-d$_2$)-1-fluoro-6H-benzo[c]chromen-6-one (D$_2$-S3h)

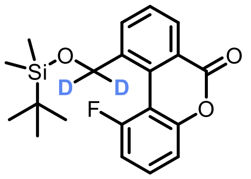

A solution of 1-fluoro-10-(hydroxymethyl-d$_2$)-6H-benzo[c]chromen-6-one D$_2$-**S3g** (1.0 eq., 230 mg, 0.93 mmol), imidazole (2.5 eq., 160 mg, 2.3 mmol), and TBS-Cl (1.7 eq., 230 mg, 1.6 mmol) in DMF (0.9 mL) was stirred at room temperature for 3 h or until TLC monitoring (hexane:EtOAc, 70:30) indicated reaction completion. The reaction was quenched by addition of NH_4Cl (aq. sat., 3 mL). H_2O (15 mL) and Et_2O (20 mL) were added, the aqueous and organic phases were separated, and the aqueous layer was extracted with Et_2O (3 × 20 mL). The combined organic phases were washed with H_2O (20 mL), dried over Na_2SO_4, filtered, and concentrated under reduced pressure. The resulting residue was purified by flash column chromatography on silica gel (hexane:EtOAc, gradient 100:0–98:2) to provide the title compound (330 mg, 0.92 mmol, 99%, 96% D-incorporation) as a colourless solid.

^{1}H NMR (400 MHz, $CDCl_3$) δ 8.34 (dd, *J* = 7.8, 1.5 Hz, 1H), 8.11 (dd, *J* = 7.8, 1.5 Hz, 1H), 7.66 (t, *J* = 7.7 Hz, 1H), 7.46 (td, *J* = 8.3, 5.9 Hz, 1H), 7.23 (dt, *J* = 8.3, 1.1 Hz, 1H), 7.06 (ddd, *J* = 11.5, 8.3, 1.2 Hz, 1H), 4.89 (d, *J* = 6.3 Hz, 0.08H), 0.85 (s, 9H), 0.00 (s, 6H).

(S_a)-2'-(((*tert*-Butyldimethylsilyl)oxy)methyl)-6-fluoro-6'-(hydroxymethyl)-[1,1'-biphenyl]-2-ol ((S_a)-5a)

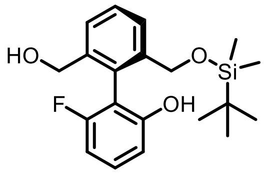

To a solution of borane tetrahydrofuran complex [1.0 M in THF] (4.0 eq., 0.39 mL, 0.39 mmol) in toluene (1.5 mL) at 0 °C was added a solution of (*R*)-(+)-2-methyl-CBS-oxazaborolidine (3.0 eq., 81 mg, 0.29 mmol) in toluene (1.5 mL) dropwise over 10 min. After the addition, the cooling bath was removed, and the mixture was stirred at 30 °C for 30 min. With careful temperature control, a solution of 10-(((*tert*-butyldimethylsilyl)oxy)methyl)-1-fluoro-6H-benzo[c]chromen-6-one **S3h** (1.0 eq., 35 mg, 0.10 mmol) in toluene (3.3 mL) was added dropwise at 30 °C over 25 min. The mixture was stirred at 30 °C for 30 min or until TLC monitoring (hexane:EtOAc, 70:30) indicated reaction completion. The reaction was quenched by the addition of NH_4Cl (aq. sat., 13 mL). H_2O (10 mL) and EtOAc (15 mL) were added, the organic and aqueous phases were separated, and the aqueous layer was extracted with

EtOAc (3 × 20 mL). The combined organic phases were dried over Na_2SO_4, filtered, and concentrated under reduced pressure. The resulting residue was purified by flash column chromatography on silica gel (hexane:EtOAc, gradient 85:15–80:20) to provide the title compound (31 mg, 90 µmol, 88%) as a colourless solid.

***R*$_f$** (hexane:EtOAc, 70:30): 0.26. **^{1}H NMR** (400 MHz, CD_3OD) δ 7.55 – 7.47 (m, 2H), 7.42 (t, *J* = 7.6 Hz, 1H), 7.23 (td, *J* = 8.3, 6.7 Hz, 1H), 6.74 (dt, *J* = 8.2, 0.9 Hz, 1H), 6.67 (ddd, *J* = 9.2, 8.3, 1.0 Hz, 1H), 4.44 (ABq, $\Delta\delta_{AB}$ = 0.03, J_{AB} = 13.7 Hz, 2H), 4.34 (ABq, $\Delta\delta_{AB}$ = 0.02, J_{AB} = 9.2 Hz, 2H), 0.88 (s, 9H), −0.04 (s, 6H). **^{13}C NMR** (101 MHz, CD_3OD) δ 161.7 (d, *J* = 241.5 Hz), 157.2 (d, *J* = 7.2 Hz), 141.5, 141.3, 130.7 (d, *J* = 10.7 Hz), 129.0, 128.8 (d, *J* = 1.6 Hz), 126.3, 126.2, 114.2 (d, *J* = 20.7 Hz), 112.4 (d, *J* = 2.9 Hz), 107.4 (d, *J* = 23.1 Hz), 64.2, 62.9, 26.4, 19.2, −5.41, −5.38. **^{19}F NMR** (377 MHz, CD_3OD) δ −115.09 (dd, *J* = 8.9, 6.7 Hz). **IR** *ν* (cm^{-1}): 3292, 2954, 2929, 2857, 1621, 1460, 1254, 1107, 1004, 835, 782, 731. **HRMS** $(ESI)^+$ *m/z*: Calculated for $C_{20}H_{27}O_3FSiNa$ 385.1606; Found $[M+Na]^+$ 385.1603. **mp**: 76–78 °C. **HPLC** Chiral Regis Whelk O1 250 × 4.6 mm, 5 µm, hexane:IPA = 99:1, flow = 1.15 mL/min, λ = 220 nm, t_R = 15.9, 18.4 min. Enantiomeric ratio: 88.7:11.3 (ee = 77%).

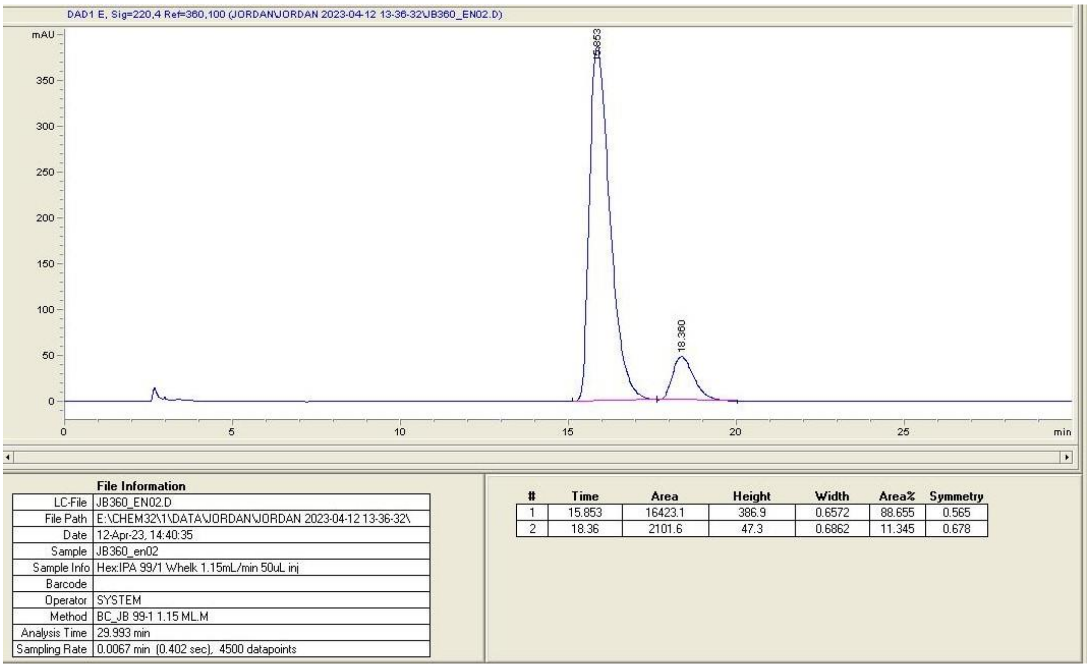

Figure S4 HPLC trace of reduction of **S3h** with (*R*)-(+)-2-methyl-CBS-oxazaborolidine; Chiral Regis Whelk O1 250 × 4.6 mm, 5 µm, hexane:IPA = 99:1, flow = 1.15 mL/min, λ = 220 nm, t_R = 15.9, 18.4 min, enantiomeric ratio: 88.7:11.3.

We assign the major enantiomer (t_R = 15.9 min) as (S_a)-**5a** based on the established sense of enantioselectivity with which Bringmann's 'lactone method' with (*R*)-(+)-2-methyl-CBS-

oxazaborolidine provides enantioenriched biaryl diols.[2,3] We assign the minor enantiomer (t_R = 18.4 min) as (R_a)-**5a**. Establishing the order of elution of the enantiomers of **5a** on the chiral stationary phase Whelk-O1, as described here, allows us to deduce the absolute configurations of the two enantiomers of D_2-**5a** obtained through separation by semi-preparative HPLC, as described directly below, and hence to assign the absolute configuration of the two isotopomeric triols that result from desilylation, (S_a)-D_2-**3a** and (R_a)-D_2-**3a**.

2'-(((*tert*-Butyldimethylsilyl)oxy)methyl-d2)-6-fluoro-6'-(hydroxymethyl)-[1,1'-biphenyl]-2-ol (D_2-5a)

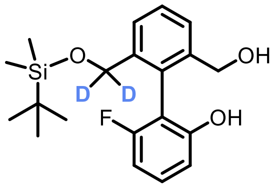

To a solution of 10-(((*tert*-butyldimethylsilyl)oxy)methyl-d_2)-1-fluoro-6H-benzo[c]chromen-6-one D_2-**S3h** (1.0 eq., 44 mg, 0.12 mmol) in THF (1.0 mL), $LiBH_4$ (2.0 eq., 4 M in THF, 0.06 mL, 0.24 mmol) was added dropwise and the reaction mixture was stirred at room temperature for 40 min. The reaction mixture was then cooled to 0 °C and H_2O (0.5 mL) was added dropwise, followed by NH_4Cl (aq. sat., 3 mL). The reaction mixture was then extracted with EtOAc (4 × 10 mL). The organic phases were combined dried over Na_2SO_4, filtered, and concentrated under reduced pressure, to provide the title compound (41 mg, 0.11 mmol, 91%, 96% D-incorporation) as a colourless solid.

^{1}H NMR (400 MHz, CD_3OD) δ 7.55 – 7.50 (m, 2H), 7.44 (t, *J* = 7.6 Hz, 1H), 7.23 (td, *J* = 8.3, 6.7 Hz, 1H), 6.74 (d, *J* = 8.3 Hz, 1H), 6.68 (ddd, *J* = 9.2, 8.3, 0.9 Hz, 1H), 4.38 – 4.30 (m, 2.08H).

Separation of 2'-(((*tert*-Butyldimethylsilyl)oxy)methyl-d2)-6-fluoro-6'-(hydroxymethyl)-[1,1'-biphenyl]-2-ol ((S_a)-D_2-5a and (R_a)-D2-5a)

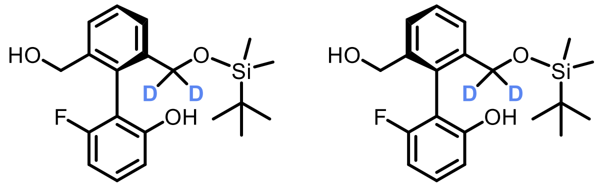

(S_a)-D_2-**5a** (R_a)-D_2-**5a**

A 5 mg/mL solution of racemic 2'-(((*tert*-butyldimethylsilyl)oxy)methyl)-6-fluoro-6'-(hydroxymethyl-d_2)-[1,1'-biphenyl]-2-ol D_2-**5a** in hexane was purified using semi-prep HPLC (Chiral Regis Whelk O1,

250 × 4.6 mm, 5 µm, 99:1 hexane:IPA, flow = 1.15 mL.min^{-1}, λ = 220 nm, t_R ≈ 17 and 19 min). Fractions containing S_a and R_a deutero-enantiomers, identified using the order of elution of the enantiomers of **5a** described above, were isolated separately, and multiple runs under the same conditions led to the isolation of (S_a)-D_2-**5a** (9.83 mg, 99% ee) and of (R_a)-D_2-**5a** (10.63 mg, 98% ee).

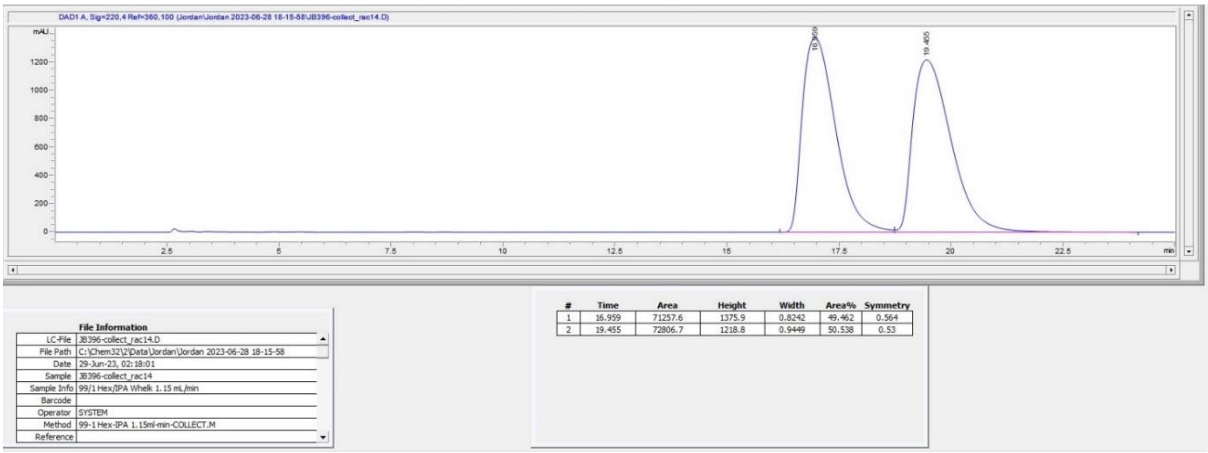

#	Time	Area	Height	Width	Area%	Symmetry
1	16.959	71257.6	1375.9	0.8242	49.462	0.564
2	19.455	72806.7	1218.8	0.9449	50.538	0.53

Figure S5 HPLC trace of racemic D_2-**5a**.

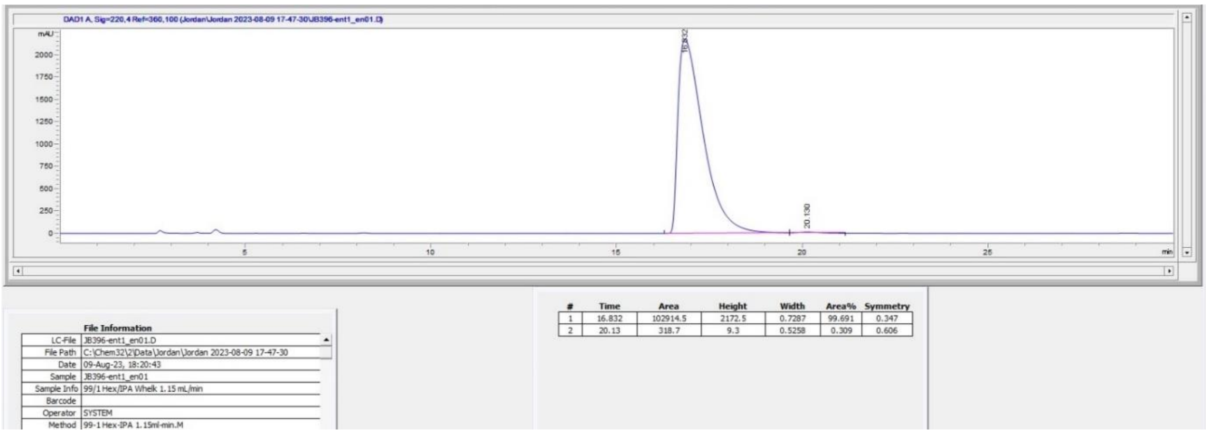

#	Time	Area	Height	Width	Area%	Symmetry
1	16.832	102914.5	2172.5	0.7287	99.691	0.347
2	20.13	318.7	9.3	0.5258	0.309	0.606

Figure S6 HPLC trace of combined fraction of (S_a)-D_2-**5a**.

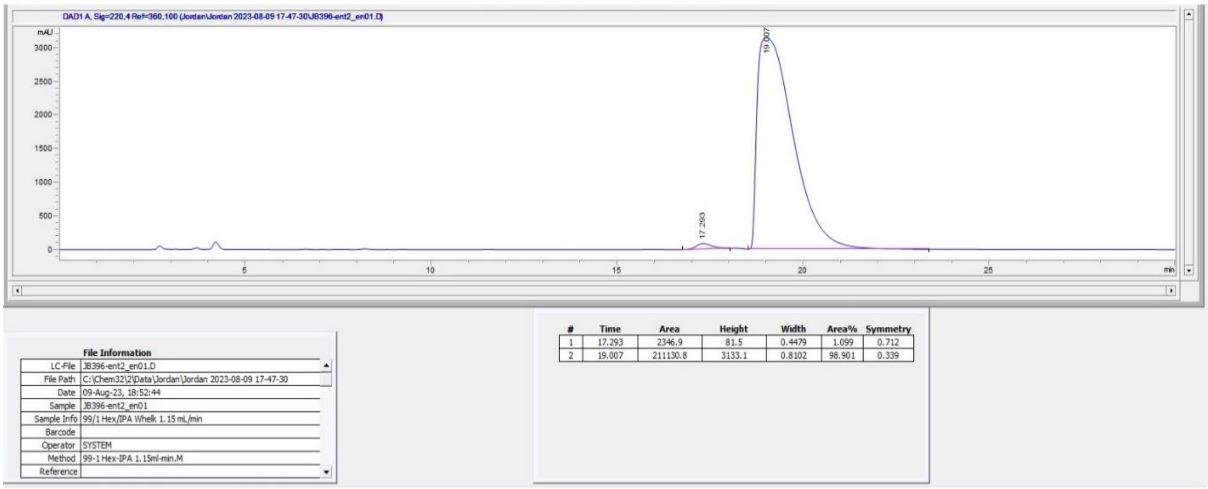

#	Time	Area	Height	Width	Area%	Symmetry
1	17.293	2346.9	81.5	0.4479	1.099	0.712
2	19.007	211130.8	3133.1	0.8102	98.901	0.339

Figure S7 HPLC trace of combined fraction of (R_a)-D_2-**5a**.

(S_a)- or (R_a)-6-Fluoro-2'-(hydroxymethyl)-6'-(hydroxymethyl-d_2)-[1,1'-biphenyl]-2-ol (D_2-3a)

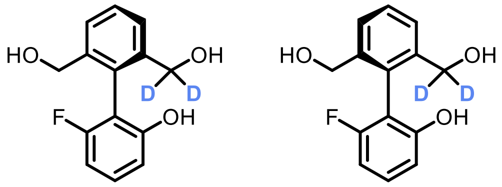

(S_a)-D_2-**3a** (R_a)-D_2-**3a**

The isolated samples of (S_a)-D_2-**5a** (99% ee) and (R_a)-D_2-**5a** (98% ee) were subjected to deprotection to give (S_a)-D_2-**3a** and (R_a)-D_2-**3a**, whose enantioenrichments were assumed to remain unchanged.

Enantioenriched 2'-(((*tert*-butyldimethylsilyl)oxy)methyl)-6-fluoro-6'-(hydroxymethyl-d_2)-[1,1'-biphenyl]-2-ol (S_a)-D_2-**5a** (1.0 eq., 9.8 mg, 27 μmol) was dissolved in MeOH (1.9 mL), and HCl (2 M, 0.1 mL) was added. The colourless reaction mixture was stirred at room temperature for 5 min or until TLC monitoring (hexane:EtOAc, 50:50) indicated reaction completion. The reaction was quenched by addition of $NaHCO_3$ (aq. sat., 1 mL). The organic volatiles were removed under reduced pressure and the remaining aqueous solution was then extracted with EtOAc (3 × 2 mL). The combined organic layers were dried over Na_2SO_4, filtered, and the volatiles were removed under reduced pressure. The residue was purified by flash column chromatography on silica gel (hexane:EtOAc, gradient 70:30–60:40) to provide (S_a)-D_2-**3a** (5.2 mg, 21 μmol, 76%, 95% D-incorporation) as a white solid.

^{1}H NMR (400 MHz, CD_3OD) δ 7.55 – 7.50 (m, 2H), 7.48 – 7.41 (m, 1H), 7.23 (td, J = 8.3, 6.7 Hz, 1H), 6.74 (d, J = 8.2 Hz, 1H), 6.71 – 6.65 (m, 1H), 4.34 (ABq, $\Delta\delta_{AB}$ = 0.02, J_{AB} = 9.6 Hz, 2.11H).

The other enantiomer of deuterated and enantioenriched TBS-protected compound (R_a)-D_2-**5a** (11 mg, 29 μmol) was deprotected using the same procedure to provide the desired deuterated and enantioenriched (R_a)-D_2-**3a** (5.0 mg, 20 μmol, 69%, 95% D-incorporation) as a white solid.

^{1}H NMR (400 MHz, CD_3OD) δ 7.56 – 7.50 (m, 2H), 7.47 – 7.41 (m, 1H), 7.23 (td, J = 8.2, 6.7 Hz, 1H), 6.74 (d, J = 8.2 Hz, 1H), 6.71 – 6.65 (m, 1H), 4.34 (ABq, $\Delta\delta_{AB}$ = 0.02, J_{AB} = 9.6 Hz, 2.11H).

2.7) Synthesis of Deuterium-Labelled D_2-S8e

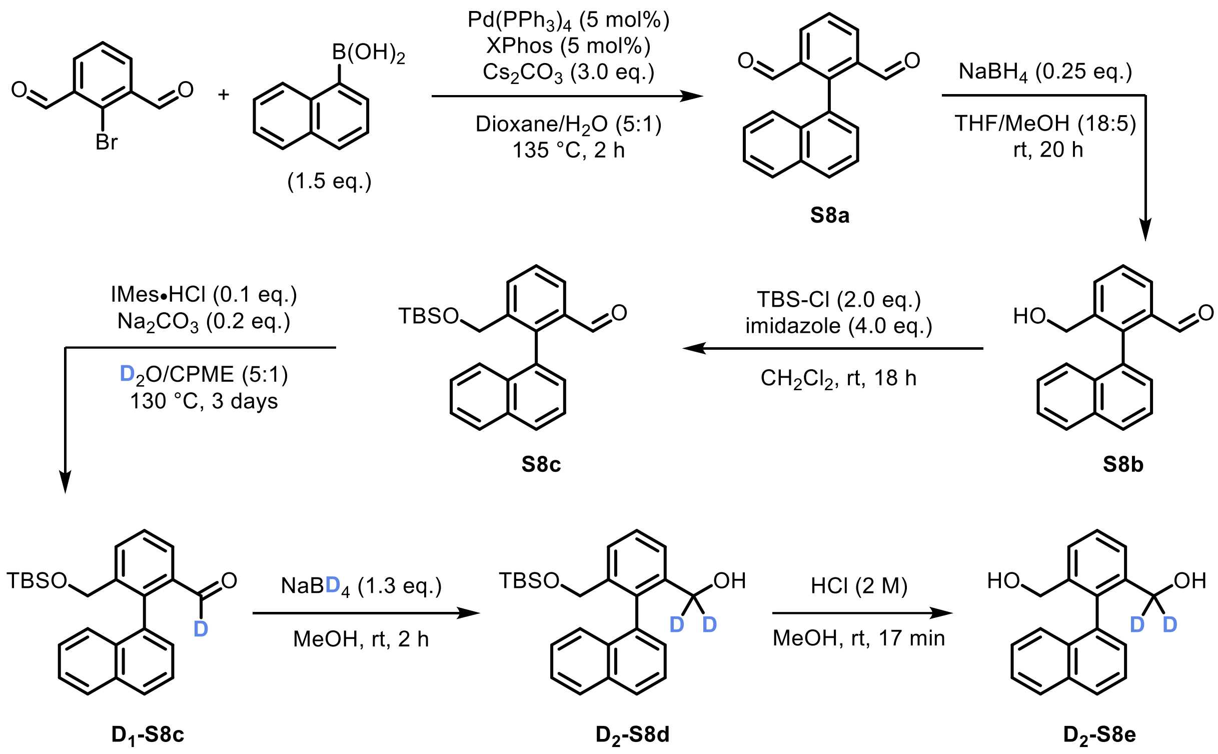

Scheme S4 General synthetic route for the preparation of deuterium labelled **S8e**, D_2-**S8e**. Full characterisation data is provided for the parent unlabelled compounds. ^{1}H NMR spectroscopy data is provided for isotopically labelled compounds.

2-(Naphthalen-1-yl)isophthalaldehyde (S8a)

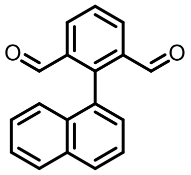

In a microwave vial (10–20 mL), a mixture of 2-bromoisophthalaldehyde (1.0 eq., 300 mg, 1.4 mmol), naphthalene-1-boronic acid (1.5 eq., 360 mg, 2.1 mmol), Cs_2CO_3 (3.0 eq., 1.2 g, 4.2 mmol) and XPhos (5 mol%, 34 mg, 70 µmol) in 1,4-dioxane (15 mL) and H_2O (3 mL) was degassed with N_2 for 5 min. $Pd(PPh_3)_4$ (5 mol%, 81 mg, 70 µmol) was then added and the reaction mixture degassed with N_2 for a further 2 min, after which the vial sealed and heated to 135 °C in the microwave. After 2 h, H_2O (20 mL) was added, and the reaction was extracted with EtOAc (3 × 10 mL). The organic phases were combined, washed with brine (20 mL), dried over $MgSO_4$, filtered and the solvent removed under reduced pressure. The resulting residue was purified by flash column chromatography on silica gel (petroleum

ether:EtOAc, gradient 100:0–85:15) to give the title compound (310 mg, 1.1 mmol, 81%) as a yellow oil.

^{1}H NMR (400 MHz, $CDCl_3$) δ 9.53 (d, *J* = 0.8 Hz, 2H), 8.35 (d, *J* = 7.7 Hz, 2H), 8.03 (dt, *J* = 8.3, 1.2 Hz, 1H), 8.00 – 7.96 (m, 1H), 7.76 (tt, *J* = 7.7, 0.9 Hz, 1H), 7.61 (dd, *J* = 8.3, 7.0 Hz, 1H), 7.58 – 7.53 (m, 1H), 7.48 (dd, *J* = 7.1, 1.3 Hz, 1H), 7.46 – 7.42 (m, 1H), 7.32 – 7.27 (m, 1H).

The spectroscopic data is in accordance with the literature.[7]

3-(Hydroxymethyl)-2-(naphthalen-1-yl)benzaldehyde (S8b)

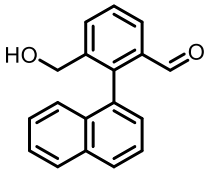

Prepared by a modification to a literature procedure.[5] $NaBH_4$ (25 mol%, 9.0 mg, 0.23 mmol) was added to a stirred solution of 2-(naphthalen-1-yl)isophthalaldehyde **S8a** (1.0 eq., 250 mg, 0.94 mmol) in THF (9 mL) and MeOH (2.5 mL) at room temperature. After 20 h, H_2O (10 mL) was added and the aqueous extracted with EtOAc (3 × 10 mL). The organics were combined, washed with brine (10 mL), dried over $MgSO_4$, filtered and the solvent removed under reduced pressure. The resulting residue was purified by flash column chromatography on silica gel (petroleum ether:EtOAc, gradient 90:10–50:50) giving the title compound (190 mg, 0.73 mmol, 80%) as a colourless solid.

R_f (hexane:EtOAc, 70:30): 0.43. **^{1}H NMR** (400 MHz, $CDCl_3$) δ 9.46 (d, *J* = 0.8 Hz, 1H), 8.05 (dd, *J* = 7.8, 1.4 Hz, 1H), 7.99 – 7.91 (m, 3H), 7.64 (t, *J* = 7.7 Hz, 1H), 7.57 (dd, *J* = 8.3, 7.0 Hz, 1H), 7.55 – 7.49 (m, 1H), 7.44 – 7.37 (m, 2H), 7.29 – 7.26 (m, 1H), 4.39 – 4.24 (m, 2H), 1.45 (t, *J* = 5.9 Hz, 1H).

The spectroscopic data is in accordance with the literature.[7]

3-(((*tert*-Butyldimethylsilyl)oxy)methyl)-2-(naphthalen-1-yl)benzaldehyde (S8c)

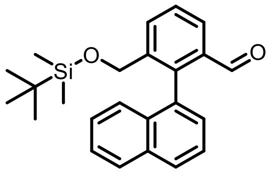

A solution of 3-(hydroxymethyl)-2-(naphthalen-1-yl)benzaldehyde **S8b** (1.0 eq., 191 mg, 0.728 mmol), imidazole (4.0 eq., 198 mg, 2.91 mmol) and *tert*-butyldimethylsilyl chloride (2.0 eq., 219 mg, 1.46 mmol) in CH_2Cl_2 (7 mL) was stirred at room temperature for 18 h. After this time, H_2O (10 mL) was added, the aqueous and organic phases were separated and the aqueous phase was extracted with CH_2Cl_2 (2 × 10 mL). The organic phases were combined, dried over $MgSO_4$, filtered and the solvent was removed under reduced pressure. The resulting residue was purified by flash column chromatography on silica gel (petroleum ether:EtOAc, gradient 100:0–70:30) to give the title compound (232 mg, 0.616 mmol, 85%) as a colourless oil.

***R*f** (hexane:EtOAc, 70:30): 0.83. **^{1}H NMR** (400 MHz, $CDCl_3$) δ 9.43 (d, *J* = 0.8 Hz, 1H), 8.03 – 7.99 (m, 1H), 7.98 – 7.91 (m, 3H), 7.62 (t, *J* = 7.7 Hz, 1H), 7.56 (dd, *J* = 8.3, 7.0 Hz, 1H), 7.53 – 7.48 (m, 1H), 7.41 – 7.34 (m, 2H), 7.30 – 7.26 (m, 1H), 4.36 (d, *J* = 14.0 Hz, 1H), 4.22 (d, *J* = 14.0 Hz, 1H), 0.81 (s, 9H), −0.15 (s, 3H), −0.18 (s, 3H). **^{13}C NMR** (126 MHz, $CDCl_3$) δ 192.3, 141.5, 141.4, 134.9, 133.5, 133.0, 132.7, 132.4, 128.9, 128.6, 128.5, 128.0, 127.0, 126.5, 125.8, 125.7, 125.3, 62.2, 26.0, 18.4, -5.4. **HRMS** (ESI)$^+$ *m/z* Calculated for $C_{24}H_{29}O_2Si$ 377.1931; Found $[M+H]^+$ 377.1934.

3-(((*tert*-Butyldimethylsilyl)oxy)methyl)-2-(naphthalen-1-yl)benzaldehyde-d$_1$ (D$_1$-S8c)

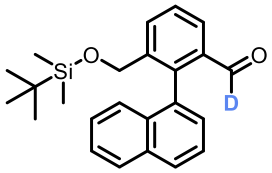

Prepared by a modification to a literature procedure.[5] In a microwave vial, a solution of (2 – 5 mL), 3-(((*tert*-butyldimethylsilyl)oxy)methyl)-2-(naphthalen-1-yl)benzaldehyde **S8c** (1.0 eq., 51 mg, 0.14 mmol, 75% D-incorporation), IMes·HCl (10 mol%, 5.0 mg, 10 µmol) and Na_2CO_3 (20 mol%, 3.0 mg, 30 µmol) in D_2O (0.5 mL) and CPME (0.1 mL) was heated to 130 °C for 3 days. The reaction mixture was cooled to room temperature and extracted with EtOAc (3 × 3 mL). The organic phases were combined, dried over $MgSO_4$, filtered and the solvent was removed under reduced pressure. The resulting residue was purified by flash column chromatography on silica gel (petroleum ether:EtOAc,

gradient 100:0–70:30) to give the title compound (25 mg, 90 µmol, 70%, 93% D-incorporation) as a yellow oil.

***R*$_f$** (hexane:EtOAc, 70:30): 0.83, **^{1}H NMR** (400 MHz, $CDCl_3$) δ 9.43 (d, *J* = 0.9 Hz, 0.07H), 8.04 – 7.99 (m, 1H), 7.98 – 7.90 (m, 3H), 7.62 (t, *J* = 7.7 Hz, 1H), 7.56 (dd, *J* = 8.3, 7.0 Hz, 1H), 7.53 – 7.48 (m, 1H), 7.41 – 7.34 (m, 2H), 7.29 – 7.26 (m, 1H), 4.36 (d, *J* = 13.9 Hz, 1H), 4.22 (d, *J* = 13.9 Hz, 1H), 0.81 (s, 9H), −0.15 (s, 3H), −0.18 (s, 3H). **HRMS** (ESI)$^+$ *m/z* Calculated for $C_{24}H_{28}DO_2Si$ 378.1994; Found [M+H]$^+$ 378.1978.

(3-(((*tert*-Butyldimethylsilyl)oxy)methyl)-2-(naphthalen-1-yl)phenyl)methanol (S8d)

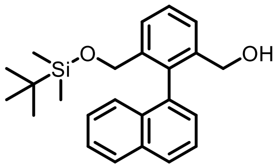

Sodium borohydride (1.3 eq., 36 mg, 0.95 mmol) was added to a stirred solution of 3-(((tert-butyldimethylsilyl)oxy)methyl)-2-(naphthalen-1-yl)benzaldehyde (**S8c**) (1.0 eq., 291 mg, 0.73 mmol) in MeOH (8 mL) at room temperature. After 1 h 30 min, H_2O (20 mL) was added and the reaction was extracted with EtOAc (3 × 20 mL). The organic phases were combined, washed with brine (20 mL), dried over $MgSO_4$, filtered and the solvent removed under reduced pressure. The resulting residue was purified by flash column chromatography on silica gel (petroleum ether:EtOAc, gradient 100:0–50:50) yielding the title compound (281 mg, 0.71 mmol, 96%, 95% purity) as a colourless oil.

^{1}H NMR (500 MHz, $CDCl_3$) δ 7.94 – 7.87 (m, 2H), 7.65 (dd, *J* = 6.6, 2.5 Hz, 1H), 7.56 – 7.51 (m, 3H), 7.51 – 7.46 (m, 1H), 7.38 – 7.33 (m, 1H), 7.32 – 7.27 (m, 2H), 4.32 (d, *J* = 0.8 Hz, 1H), 4.25 – 4.14 (m, 3H), 1.35 (t, *J* = 5.6 Hz, 1H), 0.81 (s, 9H), −0.17 (s, 3H), −0.19 (s, 3H). **^{13}C NMR** (126 MHz, $CDCl_3$) δ 140.4, 139.4, 136.2, 135.6, 133.8, 132.1, 128.5, 128.4, 128.2, 127.1, 126.6, 126.4, 126.2, 126.1, 125.6, 125.4, 63.3, 62.9, 26.0, 18.4, −5.4. **HRMS** (ESI)$^+$ *m/z* Calculated for $C_{24}H_{30}O_2SiNa$ 401.1907; Found [M+Na]$^+$ 401.1914.

(3-(((*tert*-Butyldimethylsilyl)oxy)methyl)-2-(naphthalen-1-yl)phenyl)methan-*d*$_2$-ol (D$_2$-S8d)

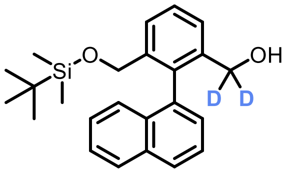

Sodium borodeuteride (1.3 eq., 3.6 mg, 86 µmol) was added to a stirred solution of 3-(((*tert*-butyldimethylsilyl)oxy)methyl)-2-(naphthalen-1-yl)benzaldehyde-d$_1$ (D$_1$-**S8c**) (1.0 eq., 25 mg, 66 µmol) in MeOH (1 mL) at room temperature. After 2 h, H_2O (3 mL) was added, and the aqueous phase was extracted with EtOAc (3 × 5 mL). The organic phases were combined, dried over $MgSO_4$, filtered and the solvent was removed under reduced pressure. The resulting residue was purified by flash column chromatography on silica gel (petroleum ether:EtOAc, gradient 100:0–70:30) to give the title compound (20 mg, 0.050 mmol, 75%, 95% D-incorporation) as a colourless oil.

***R*$_f$** (hexane:EtOAc, 90:10): 0.19. **^{1}H NMR** (400 MHz, $CDCl_3$) δ 7.93 – 7.87 (m, 2H), 7.67 – 7.63 (m, 1H), 7.56 – 7.46 (m, 4H), 7.38 – 7.33 (m, 1H), 7.32 – 7.27 (m, 2H), 4.31 (d, *J* = 13.7 Hz, 1.05H), 4.14 (d, *J* = 13.7 Hz, 1.05H), 1.25 – 1.22 (m, 1H), 0.79 (s, 9H), −0.18 (s, 3H), −0.20 (s, 3H).

Separation of (3-(((*tert*-butyldimethylsilyl)oxy)methyl)-2-(naphthalen-1-yl)phenyl)methan-*d*$_2$-ol (D$_2$-S8d) ((*S*$_a$)-D$_2$-S8d and (*R*$_a$)-D$_2$-S8d)

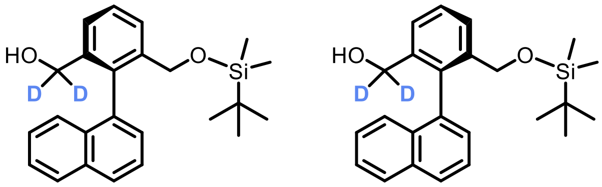

(*S*$_a$)-D$_2$-**S8d** (*R*$_a$)-D$_2$-**S8d**

A 5 mg/mL solution of racemic (3-(((*tert*-butyldimethylsilyl)oxy)methyl)-2-(naphthalen-1-yl)phenyl)methan-*d*$_2$-ol D$_2$-**S8d** in 80:20 hexane:IPA was purified using semi-prep HPLC (CHIRALCEL OD-H, 5 µm, 4.6 × 250 mm, 90:10 hexane:IPA, flow = 1.0 mL.min^{-1}, λ = 254 nm, t_R ≈ 7 and 10 min). Fractions containing *S*$_a$ and *R*$_a$ deutero-enantiomers were isolated separately, and multiple runs under the same conditions allowed the isolation of (*S*$_a$)-D$_2$-**S8d** (6.06 mg, >99% ee) and (*R*$_a$)-D$_2$-**S8d** (6.61 mg, >99% ee).

The order of elution of (*S*$_a$)-D$_2$-**S8d** and (*R*$_a$)-D$_2$-**S8d** was determined by their transformation into (*S*$_a$)-D$_2$-**S8e** and (*R*$_a$)-D$_2$-**S8e** (see below). The configuration of (*S*$_a$)-D$_2$-**S8e** and (*R*$_a$)-D$_2$-**S8e** was

assigned using the ^{1}H NMR-identified location of the deuterium atoms(s) in the products (S_a)-D_1-**S8b** and (S_a)-D_2-**S8b** that arise from their biocatalytic oxidation (see Supplementary Information Section 9). These products D_1- and D_2-**S8b** were assigned (S_a) configuration using the reported stereochemical assignment of the enantiomers of undeuterated **S8b**.[7]

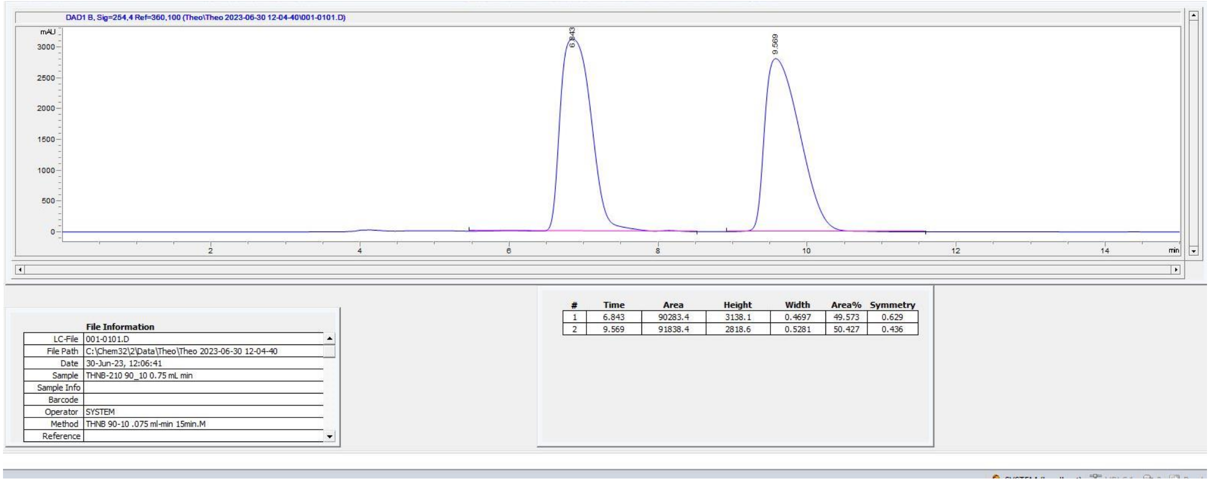

Figure S8 HPLC trace of racemic D_2-**S8d**.

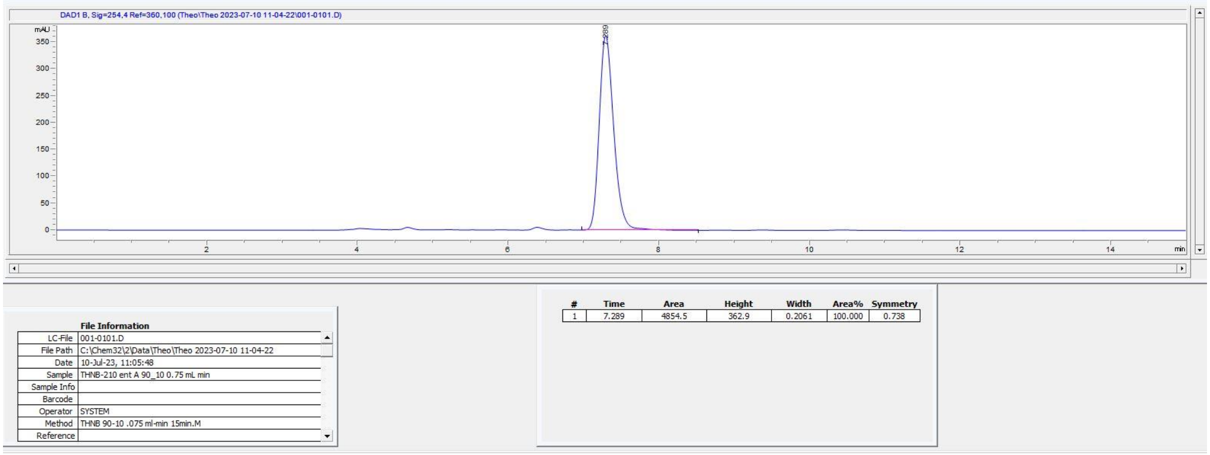

Figure S9 HPLC trace of combined fraction of (S_a)-D_2-**S8d**.

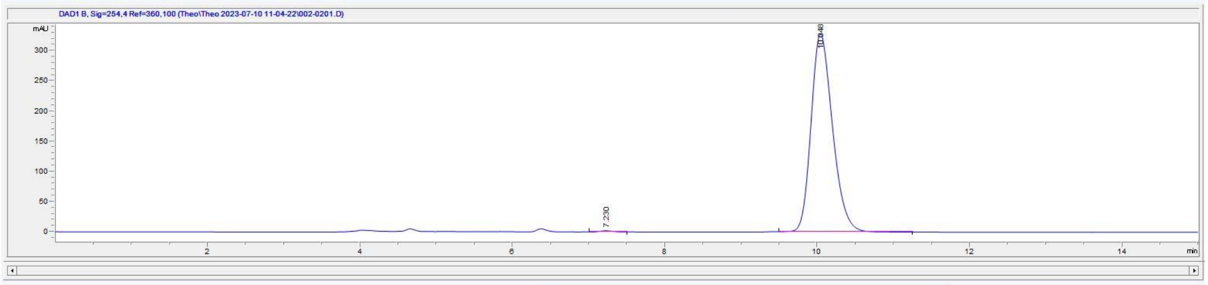

File Information

LC-File	002-0201.D
File Path	C:\Chem32\2\Data\Theo\Theo 2023-07-10 11-04-22
Date	10-Jul-23, 11:22:23
Sample	THNB-210 ent B 90_10 0.75 mL min
Sample Info	
Barcode	
Operator	SYSTEM
Method	THNB 90-10 .075 ml-min 15min.M
Reference	

#	Time	Area	Height	Width	Area%	Symmetry
1	7.23	24.2	2	0.1925	0.378	0.864
2	10.048	6385.3	326.9	0.2999	99.622	0.741

Figure S10 HPLC trace of combined fraction of (R_a)-D_2-**S8d**.

(S_a)- or (R_a)-(3-(hydroxymethyl)-2-(naphthalen-1-yl)phenyl)methan-d_2-ol (D_2-S8e)

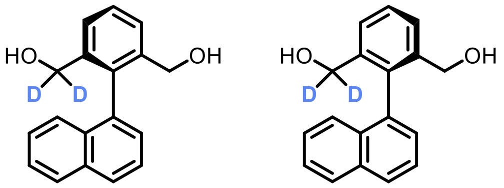

(S_a)-D_2-**S8e** (R_a)-D_2-**S8e**

The isolated samples of (S_a)-D_2-**S8d** (>99% ee) and (R_a)-D_2-**S8d** (>99% ee) were subjected to deprotection to give (S_a)-D_2-**S8e** and (R_a)-D_2-**S8e**, whose enantioenrichments were assumed to remain >99%.

To a stirred solution of enantioenriched (3-(((*tert*-butyldimethylsilyl)oxy)methyl)-2-(naphthalen-1-yl)phenyl)methan-d2-ol (S_a)-D_2-**S8d** (1.0 eq., 6.0 mg, 16 µmol) in MeOH (1.6 mL), was added HCl (2 M, 0.1 mL) at room temperature. After 17 min, $NaHCO_3$ (aq. sat. 3 mL) was added and the reaction mixture extracted with EtOAc (3 × 5 mL). The organic phases were combined, dried over $MgSO_4$, filtered and the solvent removed under reduced pressure. The resulting residue was purified by flash column chromatography on silica gel (petroleum ether:EtOAc, gradient 70:30–0:100) to provide the desired deuterated and enantioenriched (S_a)-D_2-**S8e** (2.5 mg, 9.0 µmol, 58%, 97% purity, 92% D-incorporation) as a colourless oil.

***R*f** (hexane:EtOAc, 70:30): 0.21. **^{1}H NMR** (400 MHz, $CDCl_3$) δ 7.95 – 7.89 (m, 2H), 7.62 – 7.46 (m, 5H), 7.40 – 7.32 (m, 2H), 7.31 – 7.27 (m, 1H), 4.30 – 4.18 (m, 2.16H), 1.35 – 1.28 (m, 2H).

The other enantiomer of the deuterated and enantioenriched TBS-protected compound D_2-**S8d**, (R_a)-D_2-**S8d**, (6.0 mg, 16 μmol) was deprotected using the same procedure to provide the desired deuterated and enantioenriched (R_a)-D_2-**S8e** (4.08 mg, 15 μmol, 92%, 95% purity, 92% D-incorporation) as a colourless oil.

***R*f** (hexane:EtOAc, 70:30): 0.21. **^{1}H NMR** (400 MHz, $CDCl_3$) δ 7.94 – 7.89 (m, 2H), 7.62 – 7.47 (m, 5H), 7.40 – 7.32 (m, 2H), 7.31 – 7.27 (m, 1H), 4.30 – 4.18 (m, 2.17H), 1.38 – 1.30 (m, 2H).

The spectroscopic data of (S_a)-D_2-**S8e** and (R_a)-D_2-**S8e** is consistent with literature for the non-deuterated compound.[7]

Ammonia trideuteroborane $H_3N{\bullet}BH_3$

This compound was prepared following a modified literature procedure.[8] A mixture of sodium borodeuteride (1.0 eq., 420 mg, 10 mmol) and ammonium sulfate (1.0 eq., 1.32 g, 10 mmol) was cooled to 0 °C and THF (10 mL) was added dropwise. Under vigorous stirring, D_2O (0.5 eq., 0.1 mL, 5.0 mmol) was added dropwise over a period of 5 min. After the addition, the cooling bath was removed, and the mixture was stirred at room temperature for 16 h. The reaction mixture was filtered through Celite, and the filter cake was rinsed with THF. The combined filtrates were concentrated under reduced pressure to afford the title compound (206 mg, 6.1 mmol, 61%, > 78% D-incorporation) as a colourless solid.

^{1}H NMR (400 MHz, CD_3CN) δ 3.74 – 3.28 (m, 3H), 1.70 – 0.90 (m, 0.66H). **^{11}B NMR** (128 MHz, CD_3CN) δ −24.09 (s).

Data are consistent with those reported in the literature.[8]

3) General considerations for biocatalytic reactions

Commercially available enzymes and cofactors

Alcohol dehydrogenases (ADH, freeze dried cell lysate), NAD, and NADP were obtained from Johnson Matthey as part of their “C=O Reduction” screening kit, kit number: KC120318. ADH 291 is available from Johnson Matthey on request. Powder activity values (U/mg) were obtained directly from Johnson Matthey and used without further verification. Powder activity test conditions: one unit will reduce 1.0 µmol of 2,3-pentanedione at pH 7 at 25 °C in the presence of NADPH (0.1 mM). NADPH depletion was measured spectrophotometrically at 340 nm, extinction coefficient is 6220 $M^{-1}cm^{-1}$.

The ADH enzymes used in this work were ADH 291 and ADH 20 (Johnson Matthey ADH identifiers). The following information about these enzymes was obtained from the suppliers:

Amino acid residue sequence for ADH 291

```
MVDRLKGKVAIVTGGTLGIGLSIVDLYLKEGAKVVFTGRRENVGKKAYQDLGSPKNAKFVVHDAADEE
GWKKLFADTIAEFGKVDILVNNAGIPVVGNVENTDYAQWRQTMDVNLDGVYLGTHYGVINMKNPQSGD
ASIINMSSIFGLVGDPNTFAYCATKGALRIMSKSAAIYCANQDYNLRINTIHPGPIKTPMMDKYQGAE
EMFSQRTKTPMGHVGVPDDIGWLAVYLGSEESKFATGAEFTVDGGFTAQ
```

Two batches of ADH 291 were used in the following studies, with batch numbers: E17160 (reported powder activity = 1.4 U/mg) and E17184 (reported powder activity = 2.8 U/mg).

ADH 20 (~27 kDa) is an engineered short-chain dehydrogenase. One batch was used in the following studies, with batch number: E17205 (reported powder activity = 1.1 U/mg)

Plasmid generation, expression and purification of YcnD

The nucleotide sequence encoding YcnD within *Bacillus subtilis* (Uniprot: P94424) was synthesized and subcloned into a pET151-D/TOPO plasmid bearing an N-terminal His_6 tag and a tobacco etch virus (TEV) cleavage site (ENLYFQ) by Thermo Fisher. pET151-YcnD was transformed into *Escherichia coli* T7 Express cells. Cultures were grown to OD_{600} = 0.7 in LB media (37 °C), supplemented with carbenicillin (100 µg/ml) and induced (0.2 mM IPTG) at 16 °C (16 h) before cell pellets were harvested by centrifugation (6000 rpm, 10 min) and resuspended in buffer A (50 mM Tris-HCl, 500 mM NaCl, 10% (v/v) glycerol, pH 8.0) before storing at −20 °C.

Harvested cells were sonicated and the soluble fraction was purified by immobilized metal affinity chromatography (IMAC) via a HiTrap 5 ml HP Ni column (GE Healthcare). Protein was eluted using a linear gradient from 6–100% of buffer B (50 mM Tris-HCl, 500 mM NaCl, 10% (v/v) glycerol, 800 mM imidazole, pH 8.0) as a yellow protein. Eluted protein was further purified by size exclusion chromatography (SEC) using a HiPrep 26/60 Sephacryl S200 column (GE Healthcare) in buffer C (25 mM Tris-HCl, 150 mM NaCl, pH 7.5, 1 mM DTT) before protein concentration to 730 μM.

Amino acid residue sequence for YcnD[9]

```
MHHHHHHGKPIPNPLLGLDSTENLYFQGIDPFTMNEVIKSLTDHRSIRSYTDEPVAQEQL
DQIIEAVQSAPSSINGQQVTVITVQDKERKKKISELAGGQPWIDQAPVFLLFCADFNRAK
IALEDLHDFKMEITNGLESVLVGAVDAGIALGTATAAAESLGLGTVPIGAVRGNPQELIE
LLELPKYVFPLSGLVIGHPADRSAKKPRLPQEAVNHQETYLNQDELTSHIQAYDEQMSEY
MNKRTNGKETRNWSQSIASYYERLYYPHIREMLEKQGFKVEK
```

Biocatalytic reactions

Aqueous sodium phosphate buffers (NaPi) of various concentrations and pH were prepared by dissolving appropriate amounts of NaH_2PO_4 and Na_2HPO_4 in H_2O. The pH was adjusted by addition of small amounts of HCl (1 M) or NaOH (1 M) when necessary. Stock solutions of alcohol dehydrogenase were freshly made every day using NaPi buffer as indicated and were kept on ice when not in use. Stock solutions of NAD(P) were made using NaPi buffer as indicated and could be reused for up to 3 days if stored at +4 °C in the dark when not in use.

All biocatalytic reactions were shaken in a vertical position at 300 rpm using an Eppendorf Thermomixer or in a tilted position at 200 rpm using a New Brunswick Scientific Innova 44 shaking incubator.

4) HPLC Conditions and calibration curves

4.1) Calibration for the deracemization of 1a

CHIRALPAK® IK-3, 3 µm, 4.6 × 250 mm, eluent composition and gradient detailed in Table S3, λ = 270 nm t_R = (S_a)-**1a**, 7.8 min; (R_a)-**1a**, 8.1 min; 2-naphthol, 9.7 min; **2a**, enantiomers and hemiacetal not well separated so integrated over whole 10.1–11.0 min region.

Table S3 Gradient conditions for calibration curves for (S_a)-**1a**, (R_a)-**1a**, 2-naphthol, and **2a** on CHIRALPAK® IK-3, 1 mL/min, 20 min run.

Time (min)	MeCN:H_2O (0.5% Formic Acid)
0	30:70
1	30:70
16	95:5
17	95:5
18	30:70

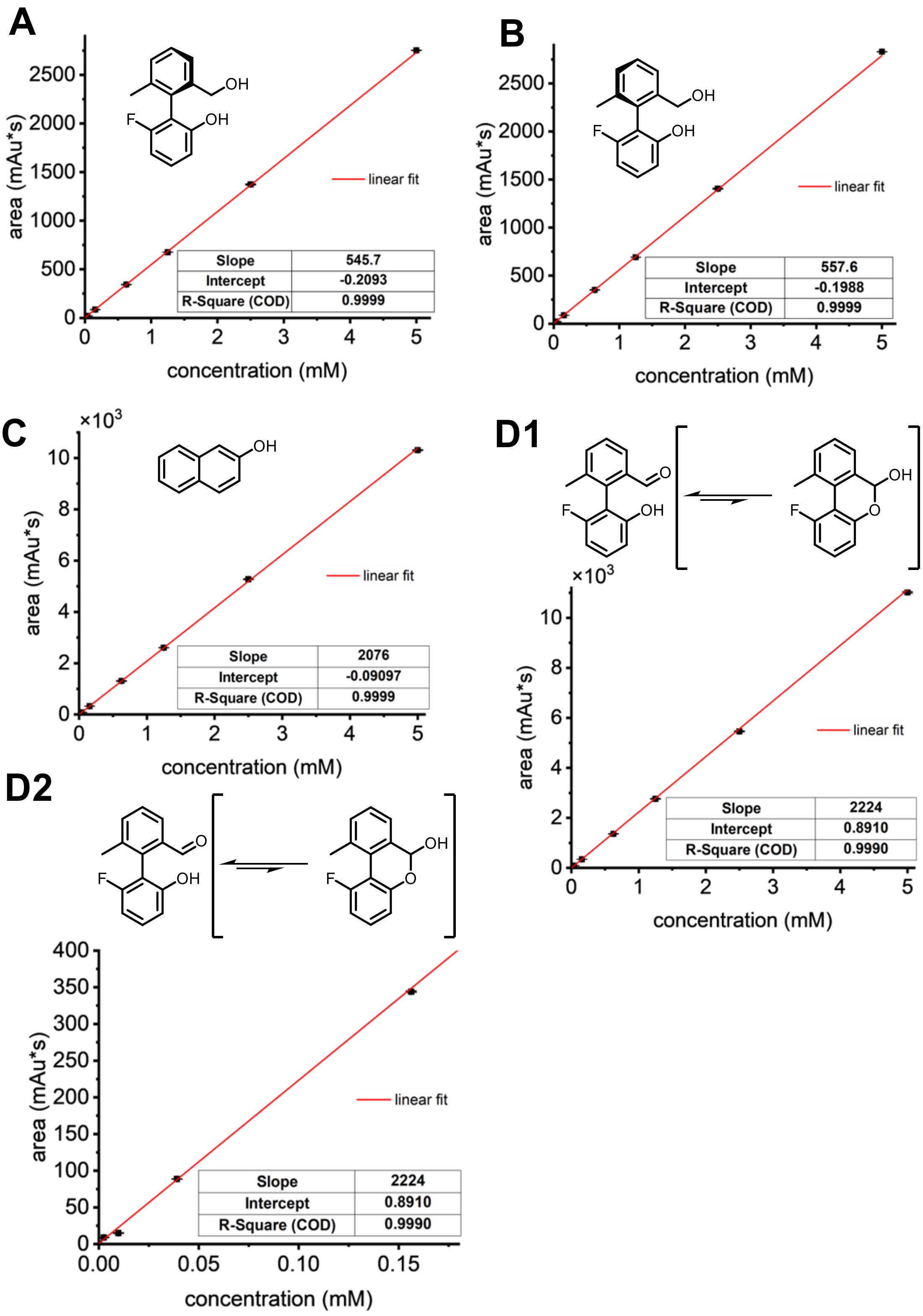

Figure S11 Calibration curves for compounds (**A**): (*S*$_a$)-**1a**; (**B**): (*R*$_a$)-**1a**; (**C**): 2-naphthol; (**D1 and D2**): **2a** on CHIRALPAK® IK-3 (reverse phase).

4.2) Normal phase calibration (operation of 3a)

HiChrom 100-5SIL 250-A, 5 µm, 4.6 × 250 mm, hexane:IPA = 90:10, flow = 1 mL/min, λ = 254 nm, 25 min run, t_R = **S3e**, 8.2 min; **S3g**, 9.8 min; **4a**, 12.7 min; **3a**, 19.5 min.

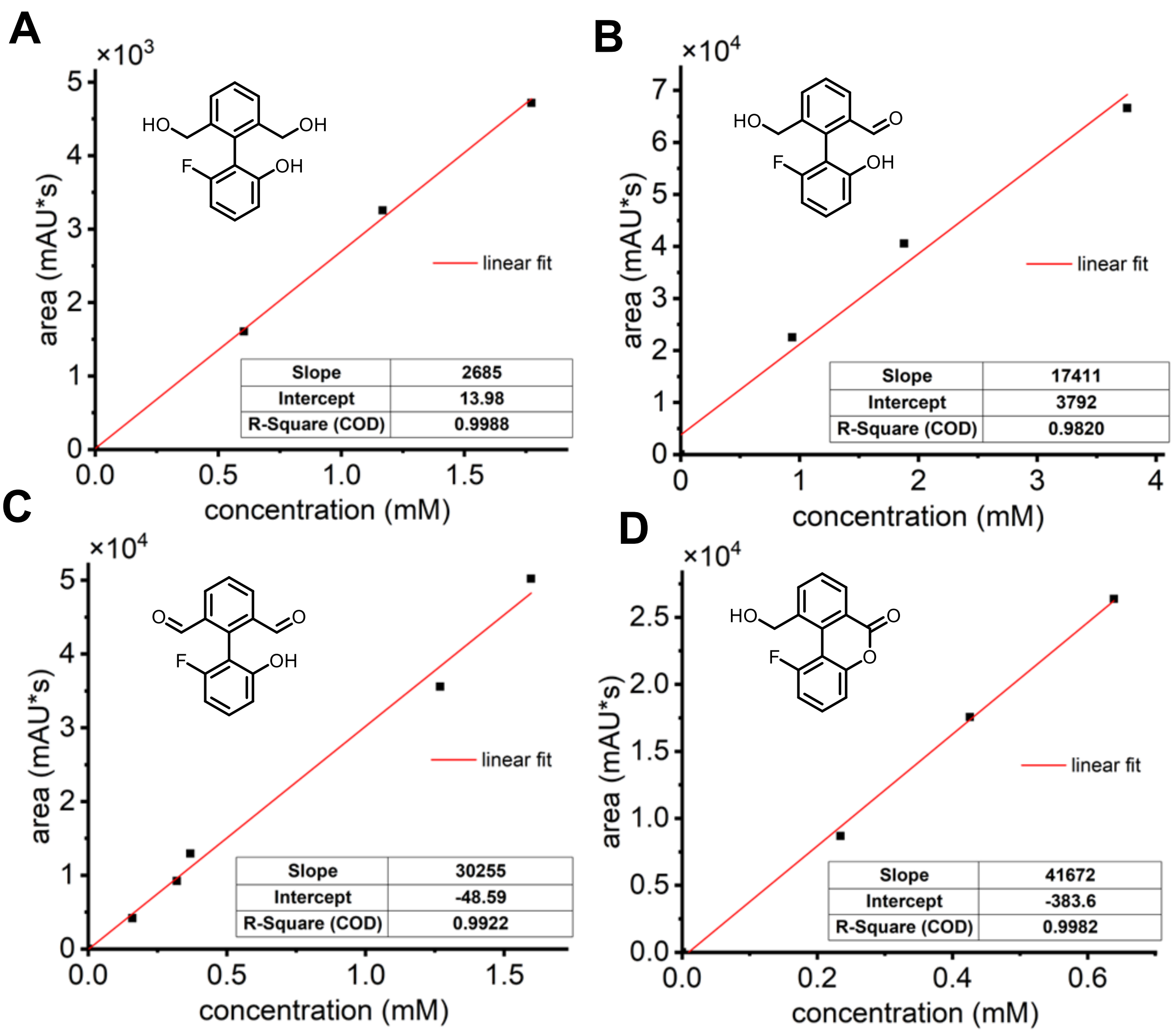

Figure S12 Calibration curves for compounds (**A**): **3a**; (**B**): **4a**; (**C**): **S3e**; (**D**): **S3g** on HiChrom 100-5SIL 250A (normal phase).

4.3) Reverse phase calibration (operation of 3a)

Kromasil 100-5-C18, 5 µm, 4.6 × 250 mm, eluent composition and gradient detailed in Table S4, λ = 270 nm, t_R = **S3k**, 10.2 min; **3a**, 10.7 min; **S3j**, 11.9 min; **4a**, 13.2 min; **S3e**, 15.6 min; 2-naphthol, 16.2 min.

Due to difficulties in purifying **S3k**, the concentration of the resulting solution was determined by the addition of 2-naphthol (2.69 mg, 18.7 µmol) to a 700 µL aliquot of the reaction mixture derived from the hydrolysis of **S3d** detailed above for the preparation of **S3k**. The ratio of 2-naphthol to **S3k** was determined by ^{1}H NMR spectroscopy (400 MHz, 8 scans, D1 = 60 s), using the relative integrals of the peaks of **S3k** and 2-naphthol at 6.25 ppm and 6.69 ppm, respectively. This solution was used for the formulation of HPLC samples for the calibration of **S3k**. The concentrations of **S3k** were determined from the peak area of 2-naphthol, which was quantified from its calibration curve (Figure S13, D), in

combination with the ratio of 2-naphthol to **S3k** determined by ^{1}H NMR spectroscopy, allowing the calibration of **S3k** (Figure S13, F).

Table S4 Gradient conditions for calibration curves for **S3k, 3a, S3j, 4a, S3e,** and 2-naphthol on Kromasil 100-5-C18, 1 mL/min, 26 min run.

Time (min)	MeCN:H_2O (0.5% Formic Acid)
0	5:95
22	95:5
25	95:5
25.2	5:95

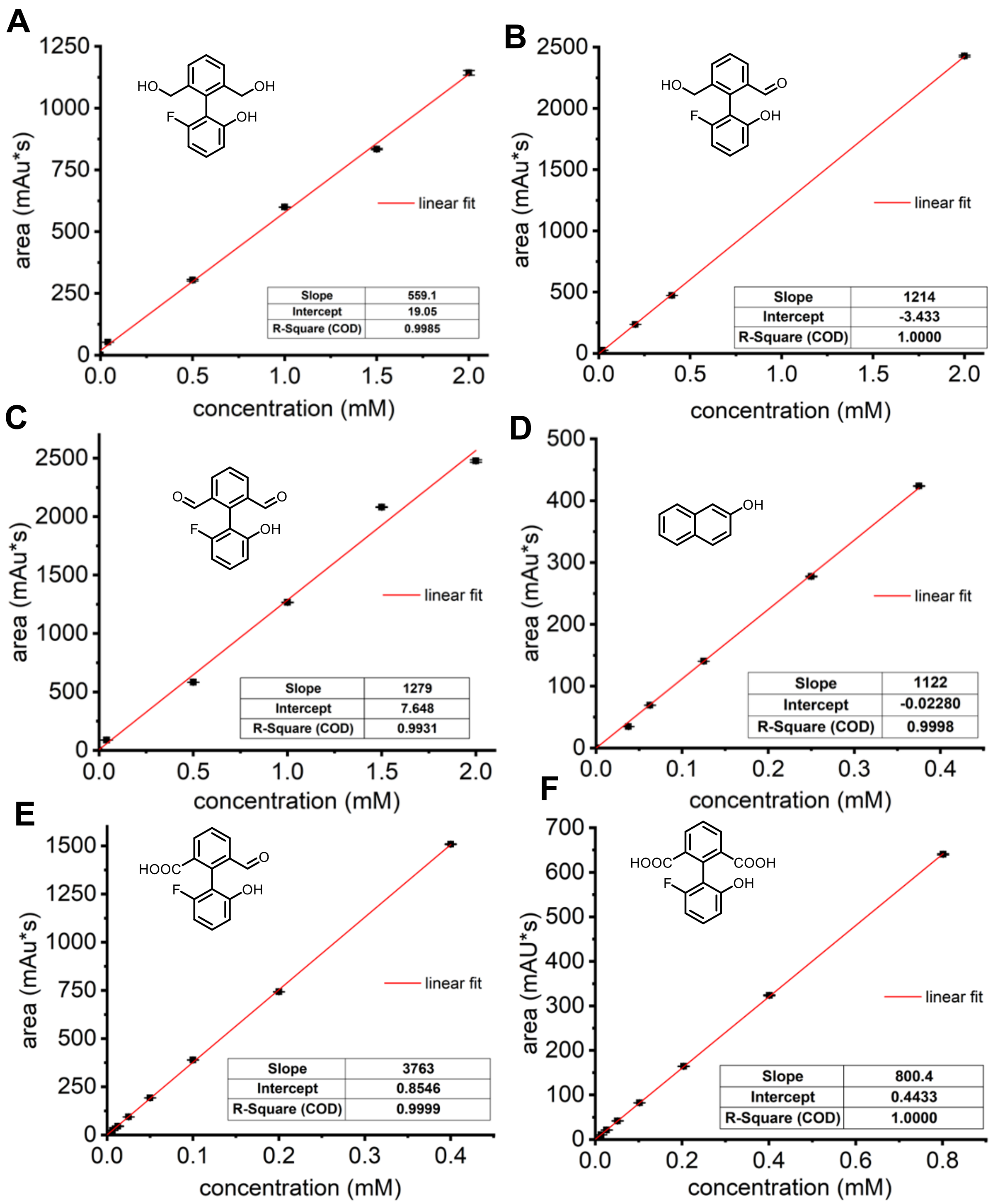

Figure S13 Calibration curves for compounds (**A**): **3a**; (**B**): **4a**; (**C**): **S3e**; (**D**): 2-naphthol (internal standard); (**E**): **S3j**; (**F**): **S3k** on Kromasil 100-5-C18 (reverse phase).

5) Deracemization of 1a

5.1) Optimisation of reaction conditions for the deracemization of 1a

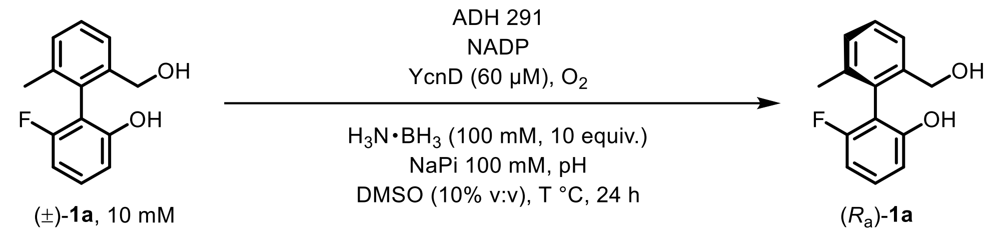

Scheme S5 Screening of conditions for the optimization of the deracemization of **1a** with ADH 291.

In a 2 mL Eppendorf vial were added ADH 291, NADPH oxidase (YcnD (60 µM)), NADP and NaPi (100 mM) and $H_3N{\cdot}BH_3$ (100 mM). To this solution was added substrate **1a** (10 mM final concentration) in DMSO (25 µL). The final volume was 250 µL. The vial was sealed, and the colourless suspension was shaken at 30 or 40 °C. After 24 hours the reaction was treated with a dilutant solution (1.0 mL; composition of dilutant solution: MeCN:H_2O:AcOH:NaOAc (8.00:1.94:0.0600, NaOAc (1.40 mg/mL), with a known concentration of 2-naphthol as an internal standard) and centrifuged for 2 minutes (14800 rpm). The supernatant was then subjected to HPLC analysis (CHIRALPAK® IK-3, 3 µm, 4.6 × 250 mm, eluent composition and gradient detailed in Table S3, λ = 270 nm t_R = **1a**, 7.8 and 8.1 min; 2-naphthol, 9.7 min; **2a**, 10.1–11.0 min) and yields of **1a** were determined by converting the observed absorbance values to concentration using the previously prepared calibration curves (see Supplementary Information Section 4.1) and comparing the ratio of **1a** : 2-naphthol in each solution. Yield and enantiomeric excess data are provided in Table S5.

Table S5 Optimisation of deracemization of **1a** with ADH 291.

Entry	ADH 291 / U/mL	NADP / mM	T / °C	pH	% yield of **1a**	% ee of **1a**
1	3.5	1	30	7.0	100	4
2	3.5	1	40	7.0	99	15
3	3.5	1	40	8.0	100	8
4	7	2	40	7.0	97	83
5	35	5	40	7.0	96	94

5.2) Optimised conditions and evolution of enantiomeric excess and yield over time for the deracemization of 1a

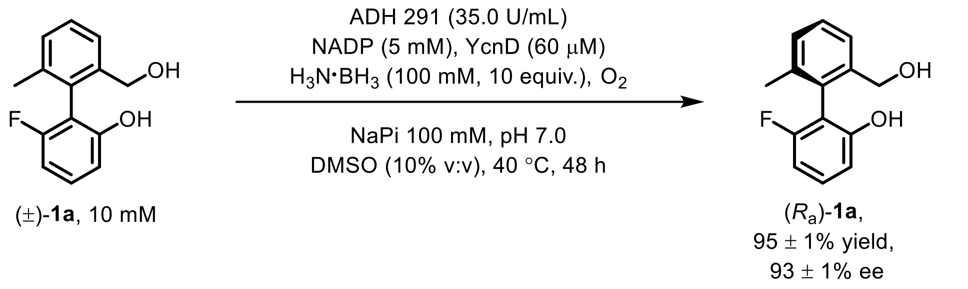

Scheme S6 Optimised conditions for the deracemization of **1a**.

A separate reaction was conducted in triplicate for each timepoint. In a 2 mL Eppendorf vial were added NaPi 100 mM pH 7.0, ADH 291 (35.0 U/mL), YcnD (60 μM), $H_3N{\cdot}BH_3$ (100 mM), and NADP (5 mM). To this solution was added substrate **1a** (10 mM final concentration) in DMSO (25 μL). The final volume was 250 μL. The vial was sealed, and the colourless suspension was shaken at 40 °C. After the required time period (see Table S6 below), the reaction was treated with a dilutant solution (1.0 mL; composition of dilutant solution: MeCN:H_2O:AcOH:NaOAc (8.00:1.94:0.0600, NaOAc (1.40 mg/mL), with a known concentration of 2-naphthol as an internal standard) and centrifuged for 2 minutes (14800 rpm). The supernatant was then subjected to reverse phase HPLC analysis (CHIRALPAK® IK-3, 3 µm, 4.6 × 250 mm, eluent composition and gradient detailed in Table S3, λ = 270 nm, t_R = (S_a)**-1a**, 7.8 min; (R_a)**-1a**, 8.1 min; 2-naphthol, 9.7 min; **2a**, 10.1–11.0 min), yields of **1a** and **2a** were determined by converting the observed absorbance values to concentration using the previously prepared calibration curves (see Supplementary Information Section 4.1) and comparing the concentration of **1a** in each reaction. Yield and enantiomeric excess data are provided in Table S6 and Figure S14 below and corresponds to averages derived from experiments conducted in triplicate.

Table S6 Evolution of yield and enantiomeric excess of **1a** over time during deracemization under optimised conditions. Errors are derived from triplicate reactions, in which the error given is the sample standard deviation between the triplicates.

Time	% yield of **1a**	% yield of **2a**	% ee of **1a**
1	99.2 ± 0.8	0.30 ± 0.01	18.4 ± 1.8
2	99.7 ± 0.8	0.31 ± 0.01	38.5 ± 0.4
5	97.7 ± 0.5	0.32 ± 0.01	73.8 ± 0.4
21	95.9 ± 0.4	0.33 ± 0.01	94.2 ± 0.7
29	95.7 ± 0.1	0.33 ± 0.01	92.8 ± 1.6
48	94.8 ± 1.2	0.32 ± 0.01	93.4 ± 1.4
96	94.6 ± 0.1	0.32 ± 0.01	93.6 ± 1.4

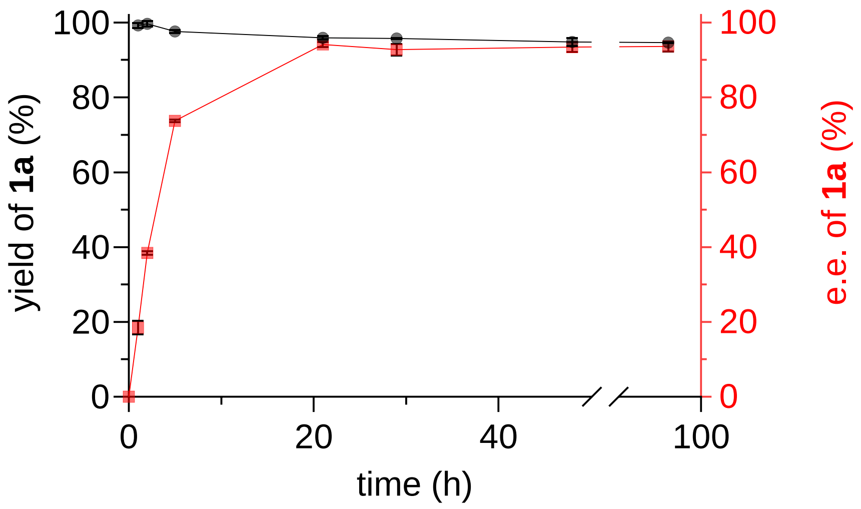

Figure S14 Evolution of yield and enantiomeric excess of **1a** over time during deracemization under optimised conditions. Errors bars are derived from triplicate reactions, in which the error given is the sample standard deviation between the triplicates.

For reference, the chiral reverse phase HPLC chromatogram for the 29 hour timepoint is provided in Figure S15 (CHIRALPAK® IK-3, 3 µm, 4.6 × 250 mm, eluent composition and gradient detailed in Table S3, λ = 270 nm, t_R = NADP, 3.0 min; (*S*a)-**1a**,7.8 min; (*R*a)-**1a**, 8.1 min (yield of **1a**: 95.7%; 93% ee); 2-naphthol, 9.7 min; **2a**, 10.1–11.0 min (0.3%)).

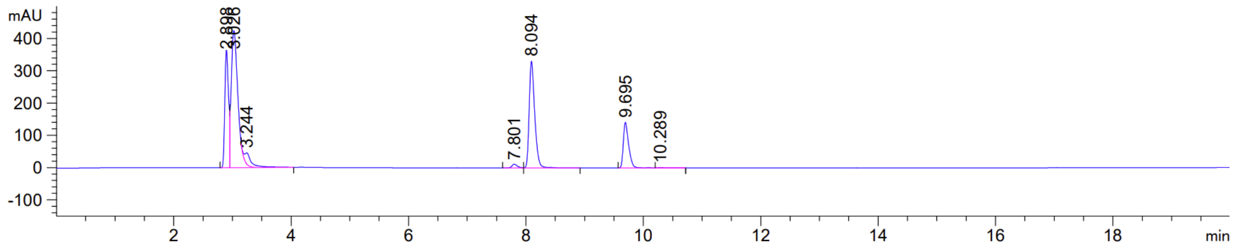

Figure S15 Chiral reverse phase HPLC chromatogram for the optimised deracemization of **1a** at 29 h.

Figure S16 provides a chromatogram of **1a**, potential oxidation products (**S1a**, **2a**, **S1b**), and 2-napththol recorded using the reverse phase HPLC protocol described above for analysis of the deracemization of **1a** (CHIRALPAK® IK-3, 3 µm, 4.6 × 250 mm, eluent composition and gradient detailed in Table S3, λ = 270 nm). The assignment of the peaks in the chromatogram was achieved by independent injections of each component onto the HPLC. Formulation of the combined sample: **1a** (< 1 mg), **S1b** (< 1 mg), **2a** (< 1 mg), **S1b** (< 1 mg) were added to a vial as pure compounds, and dissolved in DMSO (25 µL), NaPi 100 mM pH 8.0 (225 µL), dilutant solution (1 mL).

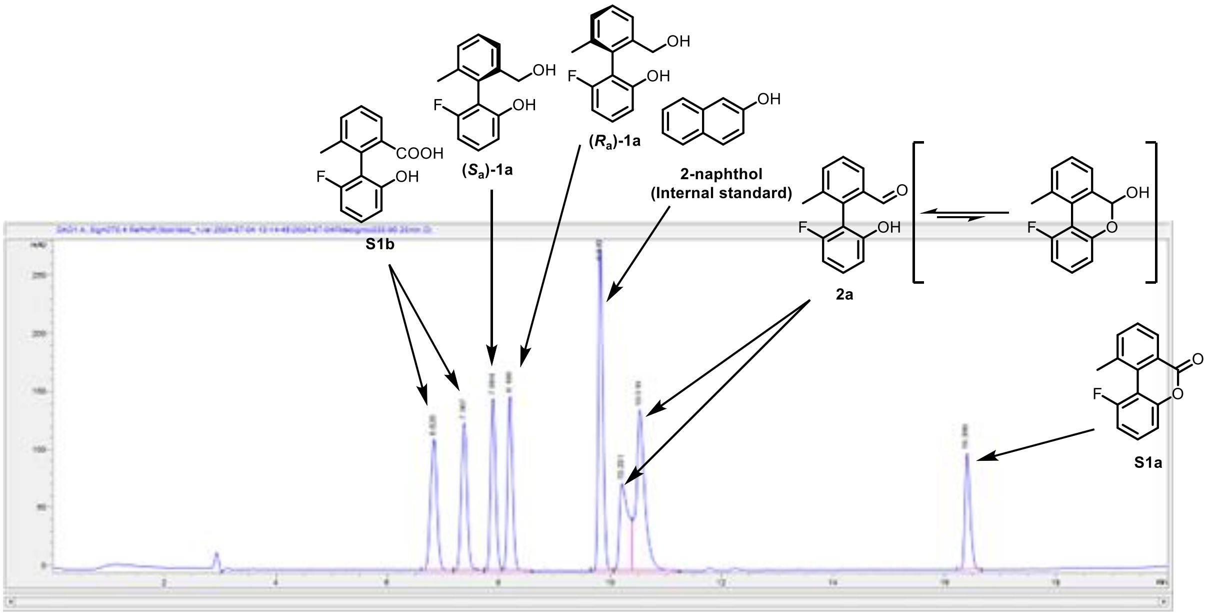

Figure S16: Chromatogram containing **1a**, potential oxidation products (**S1a**, **2a**, **S1b**), and 2-naphthol.

6) Oxidation of 3a

Optimisation of biocatalytic oxidation of 3a

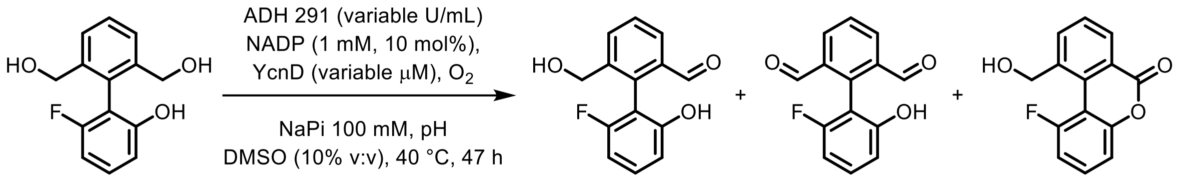

Scheme S7 Further screening of conditions for the optimisation of the biocatalytic oxidation of **3a**.

In a 2 mL Eppendorf vial were added ADH 291, NADPH oxidase (YcnD), NADP (1 mM) and NaPi (100 mM). To this solution was added substrate **3a** (10 mM final concentration) in DMSO (50 μL). The final volume was 500 μL. The vial was sealed, and the colourless suspension was shaken at 40 ºC. After 47 hours aliquots (70 μL) were taken. The aliquots were extracted with MTBE (2 × 300 μL). The organic layers were combined and transferred to a HPLC vial for analysis.

Table S7 Further screening of conditions for the optimisation of the biocatalytic oxidation of **3a**.

Entry	ADH 291 / U/mL	YcnD / μM	pH	% **3a**[a]	% **4a**	% **S3e**	% **S3g**
1	7	12	8.0	62	37	1	0
2	7	12	7.0	64	36	1	0
3[b]	35	60	7.0	42	56	2	0

[a] % composition of **3a**, **4a**, **S3e**, and **S3g** in MTBE extract as determined by HPLC. [b] NADP (5 mM).

7) Kinetic analysis

In order to display continuous directional rotary motion, the operation of **3a** under the optimised conditions of the cyclic redox reaction network must conform to the hierarchy of rates, $r_{enant} > r_{red} > r_{ox(Ra,Sa)}$. To confirm this hierarchy, we determined each rate separately under conditions matching as closely as possible the operating conditions of the motor, as detailed below.

7.1) Determination of the rate of oxidation $r_{ox(Ra,Sa)}$

The rate of oxidation $r_{ox(Ra,Sa)}$ was determined under the operating conditions of the cyclic redox reaction network but in the absence of $H_3N{\cdot}BH_3$ (Scheme S8). The kinetic analysis of the oxidation of **3a** was performed as follows:

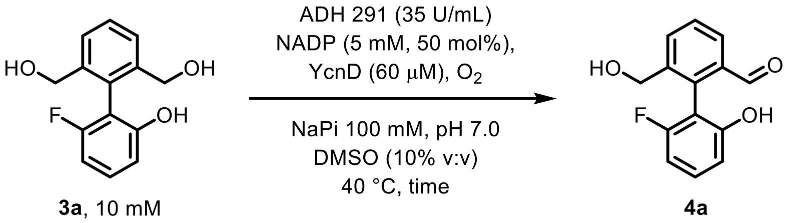

Scheme S8 Optimised conditions for the biocatalytic oxidation of **3a**.

In a 2 mL Eppendorf vial were added preheated (40 °C) solutions of NaPi 100 mM pH 7.0, ADH 291 (35 U/mL), YcnD (60 µM), and NADP (5 mM). To this solution was added substrate (preheated 40 °C, 10 mM final concentration) in DMSO (50 µL). The final volume was 500 µL. The vial was sealed, and the light-yellow suspension was shaken at 40 °C. Aliquots (either 40 µL or 50 µL) were taken at different time points in the initial stages of the reaction and were immediately quenched with 200 µL of diluent (composition detailed in Supplementary Information Section 5.1). Samples were centrifuged for 5 minutes (14800 rpm), and the resulting supernatant was subjected to reverse phase HPLC analysis (Kromasil 100-5-C18, 5 µm, 4.6 × 250 mm, eluent composition and gradient detailed in Table S4, λ = 270 nm, t_R = **3a**, 10.7 min; **4a**, 13.2 min; 2-naphthol, 16.2 min).

Conversion of triol **3a** to aldehyde **4a** over time was determined from the relative integrals of their peaks in the corresponding HPLC traces. The total concentration of reaction components was assumed to remain 10 mM where no further oxidation products (**S3j, S3k, S3e**, **S3g**) were observed.

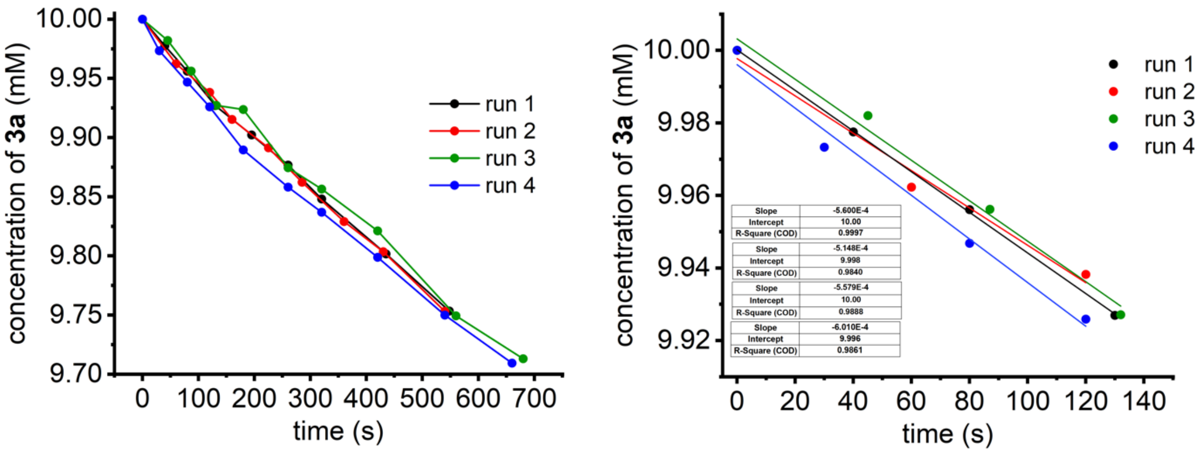

Figure S17 Initial evolution of concentration of **3a** over time during biocatalytic oxidation under the optimised conditions of motor operation. Concentration [**3a**] derived from HPLC assuming total concentration of all components remains 10 mM.

The rate of the oxidation can be seen to be constant over the first 2 min of the oxidation hence the gradient of a linear fit is taken as the rate of oxidation. In the initial stages of the reaction, we consider that [**3a**] and [O_2] are constant and that [**3a**] >> [ADH]. The rate law thus becomes dependent on constant concentrations only and can be reduced to a pseudo-zero order process.

$$initial\ rate \;=\; -k'_{oxidation}$$

where $k'_{oxidation}$ is the experimentally determined pseudo-zero order rate constant.

Table S8 Observed initial rates for the biocatalytic oxidation of **3a**.

	**Initial rate (mM.s^{-1})**	**Corresponding half-life (s)**
Run 1	5.60×10^{-4}	8929
Run 2	5.15×10^{-4}	9709
Run 3	5.58×10^{-4}	8961
Run 4	6.01×10^{-4}	8319
**Average**	5.59×10^{-4}	8945
**Standard deviation**	3.04×10^{-5}	493

The average rate for the biocatalytic oxidation of **3a** in the conditions presented in Scheme S8 is thus $r_{ox(Ra,Sa)} = 5.59 \times 10^{-4}$ mM.s^{-1}. This corresponds to a half-life of oxidation of 2.48 h for a pseudo-zero order process.

7.2) Determination of the rate of reduction r_{red}

The rate of reduction r_{red} of aldehyde **4a** was determined by reducing aldehyde **4a** using a large excess of $H_3N{\cdot}BH_3$ and monitoring the reaction using UV-Vis spectroscopy:

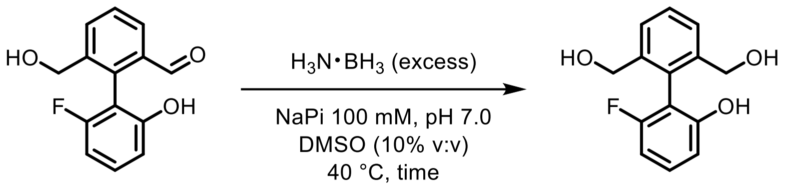

4a, 0.25 mM **3a**

Scheme S9 Reaction conditions for the reduction of **4a**.

Solutions containing aldehyde **4a** (0.25 mM) in NaPi 100 mM pH 7.0 and DMSO (50 µL) were prepared. Each solution was transferred to a transparent 1 cm plastic cuvette and placed in the cuvette port of a Denovix DS-11+ UV-Vis spectrometer pre-heated to 40 °C. The temperature was allowed to equilibrate for at least 5 min before any measurement. Solutions containing a large excess of $H_3N{\cdot}BH_3$ (at least 40 eq.) were added (final volume 500 µL), and the absorbance decay at 296 nm was monitored over time.

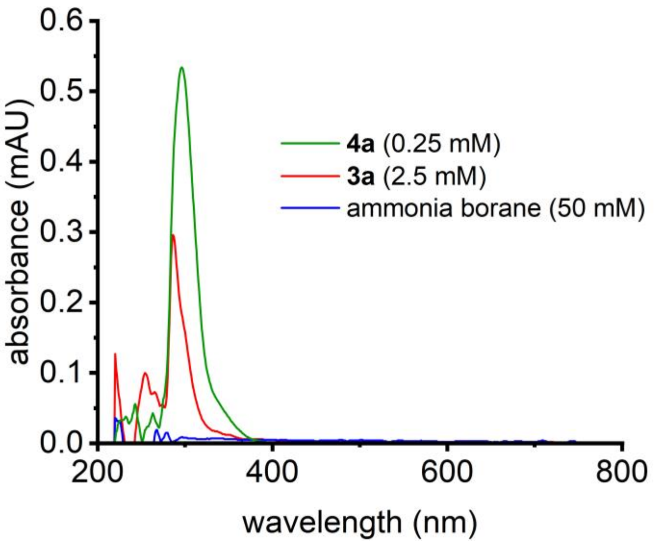

Figure S18 Measured UV Vis spectra of **4a**, **3a** and $H_3N{\cdot}BH_3$ at the indicated concentration. Respective λ_{max} are 296 nm, 286 nm and 220 nm.

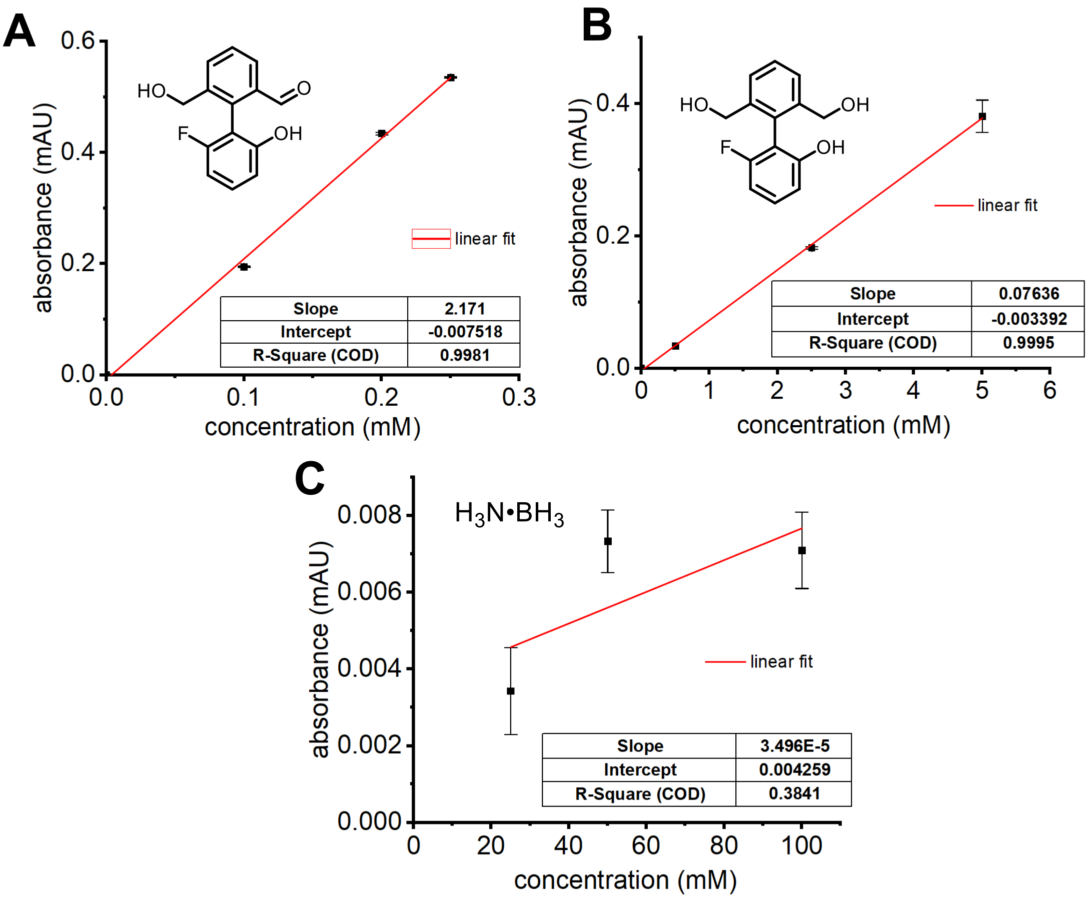

Figure S19 Plots of absorbance at λ = 296 nm as a function of concentration for (**A**): **4a**; (**B**) **3a**; (**C**) $H_3N{\cdot}BH_3$.

Using the Beer-Lambert law and the plots from Figure S19, the molar absorption coefficients ε at λ = 296 nm could be determined for **4a**, **3a** and $H_3N{\cdot}BH_3$:

$\varepsilon_{\mathbf{4a}\ (296\ \mathrm{nm})} = 2.171\ \mathrm{L.mol^{-1}.cm^{-1}}$

$\varepsilon_{\mathbf{3a}\ (296\ \mathrm{nm})} = 0.07636\ \mathrm{L.mol^{-1}.cm^{-1}}$

$\varepsilon_{\mathbf{H3N{\cdot}BH3}\ (296\ \mathrm{nm})} = 0.00003\ \mathrm{L.mol^{-1}.cm^{-1}}$

$H_3N{\cdot}BH_3$ does not absorb in the region of interest and is disregarded in the following analysis.

Concentrations determined from UV-Vis spectroscopic data using the Beer-Lambert law assuming that the observed absorbance at 296 nm is the sum of the individual absorbance of all compounds:

$$A = \varepsilon_{\mathbf{4a}}.l.C_{\mathbf{4a}} + \varepsilon_{\mathbf{3a}}.l.C_{\mathbf{3a}} + C_{4a}Intercept_{4a} + C_{3a}Intercept_{3a}$$

For the reduction of **4a**:

$$C_0 = C_{\mathbf{4a}\,(t)} + C_{\mathbf{3a}\,(t)}$$

And thus:

$$A = \varepsilon_{\mathbf{4a}}.l.C_{\mathbf{4a}\,(t)} + \varepsilon_{\mathbf{3a}}.l.(C_0 - C_{\mathbf{4a}\,(t)}) + C_{\mathbf{4a}\,(t)}(Intercept_{\mathbf{4a}} - Intercept_{\mathbf{3a}}) + C_0(Intercept_{\mathbf{3a}})$$

Rearranging gives, for $l = 1$ cm:

$$C_{\mathbf{4a}\,(t)} = \frac{A - \varepsilon_{\mathbf{3a}}C_0 - C_0 Intercept_{\mathbf{3a}}}{\varepsilon_{\mathbf{4a}} - \varepsilon_{\mathbf{3a}} + Intercept_{4a} - Intercept_{3a}}$$

Reduction kinetic analysis

The obtained absorbance data was converted to concentration using the Beer-Lambert law (Figure S20A) and linearised to fit a pseudo-first order rate equation (Figure S20B).

$$ln([aldehyde]_t) = -k'_{reduction} \times t + ln([\mathbf{4a}]_0)$$

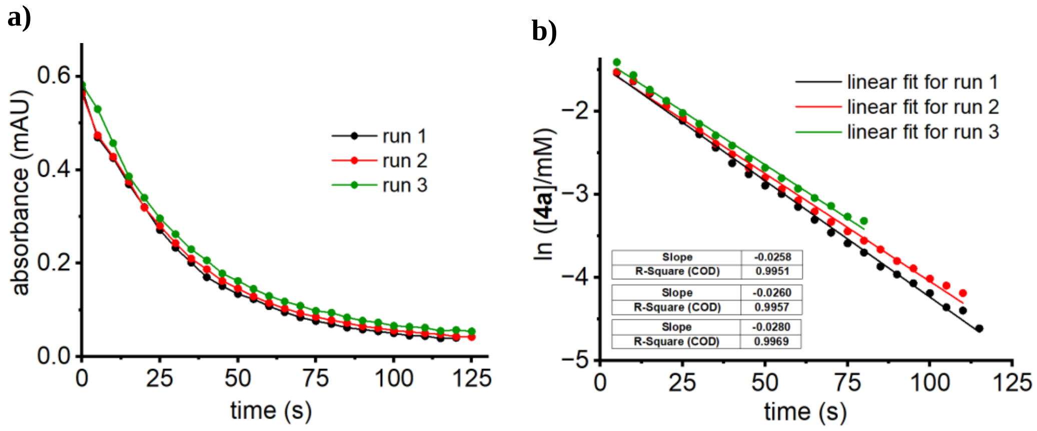

Figure S20 (**A**) Absorbance as a function of time in the reduction of **4a** when using 10 mM (40 eq.) $H_3N{\cdot}BH_3$; (**B**) Linearised plot of [**4a**] as a function of time when using 10 mM (40 eq.) $H_3N{\cdot}BH_3$.

Varying the initial concentration of $H_3N{\cdot}BH_3$ led to different values for the observed pseudo-first order rate constant:

Table S9 Observed pseudo-first order rate constants k′ as a function of initial borane concentration.

**$[AB]_0$ (mM)**	**10**	**25**	**37.5**	**50**
**Observed rate constant k′ (s^{-1})**	0.0258	0.0670	0.0945	0.1104
	0.0260	0.0692	0.0900	0.1081
	0.0280	0.0673	0.0950	0.1119
**Average k′ (s^{-1})**	0.0266	0.0678	0.0932	0.1101
**Standard deviation**	0.0010	0.0010	0.0022	0.0016

Plotting these results as a function of the initial borane concentration gives a straight line whose slope is the second order rate constant $k_{reduction}$ from the following rate equation:

$$r_{red} = -k_{reduction} \times [H_3N \cdot BH_3] \times [\mathbf{4a}]$$

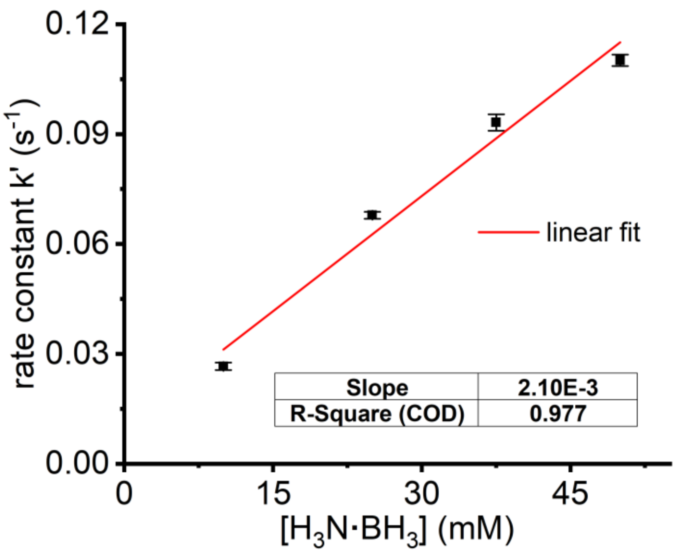

Figure S21 Plot of apparent pseudo-first order rate constant k′ as a function of $H_3N \cdot BH_3$ concentration in the reduction of aldehyde **4a**.

The calculated second-order rate constant is thus: $k_{reduction} = 2.1 \times 10^{-3}$ $mM^{-1}.s^{-1}$.

The rate of reduction is: $r_{red} = 2.1 \times 10^{-3} \cdot [H_3N \cdot BH_3] \cdot [\mathbf{4a}]$ $mM.s^{-1}$.

7.3) Determination of the rate of enantiomerization r_{enant}

Rotational energy barrier of 3a

The rotational energy barrier of **3a** was estimated using the analogous desymmetrized derivative **5a**.

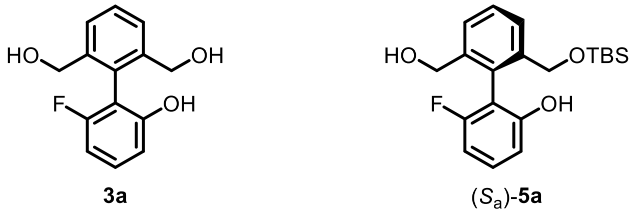

Figure S22 Structures of biaryls **3a** and (S_a)-**5a**.

An enantioenriched solution of (S_a)-2'-(((*tert*-butyldimethylsilyl)oxy)methyl)-6-fluoro-6'-(hydroxymethyl)-[1,1'-biphenyl]-2-ol **5a** (72% ee) in toluene was heated to 100 °C. An aliquot of the solution was taken at intervals, the toluene removed under reduced pressure and the resulting residue taken up in 80:20 hexane:IPA and cooled to 0 °C. The samples were analysed immediately using HPLC with a chiral stationary phase (Chiral Regis Whelk O1, 250 × 4.6 mm, 5 µm, 99:1 hexane:IPA, 1.15 mL min^{-1}).

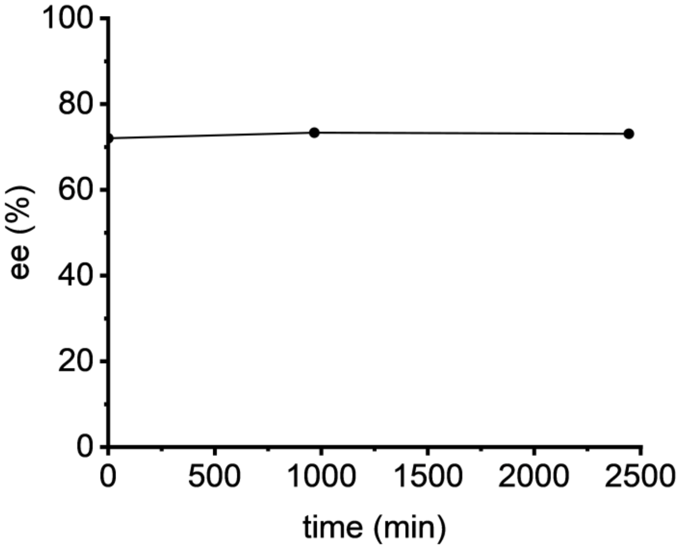

Figure S23 Evolution of enantiomeric excess of enantioenriched **5a** over time upon heating to 100 °C

No racemization was observed after 2445 min at 100 °C. A reduction in ee to, for example, 60% over this time period, would correspond to a rotational energy barrier of 136.4 kJ.mol^{-1}. Therefore, we can conclude that $\Delta G^{\ddagger}_{rot} > 136.4$ kJ.mol^{-1}. The method used to determine this limiting value is explained in the following paragraph.

Racemization is a first-order kinetic process and therefore the ee of an enantioenriched sample will decrease exponentially over time, as described by Equation A below. Rearranging Equation A to give Equation B allows 1/ln(ee) vs. time (s) to be plotted providing a straight line with a gradient = k_{rac}. From k_{rac}, using Equations C–E, the parameters k_{enant}, $t^{1/2}{}_{rac}$ and $\Delta G^{\ddagger}{}_{rac}$ can also be calculated.[10]

Equation A: $ee = ee_{t=0}\, e^{k_{rac}t}$

Equation B: $\left(ln\frac{1}{ee}\right) = k_{rac}t + ln\left(\frac{1}{ee_{t=0}}\right)$

Equation C: $k_{rac} = 2k_{enant}$

Equation D: $t^{1/2}{}_{rac} = \frac{ln2}{k_{rac}}$

Equation E: $\Delta G^{\ddagger} = RTln(\frac{k_B T}{k_{enant}h})$

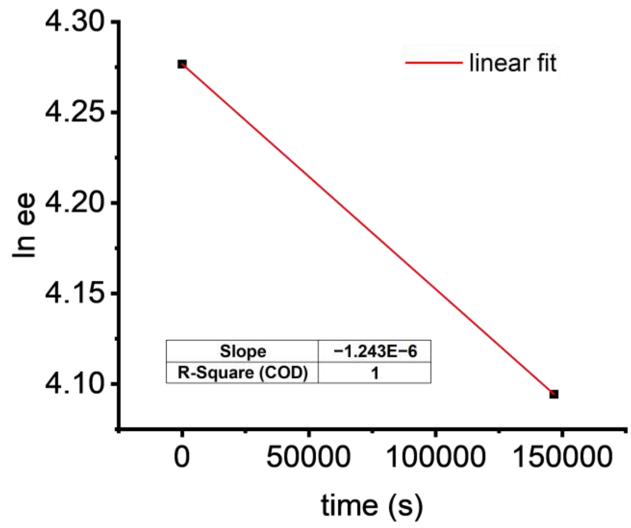

Figure S24 Linearised plot of enantiomeric excess of **5a** as a function of time assuming a drop to 60% ee upon heating in toluene to 100 °C.

$k_{rac} = 1.243 \times 10^{-6}\ s^{-1}$

From this value of k_{rac}, a limiting value of $\Delta G^{\ddagger}{}_{rot}$ = 136.4 kJ.mol^{-1} is determined.

Rate of enantiomerization of 4a

Extensive attempts were made to determine the rotational energy barrier of **4a** using HPLC methods, but separation of the enantiomers could not be achieved using a range of chiral stationary phases and at low temperatures. Furthermore, EXSY NMR analysis of **4a** was precluded by the lack of resolution of the diastereotopic benzylic protons. In view of these challenges, the rotational energy barrier of **4a** was

estimated using ester **6a** (Figure S25), whose axial diastereoisomers show resolved signals by ^{1}H and ^{19}F NMR spectroscopy.

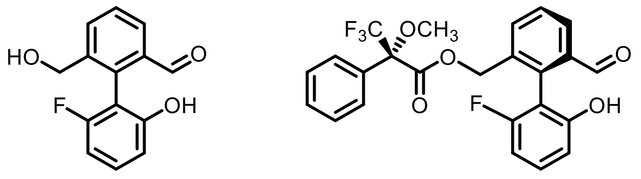

4a (*S*,*S*a)-**6a**

Figure S25 Structures of biaryls **4a** and (*S*,S_a)-**6a** (S_a axial conformer drawn arbitrarily).

^{19}F EXSY NMR spectroscopy revealed exchange of the diastereoisomeric Ar–F signals arising from rotation about the biaryl axis of **6a** on the relaxation timescale of the NMR. The rate of exchange of the Ar–F signals, and hence the energy barrier to rotation, was quantified using the procedure detailed below, which was adapted from the literature.[11]

A VT ^{19}F EXSY at 500 ms mixing time was taken using a sample of (2'-fluoro-6-formyl-6'-hydroxy-[1,1'-biphenyl]-2-yl)methyl (2*S*)-3,3,3-trifluoro-2-methoxy-2-phenylpropanoate **6a** in 2:1 DMSO-d_6:D_2O as well as a reference spectra at 5 ms, 30 °C.

Standard Bruker pulse sequences with the following parameters were used:
^{19}F, ^{19}F-EXSY: Pulse program: noesygpphpp; Relaxation delay = 2.0 s, Acquisition time = 0.36 s, SW = 5 ppm (F2), 5 ppm (F1), TD = 2048 (F2), 150 (F1), NS = 16, mixing time D8 = 5–500 ms.

The absolute integrals of the diagonal spots at −113.05 and −113.18 ppm and their corresponding exchange spots were acquired using MestreNova. Mestrelab EXSYCalc software was used to calculate forward and backward exchange rate constants (k_1 and k_{-1}).

$$\text{Equation F: } k = \frac{k_B T}{h} e^{-\frac{\Delta H^{\ddagger}}{RT}} e^{\frac{\Delta S^{\ddagger}}{R}}$$

$$\text{Equation G: } ln\frac{k}{T} = \frac{-\Delta H^{\ddagger}}{R}\frac{1}{T} + ln\frac{k_B}{h} + \frac{\Delta S^{\ddagger}}{R}$$

By plotting 1/T vs. ln(k/T), using both the forward and backward exchange rate constants, $\Delta H^{\ddagger}$ and $\Delta S^{\ddagger}$ can be calculated using Equations H and I:

$$\text{Equation H: } Gradient = -\frac{\Delta H^{\ddagger}}{R}$$

$$\text{Equation I: } y\ intercept = ln\frac{k_B}{h} + \frac{\Delta S^{\ddagger}}{R}$$

$\Delta G^{\ddagger}$ can then be calculated at any temperature (T) using Equation J:

$$\text{Equation J: } \Delta G^{\ddagger} = \Delta H^{\ddagger} - T\Delta S^{\ddagger}$$

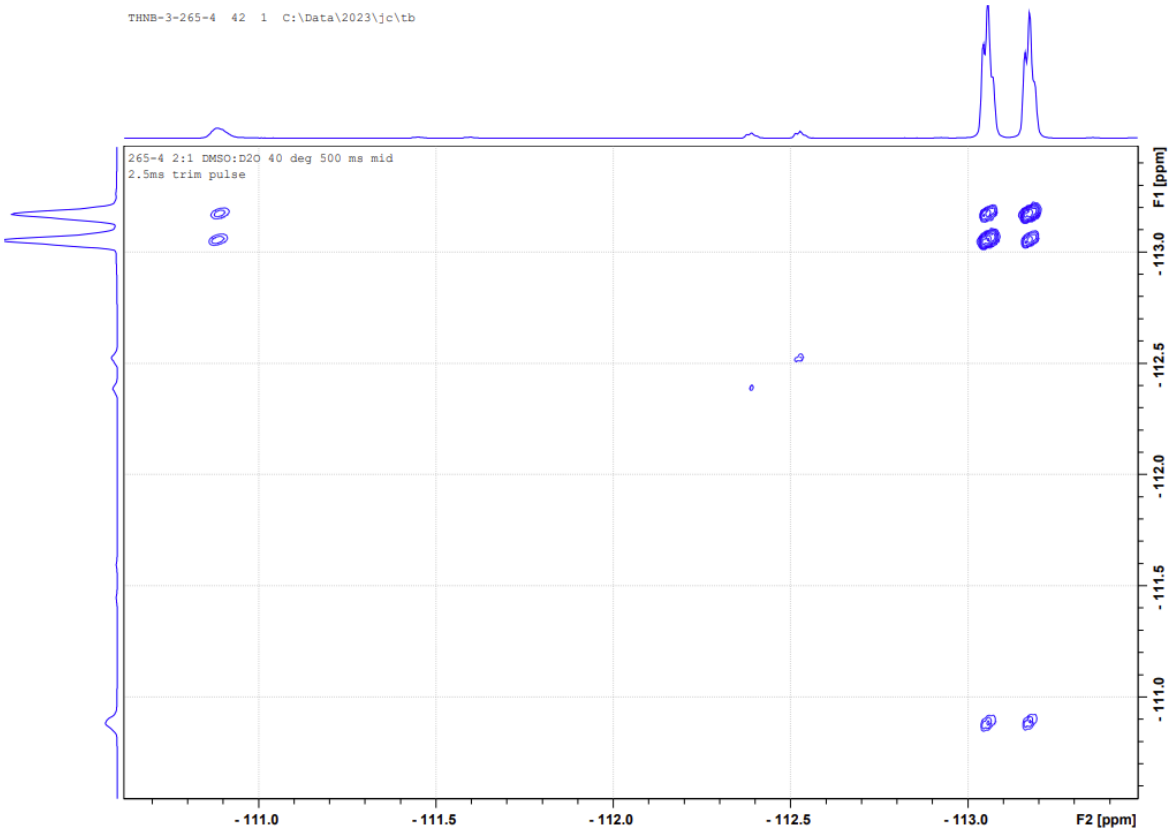

Figure S26 ^{19}F EXSY of (2'-fluoro-6-formyl-6'-hydroxy-[1,1'-biphenyl]-2-yl)methyl (2*S*)-3,3,3-trifluoro-2-methoxy-2-phenylpropanoate **6a** in 2:1 DMSO:D_2O, 500 ms mixing time, 40 °C.

Exchange spots between the Ar–F signals of the two diastereoisomers are observed and show that chemical exchange is taking place on the mixing timescale of the EXSY experiment (Figure S26 & S27). These exchange spots are not observed in the reference spectra at 5 ms mixing time (Figure S28). At 500 ms mixing time, exchange spots are also observed between the Ar–F signals and the minor signal at −111.7 ppm, which has been tentatively assigned to a minor hemiacetal isomer of **6a** (Figure S26) (see Supplementary Information Section 2.5).

EXSY data acquired at 30–40 °C at 500 ms mixing time of a freshly made sample of **6a** in 2:1 DMSO-d_6:D_2O are tabulated in Table S10 and are used to calculate the average rotational energy barrier for **6a**, as detailed below.

(It should be noted that samples of **6a** left to stand in 2:1 DMSO-d_6:D_2O over extended periods of time appear to show altered exchange behaviour corresponding to higher rotational barriers.)

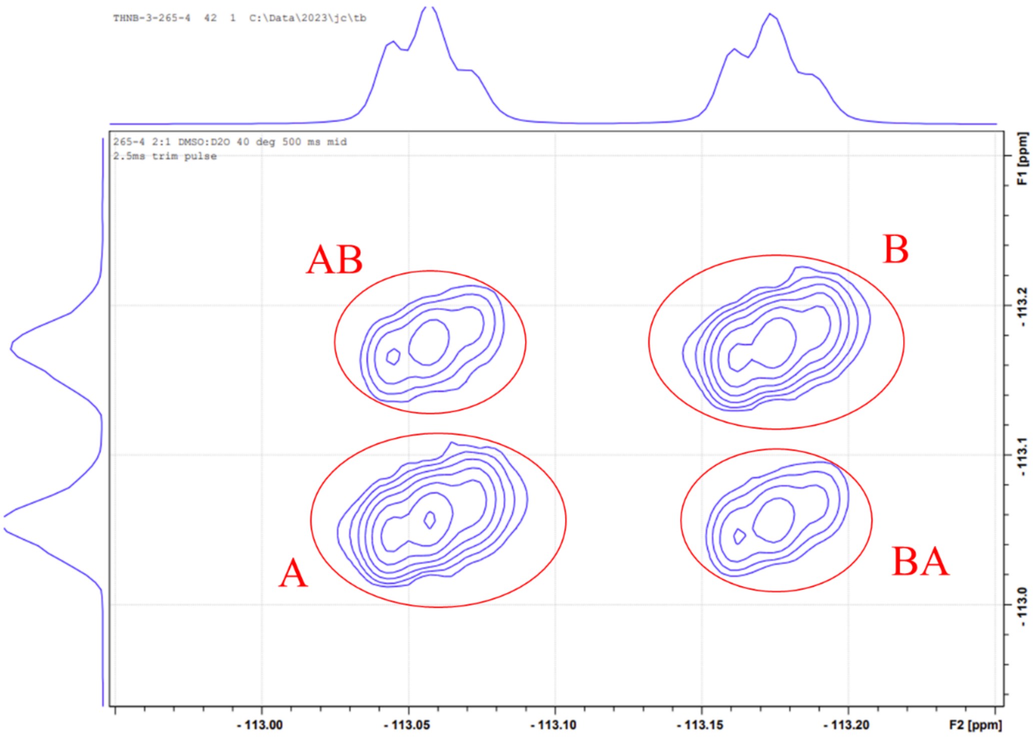

Figure S27 ^{19}F EXSY of (2’-fluoro-6-formyl-6’-hydroxy-[1,1’-biphenyl]-2-yl)methyl (2S)-3,3,3-trifluoro-2-methoxy-2-phenylpropanoate in 2:1 DMSO:D_2O, 500 ms, 40 °C. Spots have been labelled according to MestreLab EXSYCalc guidelines.

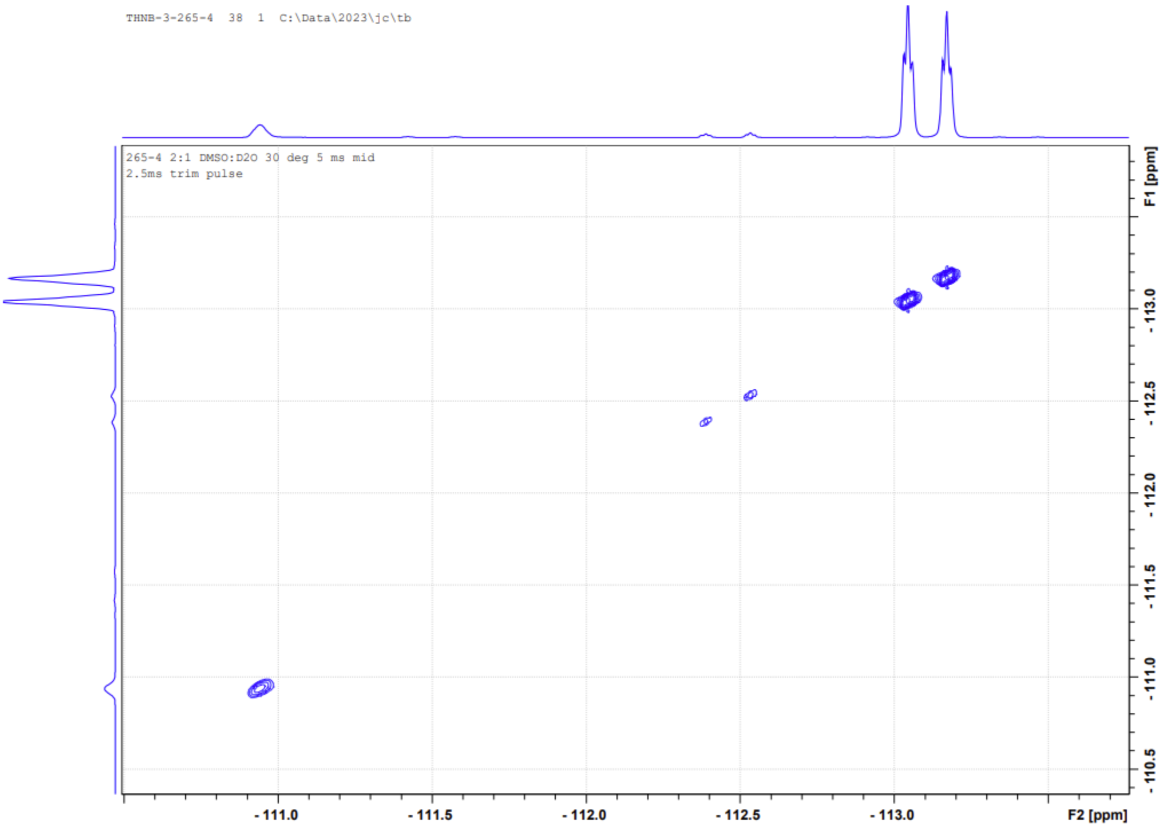

Figure S28 Reference ^{19}F EXSY of (2’-fluoro-6-formyl-6’-hydroxy-[1,1’-biphenyl]-2-yl)methyl (2S)-3,3,3-trifluoro-2-methoxy-2-phenylpropanoate in 2:1 DMSO:D_2O, 5 ms, 30 °C.

Table S10 Kinetic parameters for the rotation of compound (*S*)-**6a**.

Temp (°C)	A integral	B integral	AB integral	BA integral	k_1 (s^{-1})	k_{-1} (s^{-1})	T (K)	1/T (K^{-1})	$\ln k_1/T$	$\ln k_{-1}/T$
30	3093	2772	388	395	0.258	0.280	303.15	0.003298697	−7.0690	−6.9872
32.5	3160	2906	458	427	0.270	0.319	305.65	0.003271716	−7.0317	−6.8650
35	3087	2921	483	485	0.310	0.341	308.15	0.003245173	−6.9017	−6.8065
37.5	3062	2904	584	558	0.361	0.416	310.65	0.003219057	−6.7575	−6.6157
40	3008	2764	580	637	0.427	0.429	313.15	0.003193358	−6.5976	−6.5930

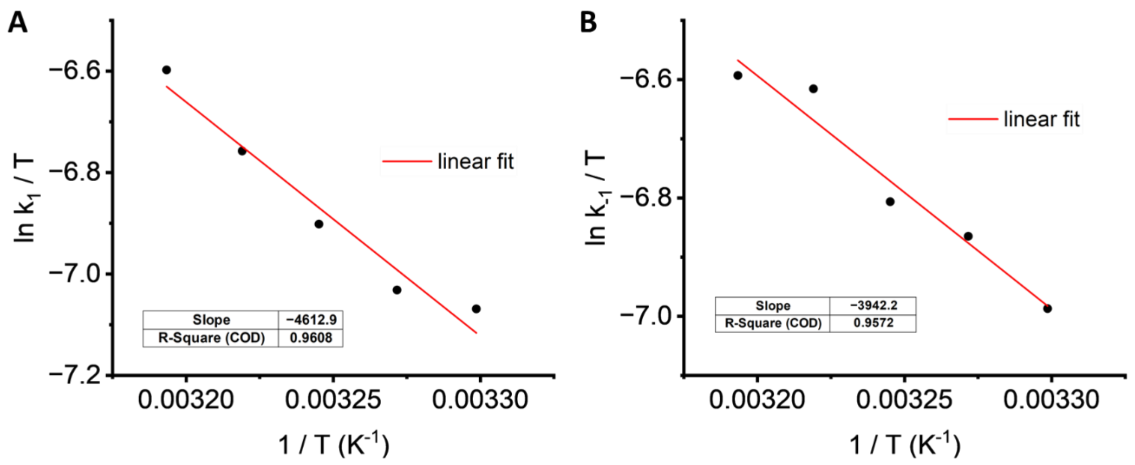

Figure S29 Linearised plots of k/T as a function of 1/T for the (**A**) forward and (**B**) backward interconversion of (*S*)-**6a**.

Forward reaction

$\Delta H^{\ddagger}$ = 38354 $J.mol^{-1}$

$\Delta S^{\ddagger}$ = −130.2 $J.mol^{-1}.K^{-1}$

$\Delta G^{\ddagger}$ = 77.18 $kJ.mol^{-1}$ at 25 °C

$\Delta G^{\ddagger}$ = 79.11 $kJ.mol^{-1}$ at 40 °C

Backward reaction

$\Delta H^{\ddagger}$ = 32777 $J.mol^{-1}$

$\Delta S^{\ddagger}$ = −147.5 $J.mol^{-1}.K^{-1}$

$\Delta G^{\ddagger}$ = 76.75 $kJ.mol^{-1}$ at 25 °C

$\Delta G^{\ddagger}$ = 78.94 $kJ.mol^{-1}$ at 40 °C

Average ΔG‡ = 79.03 kJ.mol^{-1} at 40 °C

We estimate the rate of enantiomerization of **4a** at 40 °C, the operating temperature of the motor, r_{enant}, from ΔG‡ = 79.03 kJ.mol^{-1} calculated above for **6a**.

The enantiomerization (interconversion) of aldehyde **4a** is a first-order process,[10] and the rate of enantiomerization, r_{enant}, thus depends on the concentration of **4a**, as per the following rate law:

$$r_{\text{enant}} = k_{\text{enant}} \cdot [\mathbf{4a}]$$

k_{enant} is then calculated from ΔG‡ by rearranging Equation E to give Equation K.

$$\text{Equation K: } k_{enant} = \left(\frac{k_B T}{e^{\frac{\Delta G^{\ddagger}}{RT}}}\right)/h$$

Therefore, at the operating temperature of the motor, $r_{\text{enant}} = 4.2 \times 10^{-1} \cdot [\mathbf{4a}]$ mM.s^{-1}

8) Autonomous operation

8.1) Reverse phase calibration (operation of 3a)

Waters Spherisorb S5 ODS2, 5 µm, 4.6 × 250 mm, eluent composition and gradient detailed in Table S11, λ = 270 nm, t_R = **S3k**, 6.3 min; **3a**, 7.1 min; **S3j**, 8.7 min; **4a**, 11.0 min; **S3e**, 14.8 min; 2-naphthol, 15.3 min.

Due to difficulties in purifying **S3k**, the concentration of the resulting solution was determined by the addition of 2-naphthol (2.69 mg, 18.7 µmol) to a 700 µL aliquot of the reaction mixture derived from the hydrolysis of **S3d** detailed above for the preparation of **S3k**. The ratio of 2-naphthol to **S3k** was determined by ^{1}H NMR spectroscopy (500 MHz, 8 scans, D1 = 60 s), using the relative integrals of the peaks of **S3k** and 2-naphthol at 6.25 ppm and 6.69 ppm, respectively. This solution was used for the formulation of HPLC samples for the calibration of **S3k**. The concentrations of **S3k** were determined from the peak area of 2-naphthol, which was quantified from its calibration curve (Figure S30, D), in combination with the ratio of 2-naphthol to **S3k** determined by ^{1}H NMR spectroscopy, allowing the calibration of **S3k** (Figure S30, F).

Table S11 Gradient conditions for calibration curves for **S3k, 3a, S3j, 4a, S3e,** and 2-naphthol on Waters Spherisorb S5 ODS2, 1 mL/min, 22 min run.

Time (min)	MeCN:H_2O (0.5% Formic Acid)
0	20:80
10	40:60
15	60:40
16	95:5
18	95:5
19	20:80

Quantification of **S3e** was performed at both 20 µL injection volume, the standard injection volume for HPLC analysis of the motor, and a higher (100 µL) injection volume. This allowed for determination of the LOD for **S3e** to a lower concentration, since a prominent concentration of **S3e** would be deleterious to the selectivity of the rotations of motor **3a**.

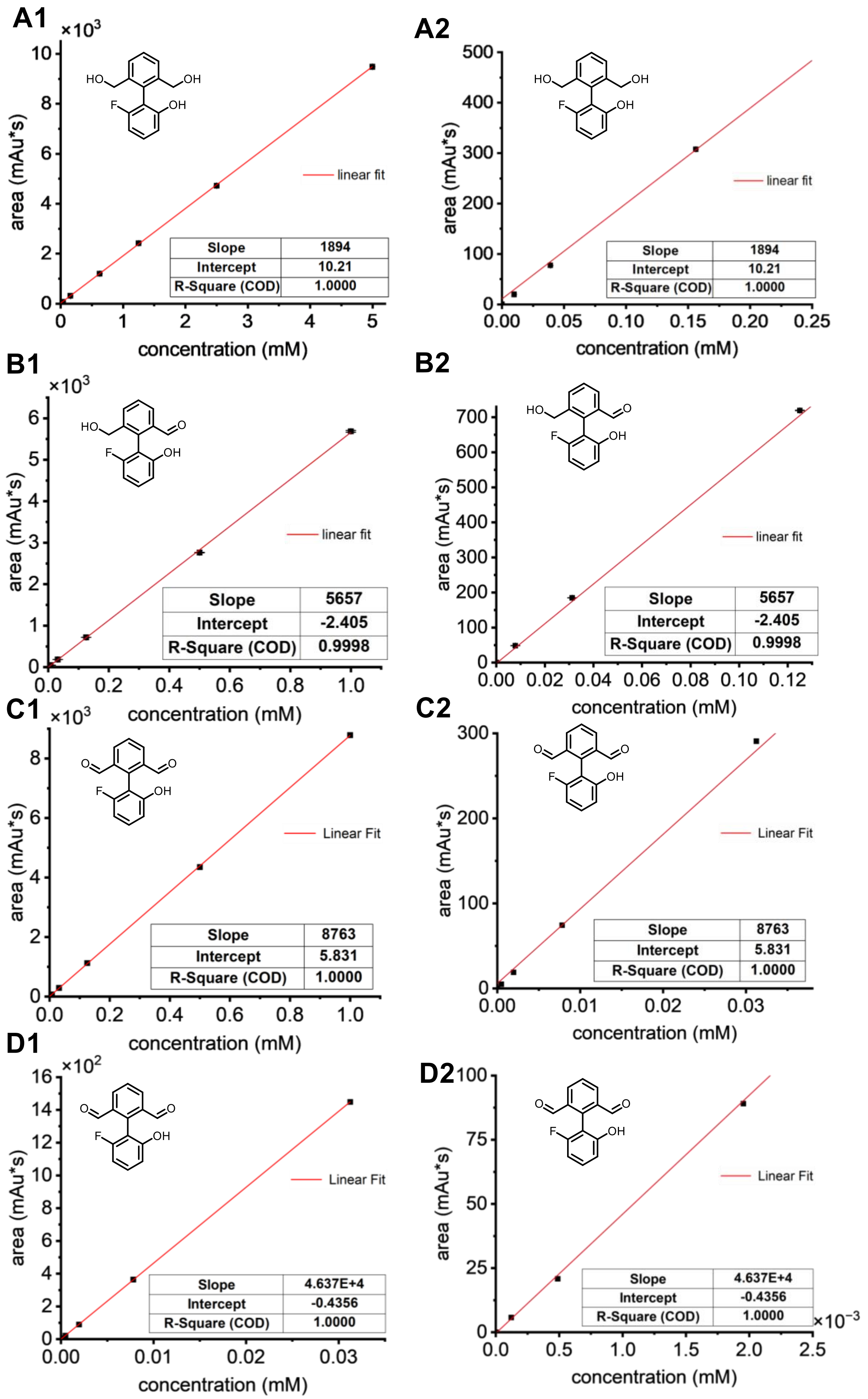
A1
A2
B1
B2
C1
C2
D1
D2
area (mAu*s)
concentration (mM)
linear fit
Linear Fit
HO
OH
F
O
Slope 1894
Intercept 10.21
R-Square (COD) 1.0000
Slope 5657
Intercept -2.405
R-Square (COD) 0.9998
Slope 8763
Intercept 5.831
R-Square (COD) 1.0000
Slope 4.637E+4
Intercept -0.4356
R-Square (COD) 1.0000
×10^3
×10^2
×10^-3

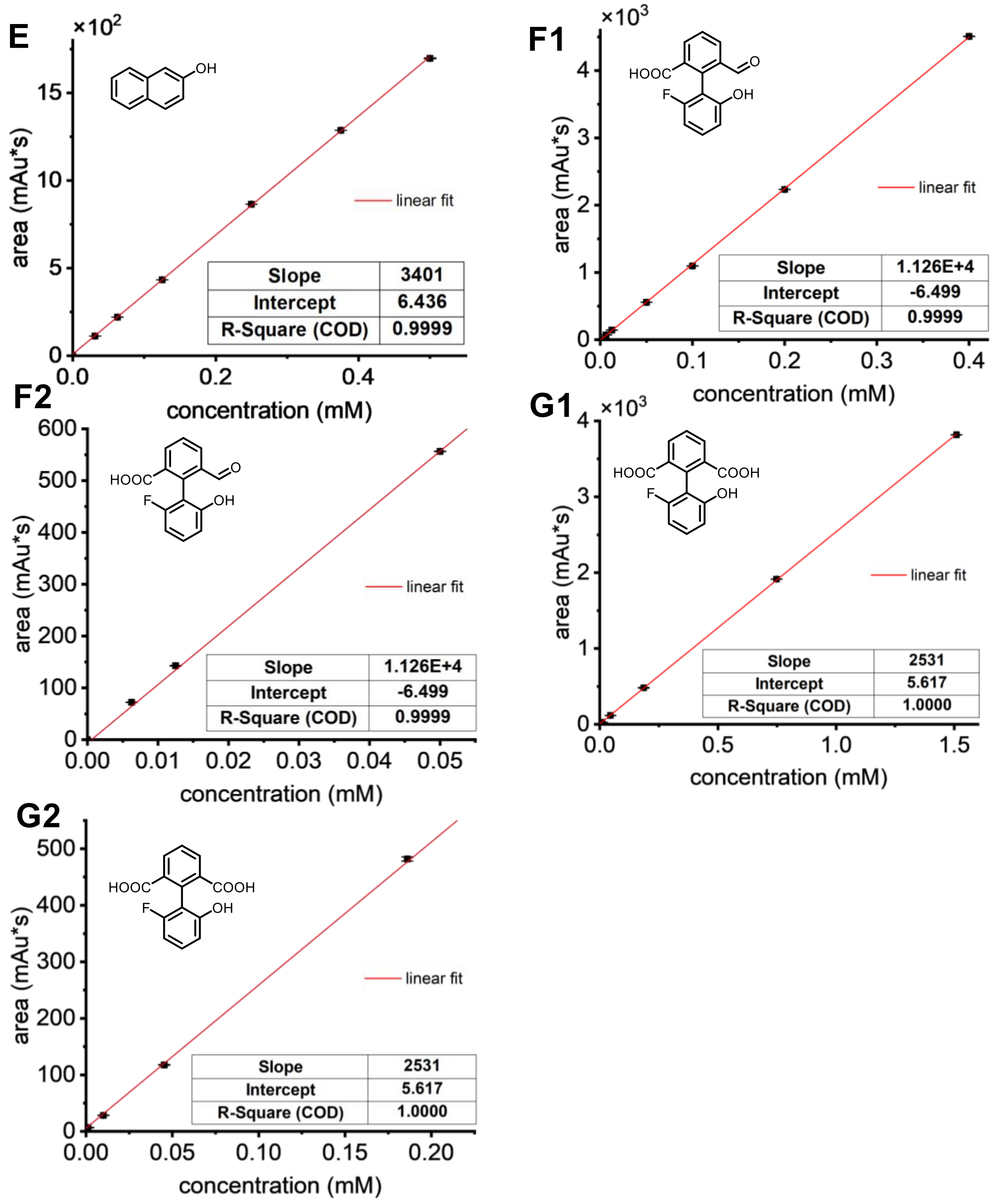

Figure S30 Calibration curves for compounds (**A1** and **A2**): **3a**; (**B1** and **B2**): **4a**; (**C1** and **C2**): **S3e**, 20 µL injection volume; (**D1** and **D2**): **S3e**, 100 µL injection volume; (**E**): 2-naphthol (internal standard); (**F1** and **F2**): **S3j**; (**G1** and **G2**): **S3k** on Waters Spherisorb S5 ODS2, 5 µm, 4.6 × 250 mm (reverse phase).

8.2) Operation of 3a under the redox cyclic reaction network

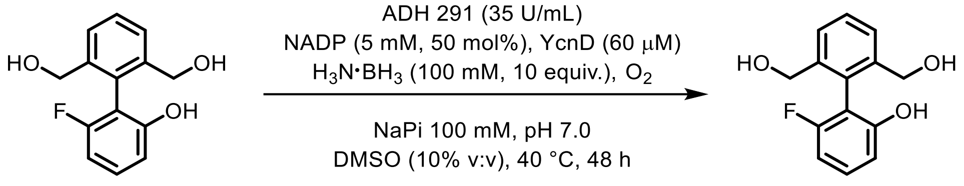

Scheme S10 Operation of **3a** under the optimised conditions of the redox cyclic reaction network.

In a 2 mL Eppendorf vial were added NaPi 100 mM pH 7.0, ADH 291 (35 U/mL), YcnD (60 µM), NADP (5 mM), and $H_3N{\cdot}BH_3$ (100 mM). To this solution was added substrate **3a** (10 mM final concentration) in DMSO (25 µL). The final volume was 250 µL. The vial was sealed, and the colourless suspension was shaken at 40 °C for 48 hours. After this time, diluent (1000 µL, composition of dilutant solution: MeCN:H_2O:AcOH:NaOAc (8.00:1.94:0.0600, NaOAc (1.40 mg/mL), with a known concentration of 2-naphthol as an internal standard) was added to each reaction vial, and each reaction mixture was centrifuged for 5 minutes (14800 rpm). The resulting supernatant was subjected to reverse phase HPLC analysis (Waters Spherisorb S5 ODS2, 5 µm, 4.6 × 250 mm, eluent composition and gradient detailed in Table S11, λ = 270 nm, t_R = **S3k**, 6.3 min; **3a**, 7.1 min; **S3j**, 8.7 min; **4a**, 11.0 min; **S3e**, 14.8 min; 2-naphthol, 15.3 min). Reverse phase HPLC analysis confirmed that the reaction mixture is composed of **3a** and there is no evidence of formation of oxidation products **4a**, **S3e**, **S3g**, **S3j** or **S3k**. For reference, the reverse phase HPLC chromatograms of the reaction mixtures after 48 hours are provided in Figure S31.

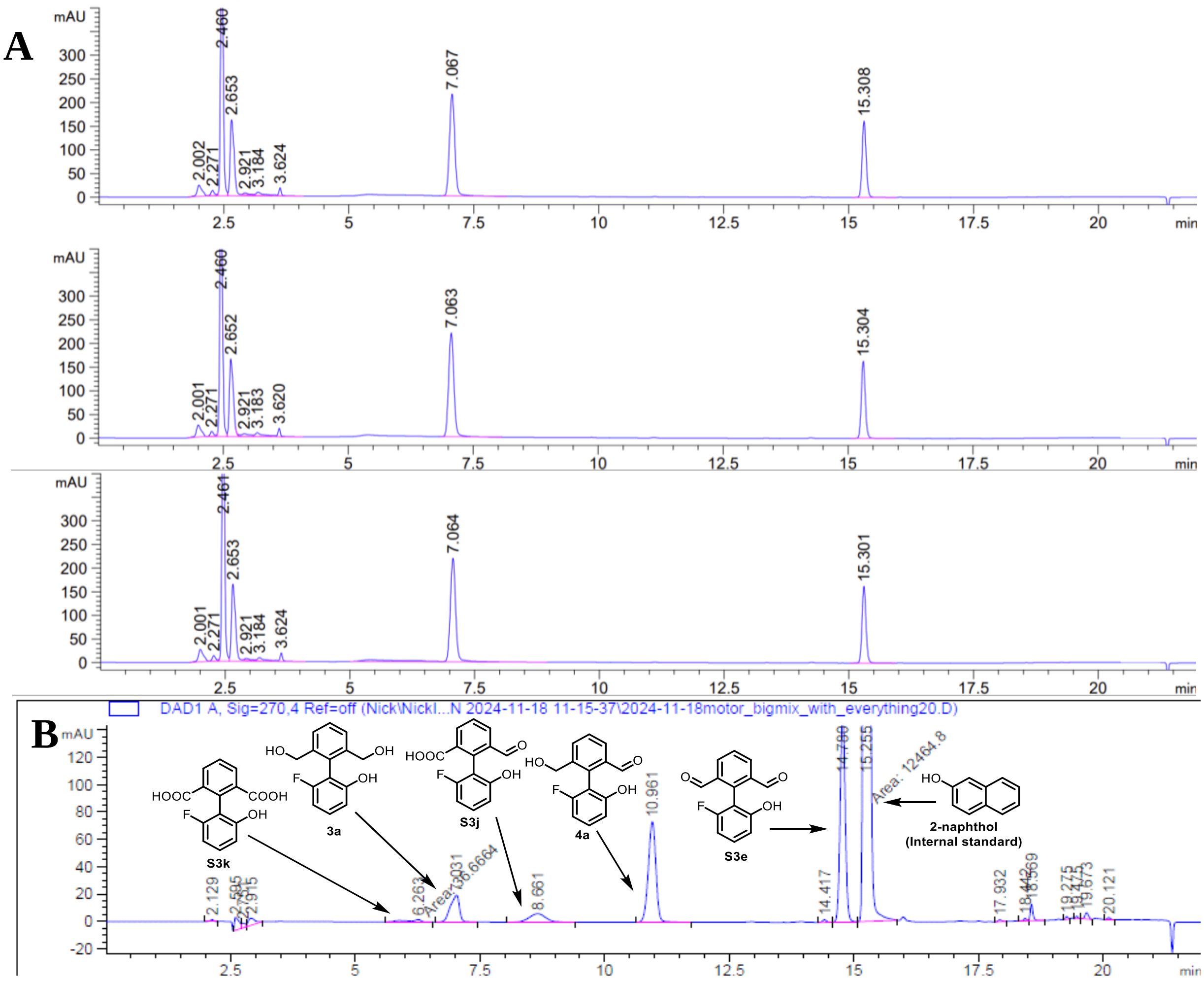

Figure S31 Reverse phase HPLC chromatograms of: (**A**) reaction mixtures of triplicate runs of the operation of **3a** under the redox cyclic reaction network after 48 h, (**B**) independently prepared chromatogram containing **S3k**, **3a**, **S3j**, **4a**, **S3e** and 2-naphthol (internal standard) to showing retention times.

The yield of **3a** in HPLC samples was determined by converting the observed absorbance values to concentration using a previously prepared calibration curve (see Supplementary Information Section 8.1) and comparing the ratio of **3a** : 2-naphthol in each reaction. The yield data from triplicate experiments is provided in Table S12 below.

Table S12 Yield data from operation of **3a** under the redox cyclic reaction network after 48 h. [**3a**] was calculated from the integral of the corresponding peak (t_R = 7.1 min) in HPLC traces using a previously prepared calibration curve (see Supplementary Information Section 8.1); analysis using reverse phase HPLC (Waters Spherisorb S5 ODS2, 5 µm, 4.6 × 250 mm). The error given is the sample standard deviation between the triplicates.

Run	Yield **3a** (%)
1	99.8
2	100.5
3	99.6
**Average**	99.9
**Standard deviation**	0.47

8.3) Evolution of deuterium content over time

In the following experiment, $H_3N{\cdot}BH_3$ was replaced by $H_3N{\cdot}BD_3$. We postulated that this substitution would allow us to confirm that **3a** was a substrate for the cyclic redox reaction network, as incorporation of deuterium at the benzylic positions of **3a** can arise only if **3a** was subject to cycles of oxidation and reduction.

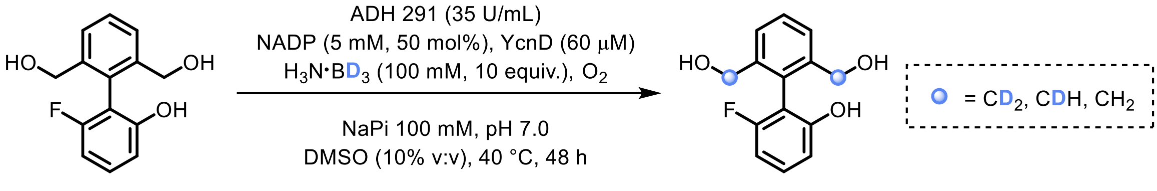

3a, 10 mM D$_2$-**3a**

Scheme S11 Operation of **3a** under the cyclic redox reaction network with $H_3N{\cdot}BD_3$ in place of $H_3N{\cdot}BH_3$.

In a 2 mL Eppendorf vial were added NaPi 100 mM pH 7.0, ADH 291 (35 U/mL), YcnD (60 µM), NADP (5 mM), and $H_3N{\cdot}BD_3$ (100 mM). To this solution was added substrate **3a** (10 mM final concentration) in DMSO (50 µL). The final volume was 500 µL. The vial was sealed, and the colourless suspension was shaken at 40 °C. Aliquots (70 µL) were taken at different time points. The aliquots were extracted twice with 300 µL MTBE. The organic layers were dried over Na_2SO_4, filtered, and the volatiles were removed under reduced pressure. Each aliquot was then analysed by ^{1}H NMR (600 MHz, 32 scans, D1 = 30 s) to determine the deuterium incorporation at the benzylic position (Figures S32 and S33).

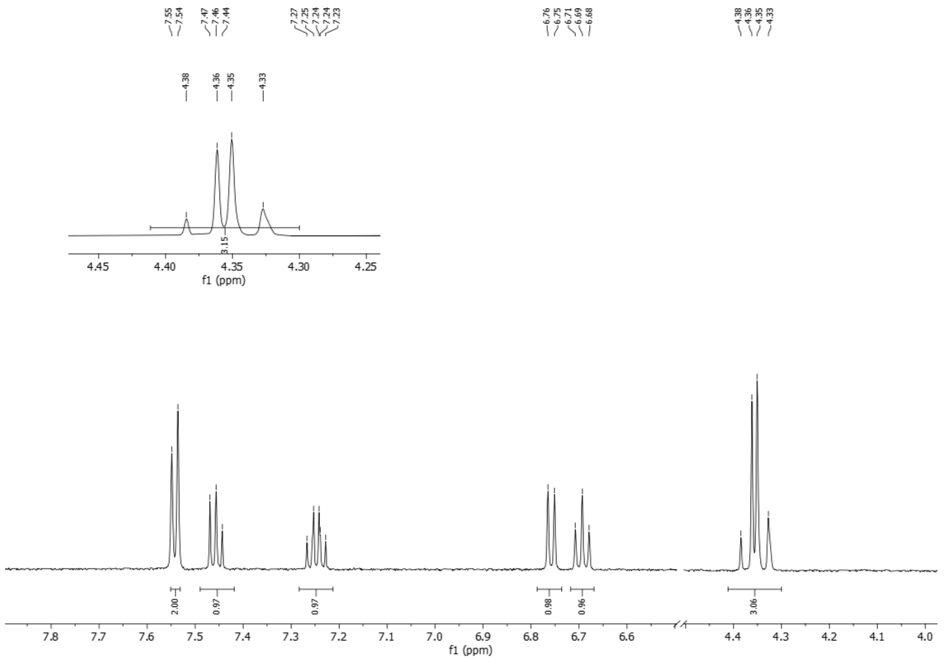

Figure S32 ^{1}H NMR of the reaction mixture after 48 h showing a decrease in the integration of the benzylic methylene signals, indicating deuterium incorporation.

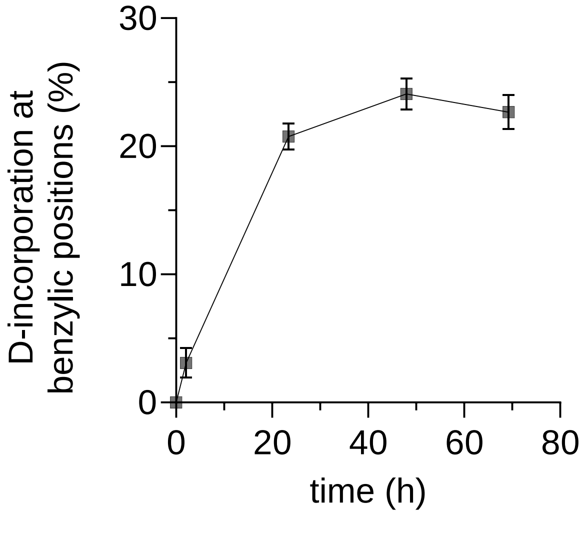

Figure S33 Deuterium incorporation at benzylic positions of **3a** when **3a** is subjected to the redox cyclic reaction network with $H_3N \cdot BD_3$ in place of $H_3N \cdot BH_3$.

The same experiment was repeated with additional pulses of the following reagents added at 43 h and the deuterium incorporation at the benzylic position was determined by ^{1}H NMR (Figures S34).

- pulse of ADH 291 (~35 U/mL, added as a solid)
- pulse of NADP (~5 mM, added as a solid)
- pulse of YcnD (~60 µM)

- pulse of all the above

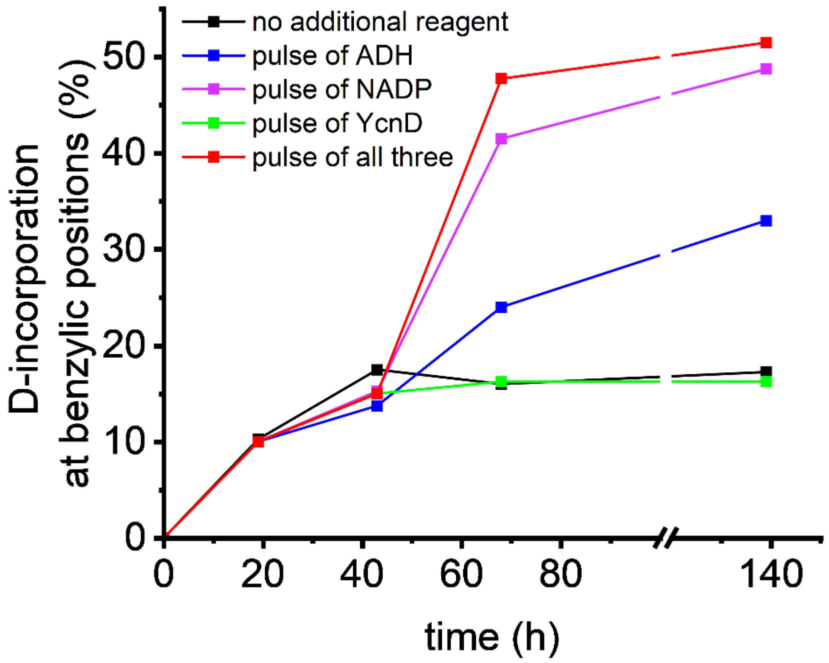

Figure S34 Deuterium incorporation at benzylic positions of **3a** when **3a** is subjected to the redox cyclic reaction network with $H_3N{\cdot}BD_3$ in place of $H_3N{\cdot}BH_3$. A pulse of the indicated reagent(s) was added after 43 h.

These results confirm that both ADH 291 and YcnD are still operational at 43 h and that sufficient $H_3N{\cdot}BD_3$ is still present at 43 h for the cyclic redox reaction network to remain operational.

8.4) Pulsed $H_3N{\cdot}BH_3$ experiment

In the following experiment, sub-stoichiometric pulses of $H_3N{\cdot}BH_3$ fuel were added at 24 h intervals to investigate the viability of the oxidation system over extended periods of time (at least 96 h).

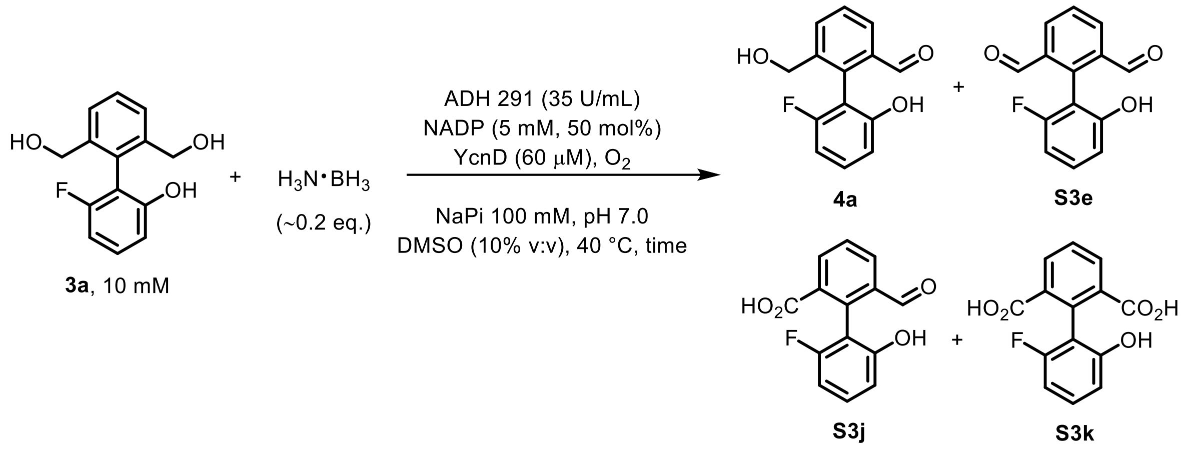

Scheme S12 Operation of **3a** under the cyclic redox reaction network but with $H_3N{\cdot}BH_3$ introduced in sub-stoichiometric pulses at 24 h intervals.

In a 2 mL Eppendorf vial were added NaPi 100 mM pH 7.0, ADH 291 (35 U/mL), YcnD (60 µM), and NADP (5 mM). To this solution was added substrate **3a** (10 mM final concentration) in DMSO (50 µL). The final volume was 500 µL. The vial was sealed, and the pale yellow solution was shaken at 40 °C. Pulses of $H_3N{\cdot}BH_3$ (10 µL of a 0.1 mM solution, ~0.2 eq. each time) were added after 24, 48, 72 and 95 h total reaction time. Aliquots (20 µL) were taken at different time points and were immediately quenched with 200 µL of diluent (composition detailed in Supplementary Information Section 5.1). Samples were centrifuged for 5 minutes (14800 rpm), and the resulting supernatant was subjected to reverse phase HPLC analysis (Kromasil 100-5-C18, 5 µm, 4.6 × 250 mm, eluent composition and gradient detailed in Table S4, $\lambda = 270$ nm, t_R = **S3k**, 10.2 min; **3a**, 10.7 min; **S3j**, 11.9 min; **4a**, 13.2 min; **S3e**, 15.6 min; 2-naphthol, 16.2 min).

Yields of triol **3a** and the oxidation products aldehyde **4a**, dialdehyde **S3e**, carboxylic acid **S3j**, and dicarboxylic acid **S3k** over time were determined from the relative integrals of their peaks in the HPLC traces and previously prepared calibration curves (see Supplementary Information Section 4.3). Yield data is provided in Table S13 and Figure S35 below and corresponds to averages derived from experiments conducted in triplicate. The total concentration of reaction components was assumed to remain 10 mM as no further oxidation products were observed by reverse phase HPLC.

For reference, Figure S36 provides a chromatogram of **3a**, potential oxidation products (**4a**, **S3e**, **S3g**, **S3j**, **S3k**), and 2-napththol recorded using the reverse phase HPLC protocol described above for analysis of the pulsed fuelling of **3a** (Kromasil 100-5-C18, 5 µm, 4.6 × 250 mm, eluent composition and gradient detailed in Table S4, $\lambda = 270$ nm). The assignment of the peaks in the chromatogram was

achieved by independent injections of each component onto the HPLC. Formulation of the combined sample: **3a** (< 1 mg), **4a** (< 1 mg), **S3e** (< 1 mg), **S3g**, (< 1 mg), **S3j** (< 1 mg), **S3k** (< 1 mg) were added to a vial as pure compounds, and dissolved in DMSO (25 µL), NaPi 100 mM pH 7.0 (225 µL), and dilutant (1 mL).

Table S13 Yield of **3a** to oxidation products **4a**, **S3e**, **S3j**, and **S3k** under the cyclic redox reaction network with $H_3N{\cdot}BH_3$ introduced in sub-stoichiometric pulses at 24 h intervals; analysis using reverse phase (Kromasil 100-5-C18); the total concentration of reaction components was assumed to remain 10 mM as no further oxidation products (for example, **S3g**) were observed; <LOD below the limit of detection; [a] total yield of oxidation products is the sum of yields of **4a**, **S3e**, **S3j** and **S3k**; yellow highlight indicates timepoints taken immediately before the addition of $H_3N{\cdot}BH_3$. For the yields of **3a**, **4a**, **S3e**, **S3j**, and **S3k**, the error given is the sample standard deviation between the triplicates; for the total yield of oxidation products, the error given is the population standard deviation between the triplicates.

Entry	Time (h)	Yield **3a** (%)	Yield **4a** (%)	Yield **S3e** (%)	Yield **S3j** (%)	Yield **S3k** (%)	Total yield oxidation products (%)[a]
1	0	98.7 ± 0.3	1.3 ± 0.3	<LOD	<LOD	<LOD	1.3 ± 0.2
2	6	82.3 ± 5.5	16.1 ± 4.5	0.9 ± 0.8	0.7 ± 0.2	<LOD	17.7 ± 3.8
3	24	55.2 ± 1.4	34.8 ± 1.3	3.7 ± 0.5	5.3 ± 0.2	0.9 ± 1.6	44.8 ± 1.7
4	25	83.9 ± 5.5	10.6 ± 4.5	0.7 ± 0.8	4.8 ± 0.2	<LOD	16.1 ± 0.7
5	30	67.9 ± 0.5	23.3 ± 0.3	2.2 ± 0.1	5.5 ± 0.1	1.2 ± 0.2	32.1 ± 0.3
6	48	49.3 ± 1.5	33.7 ± 1.2	4.0 ± 0.8	10.9 ± 0.4	2.1 ± 0.3	50.7 ± 1.7
7	49	81.1 ± 1.1	7.7 ± 0.4	0.4 ± 0.6	8.3 ± 0.1	2.5 ± 0.8	18.9 ± 0.9
8	54	65.7 ± 0.9	20.6 ± 0.3	1.9 ± 0.1	9.2 ± 0.6	2.5 ± 0.8	34.3 ± 0.8
9	72	49.6 ± 0.9	29.7 ± 0.3	3.9 ± 0.1	13.6 ± 0.6	3.1 ± 0.8	50.4 ± 1.3
10	73	82.1 ± 0.5	5.3 ± 0.3	<LOD	8.8 ± 0.4	3.7 ± 0.3	17.9 ± 0.5
11	77	69.4 ± 0.8	16.9 ± 0.2	1.6 ± 0.1	9.4 ± 0.7	2.8 ± 0.2	30.6 ± 0.6
12	95	50.8 ± 1.4	28.6 ± 0.9	3.7 ± 0.3	13.5 ± 0.4	3.3 ± 0.2	49.2 ± 0.9
13	96	86.1 ± 1.9	1.9 ± 1.1	2.2 ± 1.9	6.1 ± 0.9	3.7 ± 0.2	13.9 ± 2.0
14	167	52.1 ± 0.9	22.2 ± 0.1	2.7 ± 0.1	16.9 ± 0.9	6.2 ± 0.2	47.9 ± 0.8

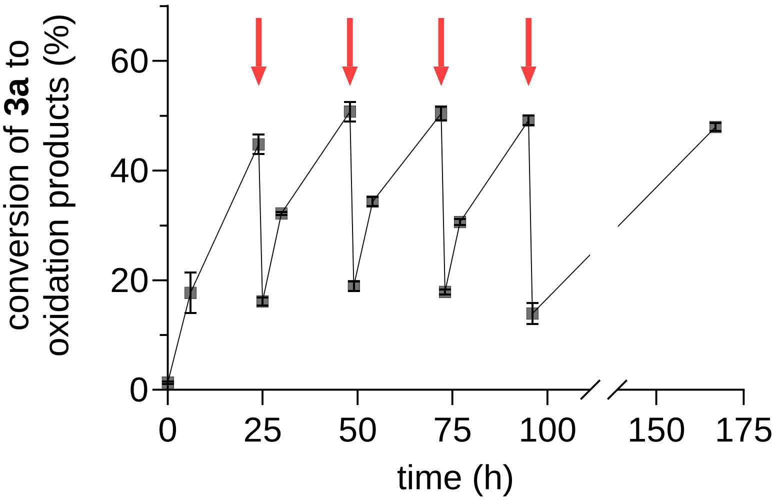

Figure S35 Conversion of **3a** to all observed oxidation products (**4a**, **S3e**, **S3j**, and **S3k**) over time during biocatalytic oxidation of **3a** with the addition of intermittent pulses of $H_3N{\cdot}BH_3$; conversion of **3a** to oxidation products **4a**, **S3e**, **S3j**, and **S3k** was determined by the relative integrals of their respective peaks in reverse phase HPLC traces where no further oxidation products (for example, **S3g**) were observed.

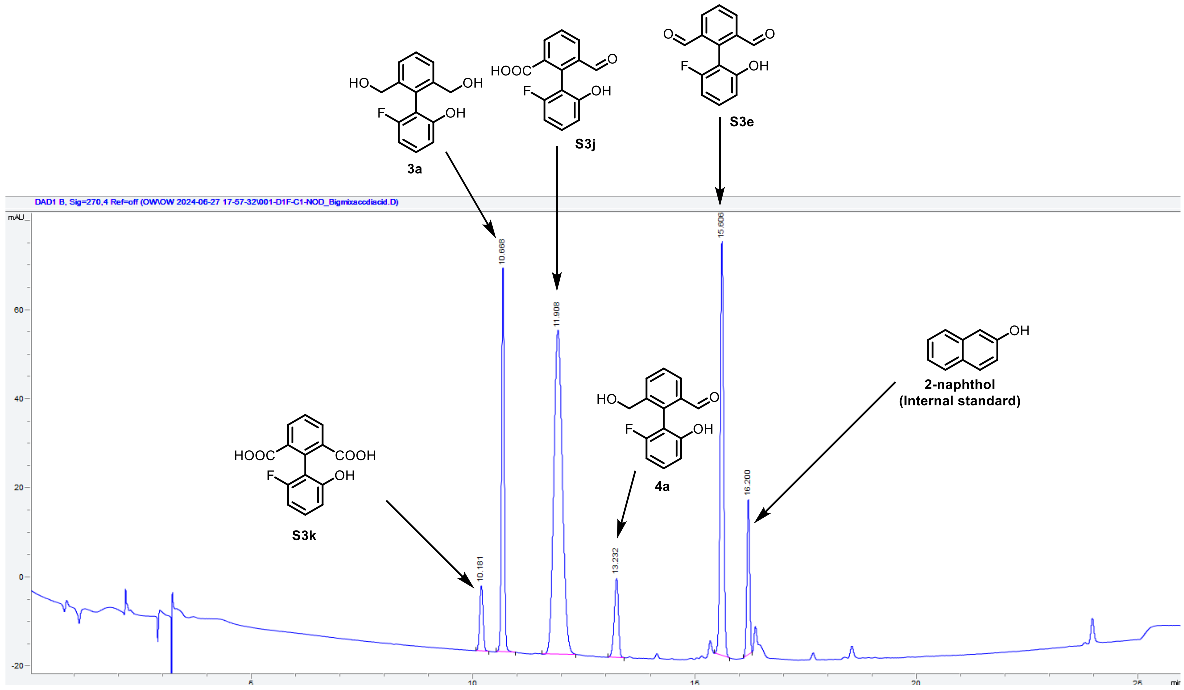

Figure S36 Chromatogram containing **3a**, potential oxidation products (**4a**, **S3e**, **S3g**, **S3j**, **S3k**), and 2-naphthol.

The results from this delayed fuelling experiment confirm that the oxidation system (ADH 291, YcnD and NADP) remains viable for at least 96 h.

8.5) Analysis of the Fuel-to-Waste Reaction

The fuel-to-waste reaction ($H_3N{\cdot}BH_3$ to $B(OH)_3$) was studied by monitoring the *in situ* formation of $B(OH)_3$ using a quantitative ^{11}B NMR spectroscopy method based on a study conducted by Frenich and Fernández.[12]

8.5.1) Calibration Curves

Solutions of $H_3N{\cdot}BH_3$ and $B(OH)_3$ at varying concentrations were prepared independently of each other in NaPi (100 mM, pH 7.0). The standard solutions were transferred to thin wall quartz NMR tubes and analysed by ^{11}B NMR (128 MHz) using the following parameters:

FID data points (TD) = 16384, Relaxation delay = 0.1 s, Acquisition time = 0.64 s, SW = 200 ppm, NS = 400, Fixed receiver gain (RG) = 50. Auto-gain and gradient shimming were turned off.

The spectra were imported into MestreNova and automatic phase and baseline correction were performed (Figures S37 & S39 below). The absolute integrals of the ^{11}B NMR signals for either $H_3N{\cdot}BH_3$ or $B(OH)_3$ were taken and plotted versus concentration (mM) to obtain calibration curves, as detailed in Figures S38 and S40 below.

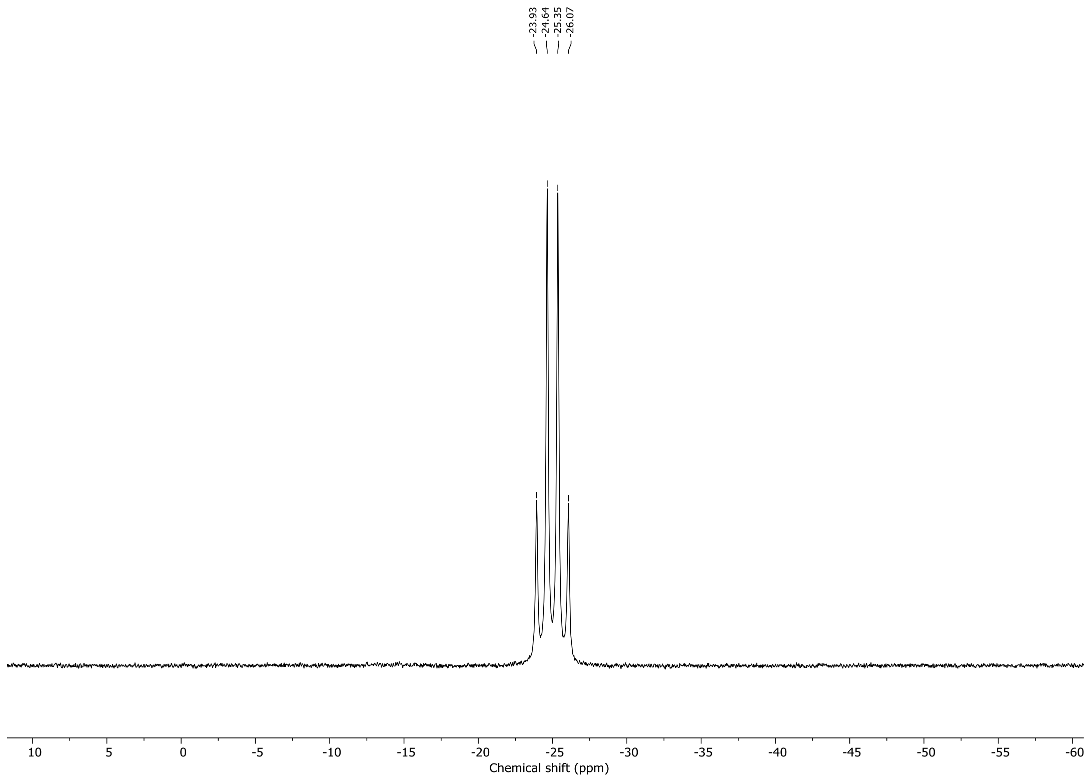

Figure S37 ^{11}B NMR (128 Hz) spectrum of $H_3N{\cdot}BH_3$ (100 mM) in NaPi (100 mM, pH 7.0).

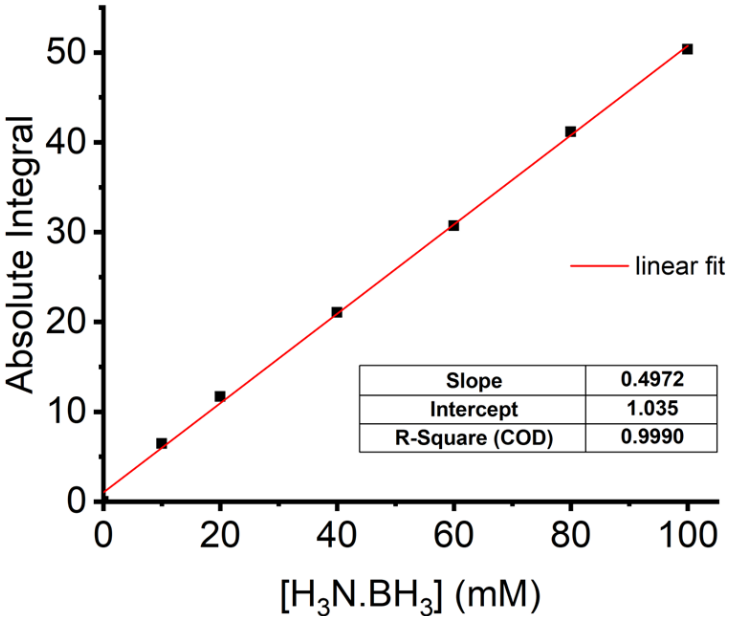

Figure S38 Calibration curves for $H_3N{\cdot}BH_3$ in NaPi (100 mM, pH 7.0) using ^{11}B NMR spectroscopy.

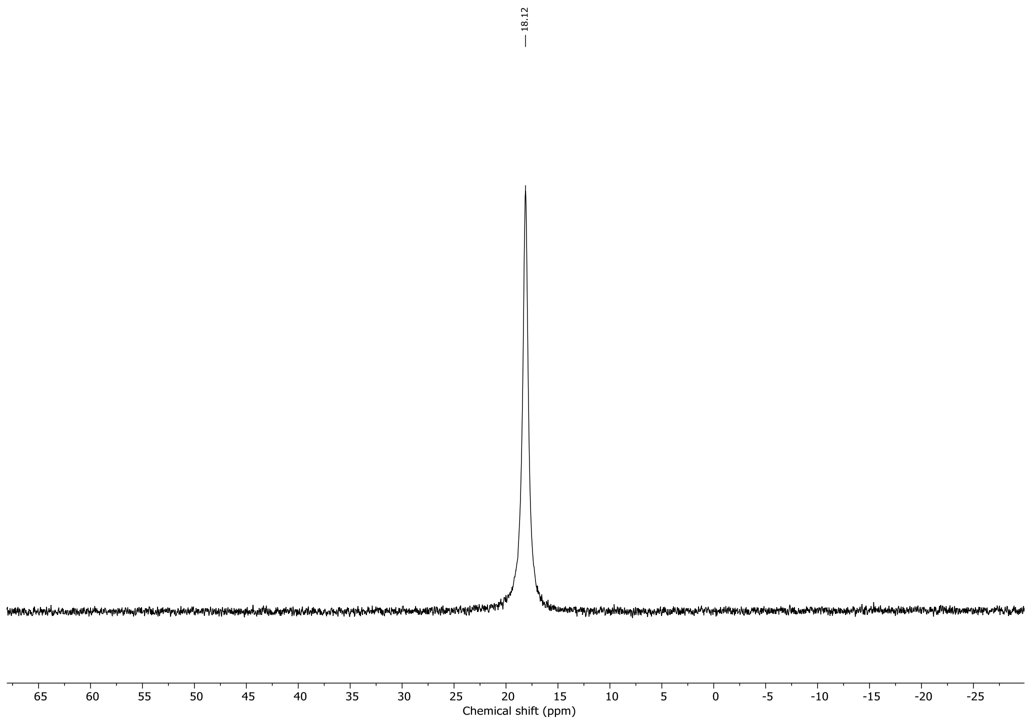

Figure S39 ^{11}B NMR (128 Hz) spectrum of $B(OH)_3$ (60 mM) in NaPi (100 mM, pH 7.0).

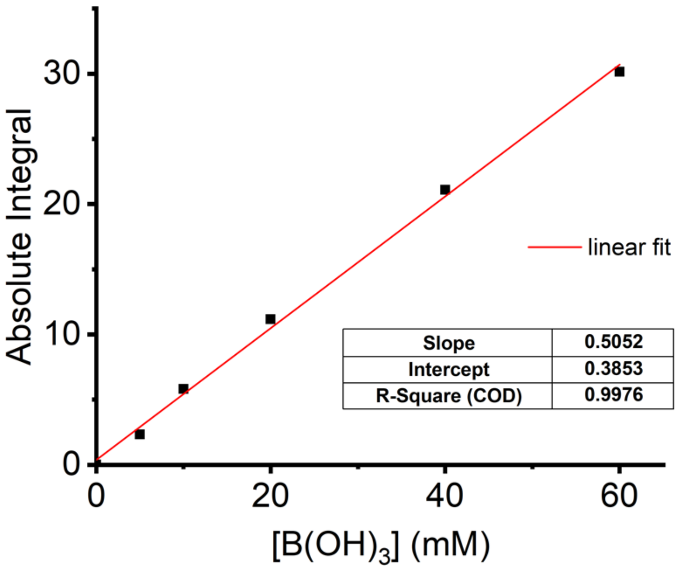

Figure S40 Calibration curves for $B(OH)_3$ in NaPi (100 mM, pH 7.0) using ^{11}B NMR spectroscopy.

8.5.3) Analysis of the Fuel-to-Waste Reaction for the Operation of 3a

ADH 291 (35 U/mL) and NaPi (100 mM, pH 7.0) were added to a 1.5 mL Eppendorf vial and shaken at the stated temperature for 2 min. **3a** (10 mM final concentration) in DMSO (10% v:v), YcnD (60 µM) and $H_3N{\cdot}BH_3$ (100 mM) were added consecutively, followed by NADP (5 mM). The final volume was 600 µL. The vial was sealed and shaken at 40 °C and 300 rpm for 48 h. After this time, the reaction mixture was directly transferred to a thin wall quartz NMR tube and analysed by ^{11}B NMR (128 MHz) to determine the absolute concentrations of $H_3N{\cdot}BH_3$ and $B(OH)_3$ using the calibration curves in Figures S38 and S40 above (see Scheme S13 and Table S14 below). In these experiments, the high concentrations of ADH 291 freeze dried cell lysate result in the formation of a heterogeneous solution, and reaction mixtures were centrifuged for 10 min (14800 rpm) prior to being transferred to NMR tubes for analysis.

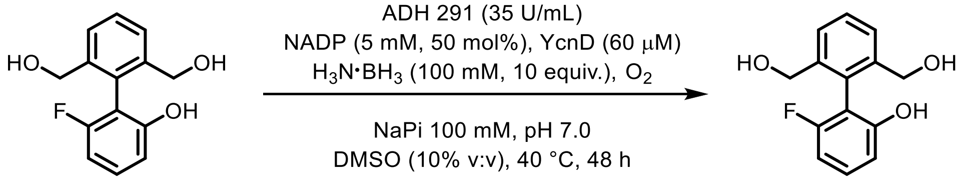

3a, 10 mM **3a**

Scheme S13 Optimized conditions for the operation of **3a** under the cyclic redox reaction network.

Table S14 Absolute concentration of $H_3N{\cdot}BH_3$ and $B(OH)_3$ after 48 hours under optimized conditions for the operation of **3a** under the cyclic redox reaction network in the presence and absence of **3a**. For experiments in the absence of **3a**, DMSO (60 μL) added to ensure DMSO (10% v:v).

Entry	3a / 10 mM	[$H_3N{\cdot}BH_3$] / mM	[$B(OH)_3$] / mM
1	no	41.30 ± 1.27	42.21 ± 0.63
2	yes	34.46 ± 4.92	50.56 ± 3.86

Under the optimized conditions for the operation of **3a** under the cyclic reaction network ($H_3N{\cdot}BH_3$ (100 mM), ADH 291 (35 U/mL), YcnD (60 μM), NADP (5 mM), pH 7.0, 40 °C), the presence of biaryl **3a** increased the rate of formation of $B(OH)_3$ over a 48 h time period (Table S14, Entries 1 and 2) from 42.21 ± 0.63 mM to 50.56 ± 3.86 mM .

9) Determining the directionality of the rotation

Confirmation that **3a** undergoes continuous directional rotation under the conditions of the cyclic redox reaction network requires evidence that the oxidation of **3a** proceeds stereoselectively, i.e., that $r_{ox(Sa)}/r_{ox(Ra)} \neq 1$. This information cannot be provided by direct observation of enantiomeric enrichment in either starting material or product, because **3a** is achiral, and (*R*a)-**4a** and (*S*a)-**4a** racemise too fast for analysis of the enantiomeric ratio. The enantioselectivity of the oxidation of motor candidate **3a** can nonetheless be deduced from the fate of isotopic labels within its benzylic methylene groups. The selectivities of oxidations of enantiopure isotopomers (*S*a)-D2-**3a** and (*R*a)-D2-**3a** are detailed in this section, and our interpretation of the results is validated by comparison with equivalent experiments using **S8e** for which enantioselectivity may also be determined by independent methods, i.e., chiral HPLC.

9.1) Biocatalytic oxidation of deuterated and enantioenriched (*S*a)-D2-S8e, (*R*a)-D2-S8e and unlabelled S8e

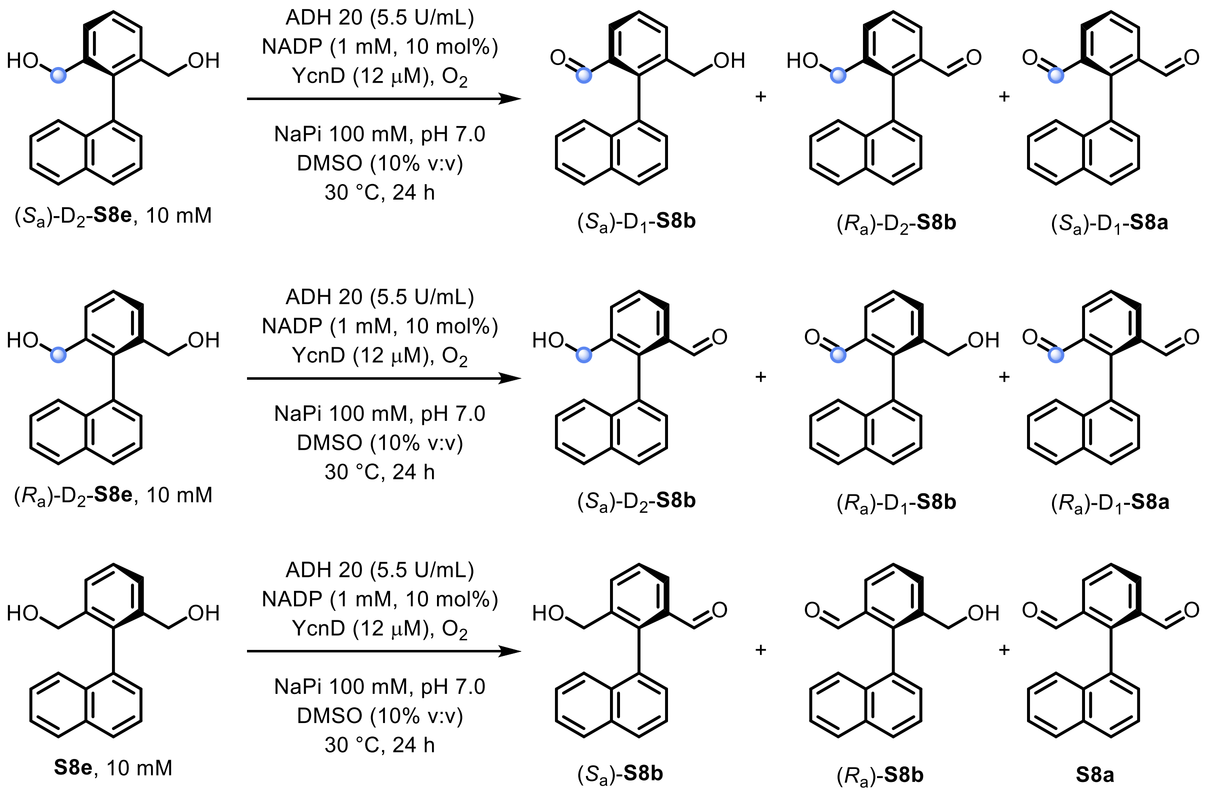

Scheme S14 Biocatalytic oxidation of deuterated and enantioenriched (*S*a)-D2-**S8e**, (*R*a)-D2-**S8e** and unlabelled **S8e**.

In six separate 2 mL Eppendorf vials were added NaPi 100 mM pH 7.0, ADH 20 (5.5 U/mL), YcnD (12 µM), and NADP (1 mM). Either (S_a)-D_2-**S8e**, (R_a)-D_2-**S8e** or non-labelled **S8e** (10 mM final concentration) in DMSO (40 µL) were added. The final volume was 400 µL. The vials were sealed, and the colourless suspensions were shaken at 30 °C for 24 h. Each reaction mixture was extracted with MTBE (3 × 1000 µL). The combined organic layers were dried over Na_2SO_4, filtered, and transferred to a HPLC vial for analysis of the crude mixture (Chiralpak IA, 250 × 4.6 mm, 5 µm, 1 mL/min, eluent composition and gradient detailed in Table S15, λ = 254 nm, t_R = **S8a**, 8.4 min; (R_a)-**S8b**, 40.1 min; (S_a)-**S8b**, 42.9 min; **S8e**, 55.5 min).

Table S15 Gradient conditions for the analysis of mixtures of **S8e**, **S8b** and **S8a** on Chiralpak IA, 1 mL/min, 70 min run.

Time (min)	hexane:IPA
0	98:2
30	98:2
45	95:5
50	80:20
60	80:20
62	98:2
70	98:2

Table S16 Conversion of **S8e** to **S8b** and **S8a** as measured by HPLC.

Entry	Substrate	% remaining **S8e**	% **S8b**	% **S8a**
1	(S_a)-D_2-**S8e**	32	64	4
2	(S_a)-D_2-**S8e**	32	64	4
3	(R_a)-D_2-**S8e**	13	86	1
4	(R_a)-D_2-**S8e**	11	88	1
5	**S8e**	10	87	3
6	**S8e**	10	86	3

The lower conversion of (S_a)-D_2-**S8e** (Table S16, Entries 1 and 2) compared to (R_a)-D_2-**S8e** (Table S16, Entries 3 and 4) and unlabelled **S8e** (Table S16, Entries 5 and 6) is a consequence of a kinetic isotope effect: ADH 20 preferentially attacks the pro-S_a benzylic alcohol (see below), which contains the deuterium label in the case of (S_a)-D_2-**S8e**, but not (R_a)-D_2-**S8e** and unlabelled **S8e**.

After HPLC analysis, each sample was purified by column chromatography on silica gel (CH_2Cl_2) to afford monoaldehyde **S8b** as a colourless solid (yields undetermined). **S8b** was analysed by ^{1}H NMR (600 MHz, 32 scans, D1 = 30 s) (Figure S41).

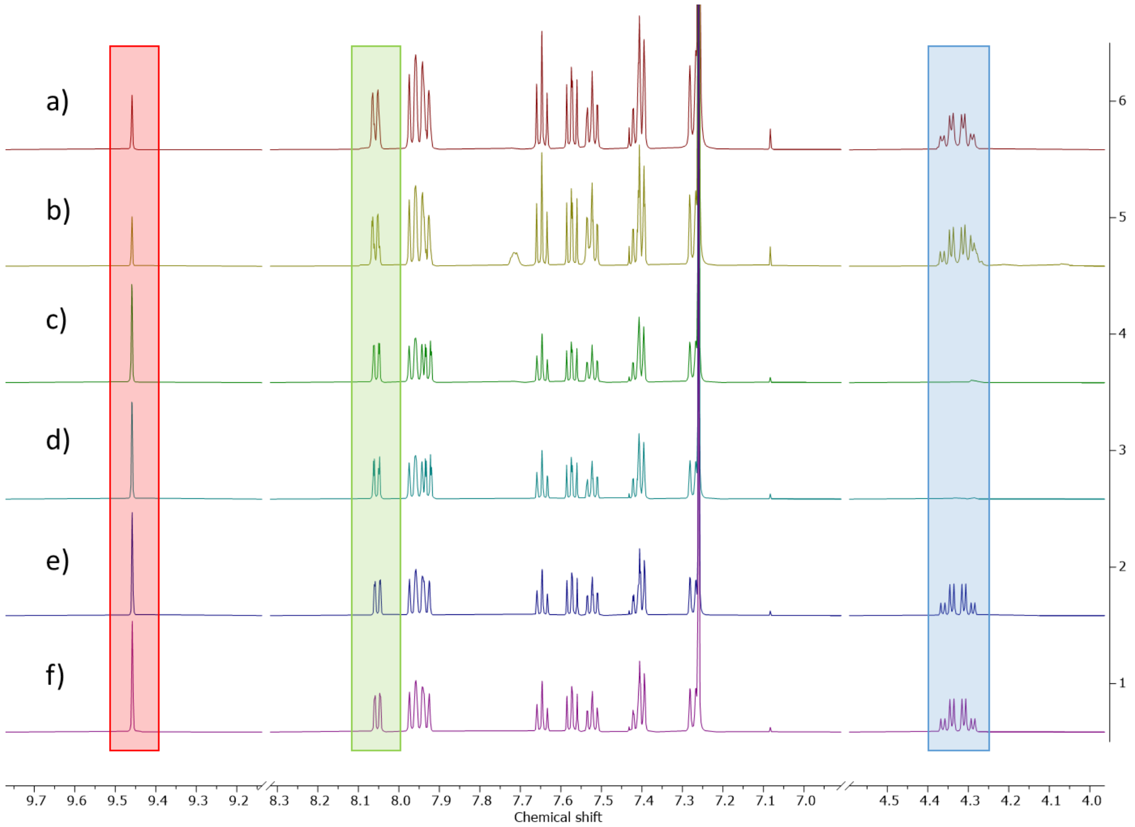

Figure S41 (a)-(b) ^{1}H NMR spectra (in $CDCl_3$) of monoaldehyde **S8b** from the biocatalytic oxidation of (*S*$_a$)-D$_2$-**S8e**. (c)-(d) ^{1}H NMR spectra of monoaldehyde **S8b** from the biocatalytic oxidation of (*R*$_a$)-D$_2$-**S8e**. (e)-(f) ^{1}H NMR spectra of monoaldehyde **S8b** from the biocatalytic oxidation of unlabelled **S8e**. The green stripe highlights the aryl signal that was used as an internal reference for integration (set to 1.00). The red stripe highlights the aldehyde signal. The blue stripe highlights the benzylic alcohol signal.

In each of the NMR spectra above, the relative integration of the aldehyde signal (highlighted in red) gives the ratio of CHO to CDO aldehyde, indicating the ratio of (*R*$_a$)-D$_2$-**S8b** to (*S*$_a$)-D$_1$-**S8b** formed from diol (*S*$_a$)-D$_2$-**S8e** and the ratio of (*S*$_a$)-D$_2$-**S8b** to (*R*$_a$)-D$_1$-**S8b** formed from diol (*R*$_a$)-D$_2$-**S8e**. In addition, in each of the NMR spectra above, the relative integration of the benzylic alcohol signal (highlighted in blue) gives the ratio of CD_2OH to CH_2OH aldehyde, which indicates the ratio of (*R*$_a$)-D$_2$-**S8b** to (*S*$_a$)-D$_1$-**S8b** formed from diol (*S*$_a$)-D$_2$-**S8e** and the ratio of (*S*$_a$)-D$_2$-**S8b** to (*R*$_a$)-D$_1$-**S8b** formed from diol (*R*$_a$)-D$_2$-**S8e**.

Validation of the method described above for the accurate determination of enantioselectivity is achieved by independent determination of the enantiomeric ratios of these biocatalytic oxidations using HPLC on a chiral stationary phase, as detailed in Table S17 and Figure S42 below.

Table S17 Integrations of the aldehyde and benzylic alcohol signals in the ^{1}H NMR spectra of (deuterated) monoaldehyde **S8b**. Enantiomeric ratio of product **S8b** measured by HPLC (Chiralpak IA, 250 × 4.6 mm, 5 µm, 1 mL/min, eluent composition and gradient detailed in Table S15, λ = 254 nm, t_R = **S8a**, 8.4 min; (*R*a)-**S8b**, 40.1 min; (*S*a)-**S8b**, 42.9 min; **S8e**, 55.5 min).

		**^{1}H NMR of monoaldehyde S8b**				**HPLC**
Entry	Substrate	**Aldehyde signal integration**	Aldehyde CHO / CDO ratio	**Alcohol signal integration**	Alcohol CD_2O / CH_2O ratio	e.r. of **S8b** (*R*a):(*S*a)
1	(*S*a)-D2-**S8e**	0.25	25:75	1.64	18:82	20.5:79.5
2	(*S*a)-D2-**S8e**	0.25	25:75	1.64	18:82	21:79
3	(*R*a)-D2-**S8e**	0.95	95:5	0.08	96:4	2:98
4	(*R*a)-D2-**S8e**	0.97	97:3	0.07	96.5:3.5	1.5:98.5
5	**S8e**	0.97	-	1.99	-	6:94
6	**S8e**	0.98	-	1.98	-	6:94

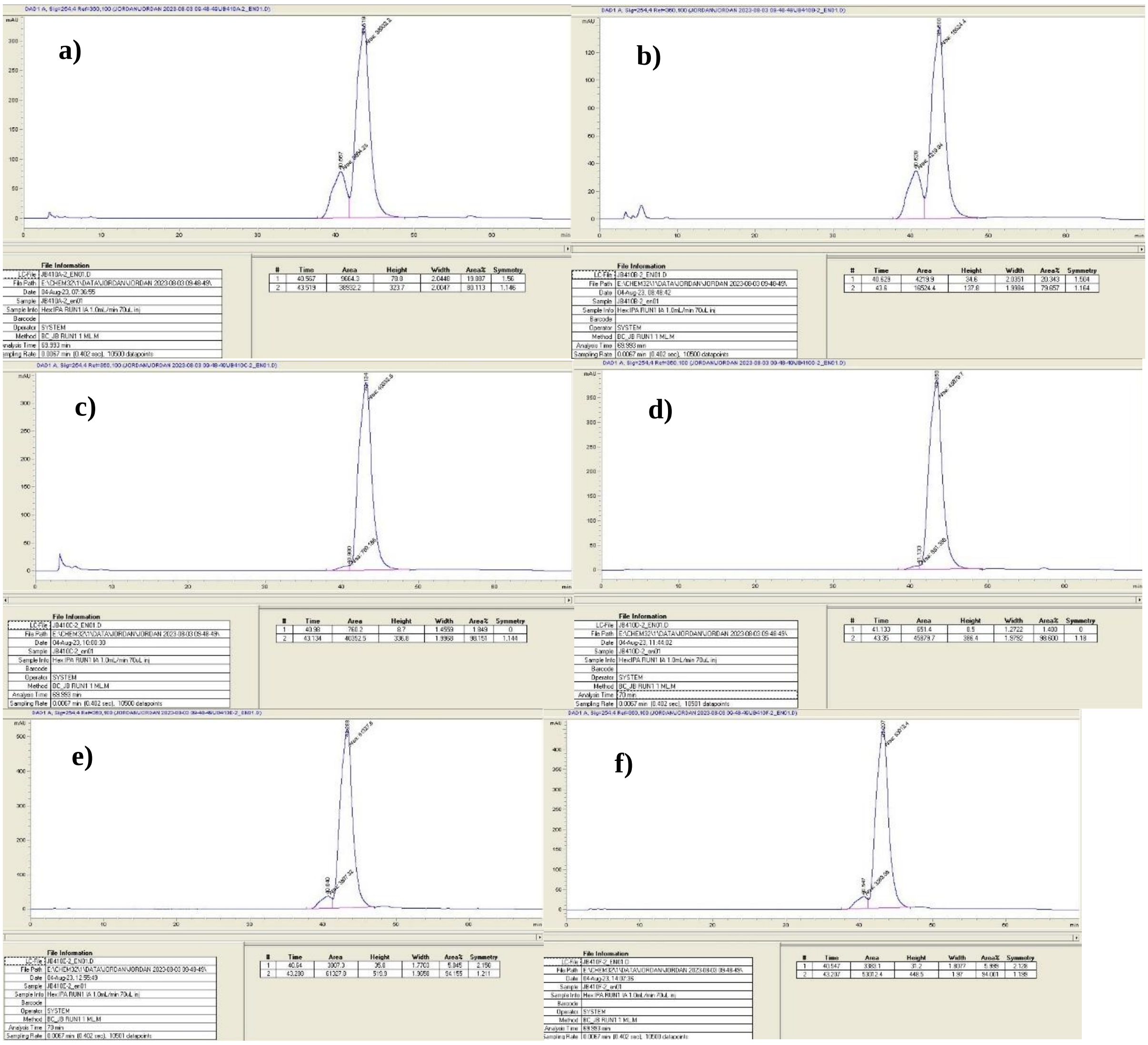

Figure S42 (a)-(b) HPLC spectra of monoaldehyde **S8b** from the biocatalytic oxidation of (*S*$_a$)-D$_2$-**S8e**. (c)-(d) HPLC spectra of monoaldehyde **S8b** from the biocatalytic oxidation of (*R*$_a$)-D$_2$-**S8e**. (e)-(f) HPLC spectra of monoaldehyde **S8b** from the biocatalytic oxidation of unlabelled **S8e**. Enantiomeric ratio of product **S8b** measured by HPLC (Chiralpak IA, 250 × 4.6 mm, 5 µm, 1 mL/min, eluent composition and gradient detailed in Table S15, λ = 254 nm, t_R = **S8a**, 8.4 min; (*R*$_a$)-**S8b**, 40.1 min; (*S*$_a$)-**S8b**, 42.9 min; **S8e**, 55.5 min).

As can be seen from Table S17, the enantiomeric ratios determined by this method of using NMR to quantify ratios of isotopomers, and those determined from chiral HPLC, are in close agreement.

The lower enantioselectivity observed for the oxidation of (*S*$_a$)-D$_2$-**S8e** (Table S17, Entries 1 and 2) compared to unlabelled **S8e** (Table S17, Entries 5 and 6) is a consequence of a kinetic isotope effect: ADH 20 preferentially attacks the pro-*S*$_a$ benzylic alcohol, which contains the deuterium label in the case of (*S*$_a$)-D$_2$-**S8e**. This leads to a decrease in enantioselectivity, as the ratio $k_{ox(S)}$ / $k_{ox(R)}$ decreases.

In addition, the higher enantioselectivity observed for the oxidation of (R_a)-D_2-**S8e** (Table S17, Entries 3 and 4) compared to unlabelled **S8e** (Table S17, Entries 5 and 6) is a consequence of a kinetic isotope effect: ADH 20 preferentially attacks the pro-S_a benzylic alcohol, which **does not** contain the deuterium label in the case of (R_a)-D_2-**S8e**. This leads to an increase in enantioselectivity, as the ratio $k_{ox(S)}$ / $k_{ox(R)}$ increases.

The enantioselectivity of the biocatalytic oxidation on the unlabelled diol **S8e** (Table S17, Entries 5 and 6) sits within the range of the two deutero-enantiomers.

This experiment demonstrates that the ^{1}H NMR spectroscopy method described above can be used to determine directly the enantioselectivity of biaryl diol desymmetrisation reactions. Its application to **3a**, whose oxidation product is configurationally unstable, is described in Supplementary Information Section 9.2 below.

9.2) Biocatalytic oxidation of deuterated and enantioenriched (S_a)-D_2-3a, (R_a)-D_2-3a and unlabelled 3a

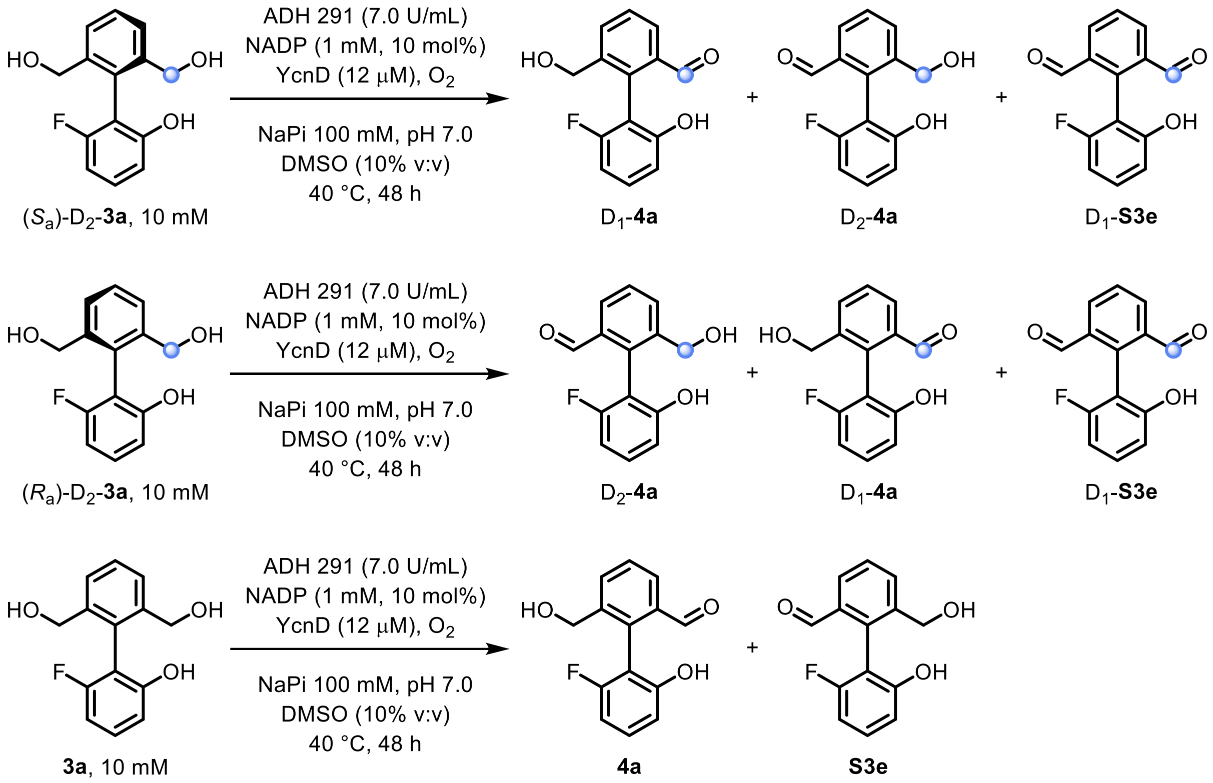

Scheme S15 Biocatalytic oxidation of deuterated and enantioenriched (S_a)-D_2-**3a**, (R_a)-D_2-**3a** and unlabelled **3a**.

In nine separate 2 mL Eppendorf vials were added NaPi 100 mM pH 7.0, ADH 291 (7.0 U/mL), YcnD (12 μM), and NADP (1 mM). Either (S_a)-D_2-**3a**, (R_a)-D_2-**3a** or non-labelled **3a** (10 mM final concentration) in DMSO (40 μL) were added. The final volume was 400 μL. The vials were sealed, and the colourless suspensions were shaken at 40 °C for 48 h. Each reaction mixture was extracted with MTBE (3 × 1000 μL). The combined organic layers were dried over Na_2SO_4, filtered, and transferred to a HPLC vial for analysis of the crude mixture.

Table S19 Conversion of **3a** as measured by HPLC.

Entry	Substrate	% remaining **3a**	% **4a**	% **S3e**
1	(S_a)-D_2-**3a**	76	23	1
2	(S_a)-D_2-**3a**	76	23	1
3	(S_a)-D_2-**3a**	76	23	1
4	(R_a)-D_2-**3a**	55.5	44	0.5
5	(R_a)-D_2-**3a**	54	45	0.5
6	(R_a)-D_2-**3a**	55	44.5	0.5
7	**3a**	60	39	1
8	**3a**	59	40	1
9	**3a**	60	39	1

The lower conversion of (S_a)-D_2-**3a** (Table S19, Entries 1–3) compared to (R_a)-D_2-**3a** (Table S19, Entries 4–6) and unlabelled **3a** (Table S19, Entries 7–9) is a consequence of a kinetic isotope effect: ADH 291 preferentially attacks the pro-S_a benzylic alcohol (see below), which contains the deuterium label in the case of (S_a)-D_2-**3a**, but not (R_a)-D_2-**3a** and unlabelled **3a**.

After HPLC analysis, each sample was purified by column chromatography on silica gel (hexane:EtOAc, 70:30) to afford monoaldehyde **4a** as a colourless solid (yields undetermined). **4a** was analysed by ^{1}H NMR (600 MHz, 32 scans, D1 = 30 s) (Figure S43).

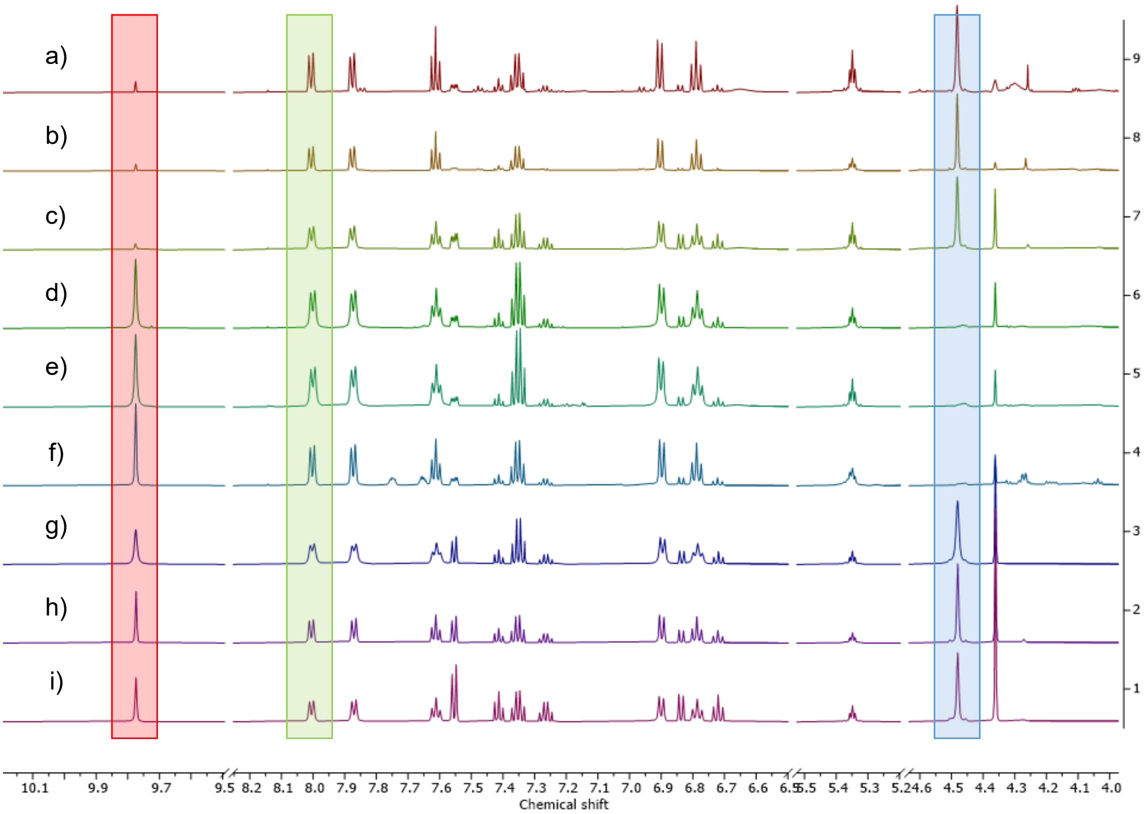

Figure S43 (a)-(c) ^{1}H NMR spectra (in $(CD_3)_2CO$) of monoaldehyde **4a** from the biocatalytic oxidation of (S_a)-D_2-**3a**. (d)-(e) ^{1}H NMR spectra of monoaldehyde **4a** from the biocatalytic oxidation of (R_a)-D_2-**3a**. (g)-(i) ^{1}H NMR spectra of monoaldehyde **4a** from the biocatalytic oxidation of unlabelled **3a**. The green stripe highlights the aryl signal that was used as an internal reference for integration (set to 1.00). The red stripe highlights the aldehyde signal. The blue stripe highlights the benzylic alcohol signal.

In each of the NMR spectra above, the relative integration of the aldehyde signal (highlighted in red) gives the ratio of CHO to CDO aldehyde, indicating the ratio of D_2-**4a** to D_1-**4a**. In addition, in each of the NMR spectra above, the relative integration of the benzylic alcohol signal (highlighted in blue) gives the ratio of CD_2OH to CH_2OH aldehyde, indicating the ratio of D_2-**4a** to D_1-**4a**.

The ratios obtained from the integration of either the aldehyde or the benzylic signal should be the same and are presented in Table S20. These ratios represent the enantioselectivities of the biocatalytic oxidations of (S_a)-D_2-**3a** and (R_a)-D_2-**3a** (Table S21), even though the oxidation product **4a** racemises rapidly.

Table S20 Integrations of aldehyde and benzylic alcohol signals in the ^{1}H NMR spectra of (deuterated) **4a**.

		^{1}**H NMR of monoaldehyde 4a**			
Entry	Substrate	**Aldehyde signal integration**	Aldehyde CHO / C**D**O ratio	**Alcohol signal integration**	Alcohol C**D**$_2$O / CH$_2$O ratio
1	(S_a)-D$_2$-**3a**	0.10	10 : 90	1.86	7 : 93
2	(S_a)-D$_2$-**3a**	0.10	10 : 90	1.93	3.5 : 96.5
3	(S_a)-D$_2$-**3a**	0.09	9 : 91	1.93	3.5 : 96.5
4	(R_a)-D$_2$-**3a**	0.96	96 : 4	0.13	93.5 : 6.5
5	(R_a)-D$_2$-**3a**	0.96	96 : 4	0.13	93.5 : 6.5
6	(R_a)-D$_2$-**3a**	0.96	96 : 4	0.13	93.5 : 6.5
7	**3a**	0.96	-	2.02	-
8	**3a**	0.96	-	2.04	-
9	**3a**	0.98	-	2.07	-

Table S21 Calculated enantiomeric ratios and excesses obtained in the biocatalytic oxidation of **3a** to monoaldehyde **4a**. The errors given are the sample standard deviation.

Entry	Ratio	Corresponding %ee	Ratio	Corresponding %ee	Average %ee	Overall average	%Standard deviation
1	10 : 90	80	7 : 93	86	83	85.7% ee (or 7:93 e.r.)	6.1
2	10 : 90	80	3.5 : 96.5	93	86.5		
3	9 : 91	82	3.5 : 96.5	93	87.5		
4	96 : 4	92	93.5 : 6.5	87	89.5	89.5% ee (or 95:5 e.r.)	2.7
5	96 : 4	92	93.5 : 6.5	87	89.5		
6	96 : 4	92	93.5 : 6.5	87	89.5		

The results in Tables S20 and S21 confirm that the ^{1}H NMR integrations of the aldehyde and benzylic alcohol signals are in close agreement.

By analogy with the experiments performed on model **S8e** (Supplementary Information Section 9.1), the slightly lower enantioselectivity observed for the oxidation of (S_a)-D$_2$-**3a** (Table S21, Entries 1–3) compared to (R_a)-D$_2$-**3a** (Table S21, Entries 4–6) is a consequence of a kinetic isotope effect: ADH 291 preferentially attacks the pro-S_a benzylic alcohol, which contains the deuterium label in the case of (S_a)-D$_2$-**3a** but not (R_a)-D$_2$-**3a**. This leads to a decrease in enantioselectivity when starting from (S_a)-D$_2$-**3a**,

as the ratio $k_{ox(S)}$ / $k_{ox(R)}$ decreases but leads to an increase in enantioselectivity when starting from (R_a)-D_2-**3a**, as the ratio $k_{ox(S)}$ / $k_{ox(R)}$ increases.

By analogy with the experiments performed on model **S8e** (Supplementary Information Section 9.1), the enantioselectivity of the biocatalytic oxidation of unlabelled **3a** (Table S20, Entries 7–9) to **4a** must sit within the range defined by the two deutero-enantiomers, i.e. 85.7–89.5% ee (Table S21). An averaged value of 87.6% ee (93.8% in er) is used for the simulation of rotational outcomes in a statistically relevant population of **3a**, as described in Supplementary Section S11.

This experiment demonstrates that the biocatalytic oxidation of **3a** is enantioselective, even though **4a** racemises rapidly. The experiment thus confirms that the expression $r_{ox(Sa)}/r_{ox(Ra)} \neq 1$ is true, and hence provides the first direct evidence of directional motion in an operational single bond rotary motor.

10) Mean number of 360° rotations after 48 hours of operation

To calculate the mean number of 360° rotations after 48 h of operation, the half-life of the slowest step (oxidation), the selectivity of both the oxidation and the reduction reactions, the probability of rotation after oxidation, the timeframe of operation, and the number of oxidation reactions that occur in the oxidation half-life are required. The Poisson distribution (Figure S44) provides the probability that n number of events occur within a fixed timeframe, here oxidation events per half-life.[13]

$$P(r = n) = \frac{\lambda^n}{n!}e^{-\lambda}$$

Figure S44: The Poisson probability function of a number of events in the same time interval, P = probability, r = number of events, λ = expected value.[13]

By definition, the probability of one molecule undergoing zero oxidation events (r = 0) is 0.5 (P = 0.5). From this we can determine λ (derivation shown Figure S45), the average number of oxidation events per molecule per half-life, as $\lambda = \ln(2) \approx 0.693$.

$$P(r = 0) = 0.5 = \frac{\lambda^0}{0!}e^{-\lambda} = e^{-\lambda} \text{ therefore } \lambda = \ln(2)$$

Figure S45: The Poisson probability function for zero oxidation events (r = 0) per half-life.

From this result we can calculate the probability that a molecule of **3a** undergoes any number of oxidation events (e.g., r = 0, 1, 2 …) per half-life (Figure S46).

$$P(r = n) = \frac{(\ln(2))^n}{n!}e^{-\ln(2)}$$

Figure S46: The probability that a molecule will undergo n number of oxidation events per half-life.

To calculate the mean rotation in a given timeframe, *t*, the following general expression can be used (Figure S47).

$$mean\ rotation(°) = \lambda \times (P_{desired_{ox}} - P_{undesired_{ox}}) \times (\frac{r_{rac}}{r_{rac} + r_{red}}) \times (P_{directional\_red}) \times 180° \times \frac{t}{t_{1/2}}$$

Figure S47: General expression for the calculation of the mean rotation in a given timeframe, *t*. λ = expectation value, $P_{desired\_ox}$ = probability of undergoing the desired enantioselective oxidation, $P_{undesired\_ox}$ = probability of undergoing the undesired enantioselective oxidation, r_{rac} = rate of racemization, r_{red} = rate of reduction, $P_{directional\_red}$ = probability of undergoing a directional reduction, 180° to convert to degrees, t = operation timeframe, $t_{1/2}$ = half-life of the slowest step (the oxidation of **3a** to **4a**).

Using the following values: λ = ln(2) (see Figure S45); $P_{desired\_ox}$ = 0.938, $P_{undesired\_ox}$ = 0.062 (see Supplementary Information Section 9.2); rate of racemization $r_{rac} = 2r_{enant} = 8.4 \times 10^{-1} \cdot$ [**4a**] mM.s^{-1} (see Supplementary Information Section 7.3); rate of reduction $r_{red} = 2.1 \times 10^{-3} \cdot [H_3N{\cdot}BH_3] \cdot$ [**4a**]

mM.s^{-1} where [$H_3N{\cdot}BH_3$] = 100 mM (see Supplementary Information Section 7.2); $P_{directional\_red}$ = 0.5 (the reduction is unselective); t = 48 h; oxidation half-life $t_{1/2}$ = 2.48 h (see Supplementary Information Section 7.1), allows us to calculate the mean rotation over a 48 h period as 846°, which is 2.35 full 360° rotations (Figure S48).

$$mean\ rotation(°) = \ln(2) \times 0.876 \times \frac{0.84[\mathbf{4a}]}{(0.84[\mathbf{4a}] + 0.21[\mathbf{4a}])} \times 0.5 \times 180° \times \frac{48}{2.48} = 846°$$

Figure S48: Calculation of the mean rotation after 48 h of operation. λ = ln(2); $P_{desired\_ox}$ - $P_{undesired\_ox}$ = 0.876; r_{rac} = $2r_{enant}$ = 0.84**[4a]**; r_{red} = 0.21**[4a]** ([$H_3N{\cdot}BH_3$] = 100 mM]); $P_{directional\_red}$ = 0.5; 180° to convert to degrees; t = 48 h; $t_{1/2}$ = 2.48 h.

11) Code for simulating rotations

The following code was used to simulate the rotational outcome of a population of 10^7 molecules of **3a** after 48 h. The code was run using Python 3.7.12 in a Jupyter notebook, with the libraries numpy and matplotlib.[14–17.]

The code works by first defining the functions to determine how many oxidation events (**3a** to **4a**) each molecule of **3a** in the 10^7 population undergoes per half-life, where the half-life is the time at which exactly half of the population has undergone zero oxidation events (the experimental value of 2.48 h for the half-life of the oxidation of **3a** to **4a** is provided in Section 7.1). The oxidation events per molecule of **3a** are simulated using a Poisson distribution, where the function is termed 'poisson_prob(r)' in the code, with variable 'r' being the number of times a molecule is oxidised per half-life.

The function, 'drop_random_poisson(arr, drop_prob)' is defined with the variables 'arr' and 'drop_prob'. The function takes the input array and removes elements based on the probability given, outputting two arrays, one with the dropped elements removed, and the other an array of the dropped elements.

A number of desired variables may then be set, such as time (48 h), oxidation rate (half-life = 2.48 h, Supplementary Information Section 7.1), enantioselectivity (0.938; Supplementary Information Section 9.2), reduction selectivity (unselective = 0.5), [$H_3N{\cdot}BH_3$] (100 mM), the rate of racemization (0.84 s^{-1}; Supplementary Information Section 7.3), the rate of reduction ($2.1 \times 10^{-3} \times [H_3N{\cdot}BH_3]\ s^{-1}$; Supplementary Information Section 7.2), and population size (10^7). The rate of racemization and reduction are both given as relative values: the **[4a]** term cancels, as shown in Supporting Information Section 10.

A working array of zeros called 'pop' is created, with the defined population size. The elements of this array are the molecules being simulated. The number of half-lives was derived by dividing the time by the length of the half-life; in the code, this number is handled as an integer value plus the remainder. The probability a molecule will undergo racemization in an oxidation event is calculated. The selectivity of the oxidation, reduction, and probability of racemisation are used to create a list for the probabilities of each rotation occurring per oxidation event, with the order set as forwards rotation, no rotation and backwards rotation. The probability of a 0° rotation occurring per oxidation event is 50% since we are working with a non-selective reduction.

The rotation each molecule has undergone per half-life is then simulated, inside a loop. The drop probability is calculated from the probability given by the Poisson distribution (detailed in Supporting Information Section 10 with λ = ln(2)), multiplied by the initial population over the remaining population. This gives the probability a molecule would be dropped from the remaining population at this number of oxidation events. When the number of oxidation events is greater than 0, the probabilities given by the oxidation and reduction selectivities are used to simulate the final rotation state of the population of molecules. The array is split in two using the 'drop_random_poisson' function, outputting a now smaller remaining 'pop' array, used for later oxidation events up to r = 20, and the removed population 'popped_this_cycle'. The rotational values in 'popped_this_cycle' are added to the list 'popped_values'. The rotational values appearing in the 'popped_values' list are then counted, returning their final rotational values as the array 'unique_values' and the frequency of these rotational values as 'counts'. The 'counts' are then normalised, giving the probability for each final rotational value per half-life in the array 'normalized_probabilities'.

The array 'pop' is replaced with another array filled with zeros of size 10^7. The elements of this array are the simulated molecules undergoing the reaction. The array 'pop' goes through a loop simulating the oxidations occurring in each half-life with the rotational value and probability of undergoing that rotational value given by the array 'unique_values' and 'normalized_probabilities', respectively. The results from each half-life are gathered in a further array called 'samples', which are then added to array 'pop'.

Any half-life remainder is then simulated. To represent this remainder, the probabilities of all rotational values in array 'normalized_probabilities' are multiplied by the value of the half-life remainder, and the probability of 0° is set to 0. The sum of the resulting probability array 'partial_cycle_probs' is calculated and the probability of 0° is set to [1 – 'partial_cycle_probs.sum()'], and the array is normalised. The molecules then undergo the rotation corresponding to the half-life remainder, and this is added to the array 'pop'.

A histogram is then generated (Fig. 4c of the manuscript and Figure S49 below), using the final 'pop' array generated as a result of these iterations, to give the plot for the simulated rotation for the 10^7 molecules of **3a**. The rest of the code sets the y-axis to a % of the total, instead of the population at that rotation, and for formatting and adding visual aids to the chart. Due to the nature of probabilities extreme cases (+3780, −900) may appear/disappear from the x-axis which may affect the formatting.

The code outputs the following histogram and summary. The mean rotations can vary slightly depending on the random sampling hence the large population size:

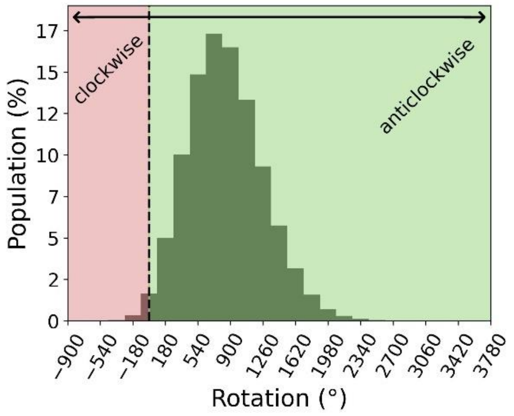

The mean rotation of simulated molecules in 48 h: 846°.

Figure S49: Output of the code, including a histogram illustrating the distribution of the net rotational values of 10^7 simulated molecules of **3a** after 48 h of operation.

For this simulation the mean rotation of the simulated molecules after 48 h of operation is 846°, which is in agreement with the calculated mean rotation of 846°, as described in Supporting Information Section 10.

The code to plot the histogram is included below, and is also available at GitHub (https://github.com/NODbristol/Nature-manuscript-2024-02-03159B)

```
import numpy as np
import matplotlib.pyplot as plt
from matplotlib.ticker import FuncFormatter
from math import exp, factorial, log

#Poisson probability formula
def poisson_prob(r):
    return (exp(-log(2)) * log(2) ** r) / factorial(r)

#Array sampler
def drop_random_poisson(arr, drop_prob):
    num_to_drop = min(int(drop_prob * len(arr)), len(arr))
    drop_indices = np.random.choice(len(arr), size=num_to_drop, replace=False)
    return np.delete(arr, drop_indices), arr[drop_indices]

# Constants
time, half_life = 48, 2.48
oxidation_selectivity, reduction_selectivity = 0.938, 0.5
AB_conc = 100 #mM
r_rac, r_red = 0.84, 0.0021*AB_conc
pop_size = 10**7
num_cycles = time / half_life
integer_cycles, partial_cycle = int(num_cycles), num_cycles % 1


# Probabilities
```

```
rotation_prob = r_rac/(r_rac+r_red)
forwards_prob = reduction_selectivity * oxidation_selectivity * rotation_prob
backwards_prob = (1-reduction_selectivity) * (1 - oxidation_selectivity) *
rotation_prob
prob_per_oxidation = [forwards_prob, 1 - forwards_prob - backwards_prob,
backwards_prob]


# Working out rotations per half-life
pop = np.zeros(pop_size, dtype=int)
popped_values = []
mean_samples = []

for r in range(20):
    drop_prob = poisson_prob(r) * (pop_size / len(pop))
    if r > 0:
        pop += np.random.choice([180, 0, -180], size=len(pop),
p=prob_per_oxidation)
    pop, popped_this_cycle = drop_random_poisson(pop, drop_prob)
    popped_values.extend(popped_this_cycle)

# Normalize probabilities
unique_values, counts = np.unique(popped_values, return_counts=True)
normalized_probabilities = counts / counts.sum()

# Full cycles
pop = np.zeros(pop_size, dtype=int)
for _ in range(integer_cycles):
    samples = np.random.choice(unique_values, size=pop_size,
p=normalized_probabilities)
    pop += samples

# Partial cycle
if partial_cycle > 0:
    partial_cycle_probs = normalized_probabilities * partial_cycle
    partial_cycle_probs[unique_values == 0] = 0
    partial_cycle_probs[unique_values == 0] = 1 - partial_cycle_probs.sum()
    samples = np.random.choice(unique_values, size=pop_size,
p=partial_cycle_probs)
    pop += samples

# Plot results
bin_edges = np.arange(pop.min() - 90, pop.max() + 90, 180)
plt.hist(pop, bins=bin_edges, color='#333333')
plt.xlabel('Rotation (°)', fontsize=20)
plt.ylabel('Population (%)', fontsize=20)
plt.xticks(np.arange(pop.min(), pop.max() + 360, 360), fontsize=16, rotation=60)
plt.gca().yaxis.set_major_formatter(FuncFormatter(lambda x, _: f'{int(x * 100 /
pop_size)}'))
plt.gca().tick_params(axis='y', labelsize=16)
plt.axvline(x=0, color='black', linestyle='--', linewidth=2)
plt.axhline(y=1832000, xmin=.05, xmax=.95, color='black', linewidth=2)
plt.axvspan(min(pop), 0, color='#E18B8A', alpha=0.5)
plt.axvspan(0, max(pop), color='#97D57D', alpha=0.5)
plt.annotate('clockwise', xy=(0, 0), xytext=(min(pop)/2, 1750000), fontsize=16,
ha='center', va='top', rotation=45)
plt.annotate('anticlockwise ', xy=(0, 0), xytext=(max(pop)*(8.8/9), 1750000),
fontsize=16, ha='right', va='top', rotation=45)
```

```
plt.annotate('\u2190', xy=(0, 0), xytext=(min(pop)+180, 1825000), fontsize=25,
ha='center', va='center')
plt.annotate('\u2192', xy=(0, 0), xytext=(max(pop)-180, 1825000), fontsize=25,
ha='center', va='center')
plt.ylim(0,1900000)
plt.xlim(min(pop), max(pop))
print(f"The mean rotation of simulated molecules in 48 h:{np.mean(pop): .3g}°.")
plt.show()
```

12) References

1. Hu, L. *et al.* CuH-Catalyzed Atropoenantioselective Reduction of Bringmann's Lactones via Dynamic Kinetic Resolution. *Org. Lett.* **21**, 5575–5580 (2019).
2. Bringmann, G., Menche, D. Stereoselective Total Synthesis of Axially Chiral Natural Products via Biaryls Lactones. *Acc. Chem. Res.* **34**, 615–624 (2001).
3. Bringmann, G., Breuning, M., Henshel, P., Hinrichs, J. Asymmetric Synthesis of (*M*)-2-Hydroxymethyl-1-(2-hydroxy-4,6-dimethylphenyl)naphthalene via a Configurationally Unstable Biaryl Lactone. *Org. Synth.* **79**, 72–78 (2002).
4. Frisch, M. J., *et al*. *Gaussian 16 Rev. C.01*, (Wallingford, CT, 2016).
5. Wu, Y., Li, M., Sun, J., Zheng, G. & Zhang, Q. Synthesis of Axially Chiral Aldehydes by N-Heterocyclic-Carbene-Catalyzed Desymmetrization Followed by Kinetic Resolution. *Angew. Chem. Int. Ed.* **61**, e202117340 (2022).
6. Miura, M. *et al.* Potent and selective TF/FVIIa inhibitors containing a neutral P1 ligand. *Bioorg. Med. Chem.* **14**, 7688–7705 (2006).
7. Staniland, S. *et al.* Enzymatic Desymmetrising Redox Reactions for the Asymmetric Synthesis of Biaryl Atropisomers. *Chem. Eur. J.* **20**, 13084–13088 (2014).
8. Ramachandran, P. V., Alawaed, A. A. & Hamann, H. J. $TiCl_4$-Catalyzed Hydroboration of Ketones with Ammonia Borane. *J. Org. Chem.* **87**, 13259–13269 (2022).
9. Morokutti, A. *et al.* Structure and Function of YcnD from *Bacillus subtilis*, a Flavin-Containing Oxidoreductase. *Biochemistry* **44**, 13724–13733 (2005).
10. Heeb, J.-P., Clayden, J., Smith, M. D. & Armstrong, R. J. Interrogating the configurational stability of atropisomers. *Nat. Protoc.* **18**, 2745–2771 (2023).
11. Lu, J., Ma, D., Hu, J., Tang, W. & Zhu, D. Nuclear magnetic resonance spectroscopic studies of pyridine methyl derivatives binding to cytochrome c. *J. Chem. Soc., Dalton Trans.* 2267–2274 (1998).

12. Aguilera-Sáez, L. M., *et al*. Pushing the frontiers: boron-11 NMR as a method for quantitative boron analysis and its application to determine boric acid in commercial biocides. *Analyst* **143**, 4707–4714 (2018).

13. Florescu, I. *Probability and Stochastic Processes*. (Newark, John Wiley & Sons, Incorporated, 2014)

14. Van Rossum, G., & Drake, F. L.. *Python 3 Reference Manual*. (Scotts Valley, CA, CreateSpace, 2009).

15. Kluyver, T., *et al*. *Jupyter Notebooks - a publishing format for reproducible computational workflows*. (Amsterdam, IOS Press, 2016).

16. Harris, C. R. *et al.* Array programming with NumPy. *Nature* **585**, 357–362 (2020).

17. Hunter, J. D. Matplotlib: A 2D Graphics Environment. *Comput. Sci. Eng.* **9**, 90–95 (2007).

13) NMR spectra

1-Fluoro-10-methyl-6H-benzo[c]chromen-6-one (S1a) (^{1}H NMR, 400 MHz, $CDCl_3$)

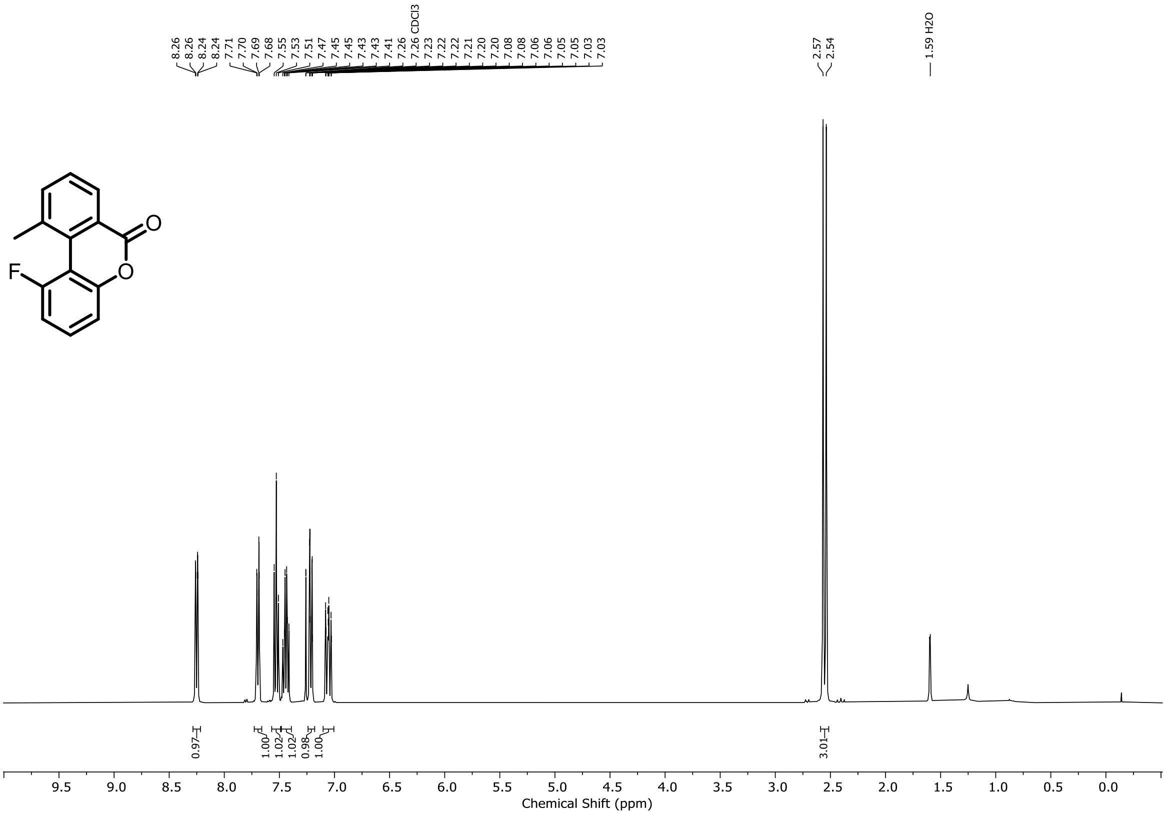

1-Fluoro-10-methyl-6H-benzo[c]chromen-6-one (S1a) (^{13}C NMR, 101 MHz, $CDCl_3$)

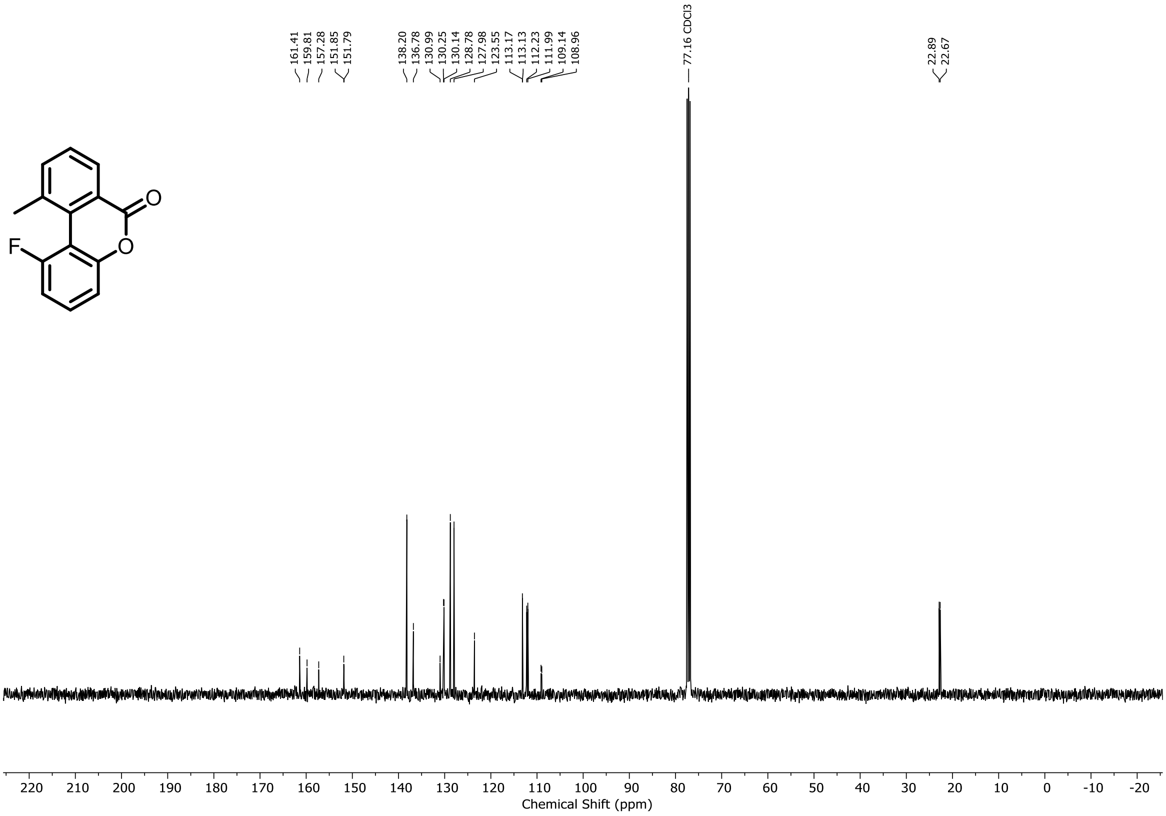

1-Fluoro-10-methyl-6H-benzo[c]chromen-6-one (S1a) (^{19}F NMR, 376 MHz, $CDCl_3$)

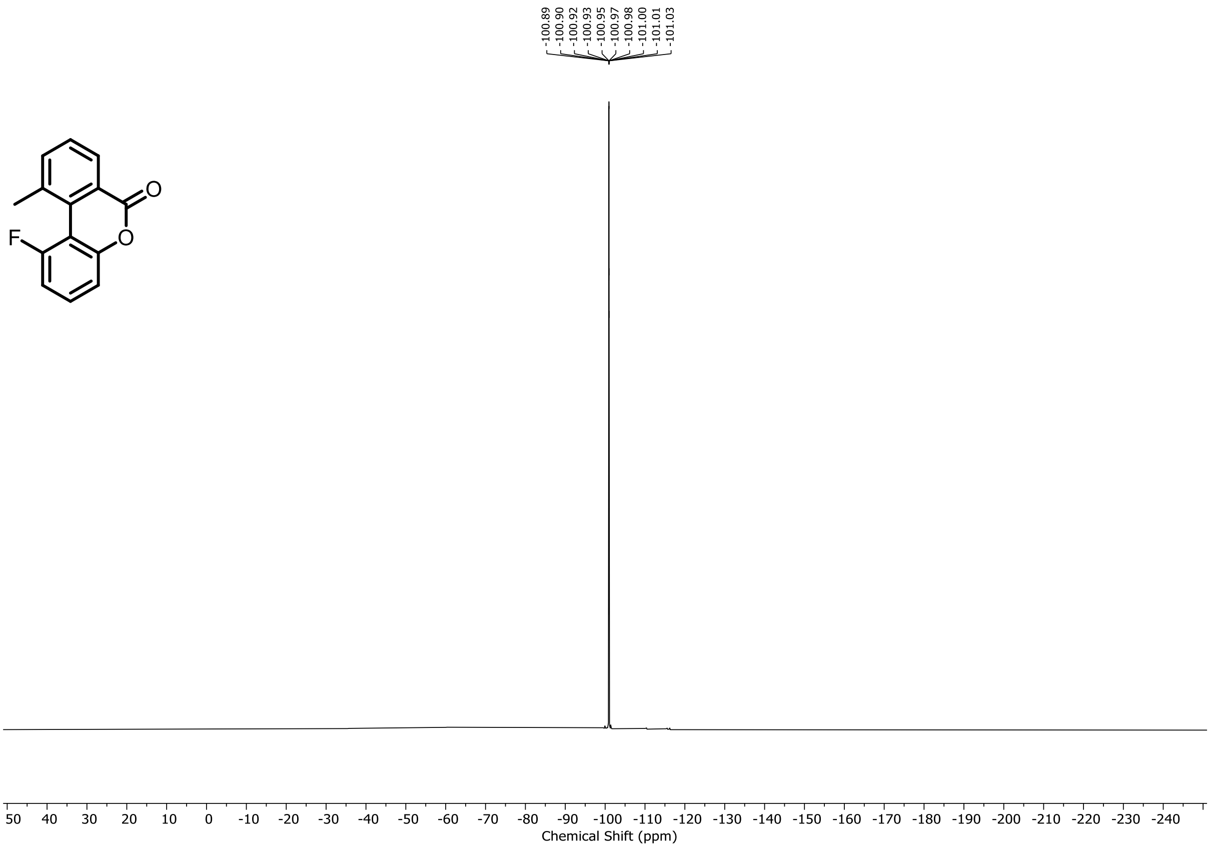

6-Fluoro-2'-(hydroxymethyl)-6'-methyl-[1,1'-biphenyl]-2-ol (1a) (^{1}H NMR, 400 MHz, CD_3OD)

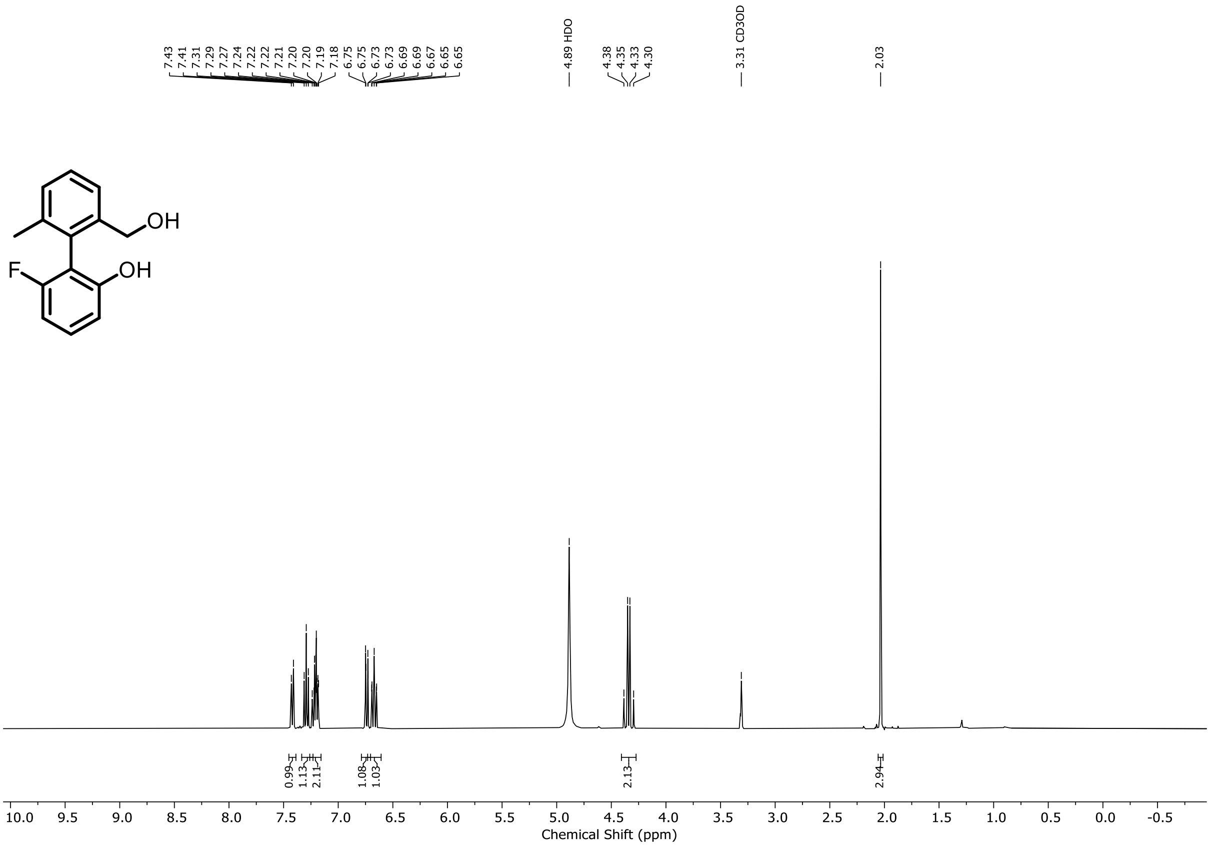

6-Fluoro-2'-(hydroxymethyl)-6'-methyl-[1,1'-biphenyl]-2-ol (1a) (^{13}C NMR, 101 MHz, CD_3OD)

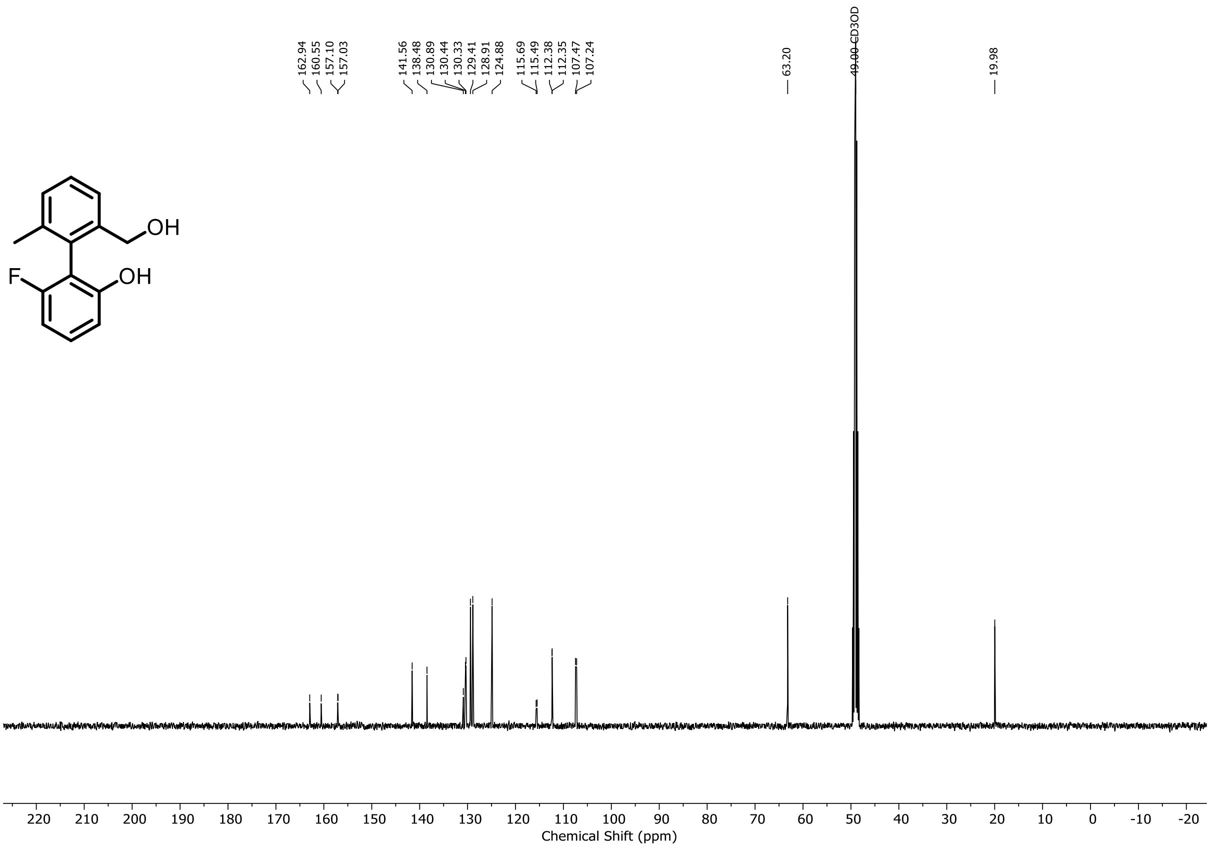

6-Fluoro-2'-(hydroxymethyl)-6'-methyl-[1,1'-biphenyl]-2-ol (1a) (^{19}F NMR, 376 MHz, CD_3OD)

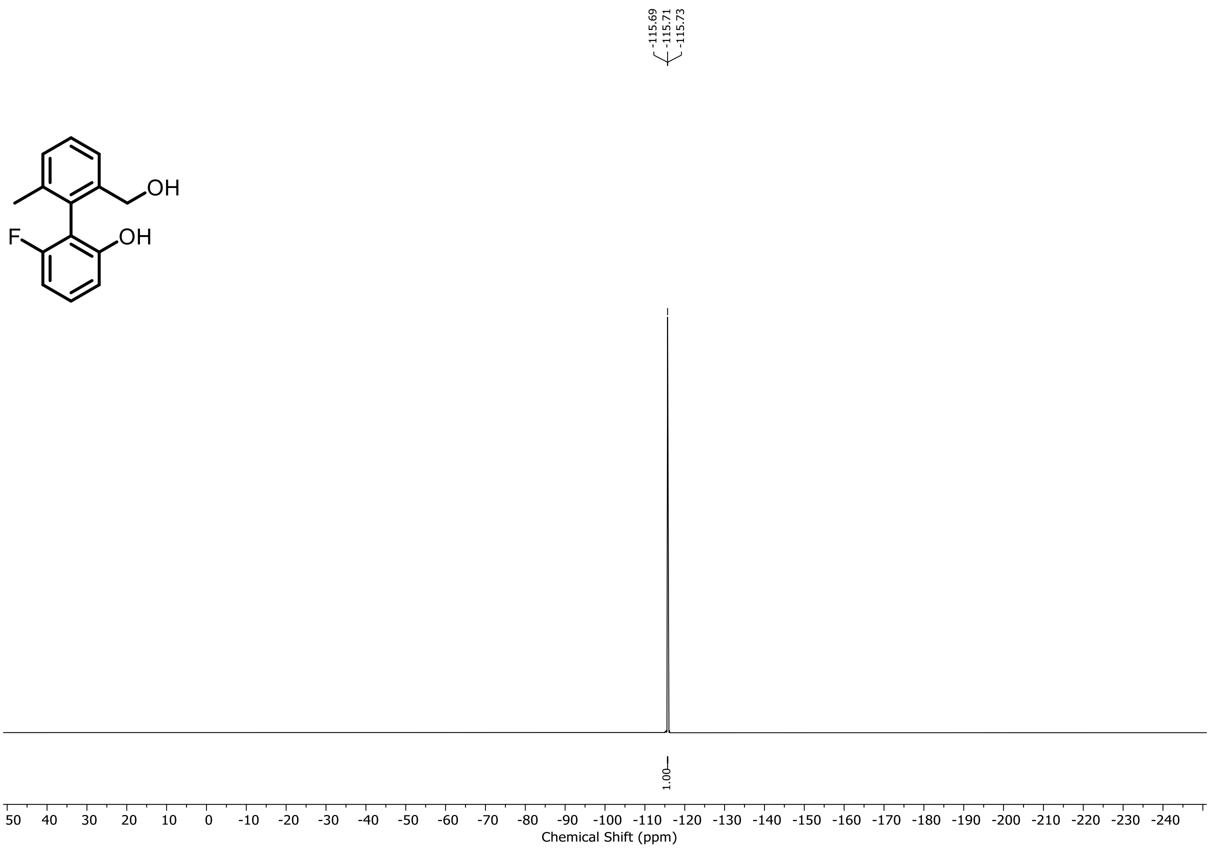

2'-Fluoro-6'-hydroxy-6-methyl-[1,1'-biphenyl]-2-carbaldehyde (2a) (^{1}H NMR, 400 MHz, CD_3OD)

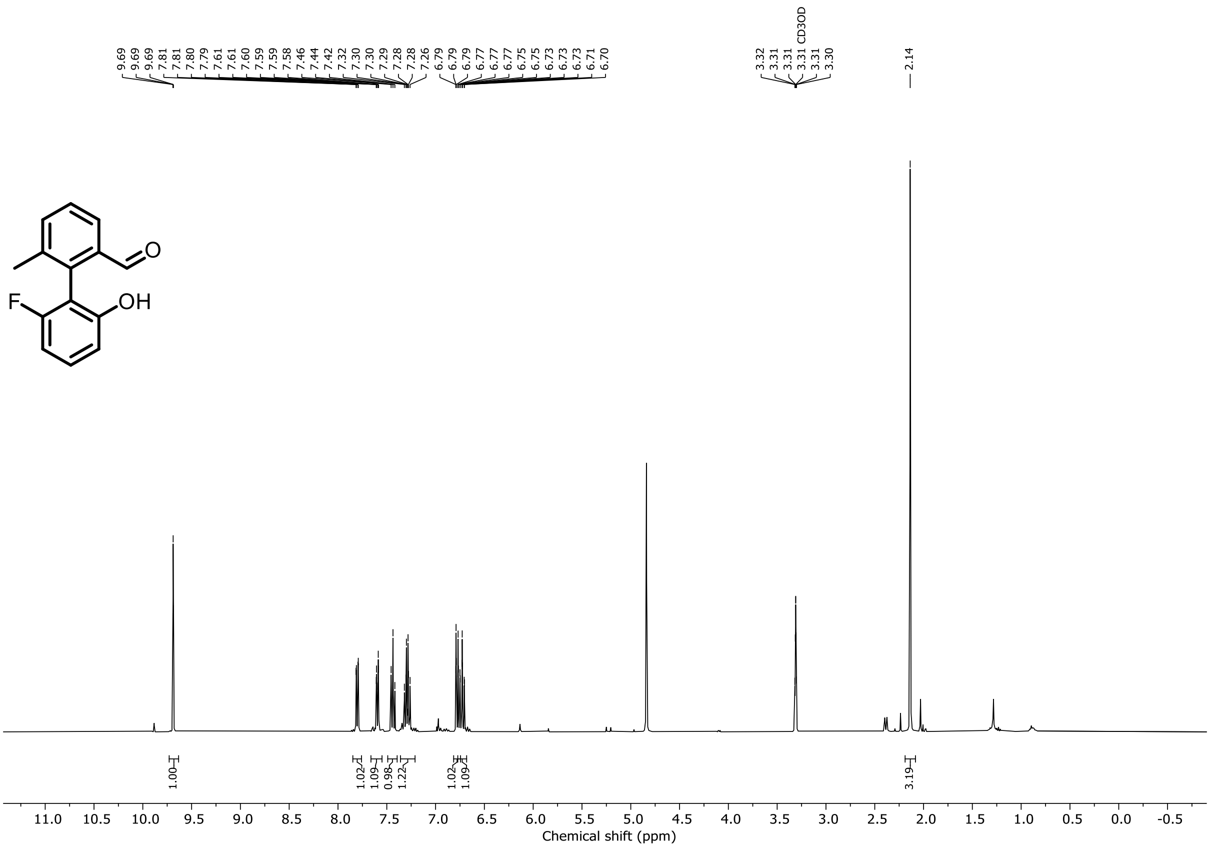

2'-Fluoro-6'-hydroxy-6-methyl-[1,1'-biphenyl]-2-carbaldehyde (2a) (^{13}C NMR, 101 MHz, CD_3OD)

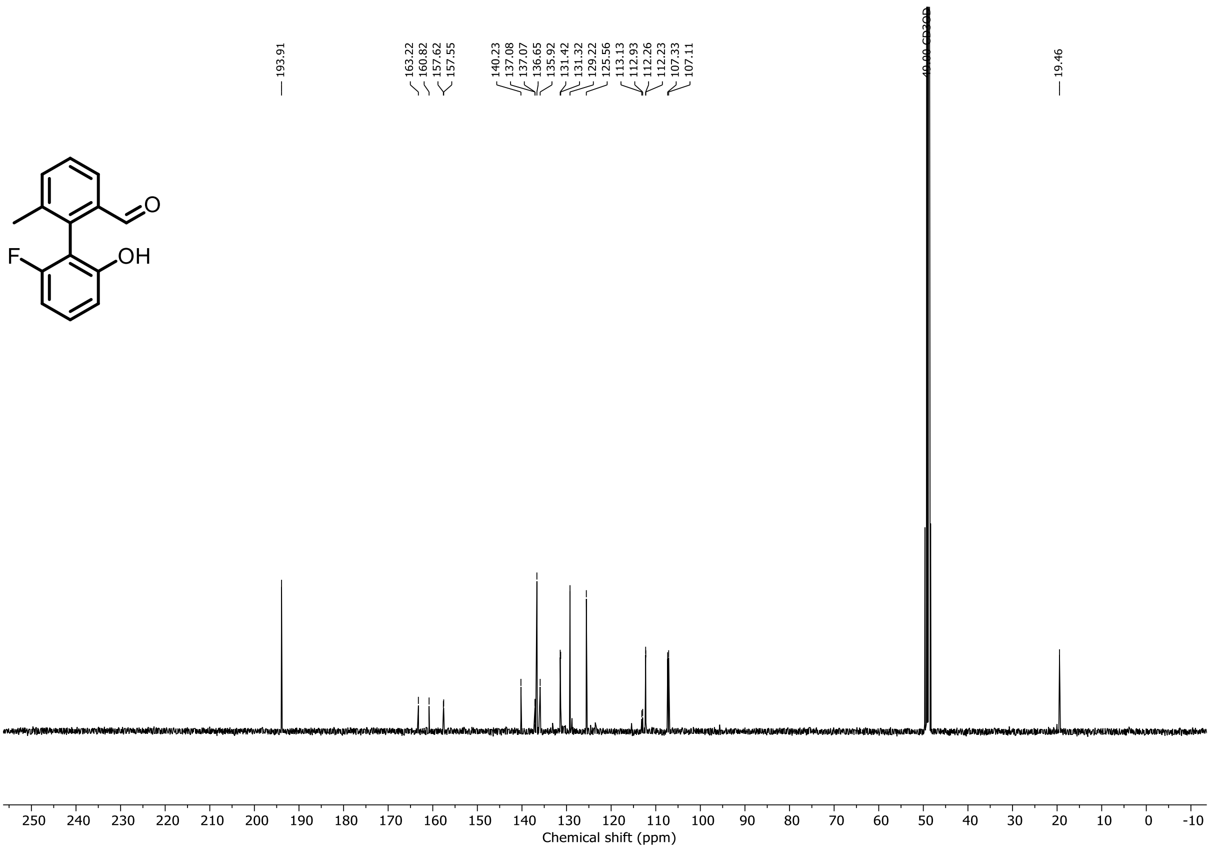

2'-Fluoro-6'-hydroxy-6-methyl-[1,1'-biphenyl]-2-carbaldehyde (2a) (^{19}F NMR, 377 MHz, CD_3OD)

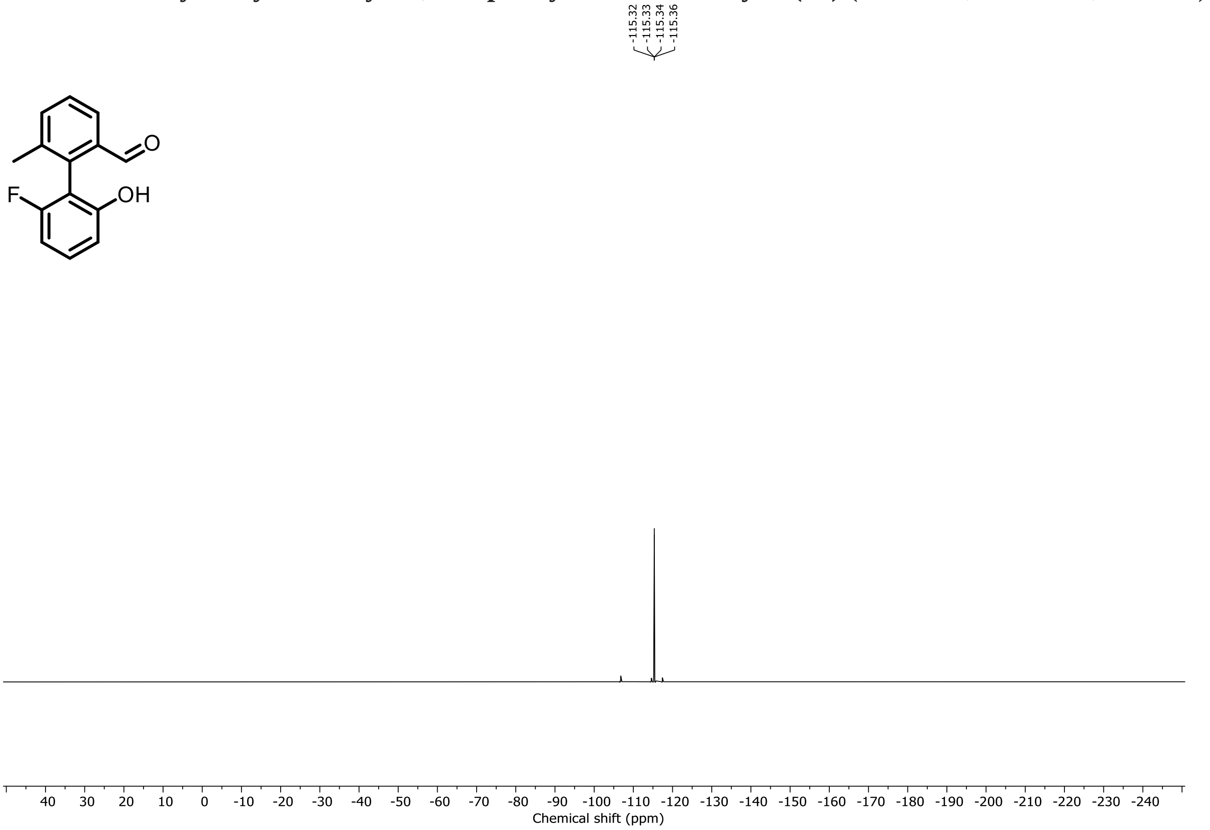

2'-Fluoro-6'-hydroxy-6-methyl-[1,1'-biphenyl]-2-carboxylic acid (S1b) (^{1}H NMR, 600 MHz, CD_3OD)

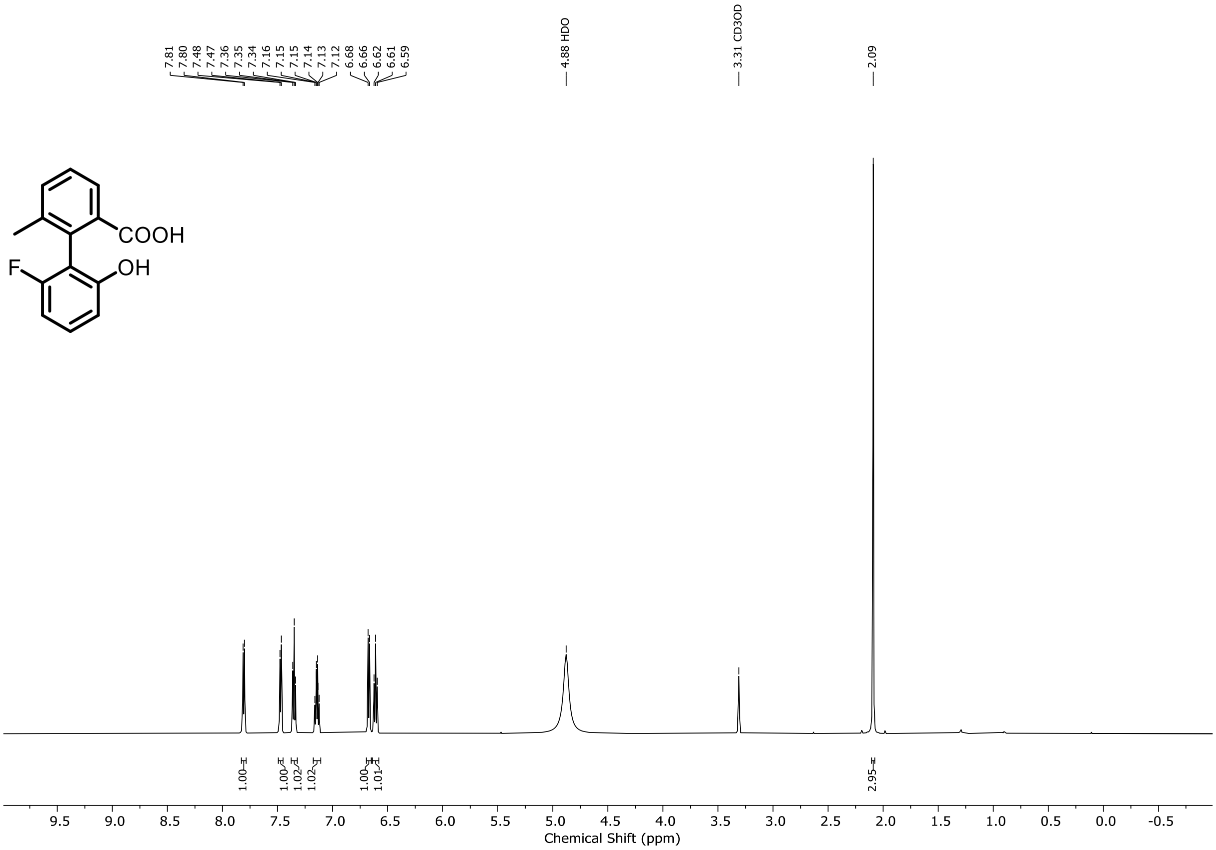

2'-Fluoro-6'-hydroxy-6-methyl-[1,1'-biphenyl]-2-carboxylic acid (S1b) (^{13}C NMR, 151 MHz, CD_3OD)

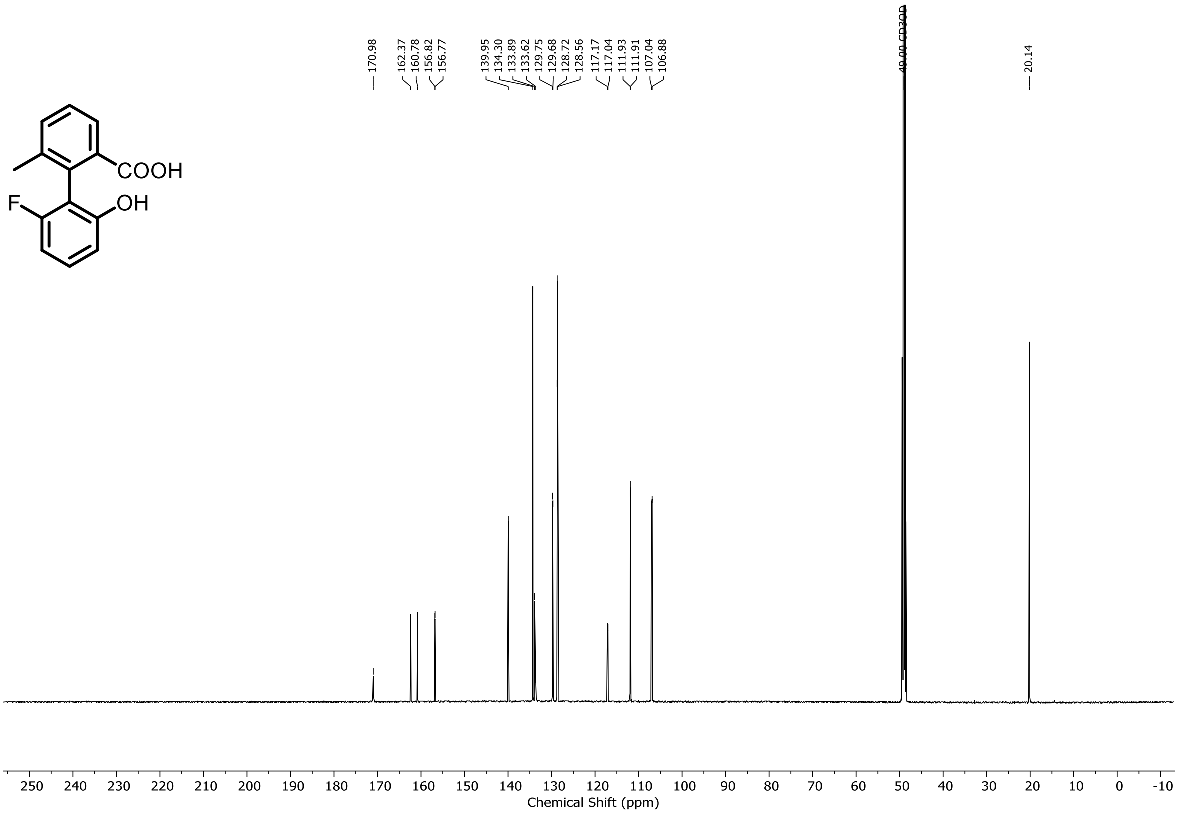

2'-Fluoro-6'-hydroxy-6-methyl-[1,1'-biphenyl]-2-carboxylic acid (S1b) (^{19}F NMR, 565 MHz, CD_3OD)

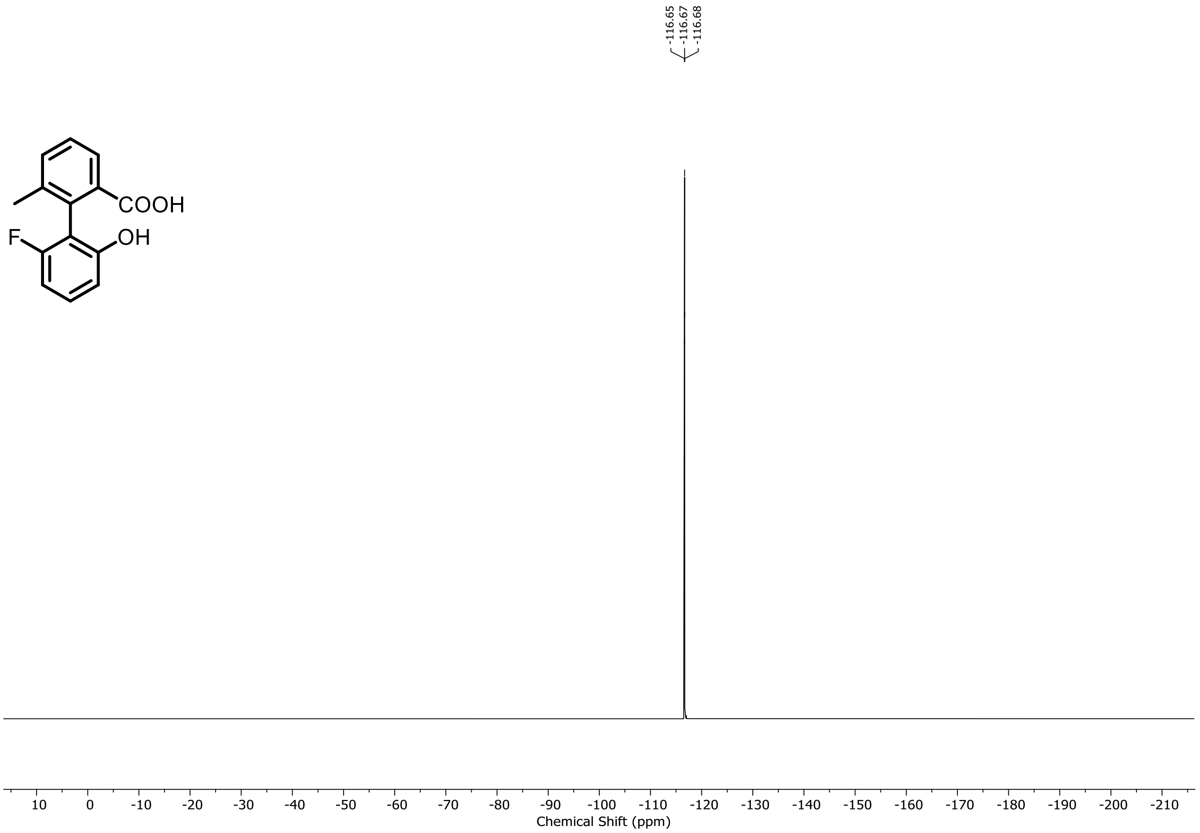

2'-Fluoro-6'-methoxy-[1,1'-biphenyl]-2,6-dicarbaldehyde (S3a) (^{1}H NMR, 400 MHz, $CDCl_3$)

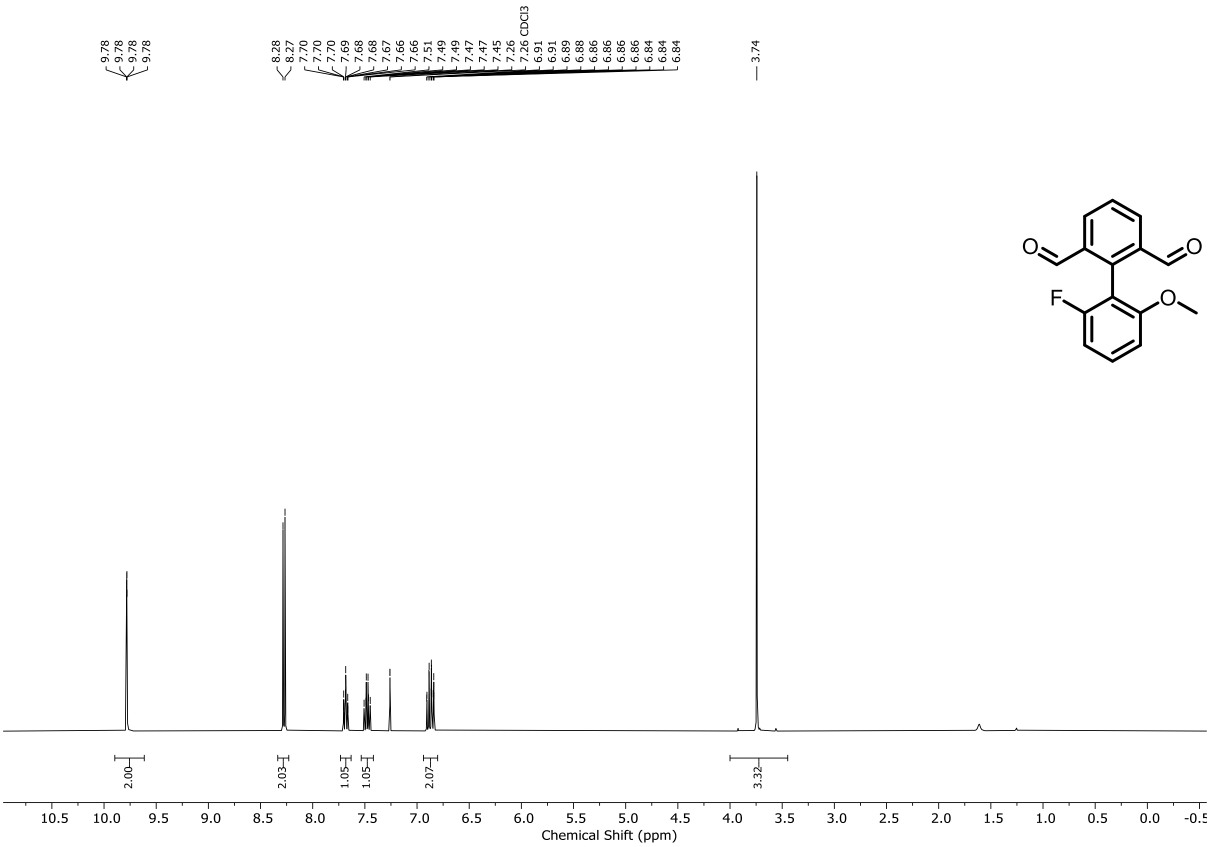

2'-Fluoro-6'-methoxy-[1,1'-biphenyl]-2,6-dicarbaldehyde (S3a) (^{13}C NMR, 126 MHz, $CDCl_3$)

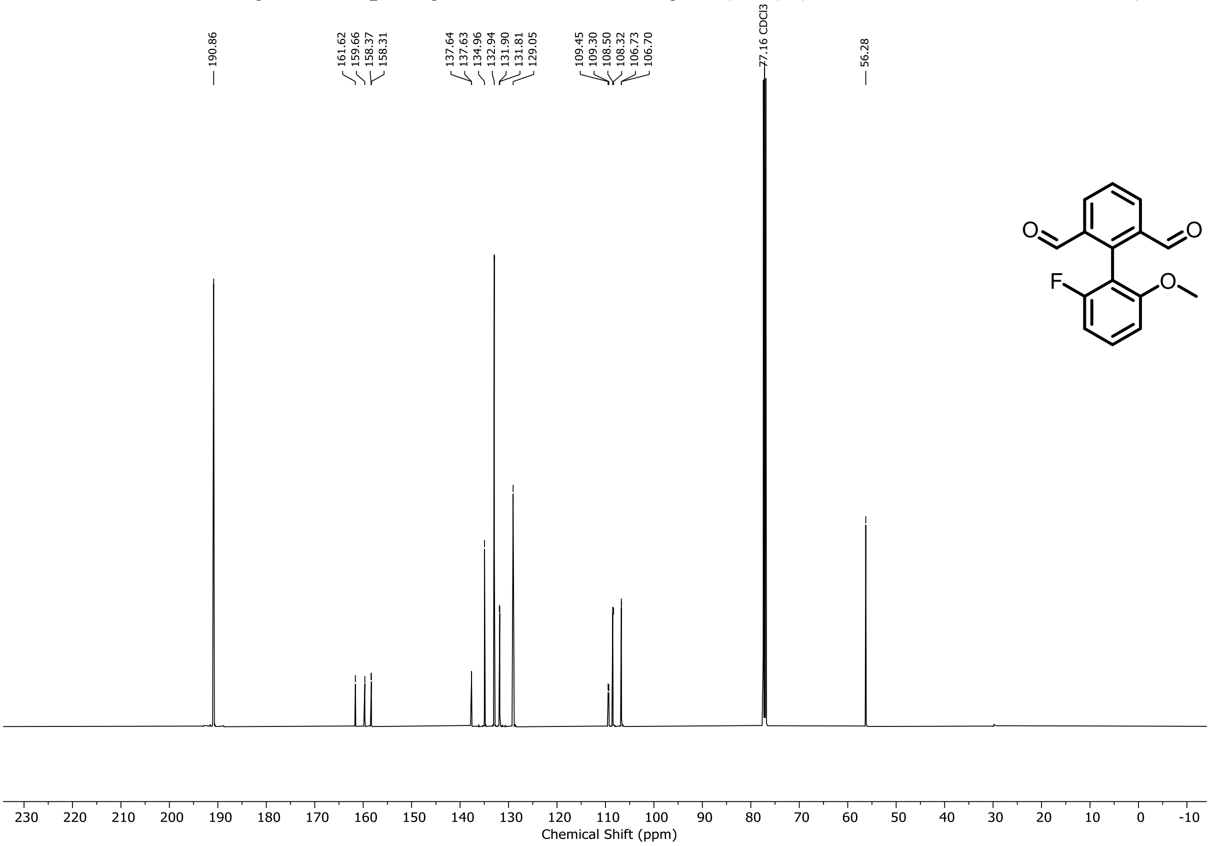

2'-Fluoro-6'-methoxy-[1,1'-biphenyl]-2,6-dicarbaldehyde (S3a) (^{19}F NMR, 377 MHz, $CDCl_3$)

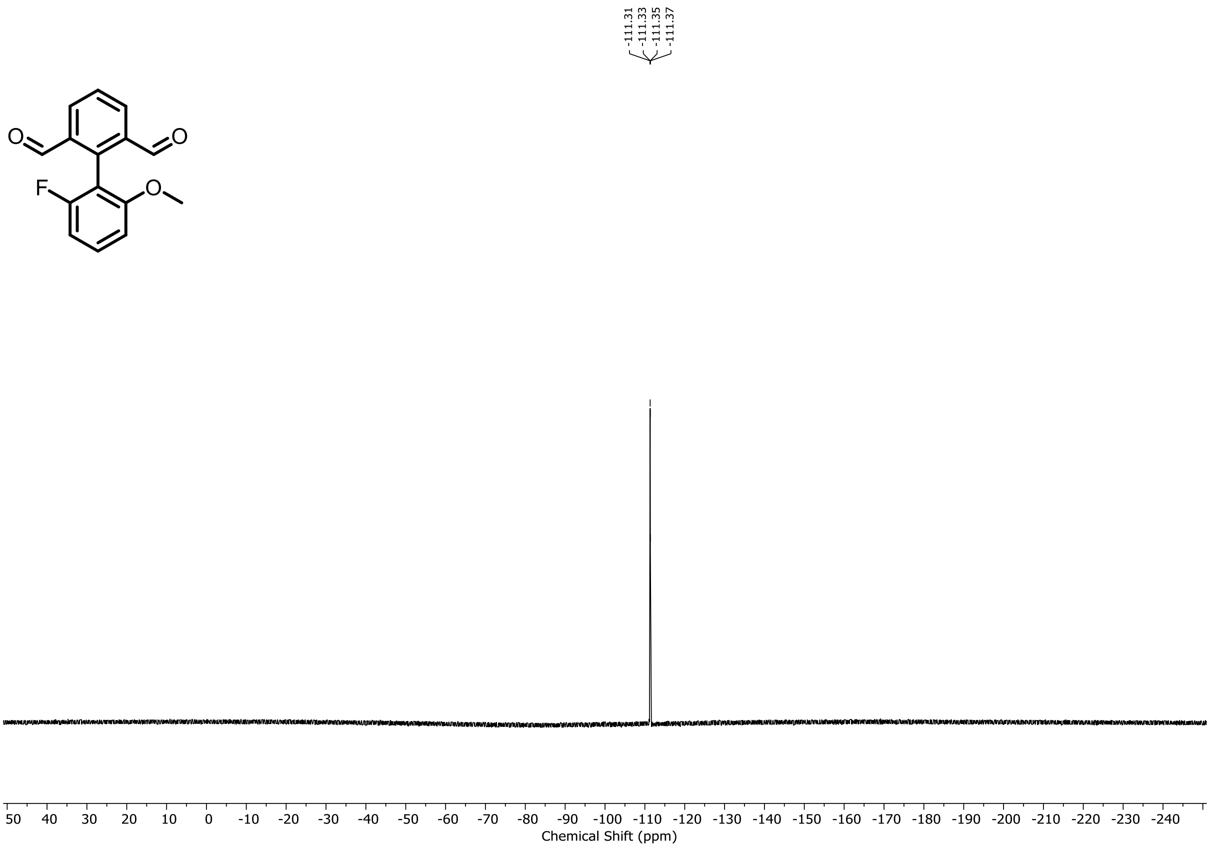

2'-Fluoro-6'-methoxy-[1,1'-biphenyl]-2,6-dicarboxylic acid (S3b) (^{1}H NMR, 400 MHz, $(CD_3)_2CO$)

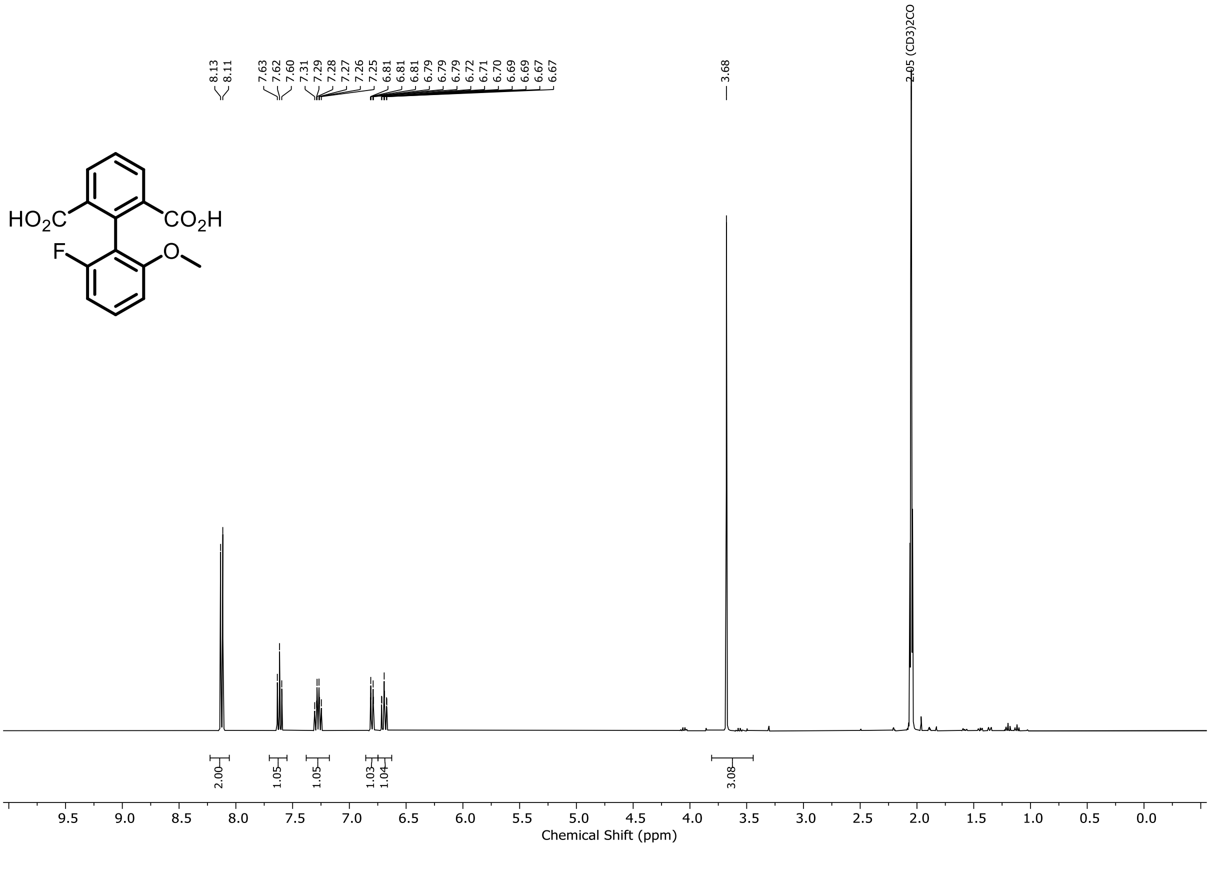

2'-Fluoro-6'-methoxy-[1,1'-biphenyl]-2,6-dicarboxylic acid (S3b) (^{13}C NMR, 126 MHz, $(CD_3)_2CO$)

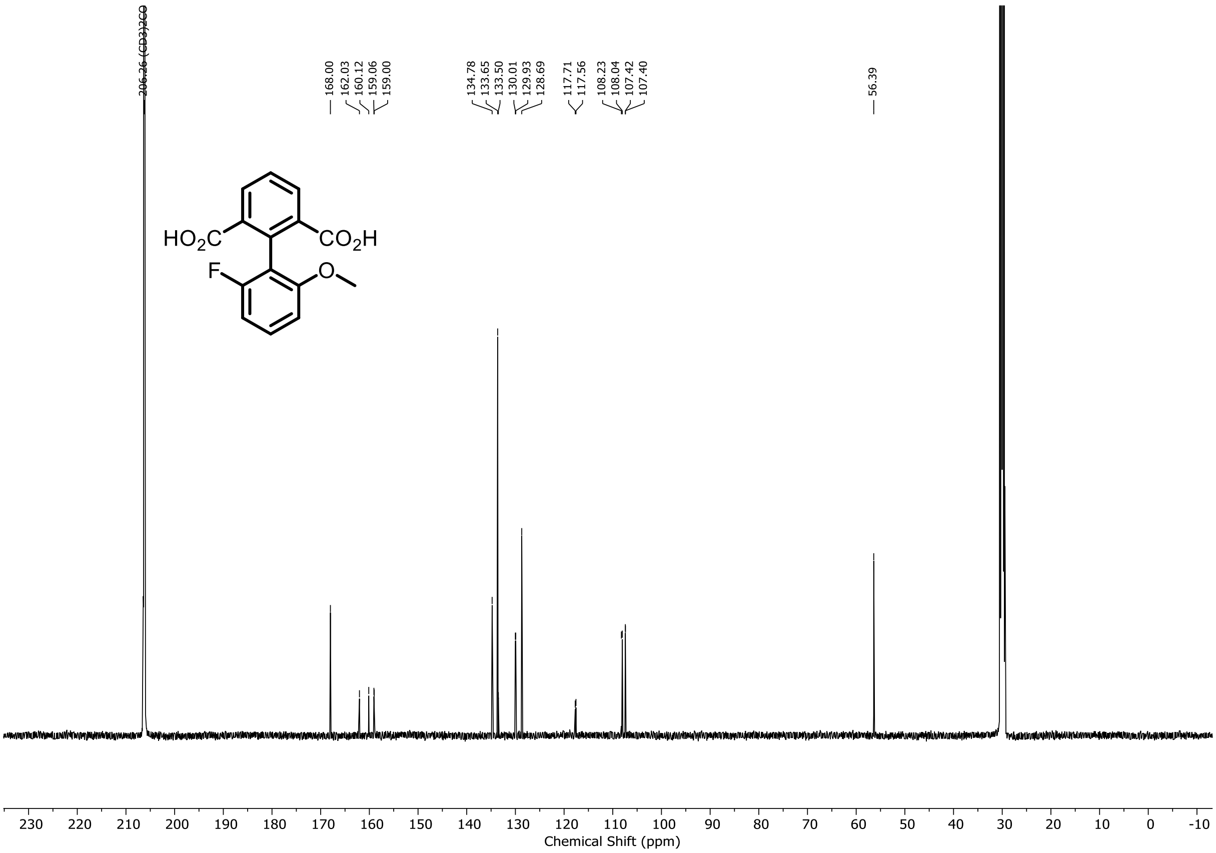

2'-Fluoro-6'-methoxy-[1,1'-biphenyl]-2,6-dicarboxylic acid (S3b) (^{19}F NMR, 377 MHz, $(CD_3)_2CO$)

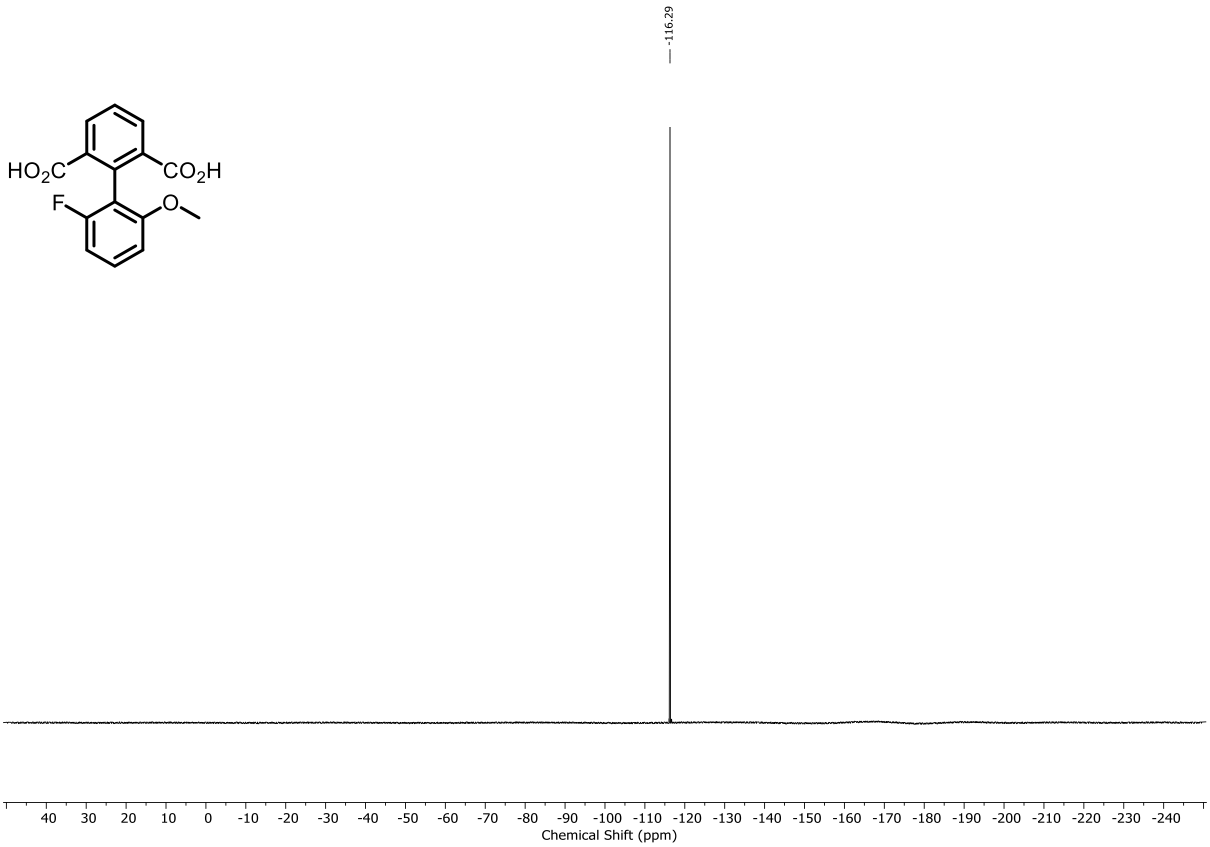

Dimethyl 2'-fluoro-6'-methoxy-[1,1'-biphenyl]-2,6-dicarboxylate (S3c) (^{1}H NMR, 500 MHz, $CDCl_3$)

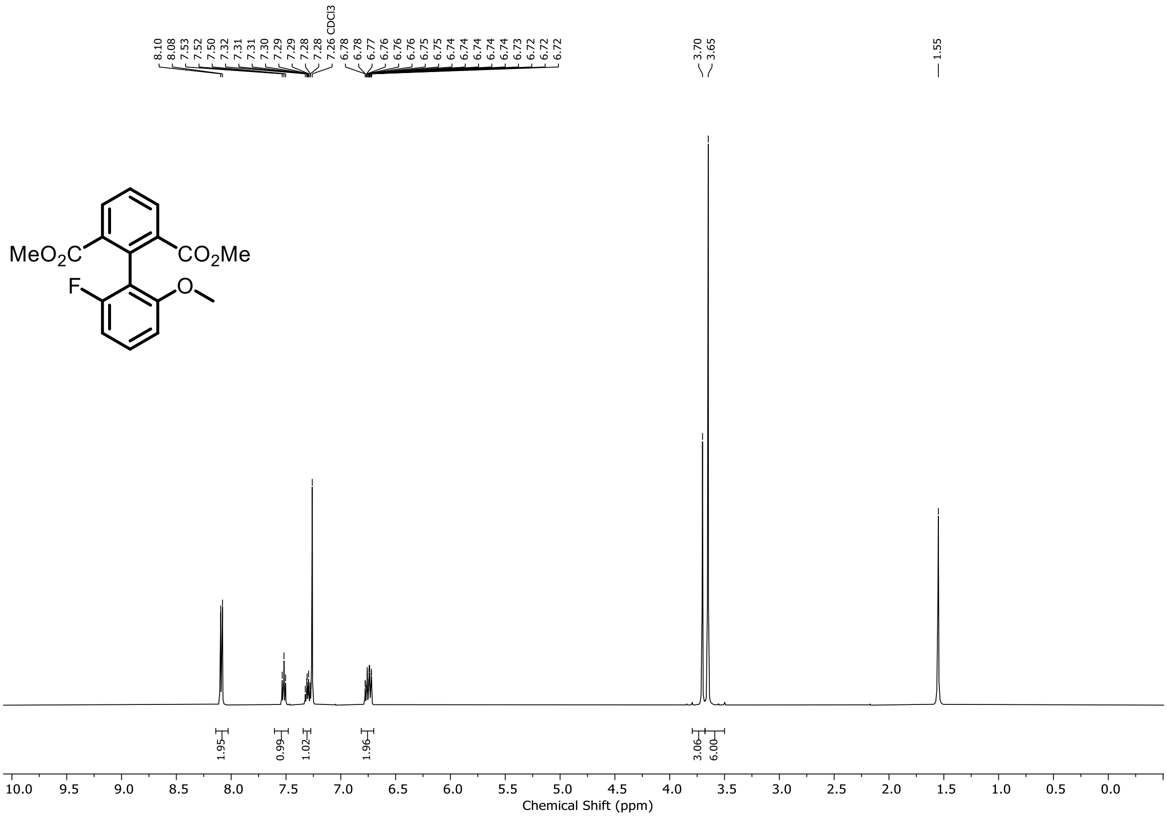

Dimethyl 2'-fluoro-6'-methoxy-[1,1'-biphenyl]-2,6-dicarboxylate (S3c) (^{13}C NMR, 126 MHz, $CDCl_3$)

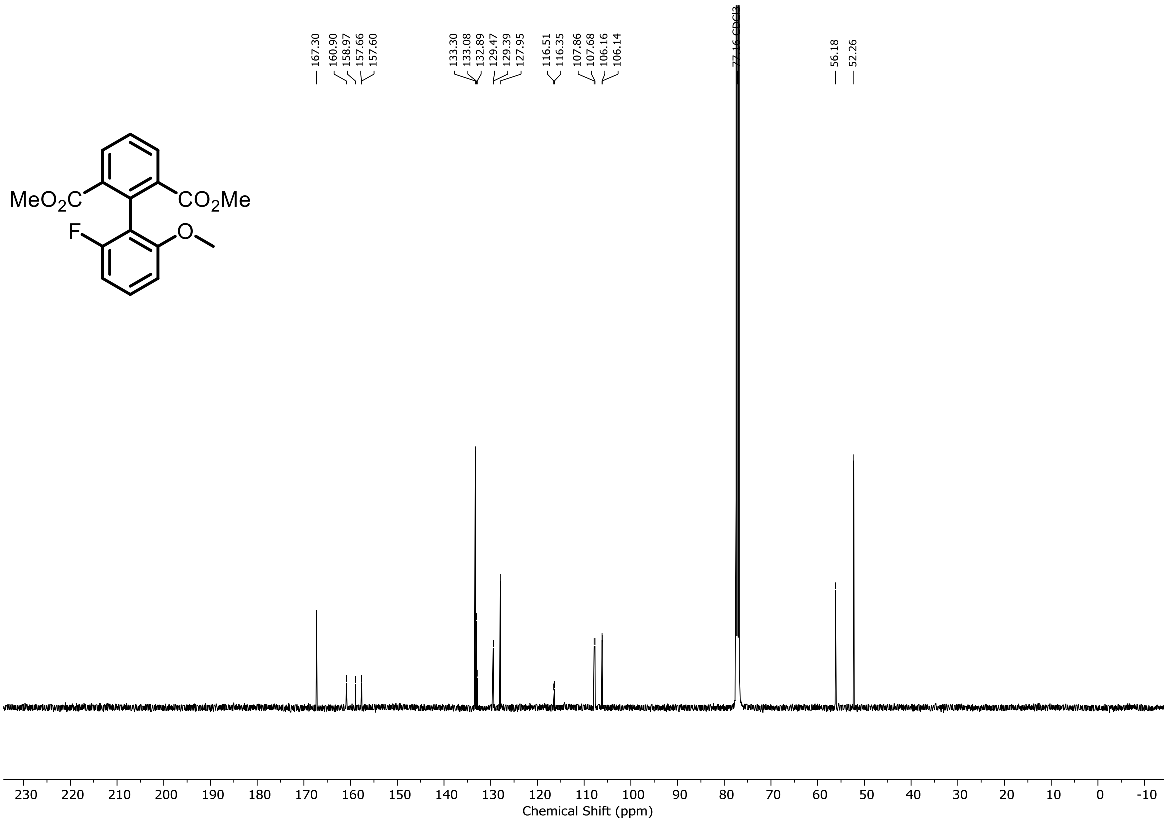

Dimethyl 2'-fluoro-6'-methoxy-[1,1'-biphenyl]-2,6-dicarboxylate (S3c) (^{19}F NMR, 377 MHz, $CDCl_3$)

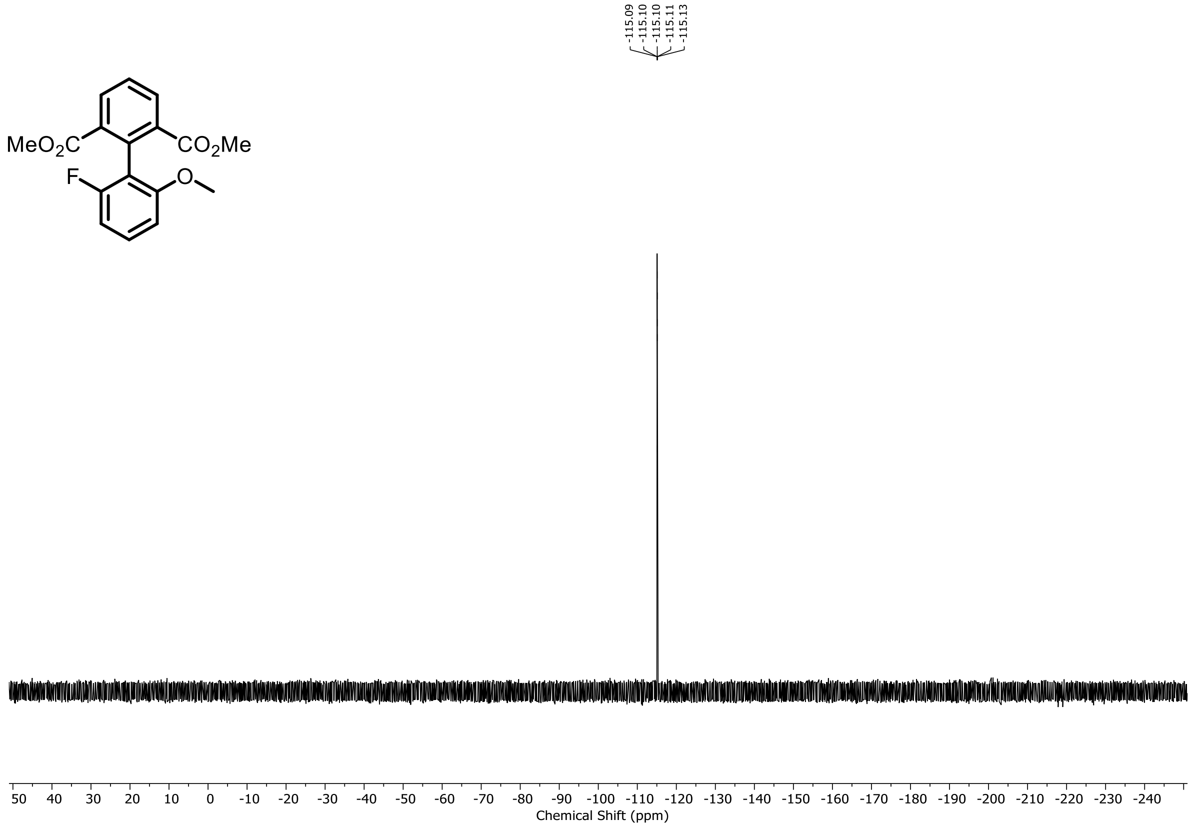

Methyl 1-fluoro-6-oxo-6*H*-benzo[*c*]chromene-10-carboxylate (S3d) (^{1}H NMR, 400 MHz, $CDCl_3$)

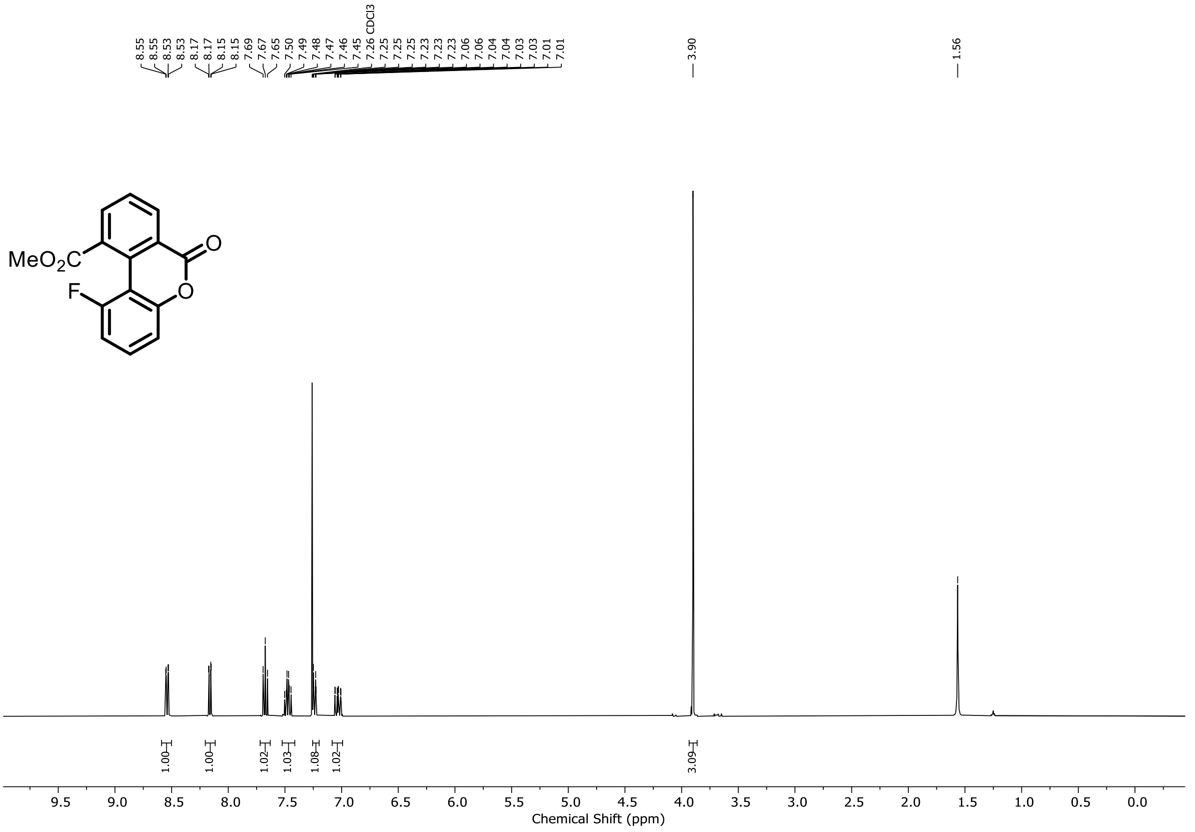

Methyl 1-fluoro-6-oxo-6*H*-benzo[*c*]chromene-10-carboxylate (S3d) (^{13}C NMR, 151 MHz, $(CD_3)_2CO$)

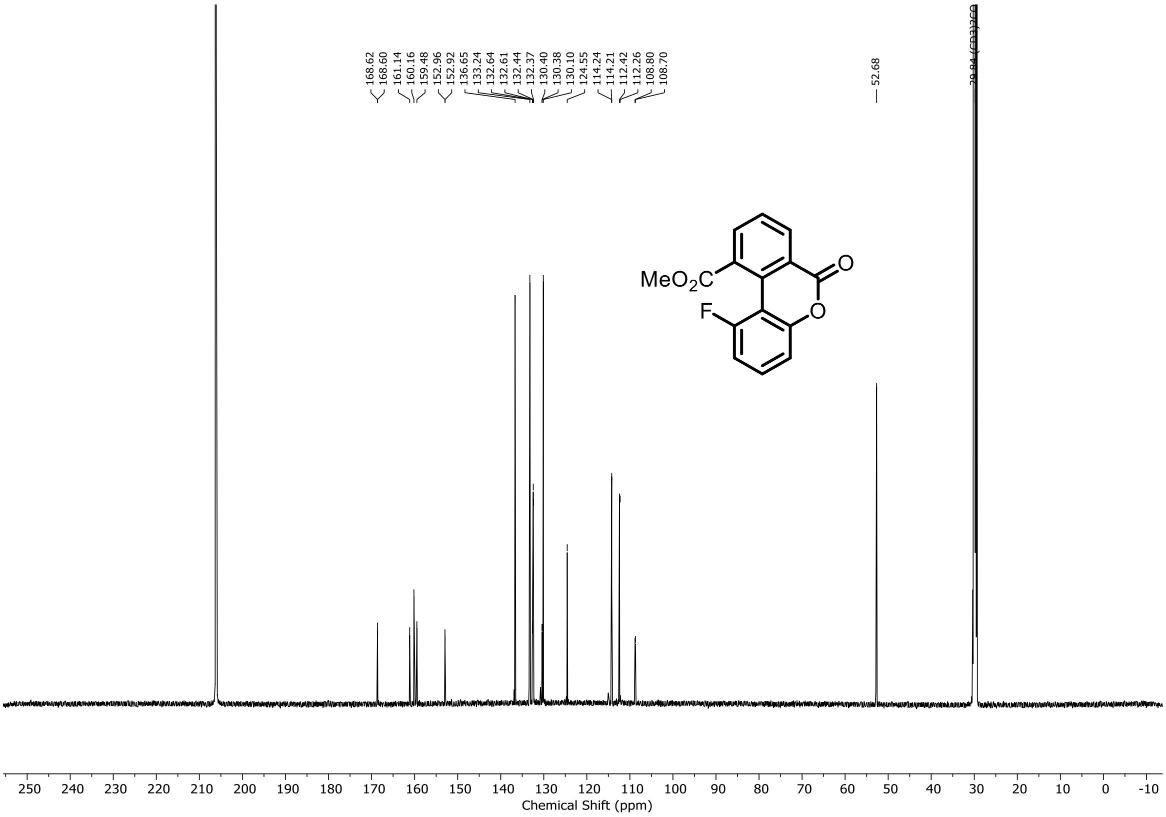

Methyl 1-fluoro-6-oxo-6*H*-benzo[*c*]chromene-10-carboxylate (S3d) (^{19}F NMR, 377 MHz, $(CD_3)_2CO$)

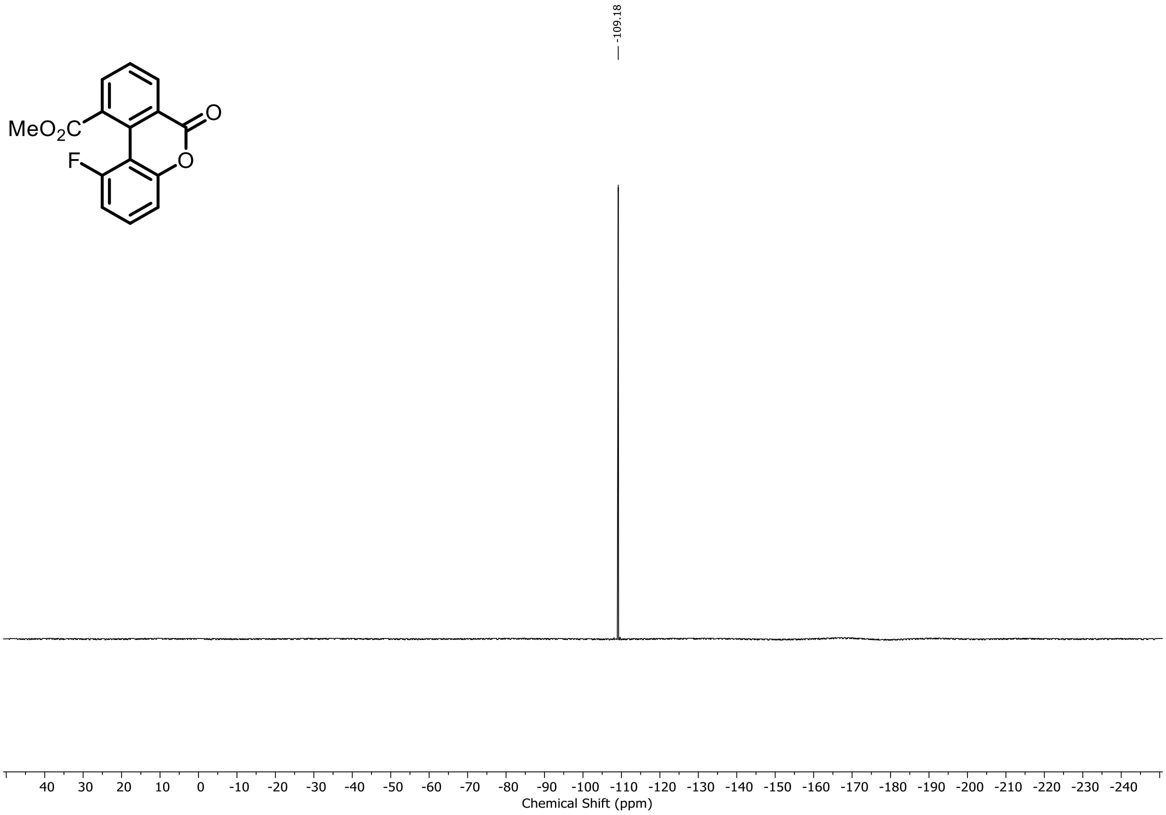

2'-Fluoro-6'-hydroxy-[1,1'-biphenyl]-2,6-diyl)dimethanol (3a) (^{1}H NMR, 600 MHz, $(CD_3)_2CO$)

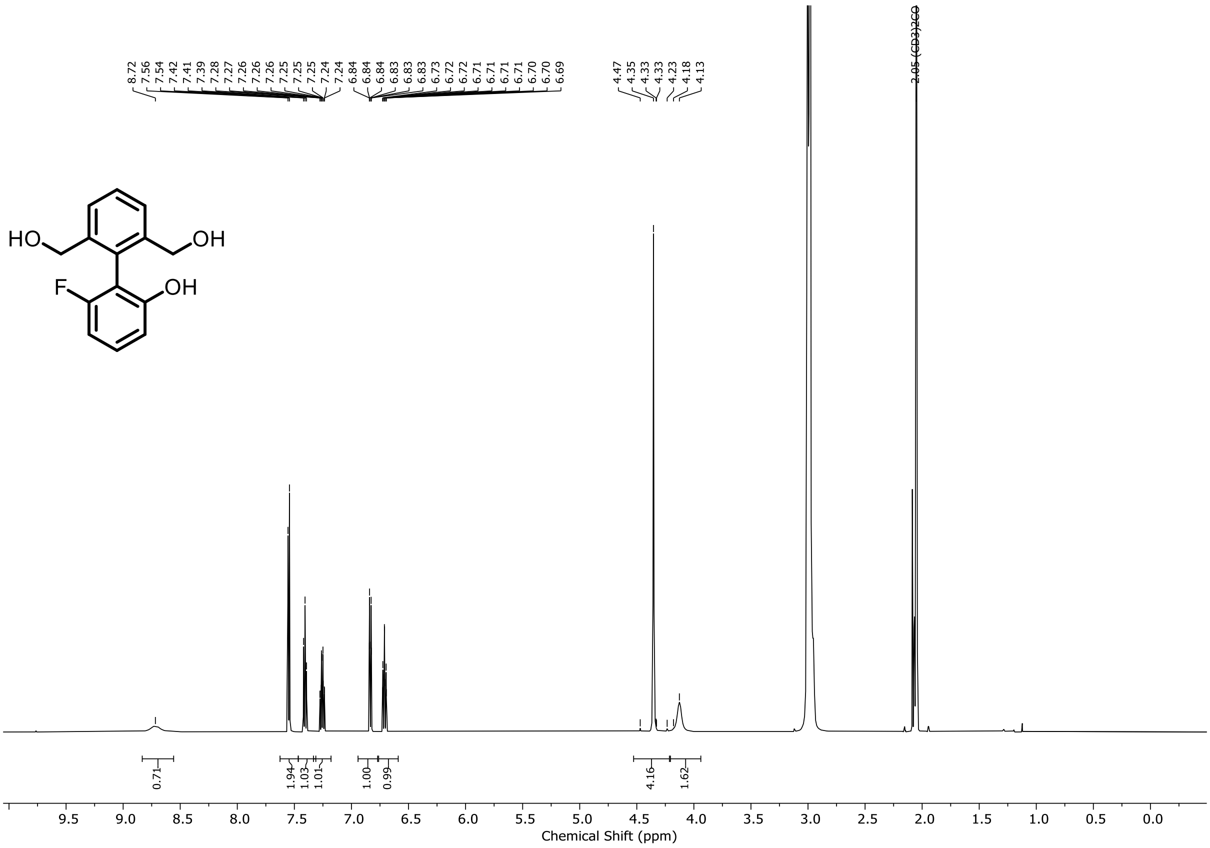

2'-Fluoro-6'-hydroxy-[1,1'-biphenyl]-2,6-diyl)dimethanol (3a) (^{13}C NMR, 151 MHz, $(CD_3)_2CO$)

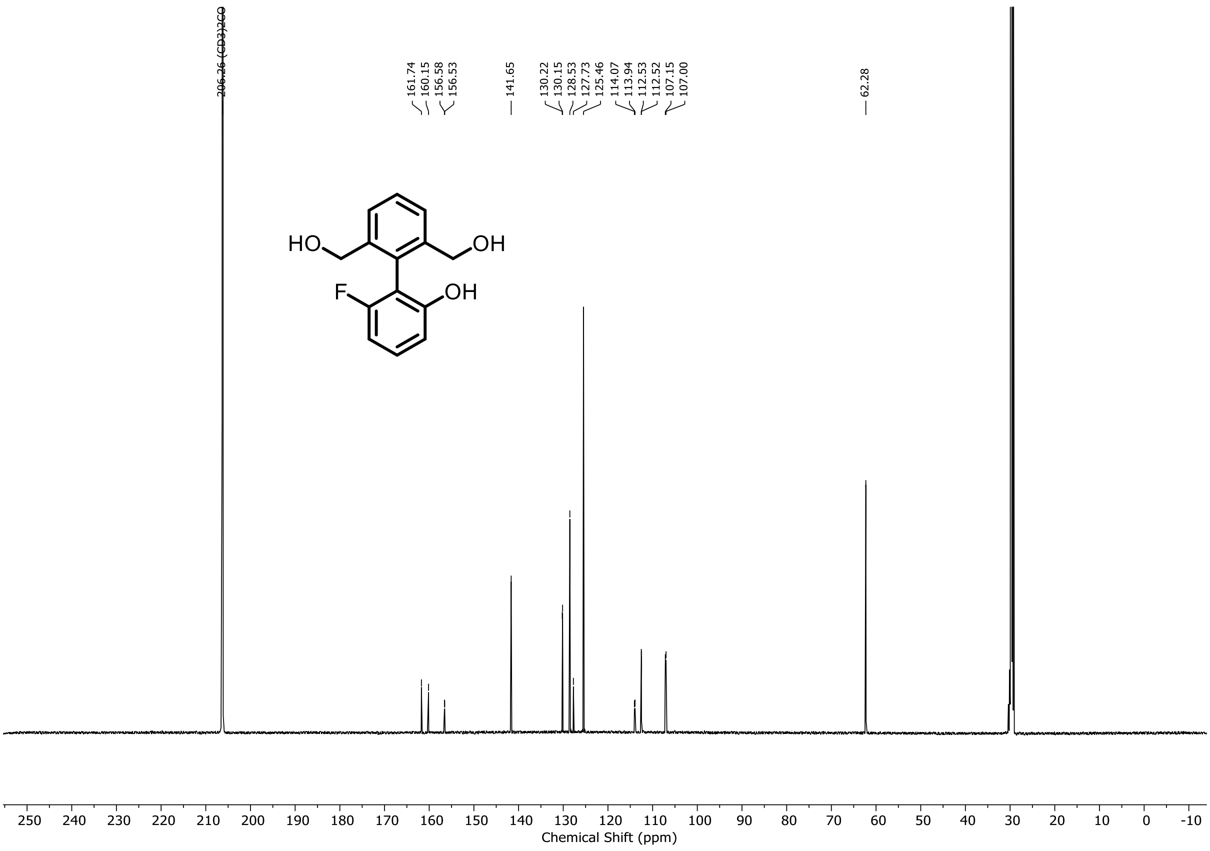

2'-Fluoro-6'-hydroxy-[1,1'-biphenyl]-2,6-diyl)dimethanol (3a) (^{19}F NMR, 376 MHz, $(CD_3)_2CO$)

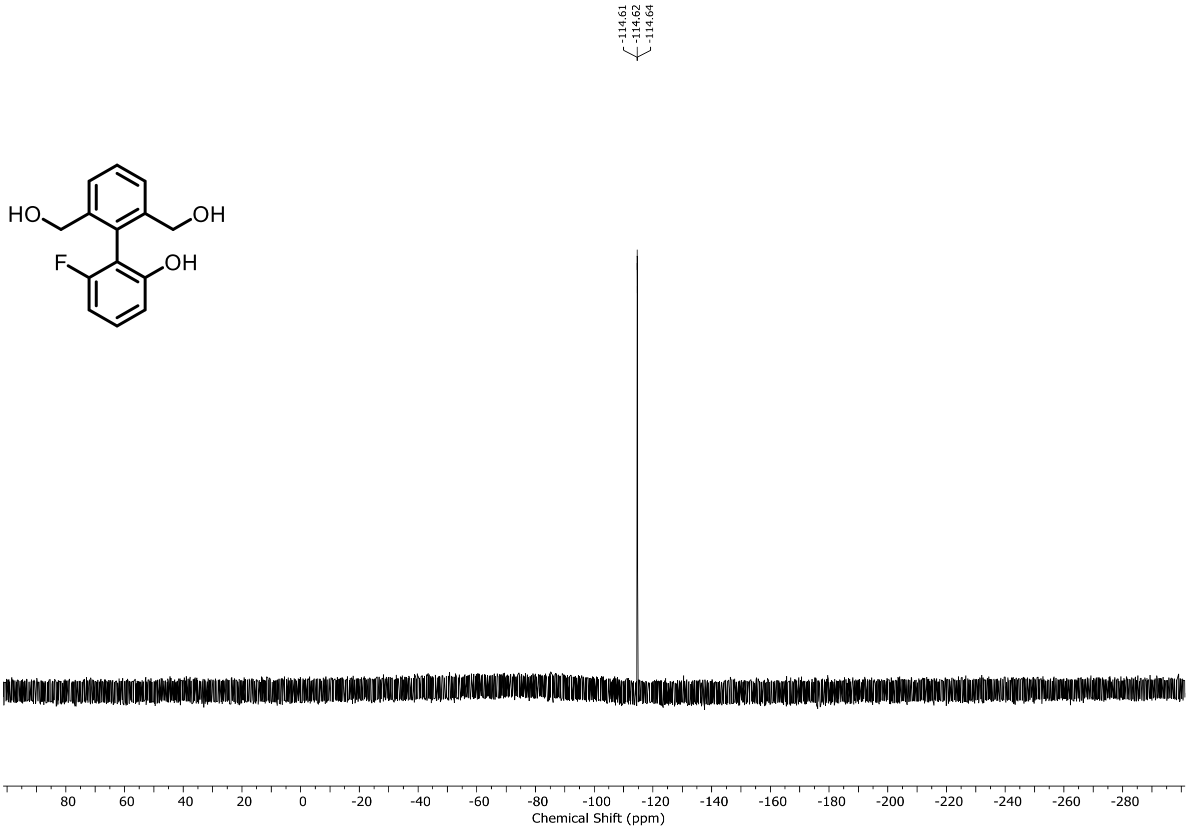

2'-Fluoro-6'-hydroxy-6-(hydroxymethyl)-[1,1'-biphenyl]-2-carbaldehyde (4a) (^{1}H NMR, 400 MHz, CD_3OD)

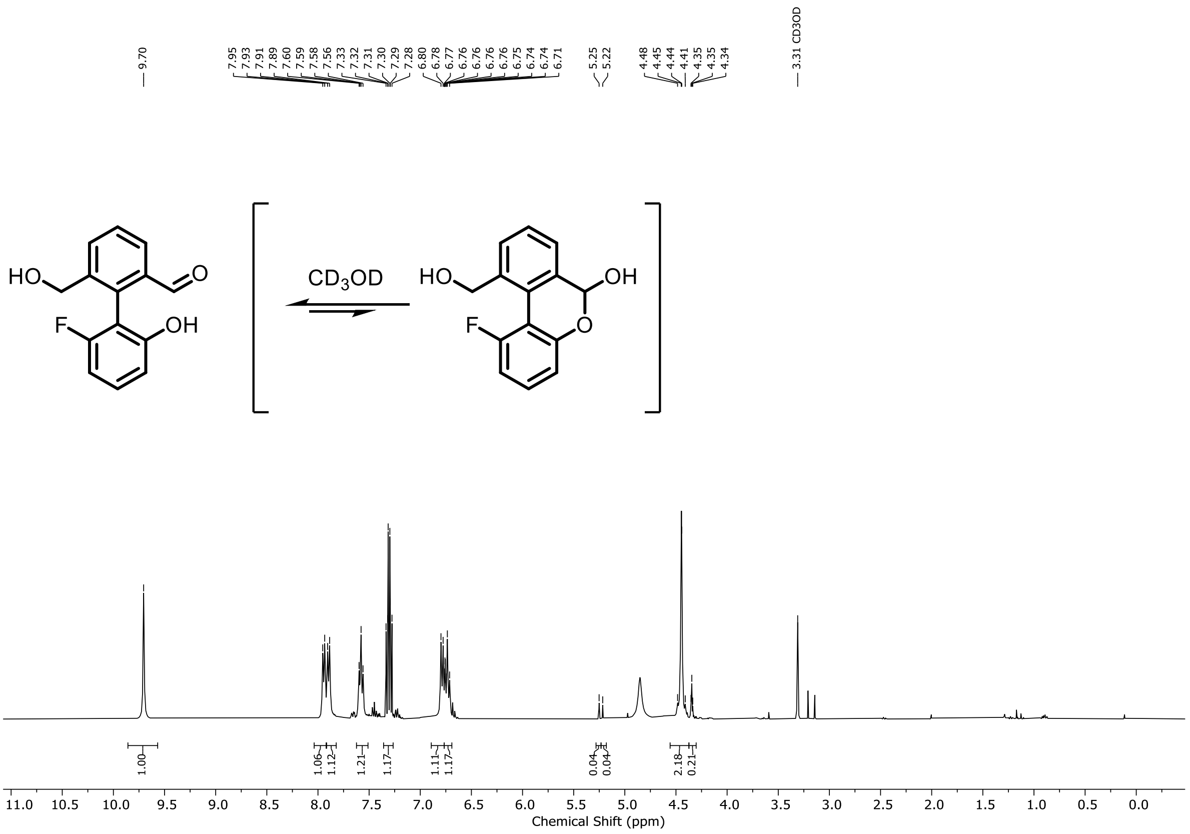

2'-Fluoro-6'-hydroxy-6-(hydroxymethyl)-[1,1'-biphenyl]-2-carbaldehyde (4a) (^{13}C NMR, 101 MHz, CD_3OD)

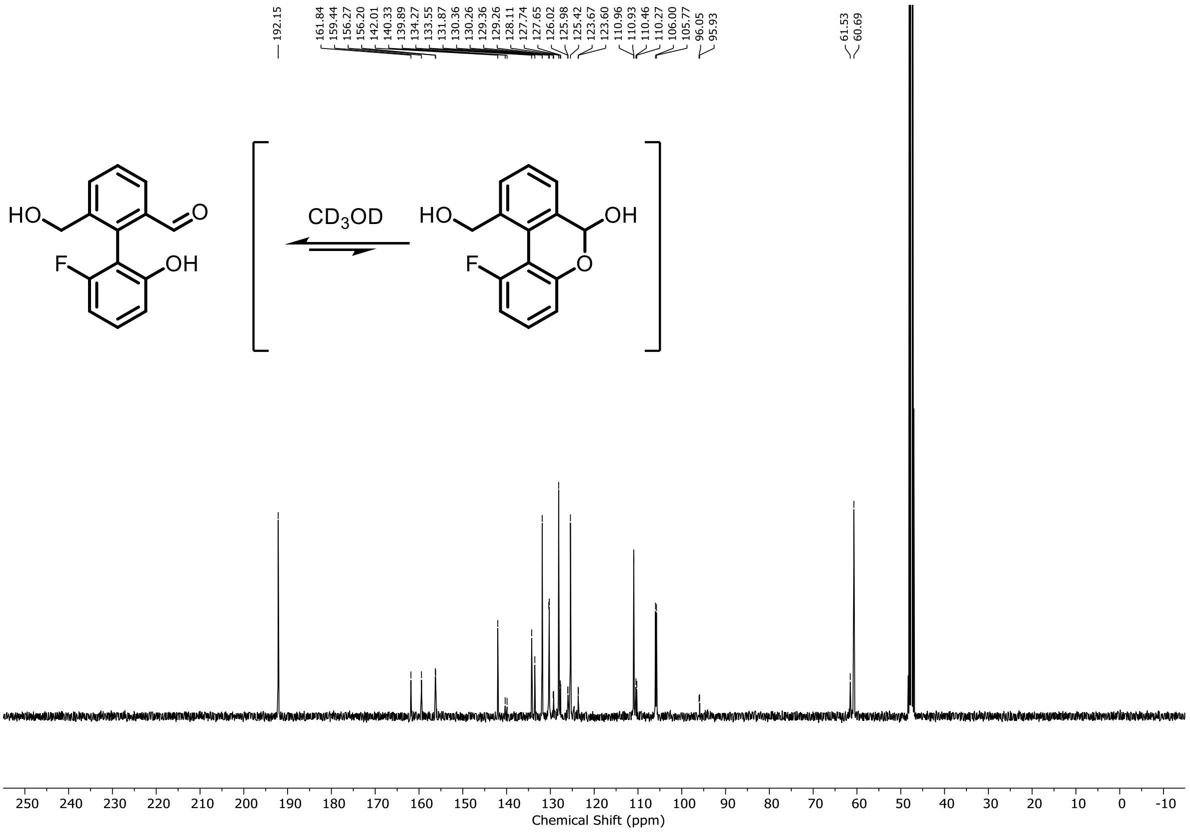

2'-Fluoro-6'-hydroxy-6-(hydroxymethyl)-[1,1'-biphenyl]-2-carbaldehyde (4a) (^{19}F NMR, 376 MHz, CD_3CN)

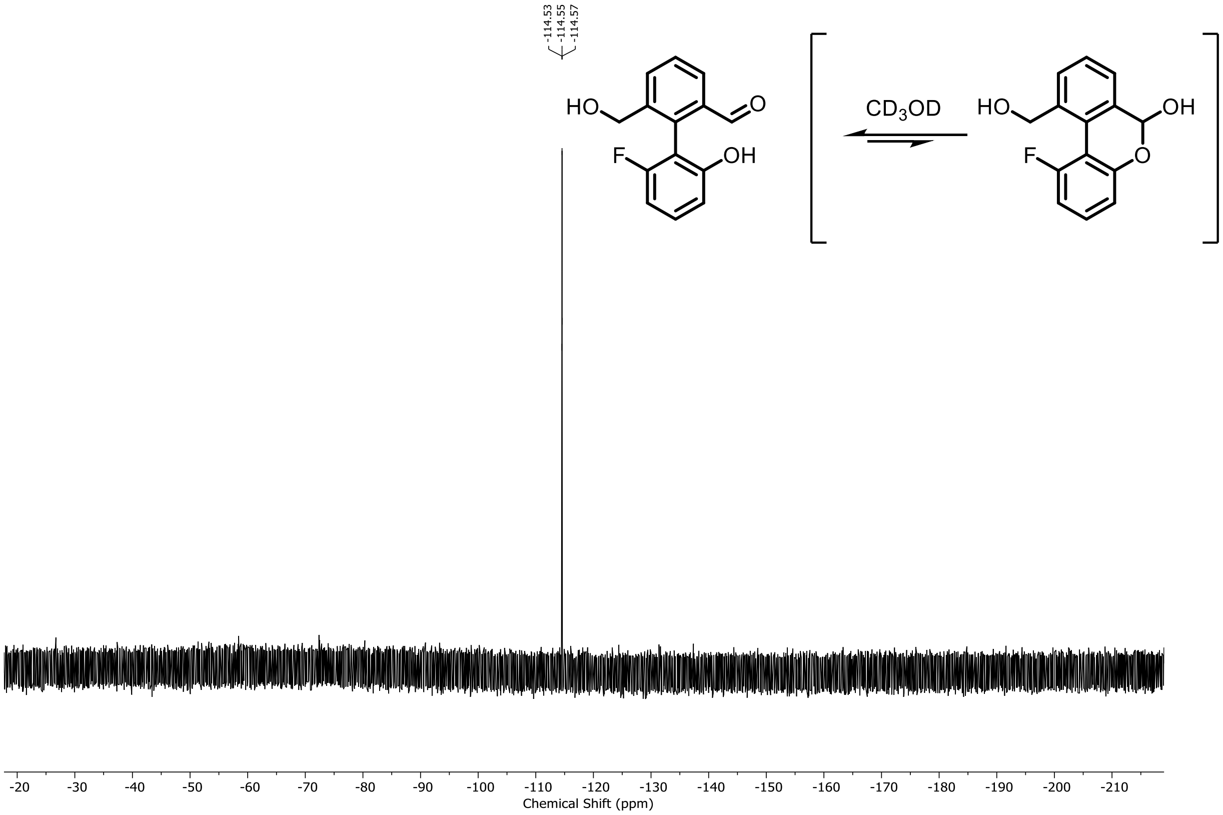

2'-Fluoro-6'-hydroxy-[1,1'-biphenyl]-2,6-dicarbaldehyde (S3e) (^{1}H NMR, 500 MHz, THF-d_8)

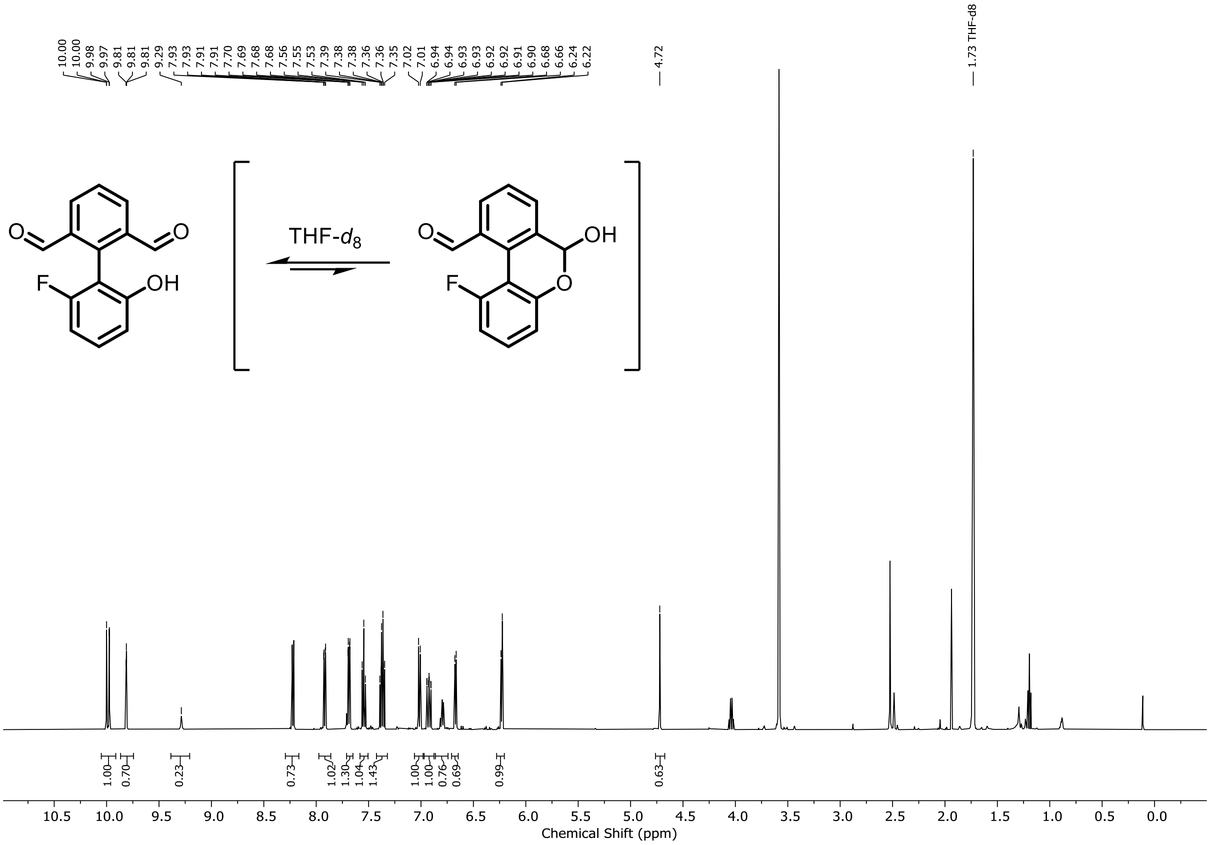

2'-Fluoro-6'-hydroxy-[1,1'-biphenyl]-2,6-dicarbaldehyde (S3e) (^{13}C NMR, 126 MHz, THF-d_8)

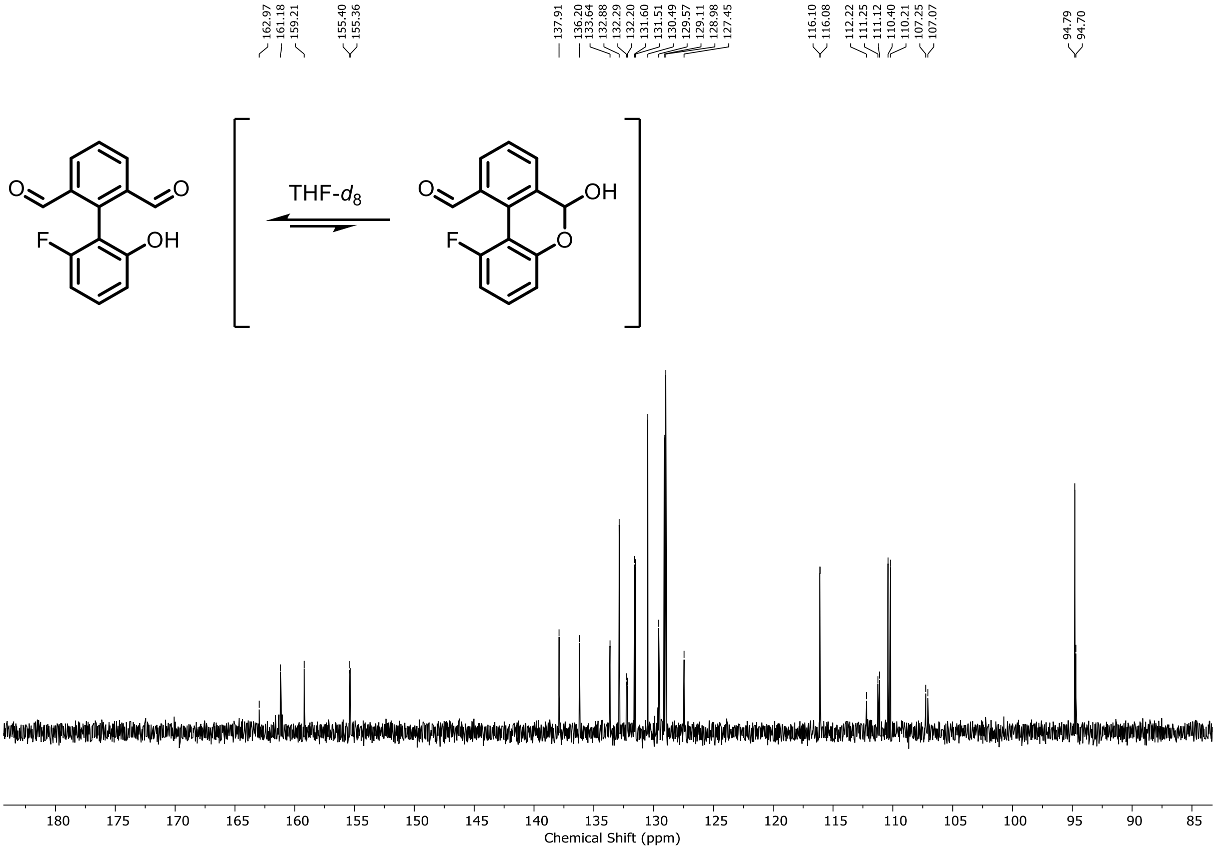

2'-Fluoro-6'-hydroxy-[1,1'-biphenyl]-2,6-dicarbaldehyde (S3e) (^{19}F NMR, 471 MHz, THF-d_8)

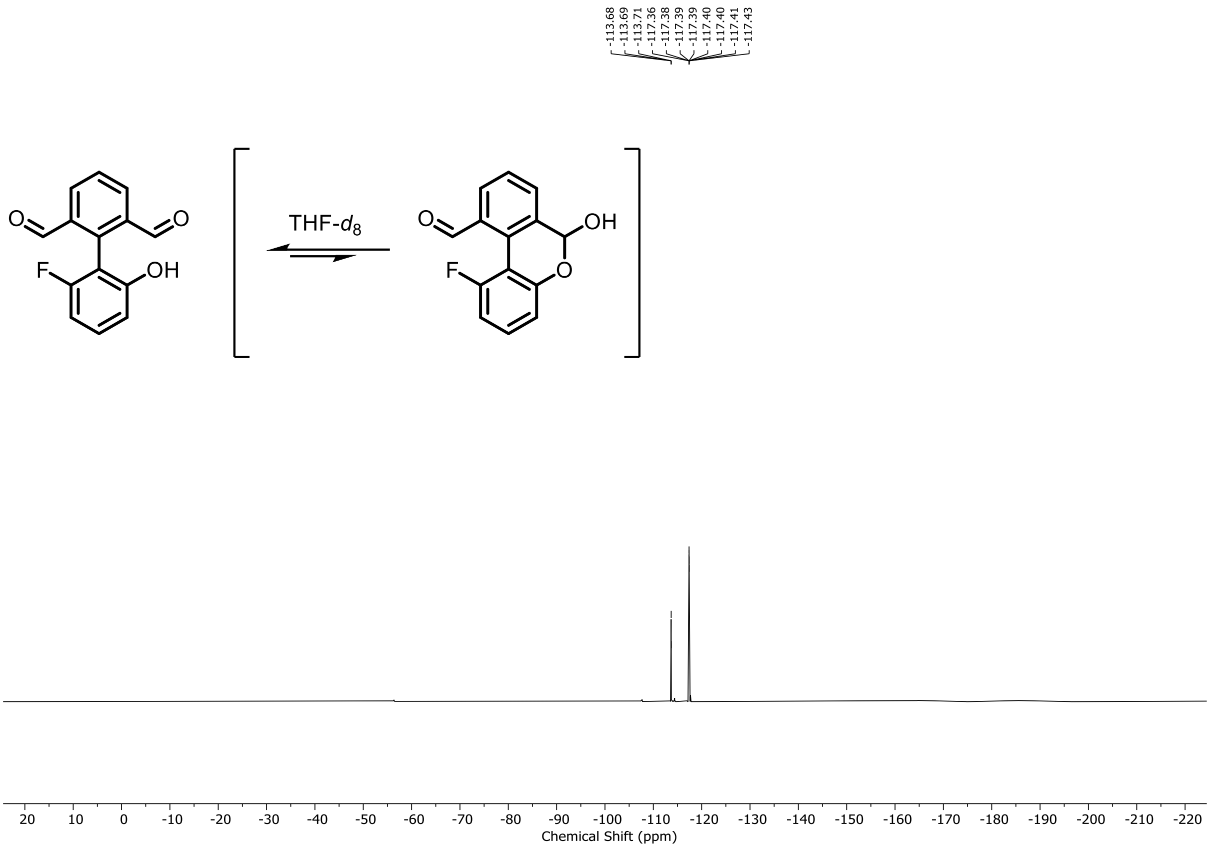

Methyl 1-fluoro-6-hydroxy-6H-benzo[c]chromene-10-carboxylate (S3i) (^{1}H NMR, 400 MHz, THF-d_8)

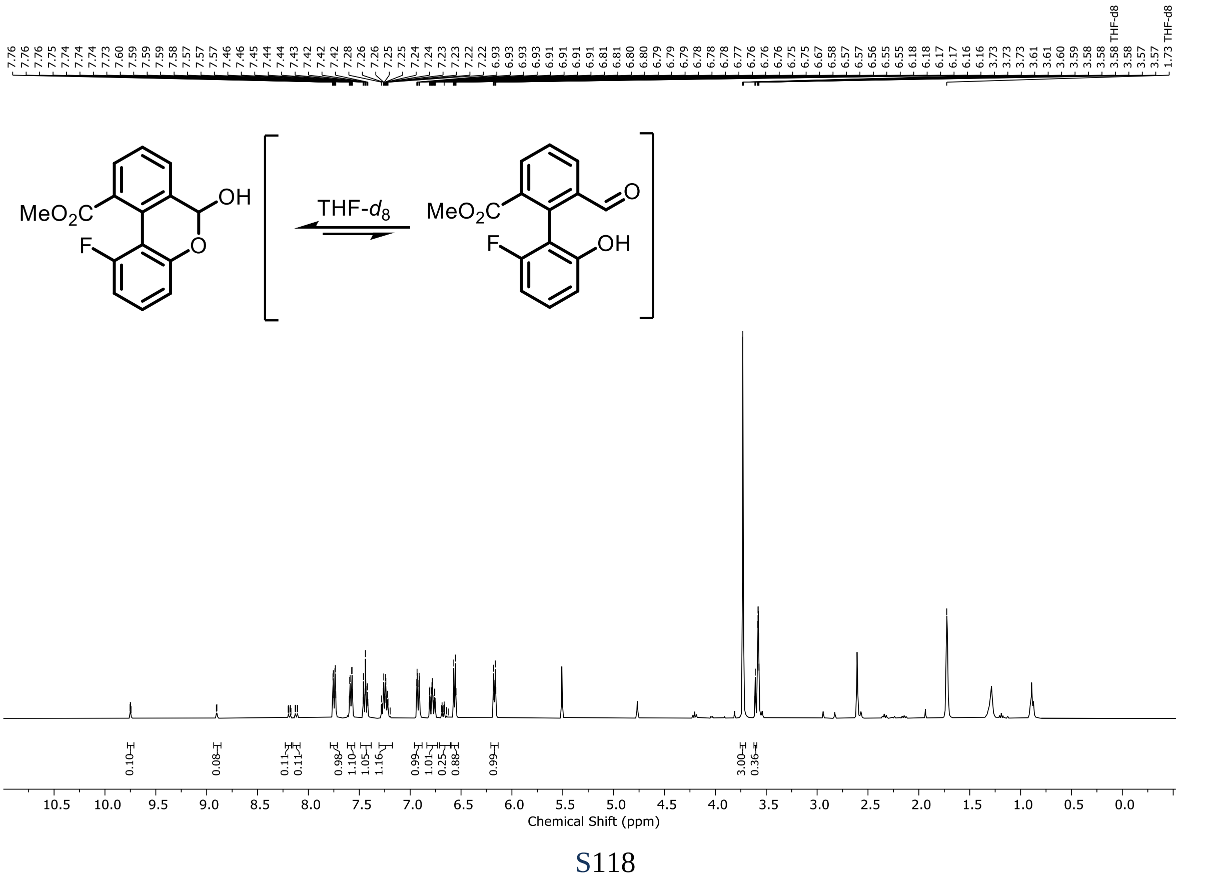

Methyl 1-fluoro-6-hydroxy-6H-benzo[c]chromene-10-carboxylate (S3i) (^{13}C NMR, 101 MHz, THF-d_8)

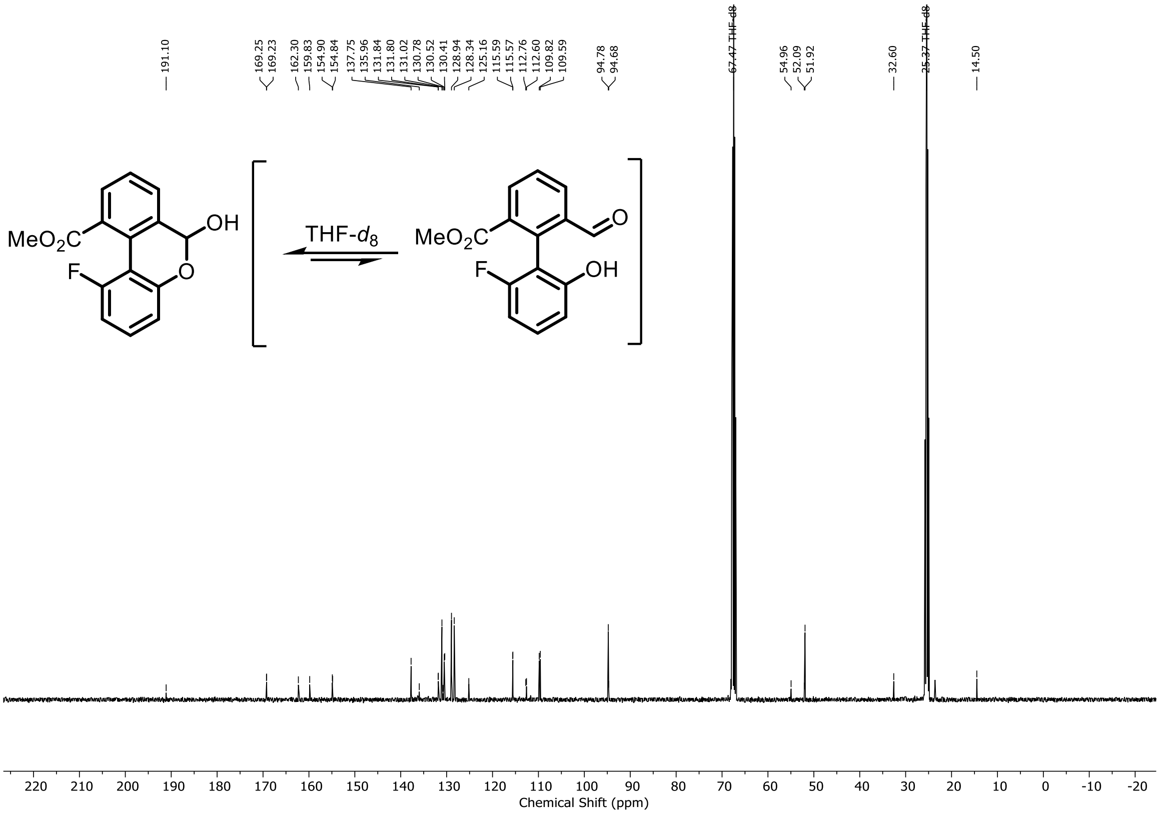

Methyl 1-fluoro-6-hydroxy-6H-benzo[c]chromene-10-carboxylate (S3i) (^{19}F NMR, 376 MHz, THF-d_8)

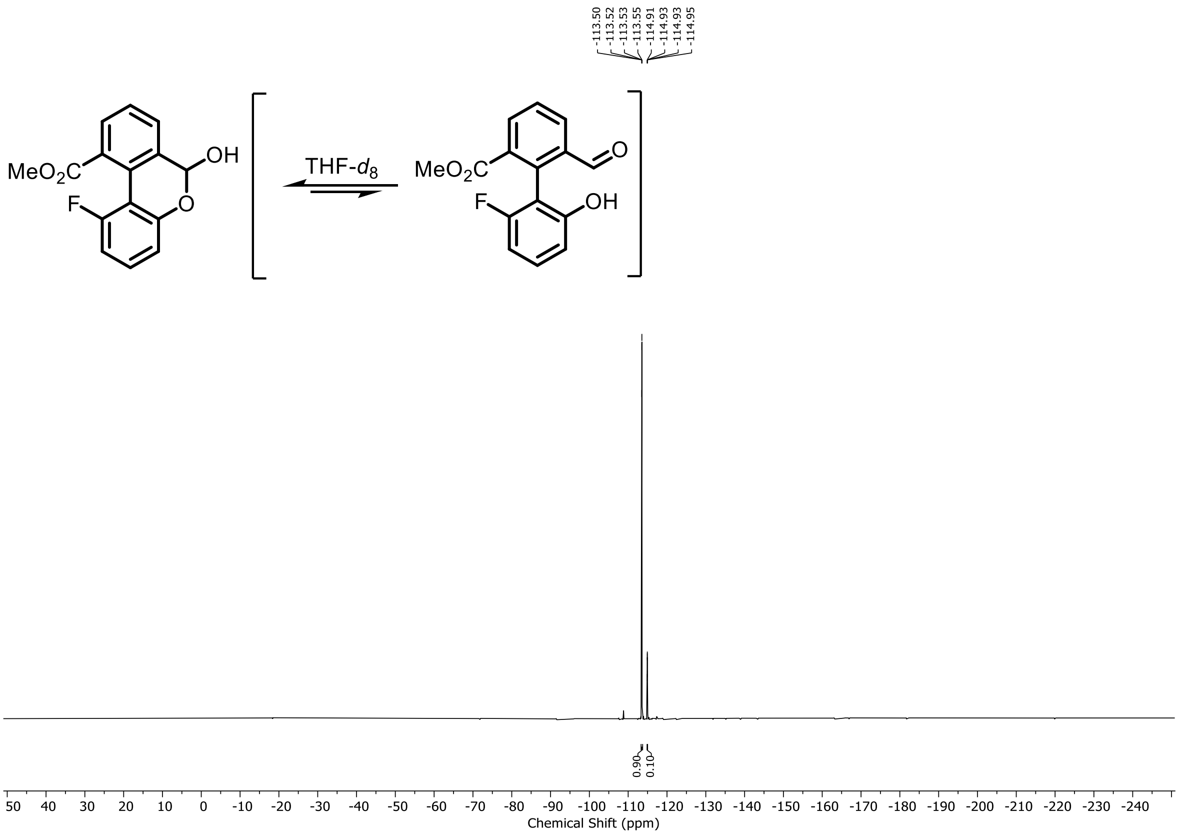

1-Fluoro-6-hydroxy-6H-benzo[c]chromene-10-carboxylic acid (S3j) (^{1}H NMR, 600 MHz, THF-d_8)

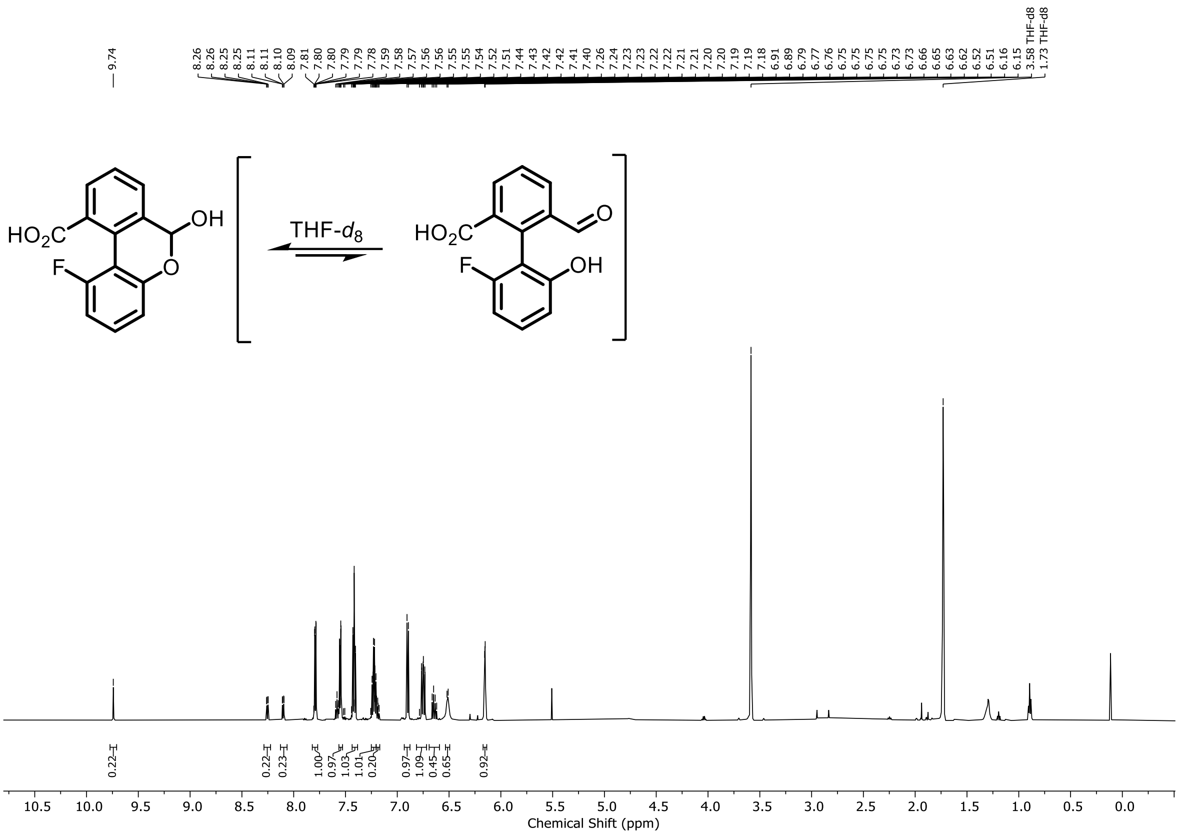

1-Fluoro-6-hydroxy-6H-benzo[c]chromene-10-carboxylic acid (S3j) (^{13}C NMR, 151 MHz, THF-d_8)

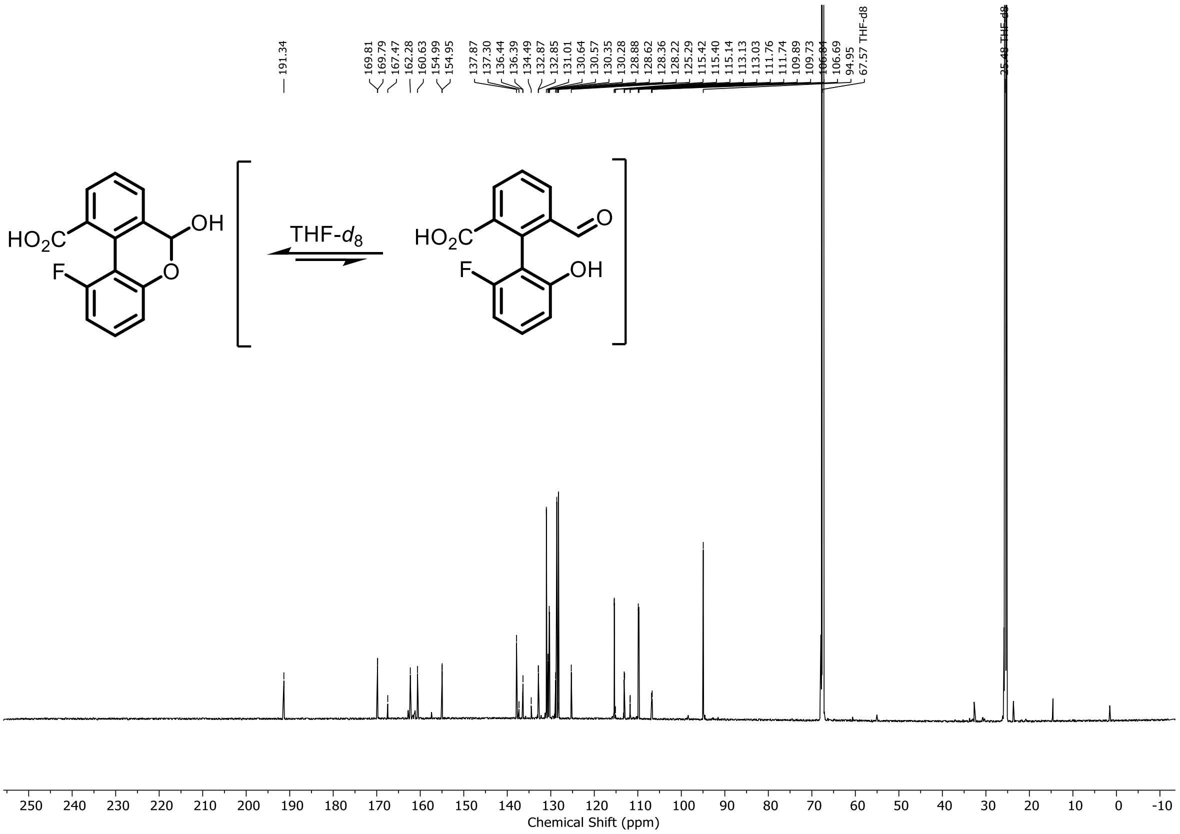

1-Fluoro-6-hydroxy-6H-benzo[c]chromene-10-carboxylic acid (S3j) (^{19}F NMR, 565 MHz, THF-d_8)

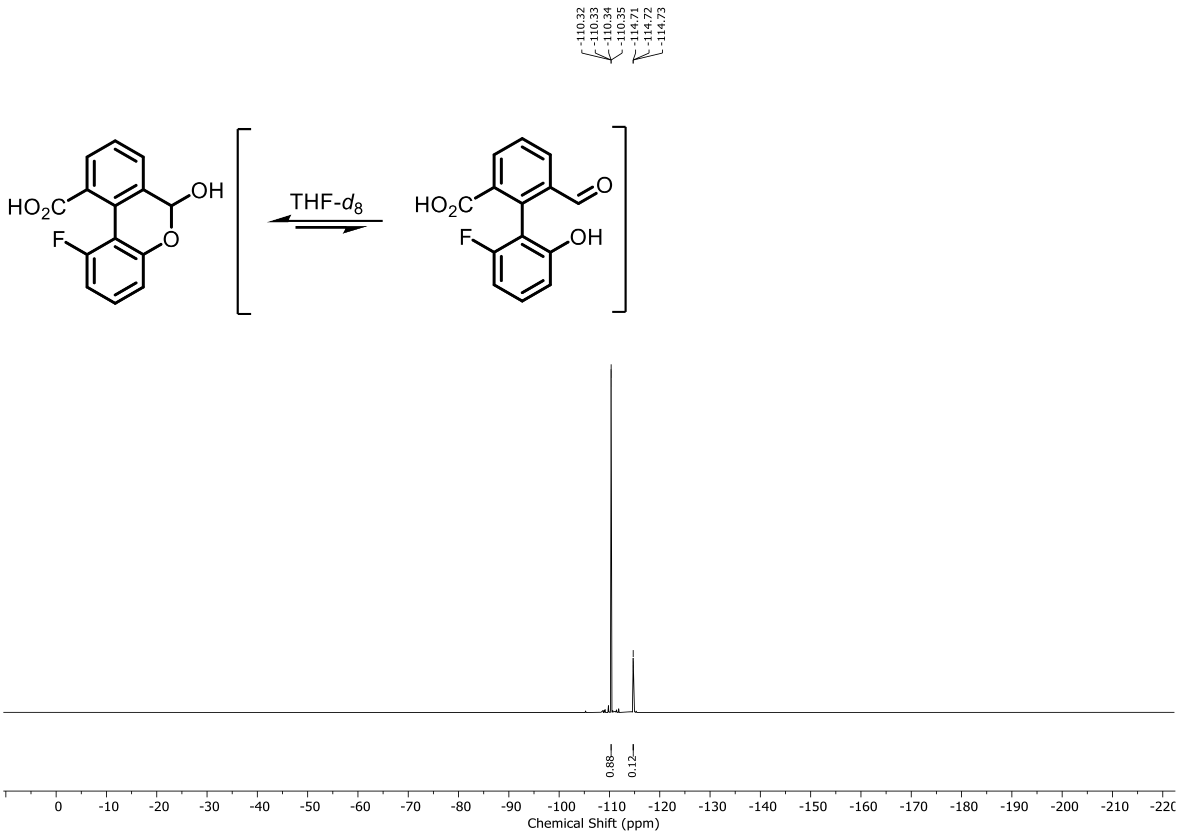

Lithium 2'-fluoro-6'-oxido-[1,1'-biphenyl]-2,6-dicarboxylate (S3k′) (^{1}H NMR, 600 MHz, D_2O/THF-d_8)

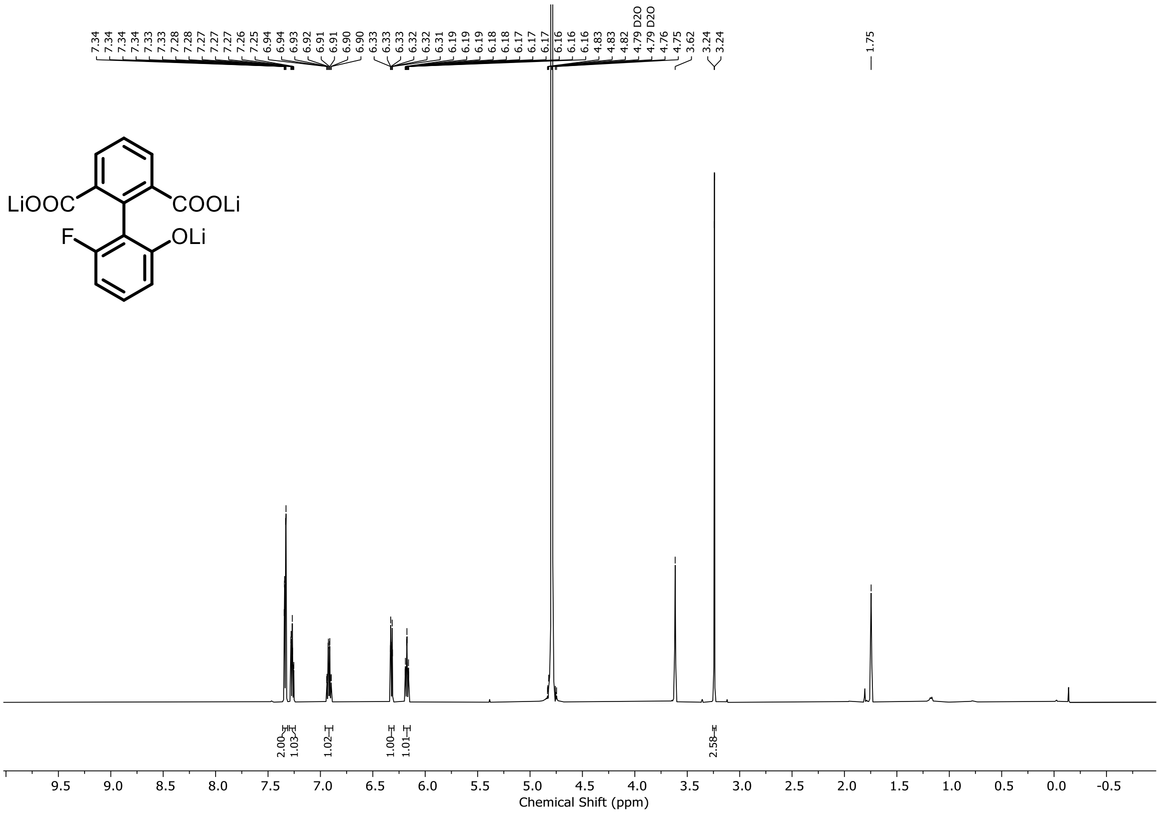

Lithium 2'-fluoro-6'-oxido-[1,1'-biphenyl]-2,6-dicarboxylate (S3k′) (^{13}C NMR, 151 MHz, D_2O/THF-d_8)

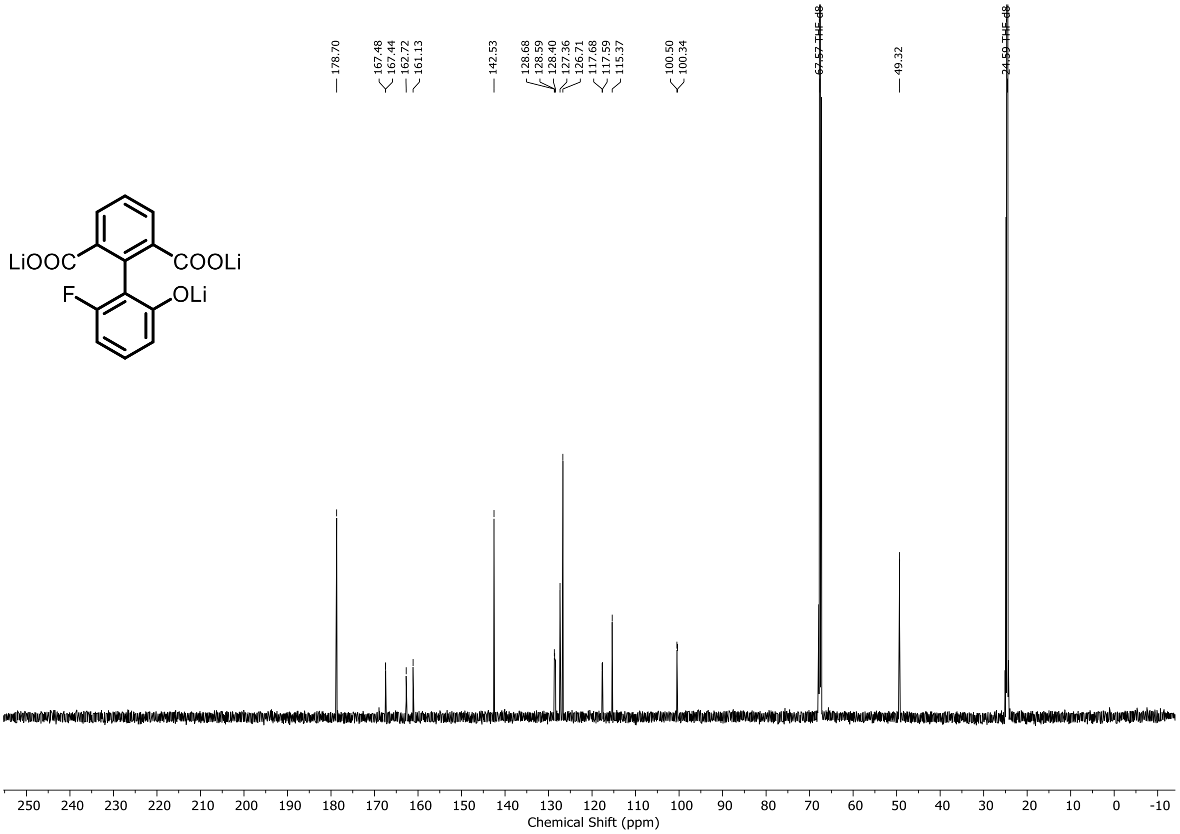

Lithium 2'-fluoro-6'-oxido-[1,1'-biphenyl]-2,6-dicarboxylate (S3k′) (^{19}F NMR, 565 MHz, D_2O/THF-d_8)

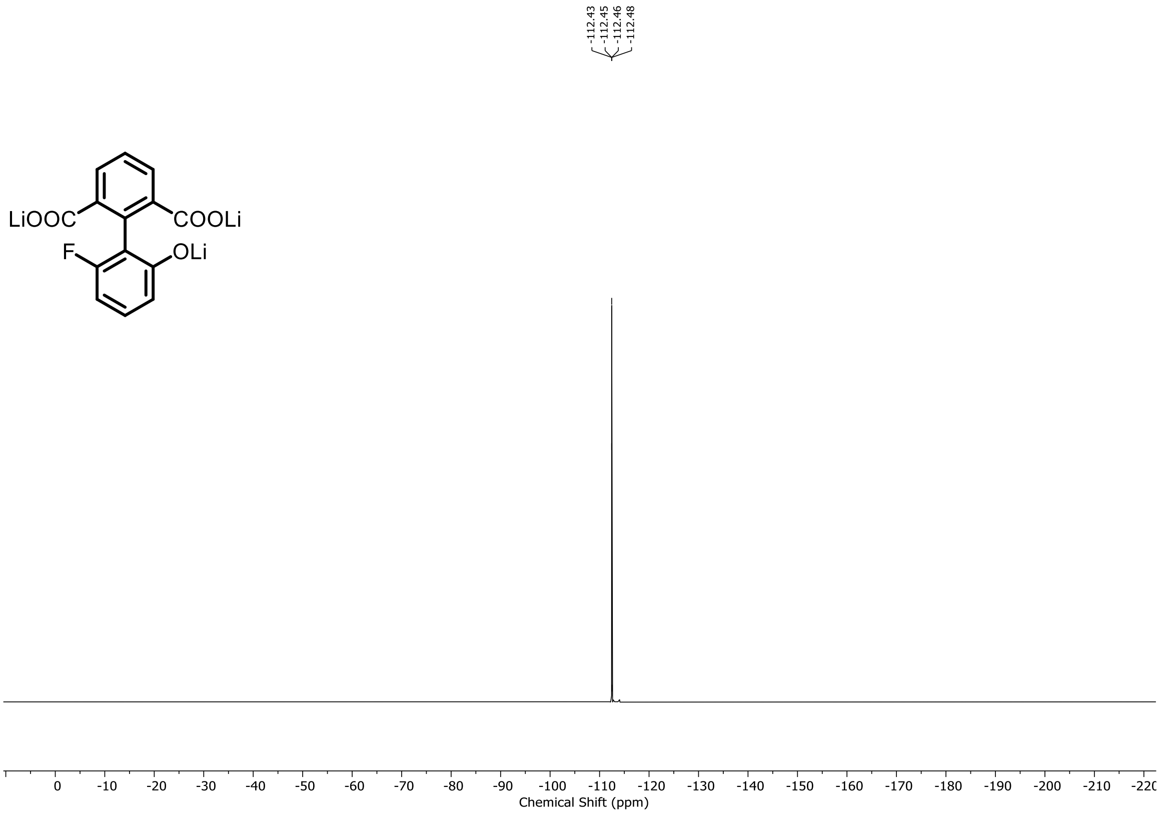

(2'-Fluoro-6-formyl-6'-hydroxy-[1,1'-biphenyl]-2-yl)methyl (2*S*)-3,3,3-trifluoro-2-methoxy-2-phenylpropanoate (6a) (^{1}H NMR, 500 MHz, $(CD_3)_2SO$)

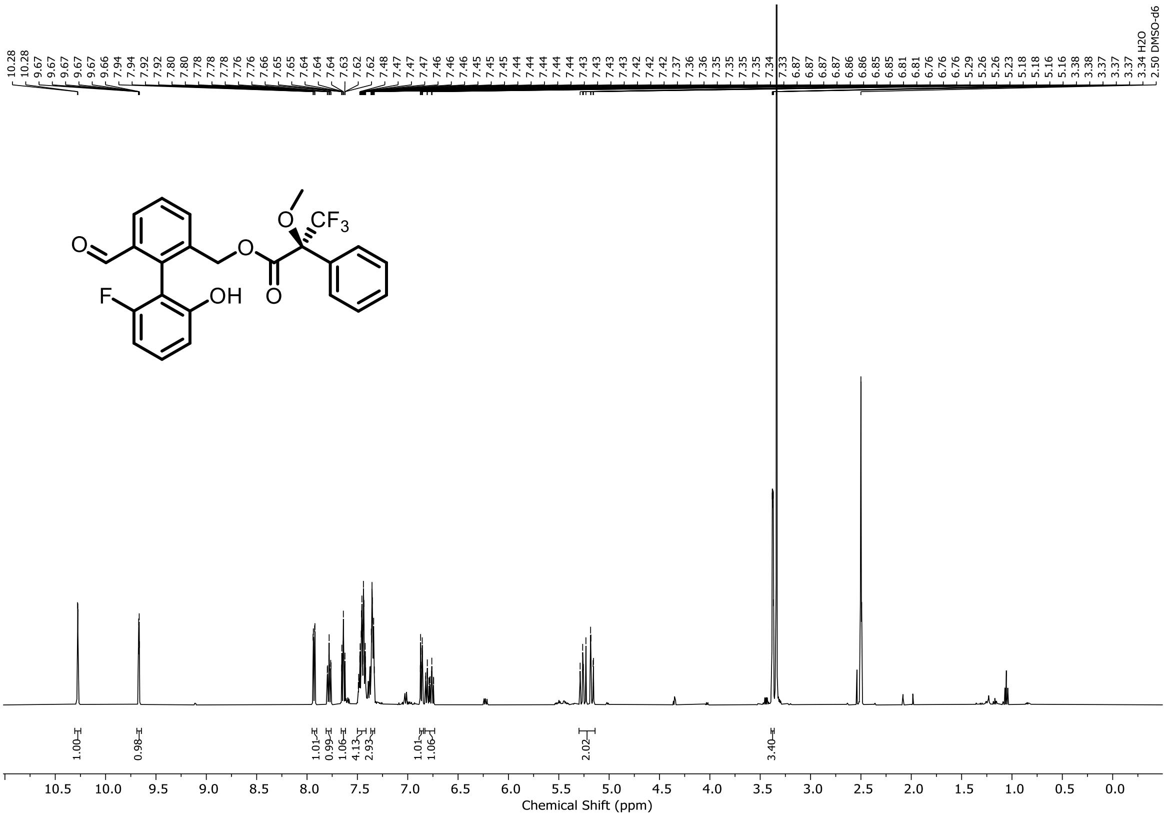

(2'-Fluoro-6-formyl-6'-hydroxy-[1,1'-biphenyl]-2-yl)methyl (2*S*)-3,3,3-trifluoro-2-methoxy-2-phenylpropanoate (6a) (^{13}C NMR, 126 MHz, $(CD_3)_2SO$)

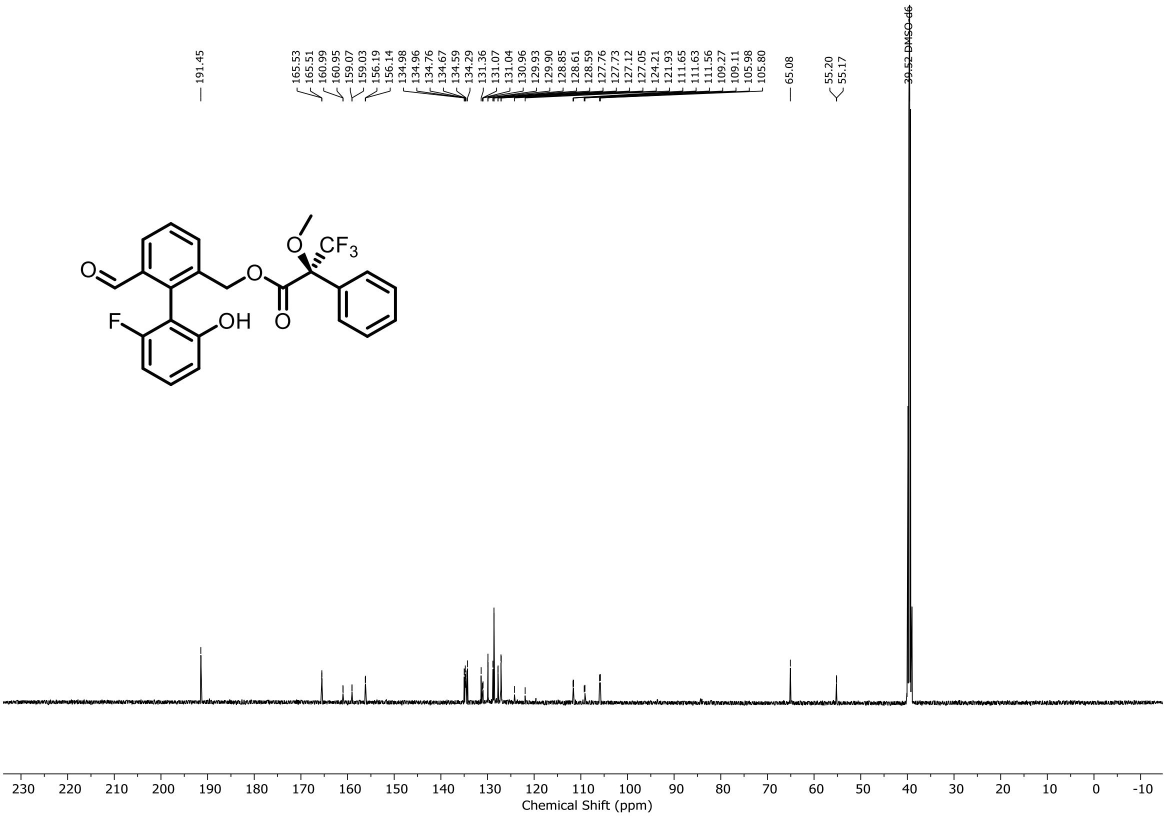

(2'-Fluoro-6-formyl-6'-hydroxy-[1,1'-biphenyl]-2-yl)methyl (2*S*)-3,3,3-trifluoro-2-methoxy-2-phenylpropanoate (6a) (^{19}F NMR, 376 MHz, $(CD_3)_2SO$)

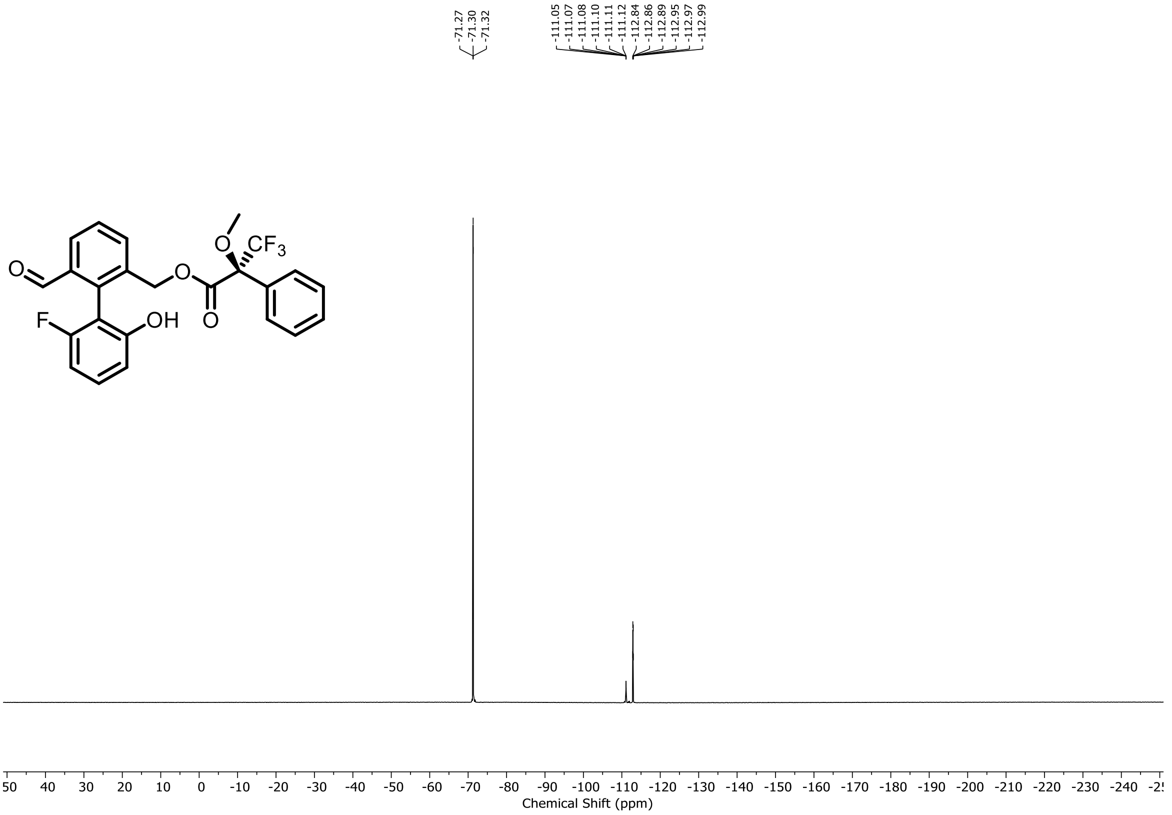

Methyl 2'-fluoro-6'-hydroxy-6-(hydroxymethyl)-[1,1'-biphenyl]-2-carboxylate (S3f) (^{1}H NMR, 400 MHz, CD_3OD)

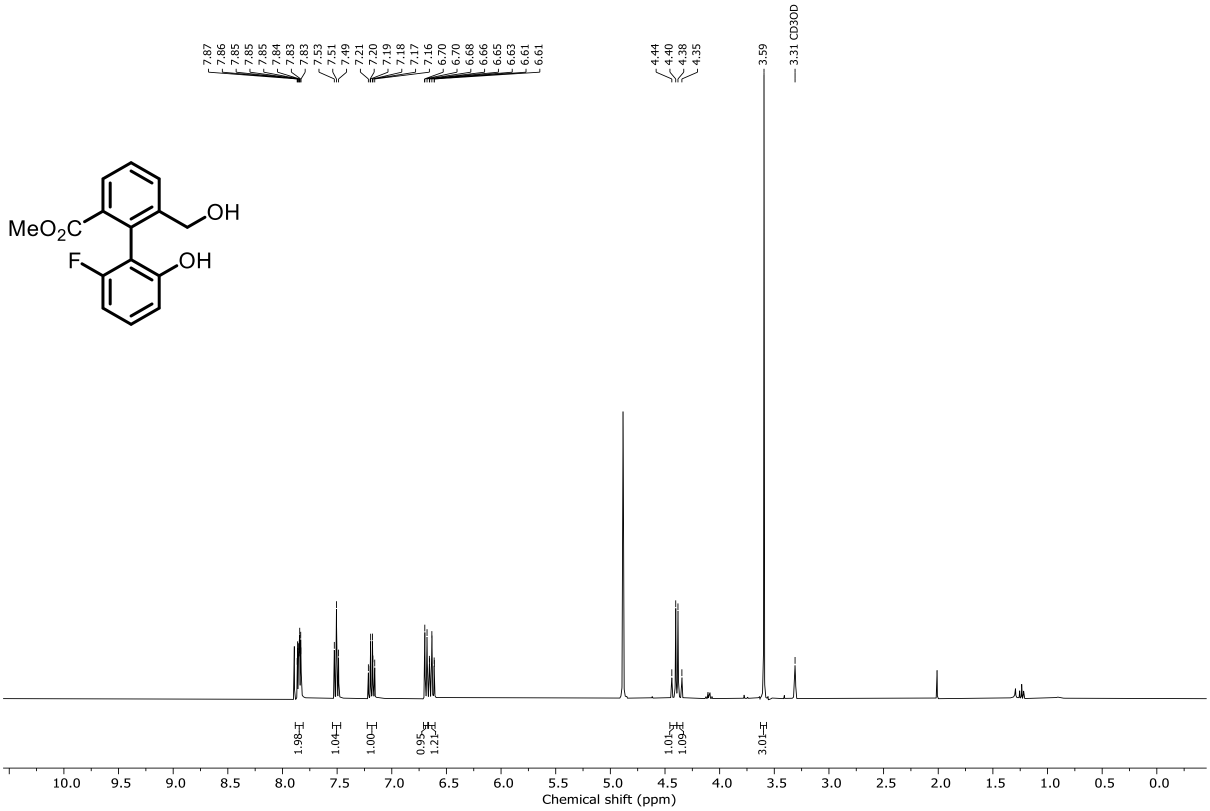

Methyl 2'-fluoro-6'-hydroxy-6-(hydroxymethyl)-[1,1'-biphenyl]-2-carboxylate (S3f) (^{13}C NMR, 101 MHz, CD_3OD)

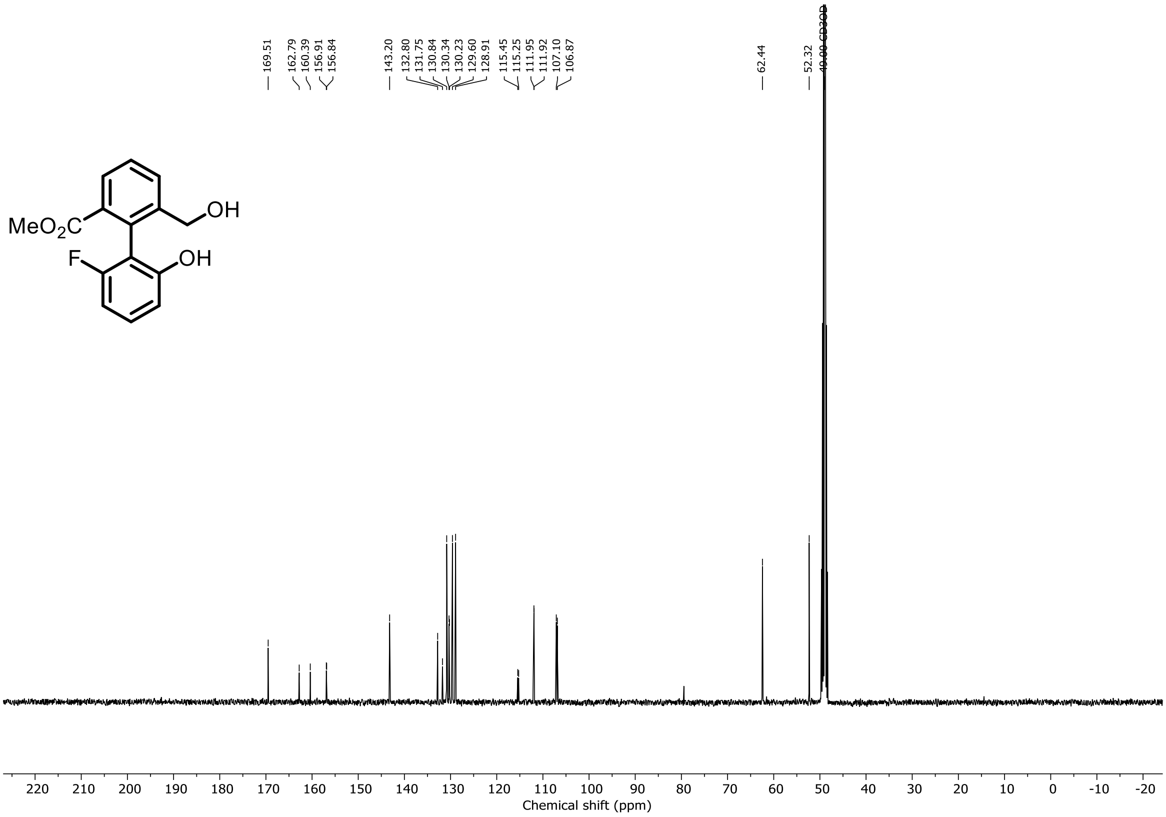

Methyl 2'-fluoro-6'-hydroxy-6-(hydroxymethyl)-[1,1'-biphenyl]-2-carboxylate (S3f) (^{19}F NMR, 376 MHz, CD_3OD)

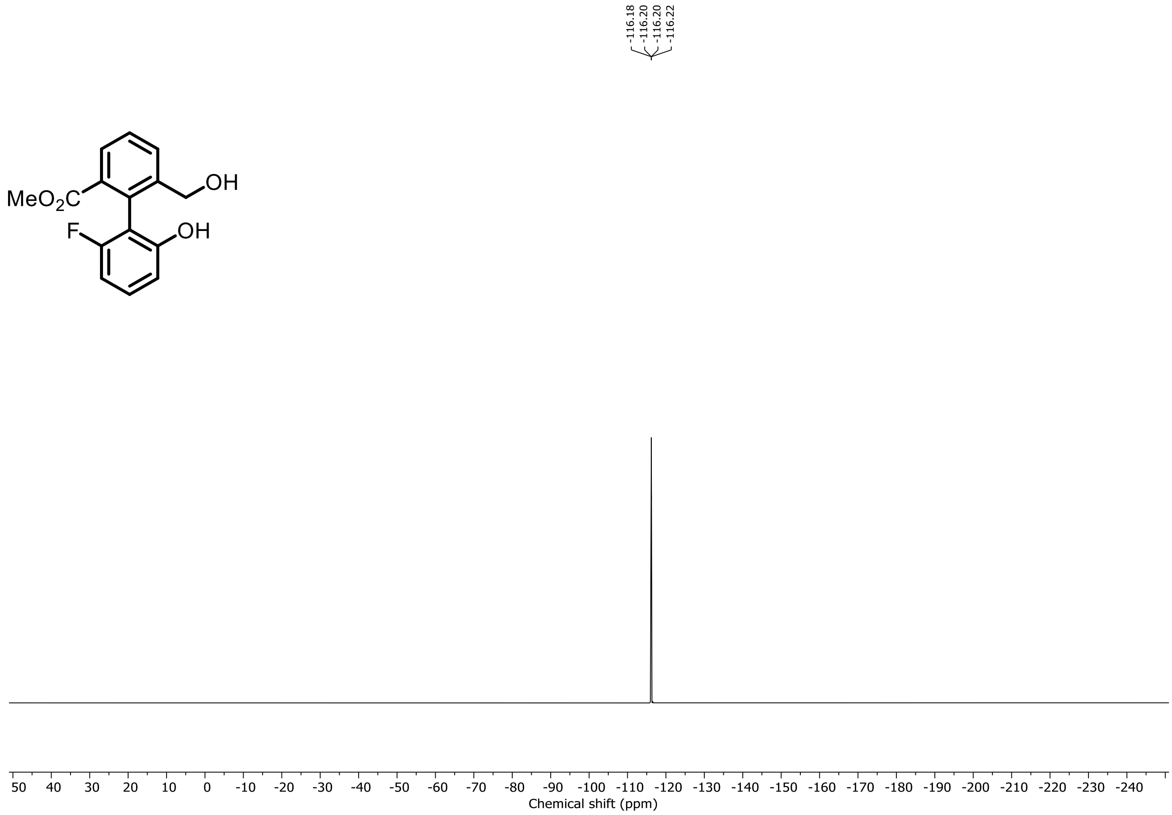

Methyl 2'-fluoro-6'-hydroxy-6-(hydroxymethyl-d2)-[1,1'-biphenyl]-2-carboxylate (D$_2$-S3f) (^{1}H NMR, 400 MHz, CD_3OD)

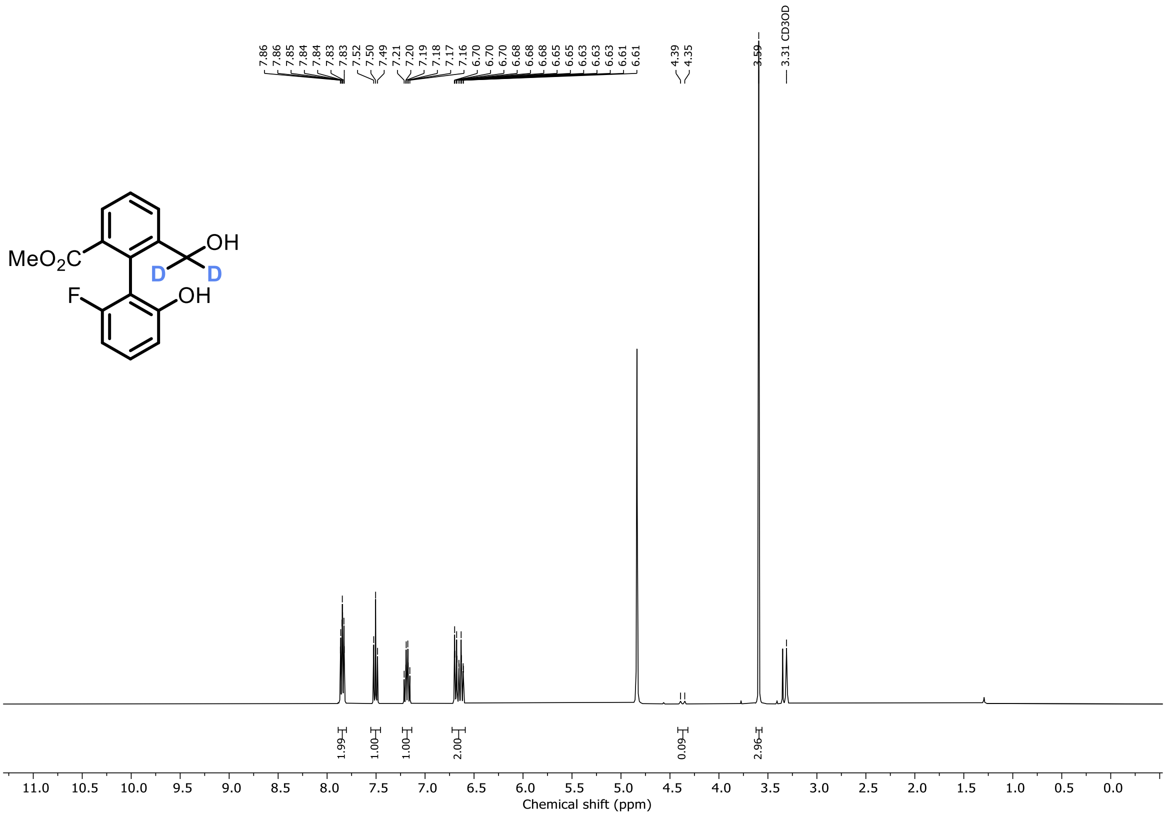

1-Fluoro-10-(hydroxymethyl)-6H-benzo[c]chromen-6-one (S3g) (^{1}H NMR, 400 MHz, $CDCl_3$)

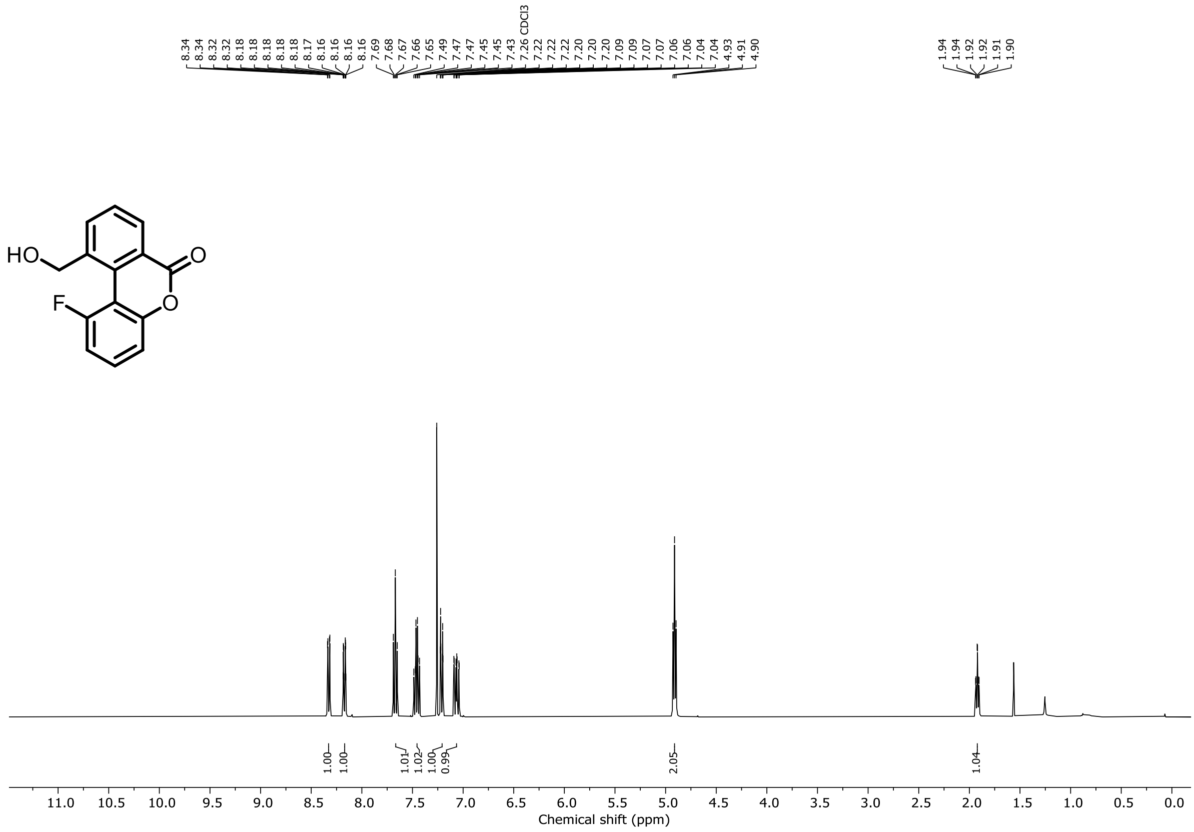

1-Fluoro-10-(hydroxymethyl)-6H-benzo[c]chromen-6-one (S3g) (^{13}C NMR, 101 MHz, $CDCl_3$)

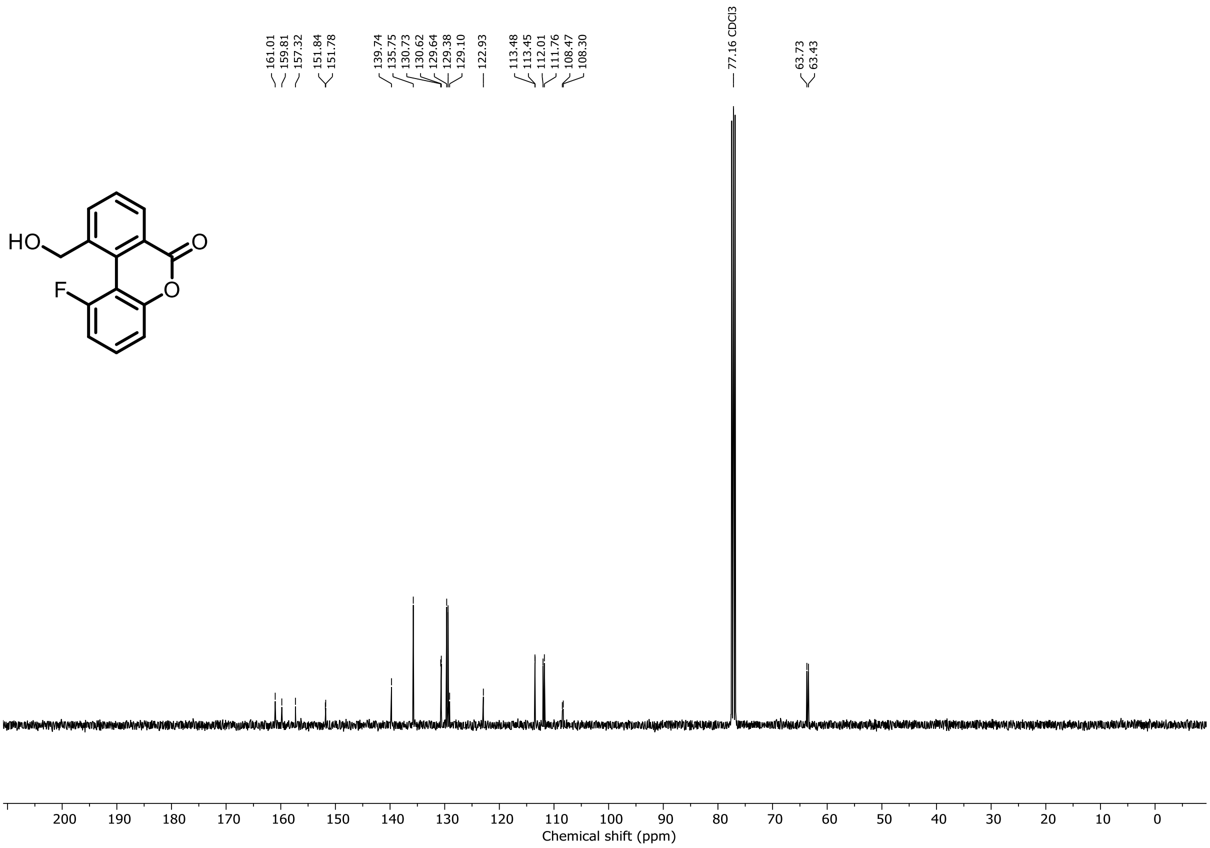

1-Fluoro-10-(hydroxymethyl)-6H-benzo[c]chromen-6-one (S3g) (^{19}F NMR, 377 MHz, $CDCl_3$)

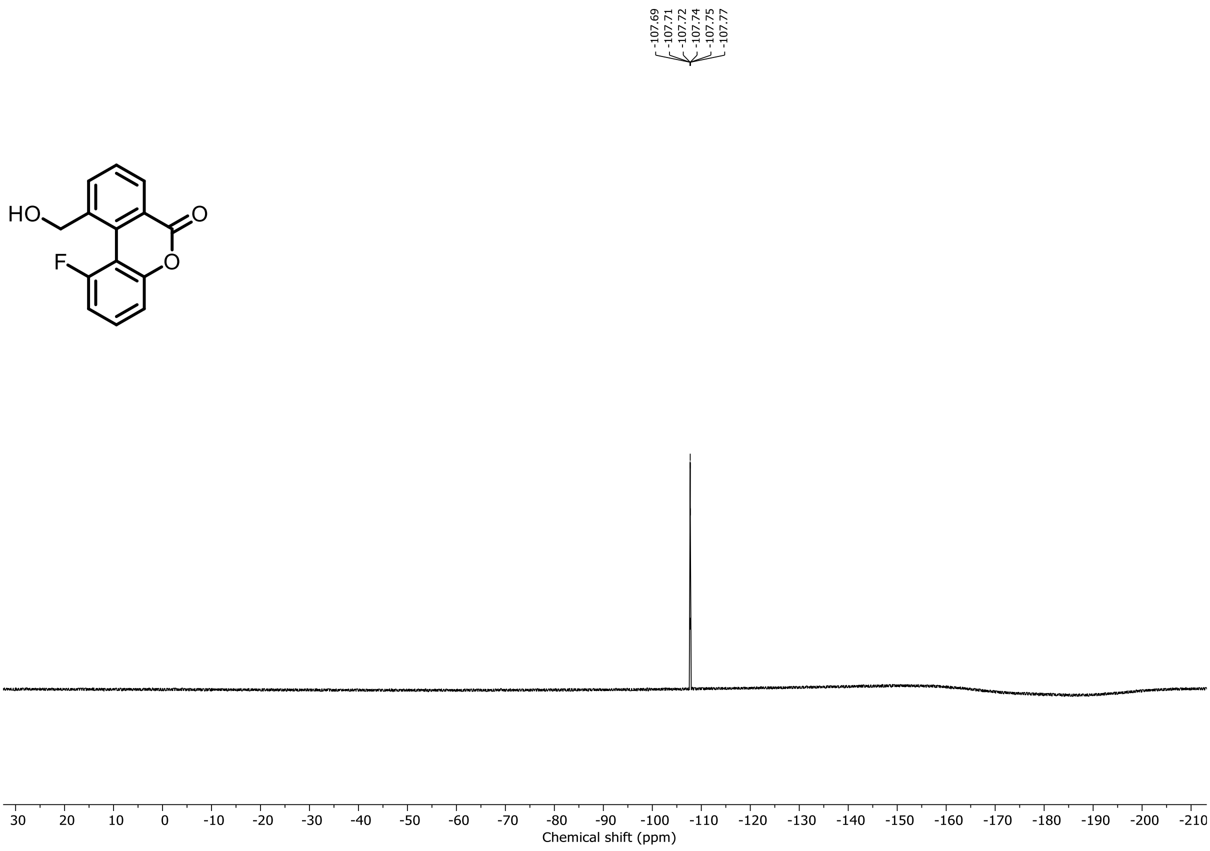

1-Fluoro-10-(hydroxymethyl-d$_2$)-6H-benzo[c]chromen-6-one (D$_2$-S3g) (^{1}H NMR, 400 MHz, $CDCl_3$)

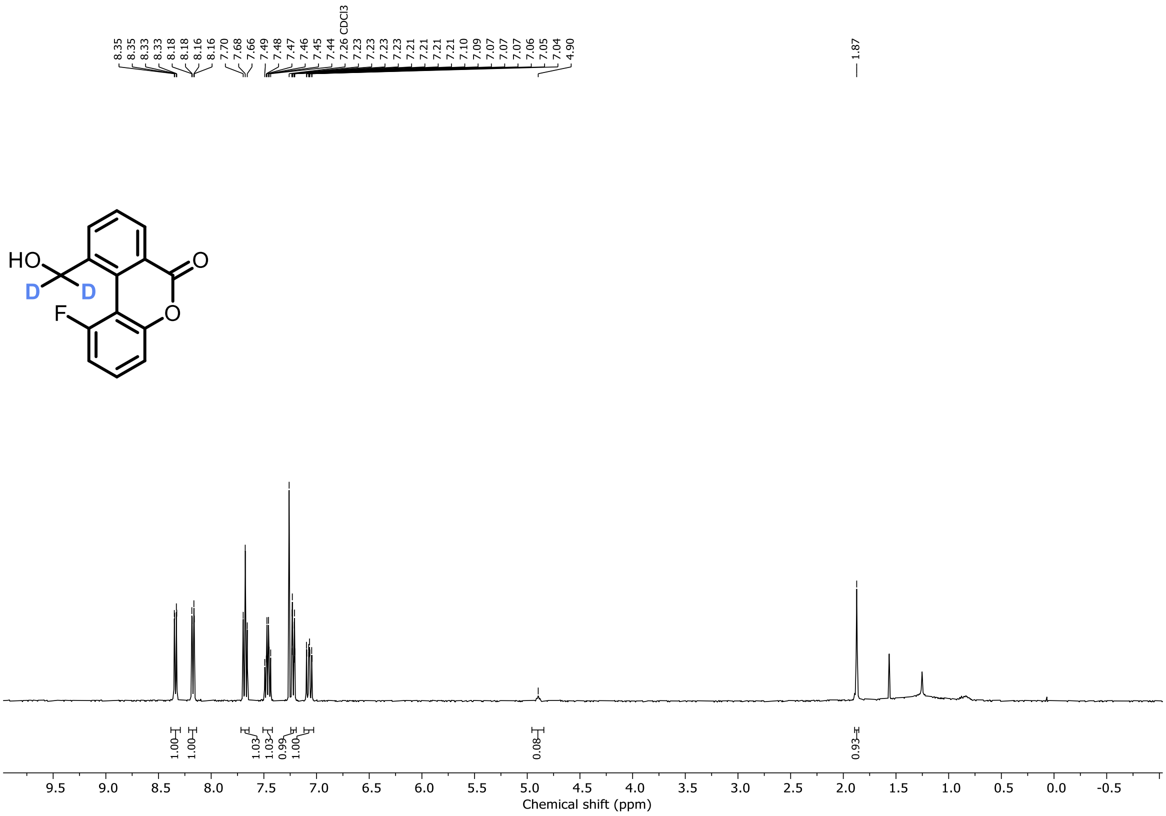

10-(((*tert*-Butyldimethylsilyl)oxy)methyl)-1-fluoro-6H-benzo[c]chromen-6-one (S3h) (^{1}H NMR, 400 MHz, $CDCl_3$)

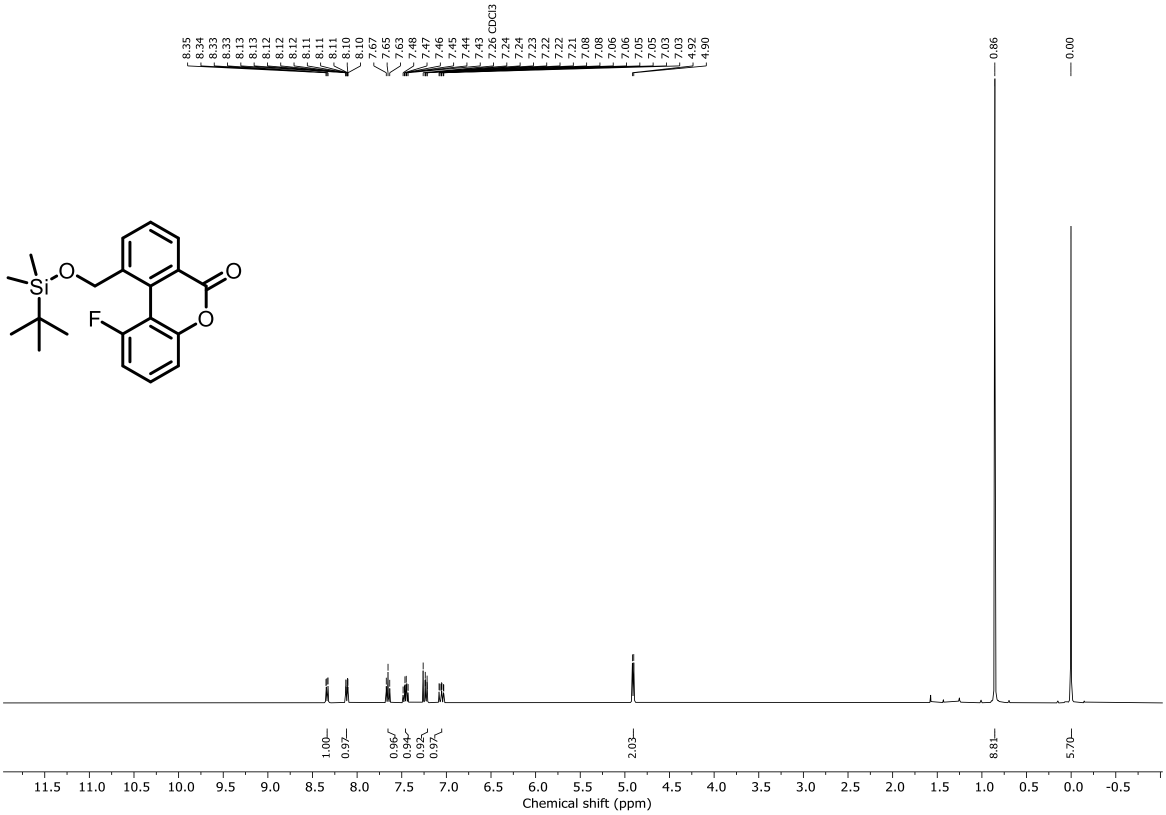

10-(((*tert*-Butyldimethylsilyl)oxy)methyl)-1-fluoro-6H-benzo[c]chromen-6-one (S3h) (^{13}C NMR, 101 MHz, $CDCl_3$)

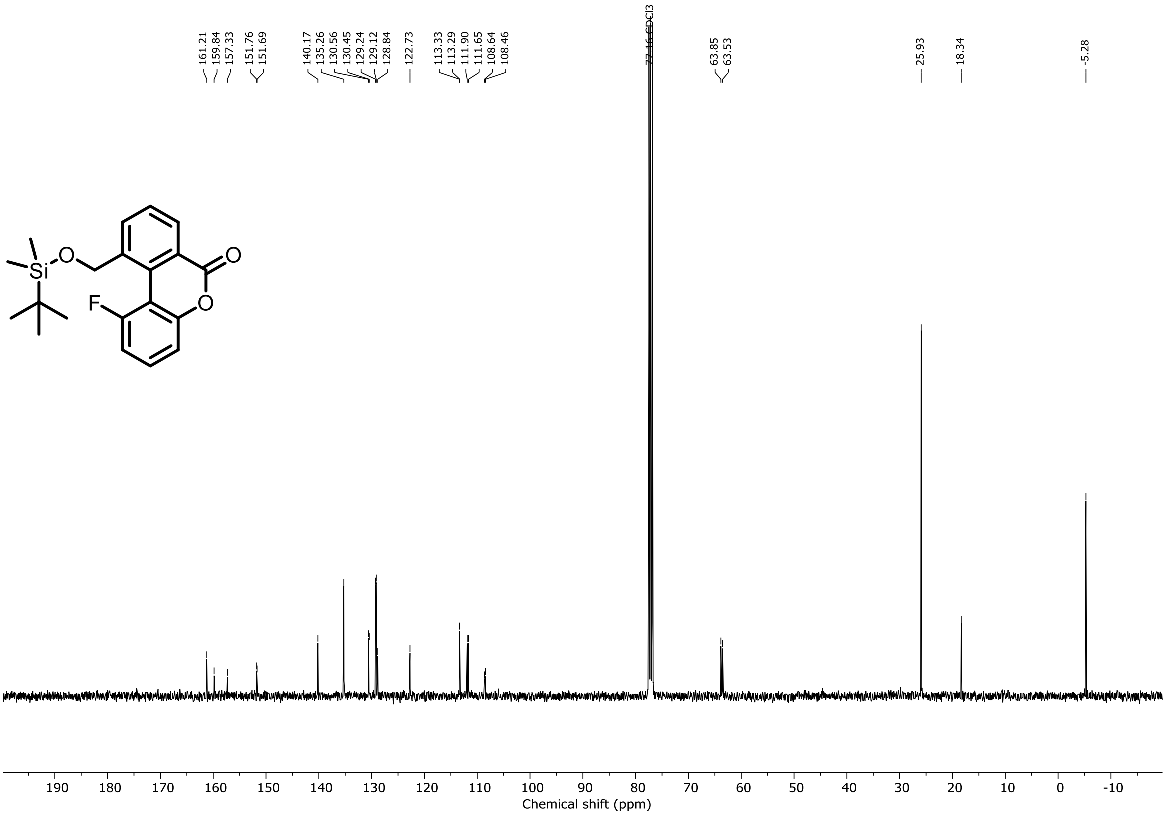

10-(((*tert*-Butyldimethylsilyl)oxy)methyl)-1-fluoro-6H-benzo[c]chromen-6-one (S3h) (^{19}F NMR, 377 MHz, $CDCl_3$)

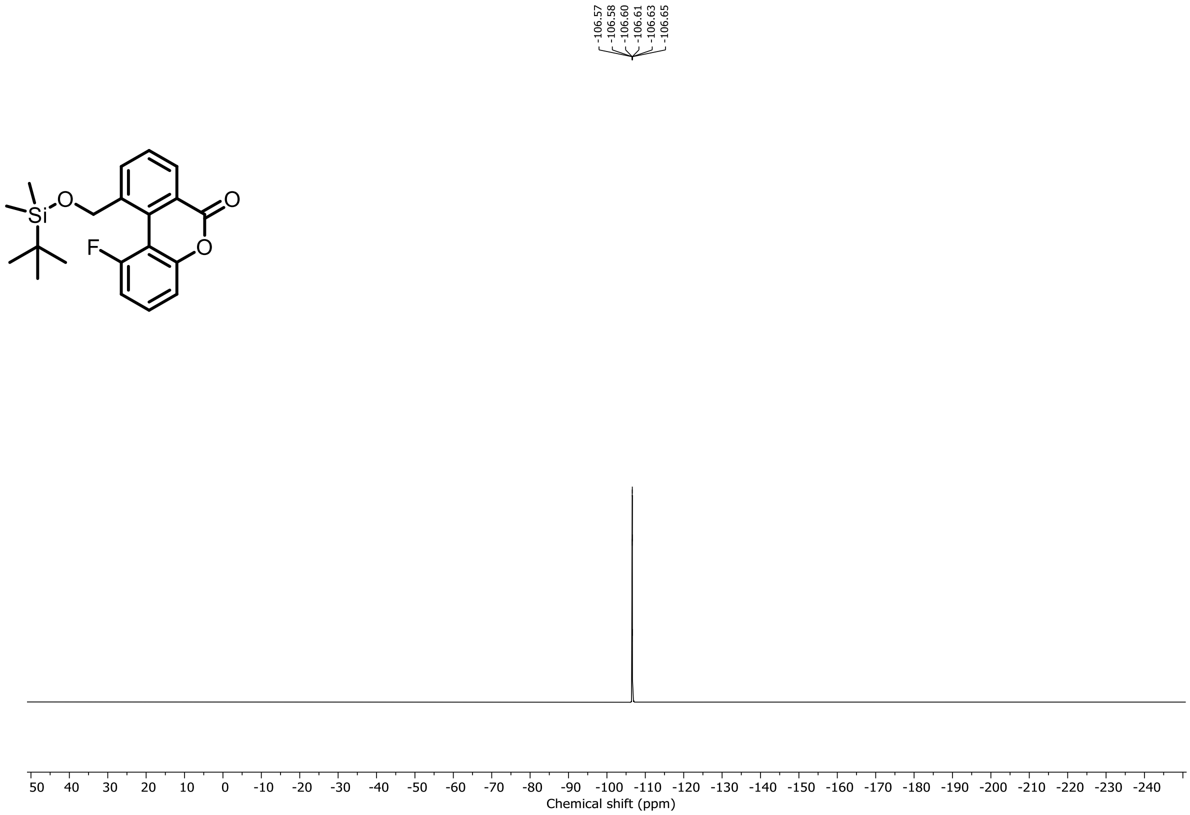

10-(((*tert*-Butyldimethylsilyl)oxy)methyl-d$_2$)-1-fluoro-6H-benzo[c]chromen-6-one (D$_2$-S3h)

(^{1}H NMR, 400 MHz, $CDCl_3$)

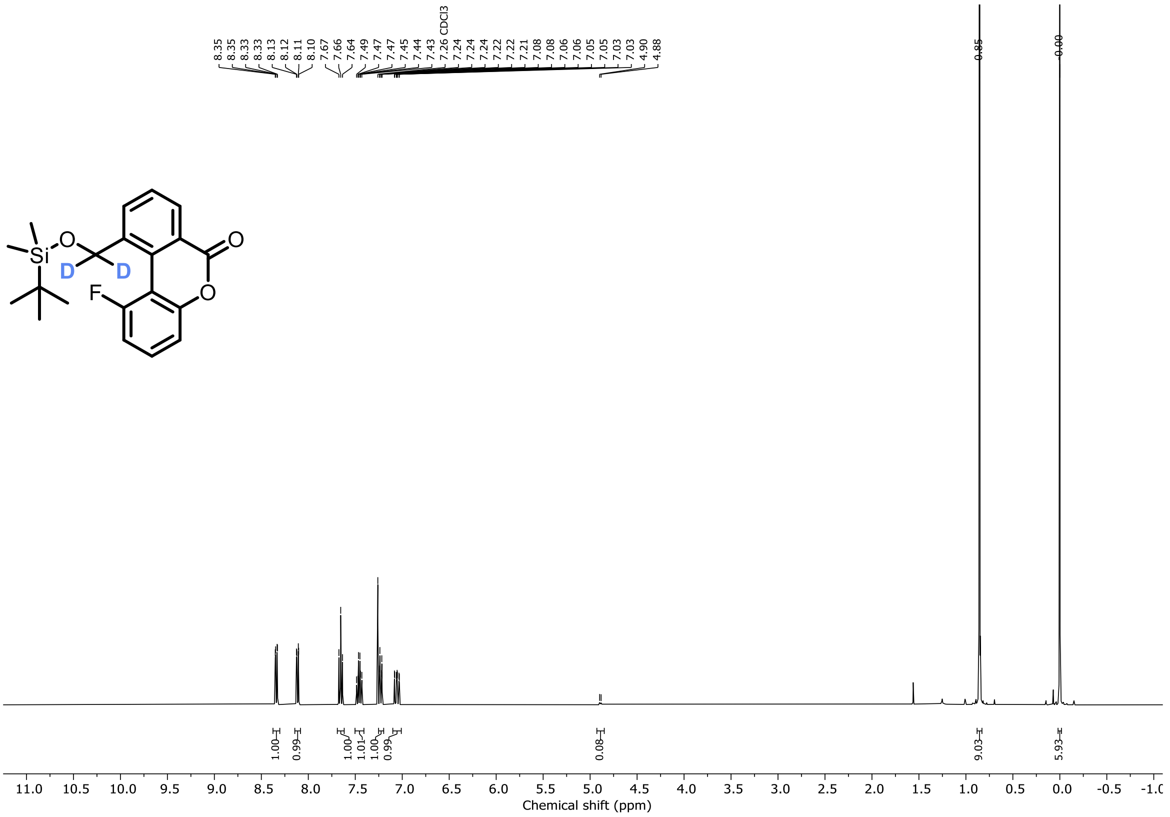

2'-(((*tert*-Butyldimethylsilyl)oxy)methyl)-6-fluoro-6'-(hydroxymethyl)-[1,1'-biphenyl]-2-ol (5a)

(^{1}H NMR, 400 MHz, CD_3OD)

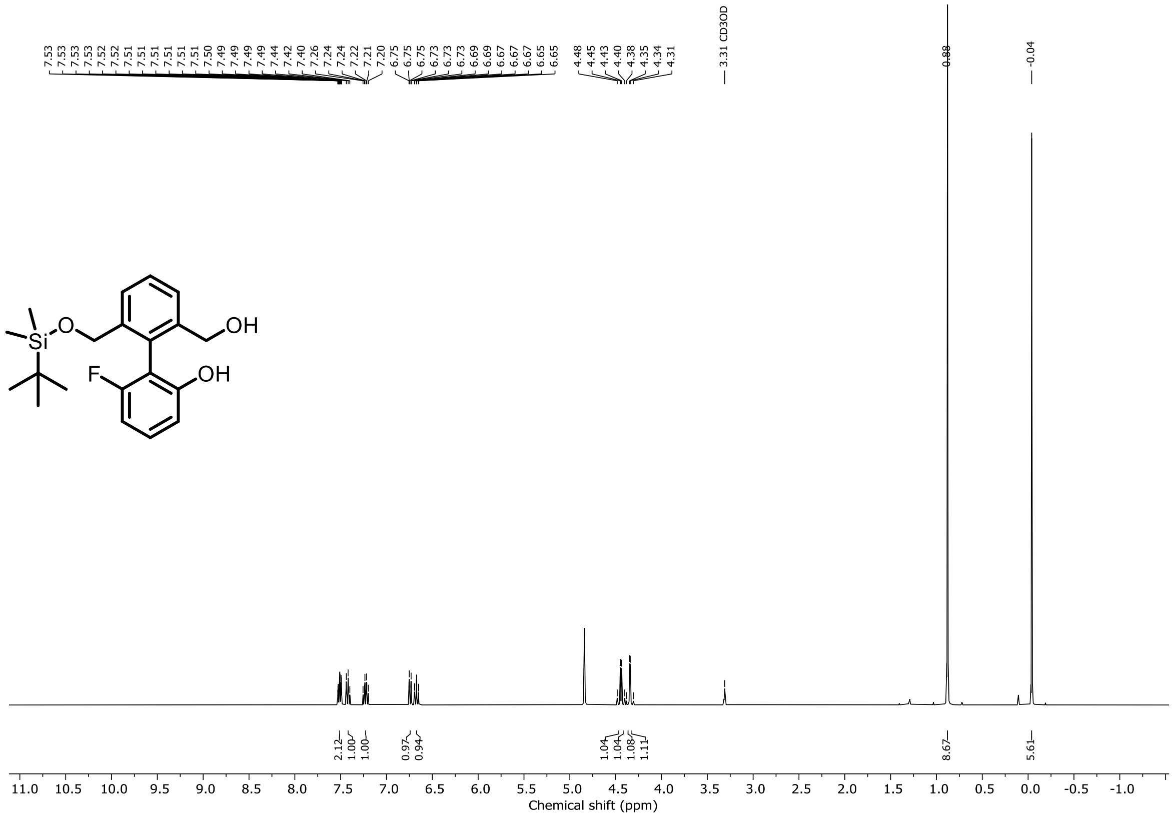

2'-(((*tert*-Butyldimethylsilyl)oxy)methyl)-6-fluoro-6'-(hydroxymethyl)-[1,1'-biphenyl]-2-ol (5a)

(^{13}C NMR, 101 MHz, CD_3OD)

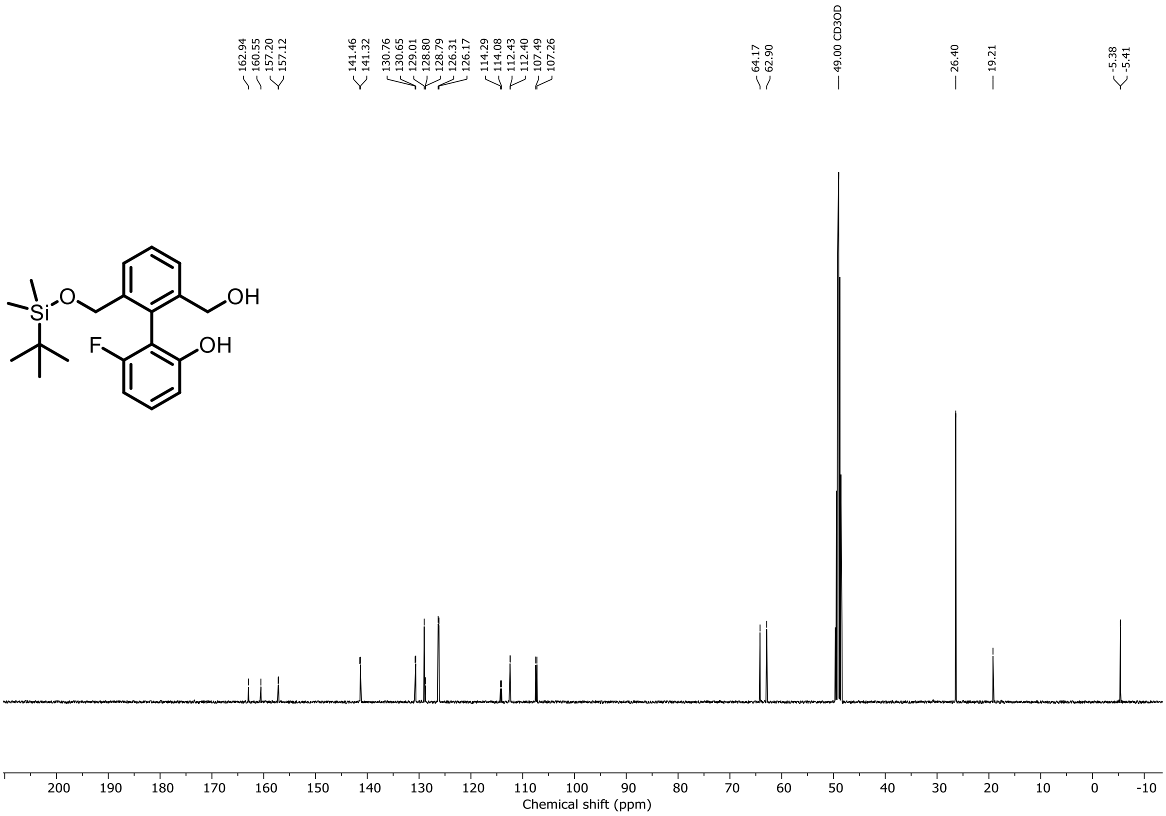

2'-(((*tert*-Butyldimethylsilyl)oxy)methyl)-6-fluoro-6'-(hydroxymethyl)-[1,1'-biphenyl]-2-ol (5a)

(^{19}F NMR, 377 MHz, CD_3OD)

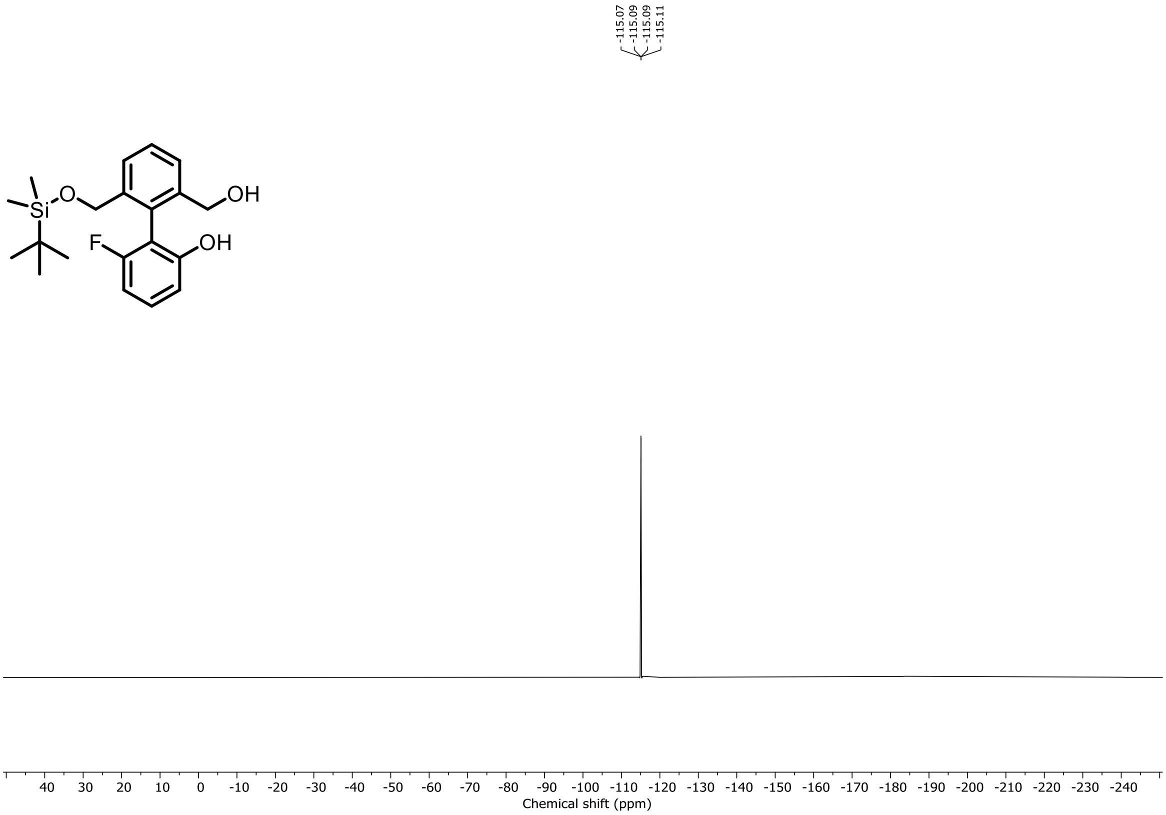

2'-(((*tert*-Butyldimethylsilyl)oxy)methyl-d2)-6-fluoro-6'-(hydroxymethyl)-[1,1'-biphenyl]-2-ol (D_2-5a) (^1H NMR, 400 MHz, CD_3OD)

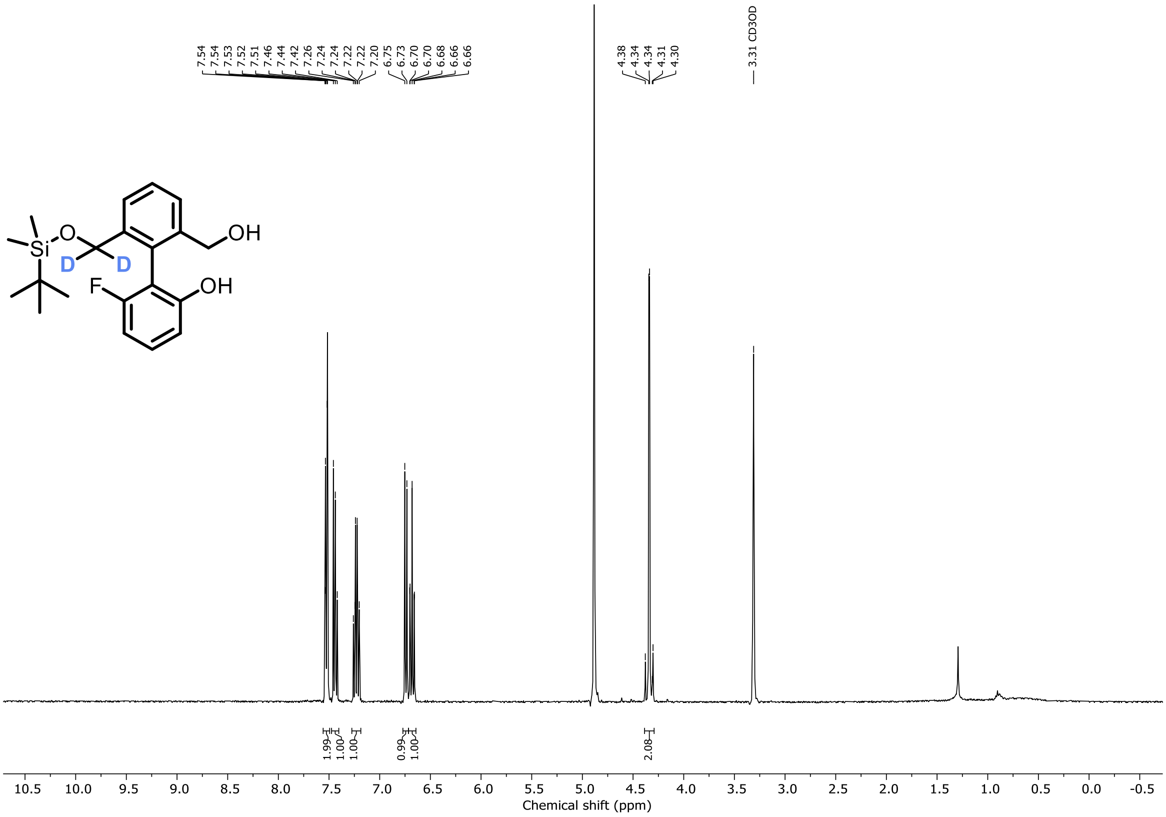

(S_a)-6-Fluoro-2'-(hydroxymethyl)-6'-(hydroxymethyl-d_2)-[1,1'-biphenyl]-2-ol ((S_a)-D_2-3a) (^1H NMR, 400 MHz, CD_3OD)

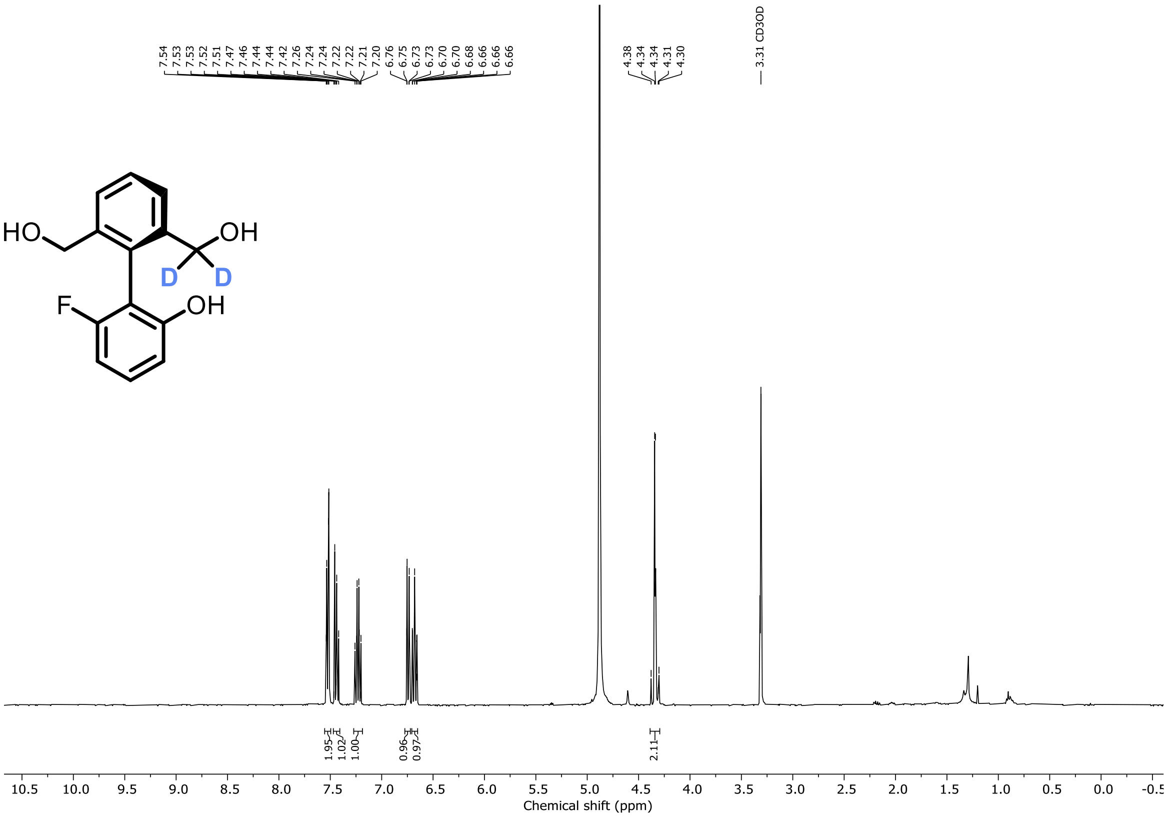

(R_a)-6-Fluoro-2'-(hydroxymethyl)-6'-(hydroxymethyl-d_2)-[1,1'-biphenyl]-2-ol **((R_a)-D_2-3a)**

(^{1}H NMR, 400 MHz, CD_3OD)

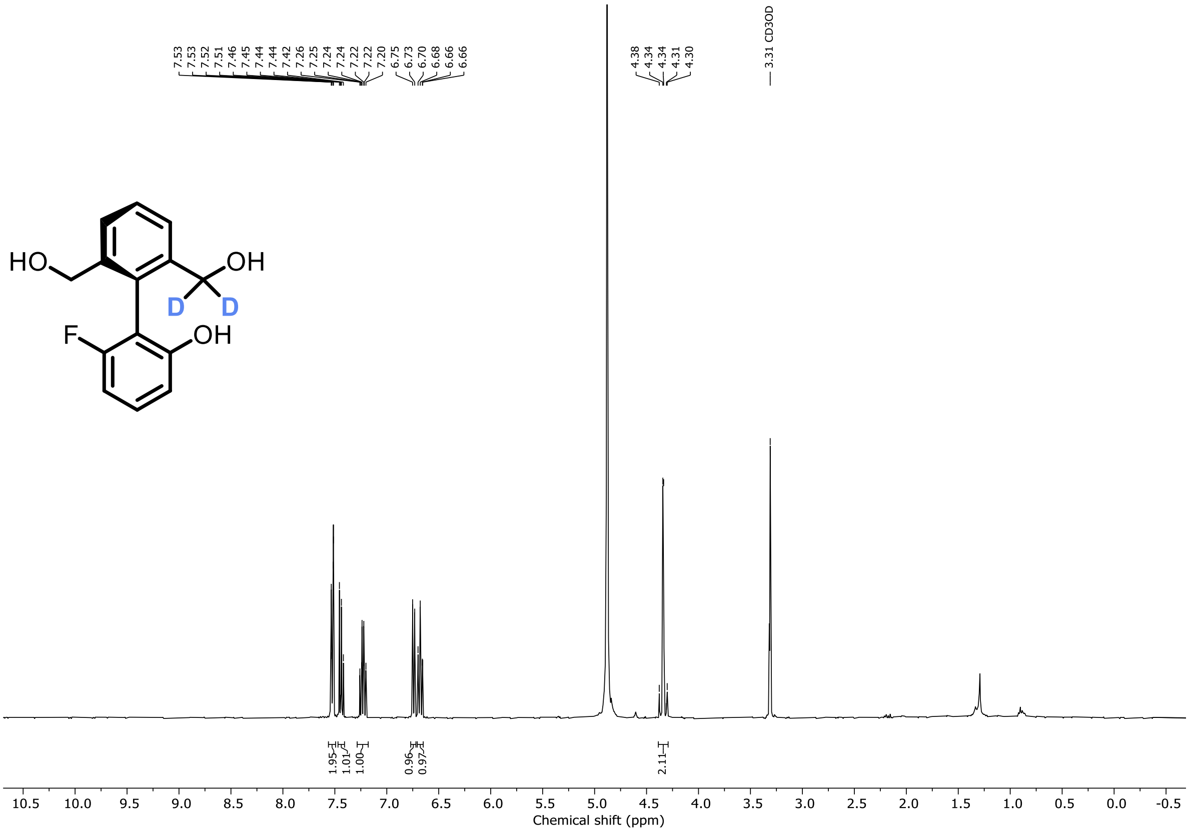

2-(Naphthalen-1-yl)isophthalaldehyde (S8a) (^{1}H NMR, $CDCl_3$, 400 MHz)

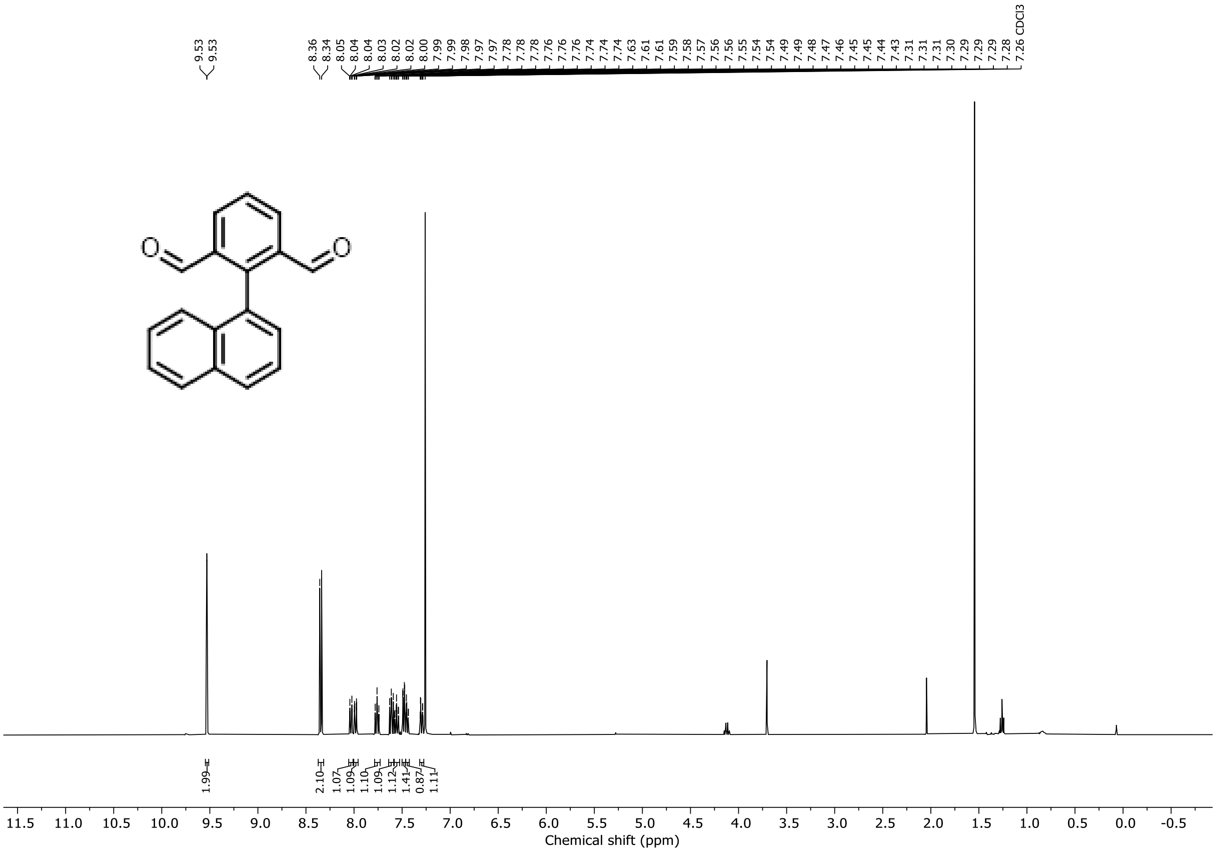

3-(Hydroxymethyl)-2-(naphthalen-1-yl)benzaldehyde (S8b) (^{1}H NMR, 400 MHz, $CDCl_3$)

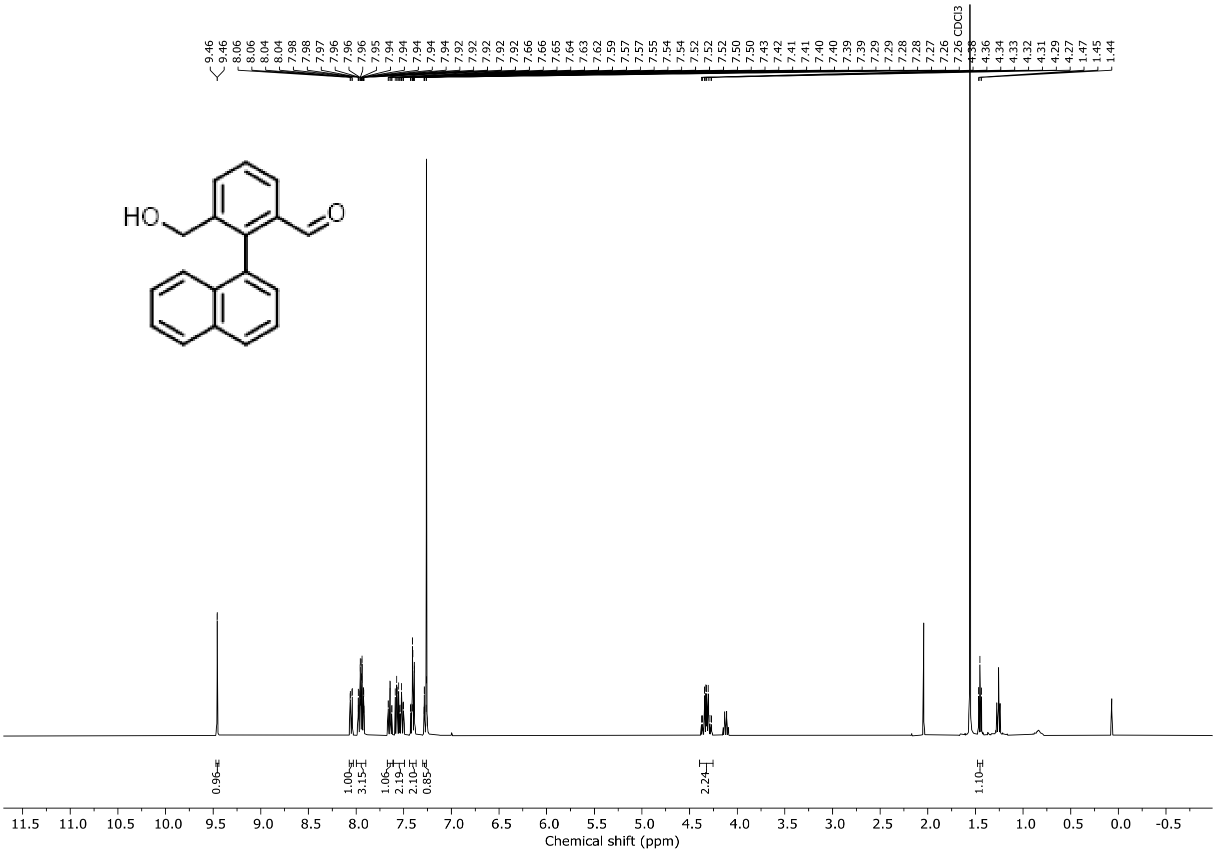

3-(((*tert*-Butyldimethylsilyl)oxy)methyl)-2-(naphthalen-1-yl)benzaldehyde (S8c) (^{1}H NMR, 400 MHz, $CDCl_3$)

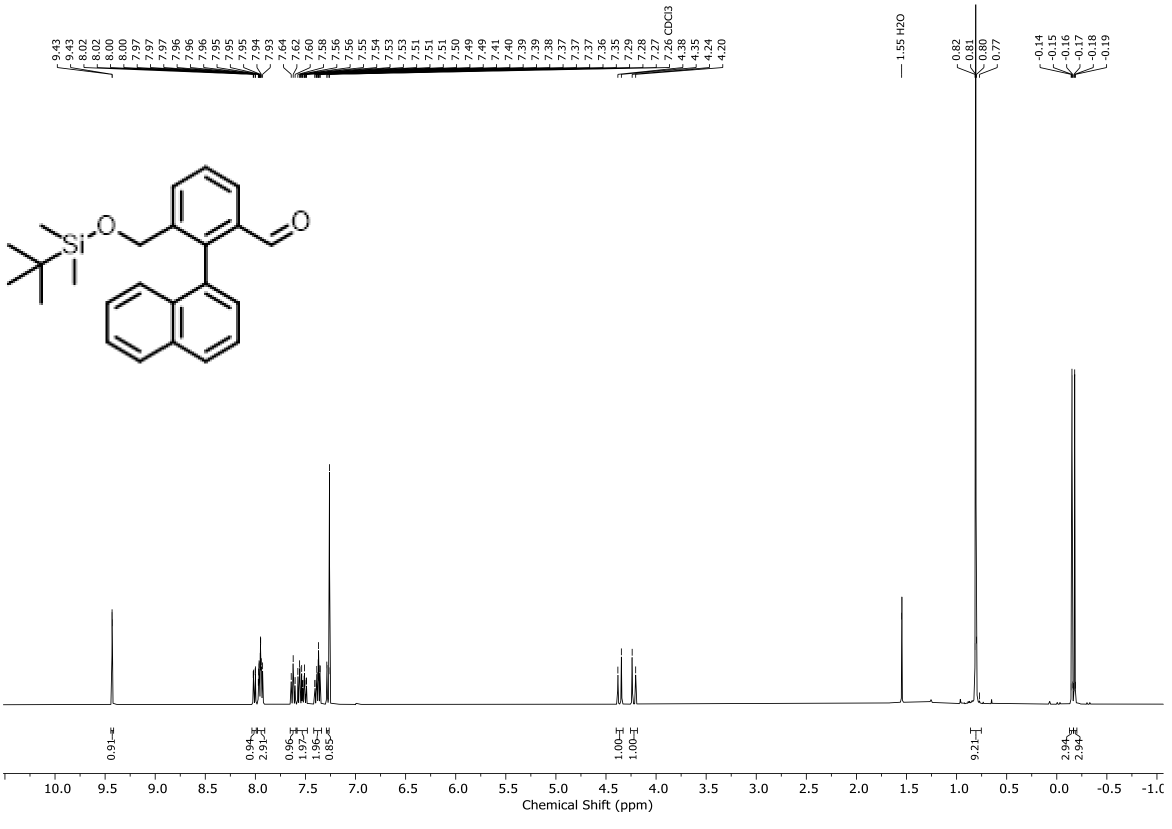

3-(((*tert*-Butyldimethylsilyl)oxy)methyl)-2-(naphthalen-1-yl)benzaldehyde (S8c) (^{13}C NMR, 126 MHz, $CDCl_3$)

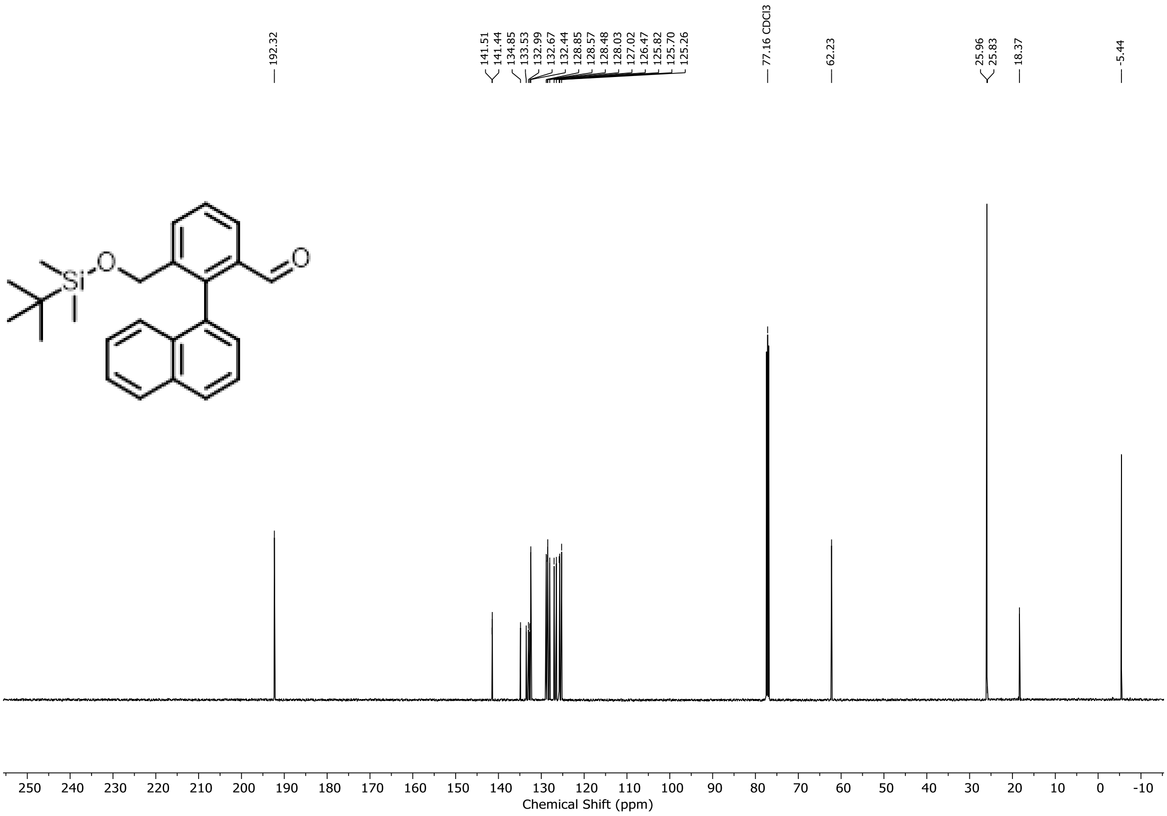

3-(((*tert*-Butyldimethylsilyl)oxy)methyl)-2-(naphthalen-1-yl)benzaldehyde-d$_1$ (D$_1$-S8c) (^{1}H NMR, 400 MHz, $CDCl_3$)

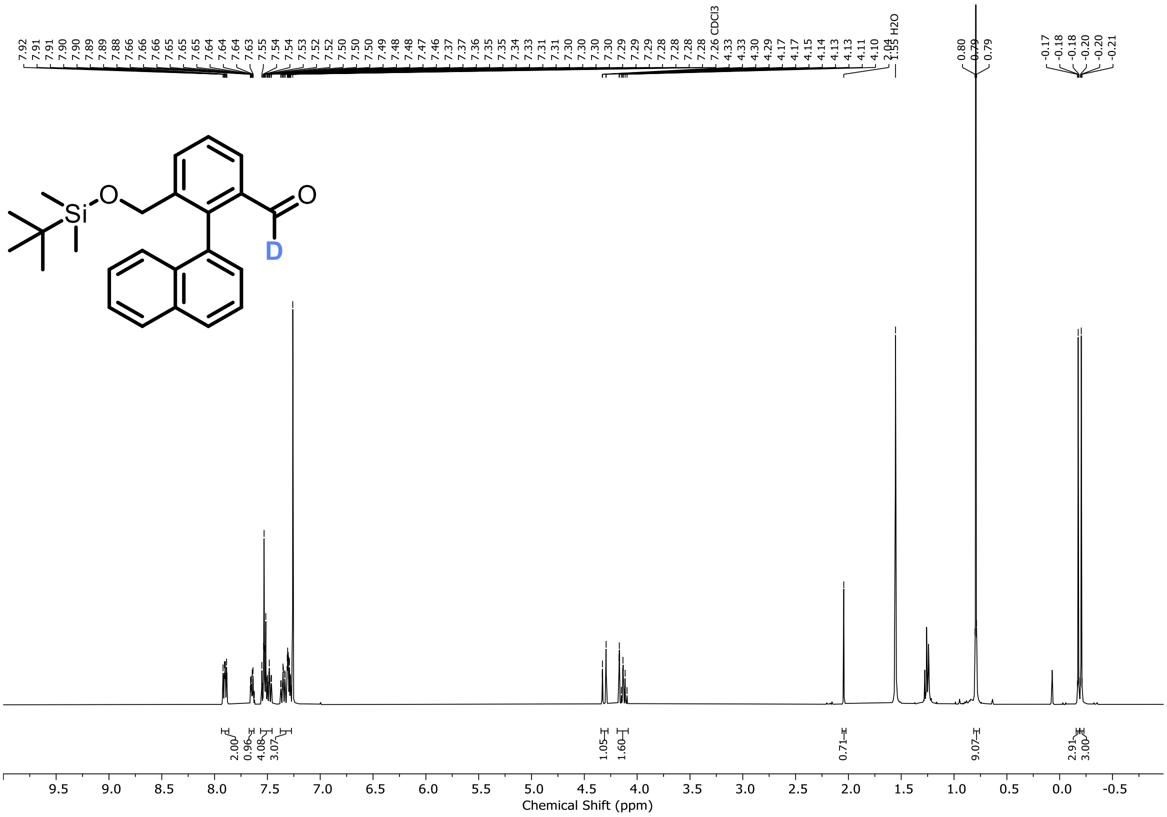

(3-(((*tert*-Butyldimethylsilyl)oxy)methyl)-2-(naphthalen-1-yl)phenyl)methanol (S8d) (^{1}H NMR, 500 MHz, $CDCl_3$)

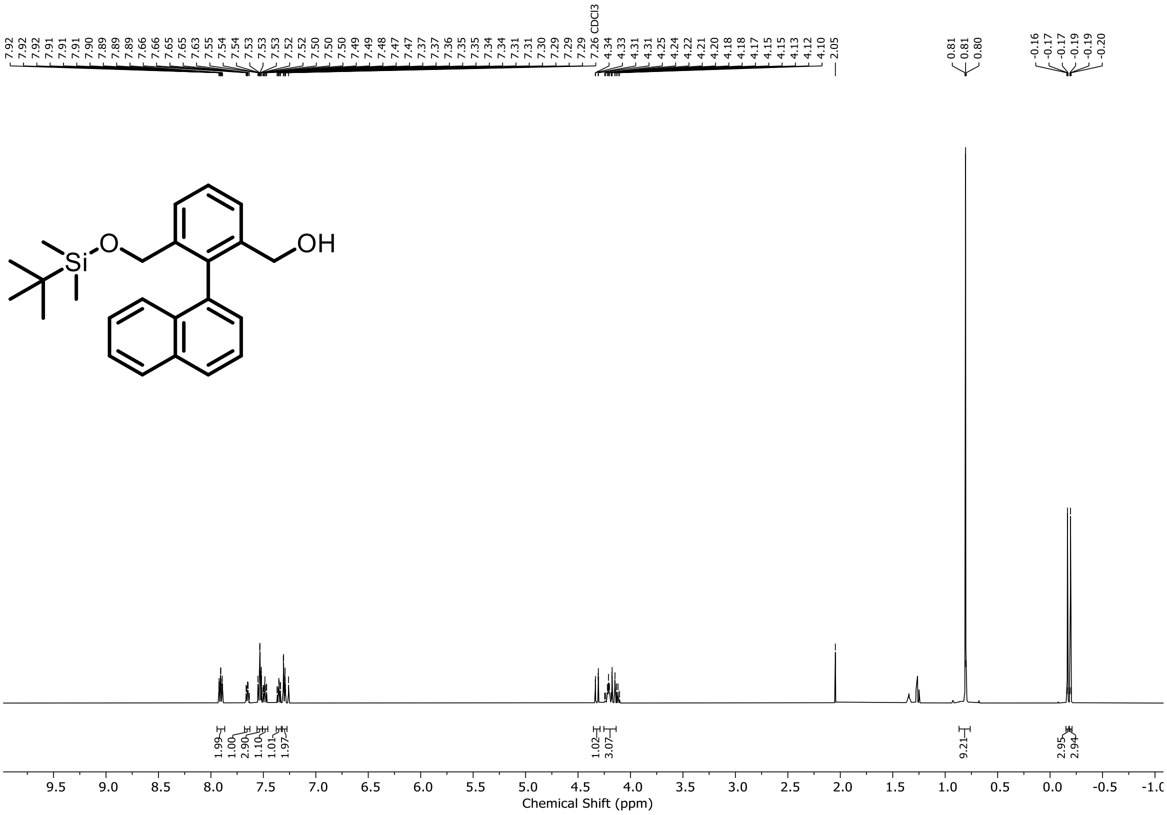

(3-(((*tert*-Butyldimethylsilyl)oxy)methyl)-2-(naphthalen-1-yl)phenyl)methanol (S8d) (^{13}C NMR, 126 MHz, $CDCl_3$)

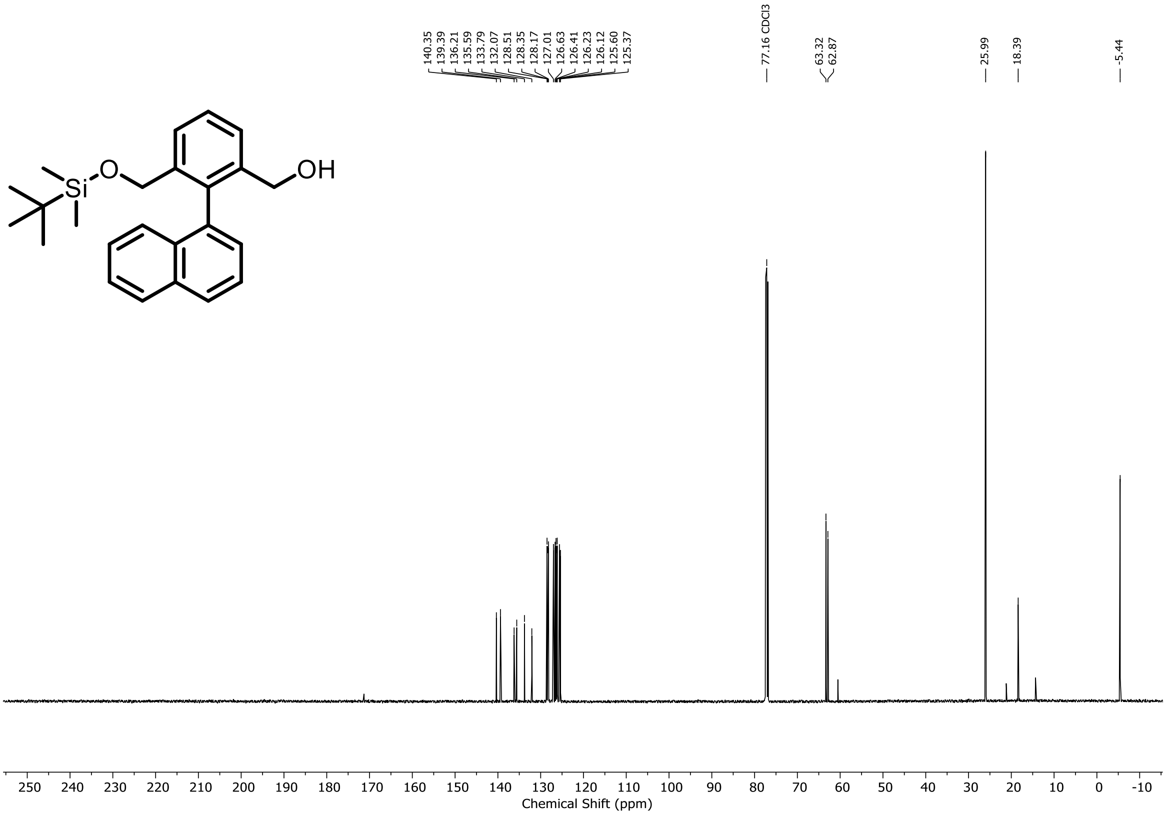

(3-(((*tert*-Butyldimethylsilyl)oxy)methyl)-2-(naphthalen-1-yl)phenyl)methan-d2-ol (D$_2$-S8d) (^{1}H NMR, 400 MHz, $CDCl_3$)

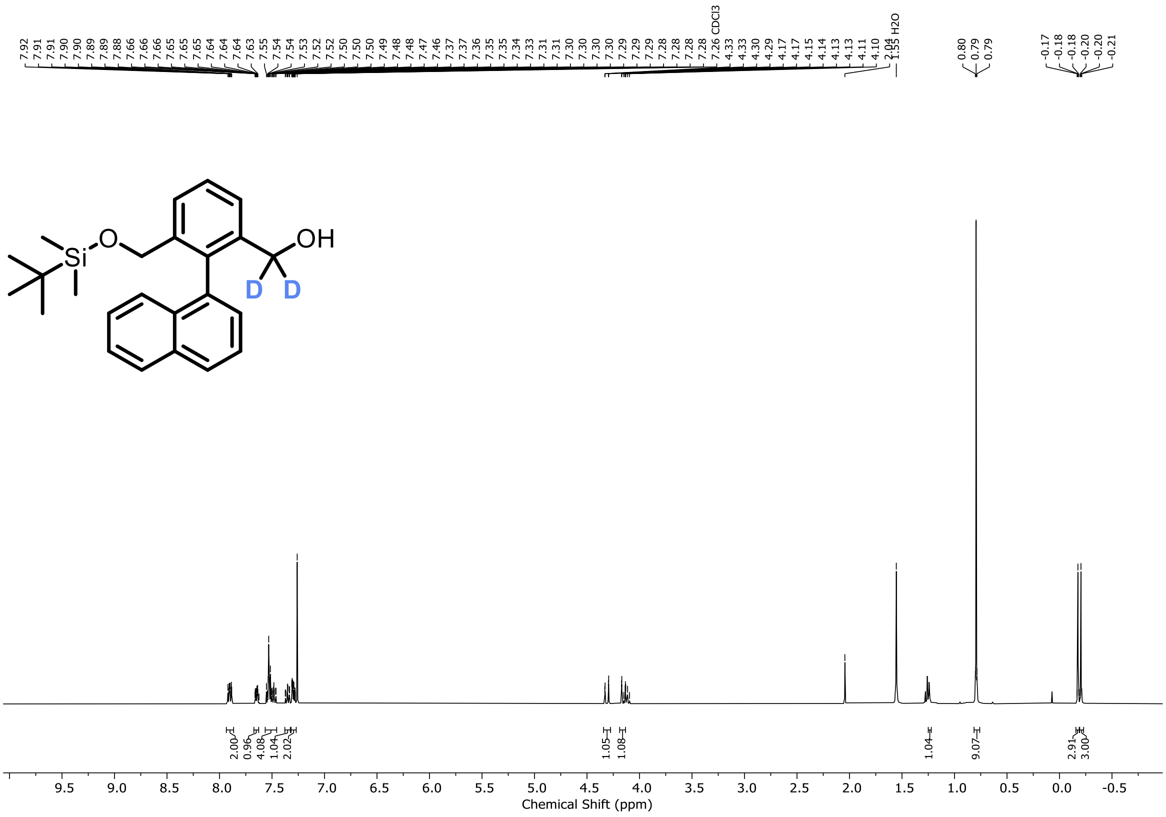

(*S*$_a$)-(3-(Hydroxymethyl)-2-(naphthalen-1-yl)phenyl)methan-*d*$_2$-ol (D$_2$-S8e) (^{1}H NMR, 400 MHz, $CDCl_3$)

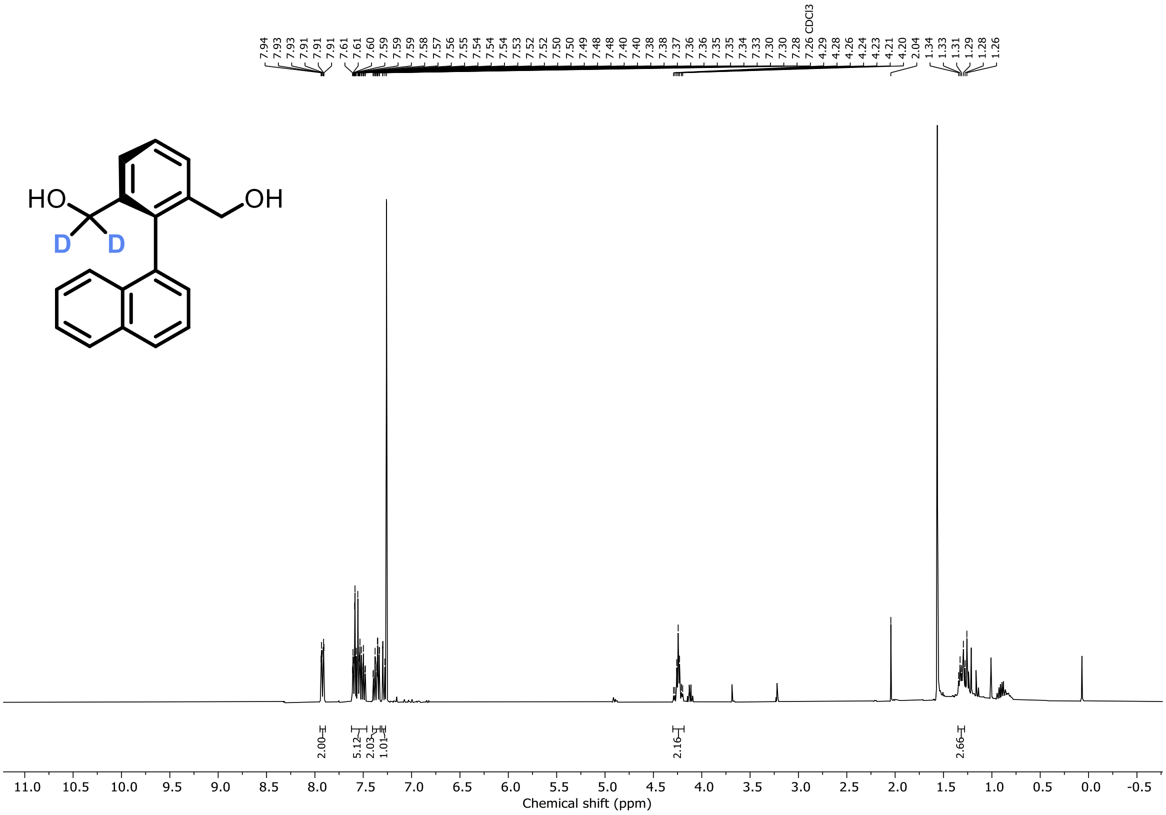

(R_a)-(3-(Hydroxymethyl)-2-(naphthalen-1-yl)phenyl)methan-d_2-ol (D$_2$-S8e) (^{1}H NMR, 400 MHz, $CDCl_3$)

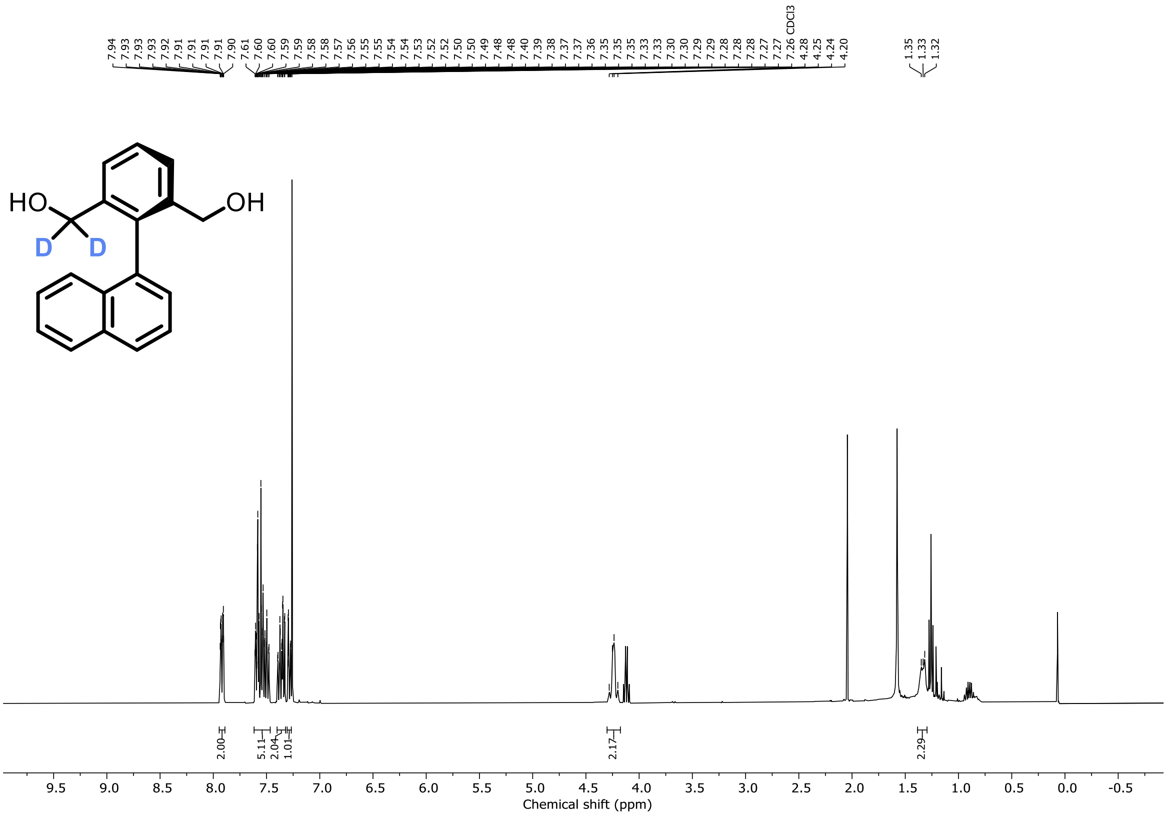

Ammonia trideuteroborane ($H_3N{\cdot}BD_3$) (^{1}H NMR, 400 MHz, CD_3CN)

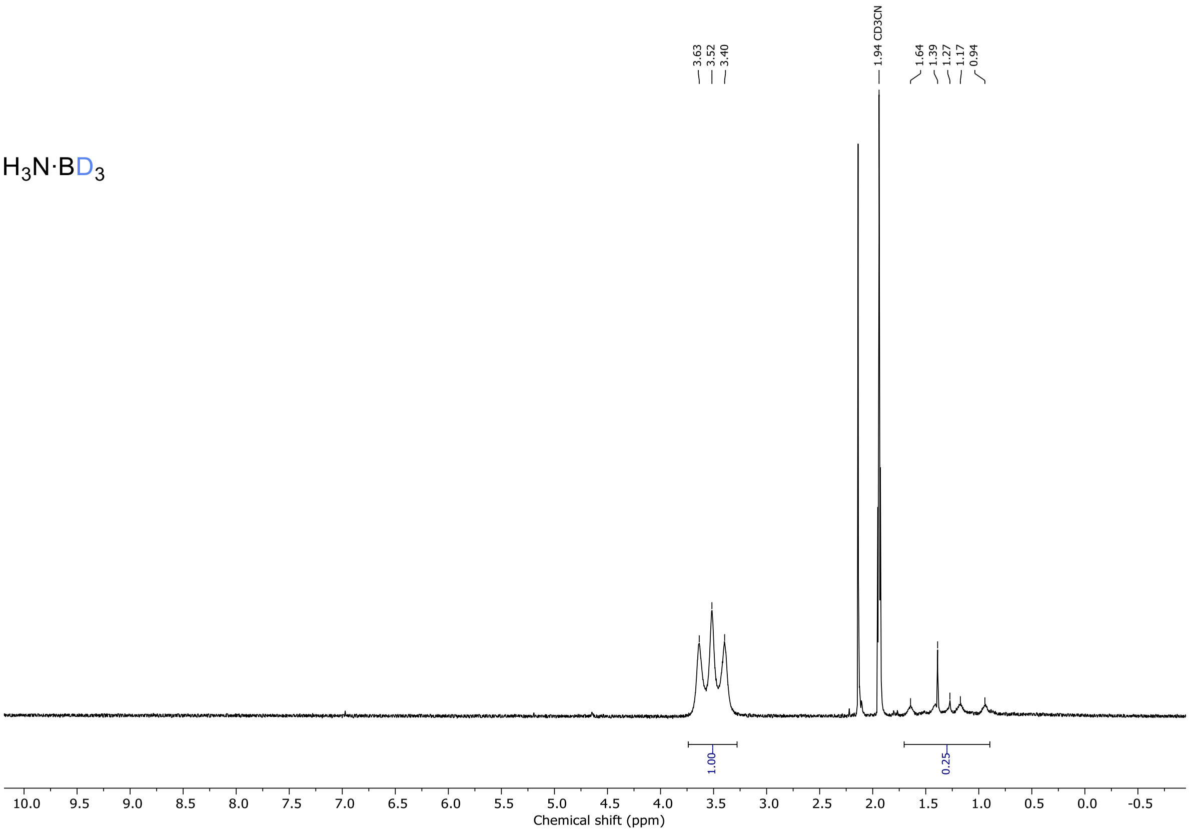

Ammonia trideuteroborane ($H_3N{\cdot}BD_3$) (^{11}B NMR, 128 MHz, CD_3CN)

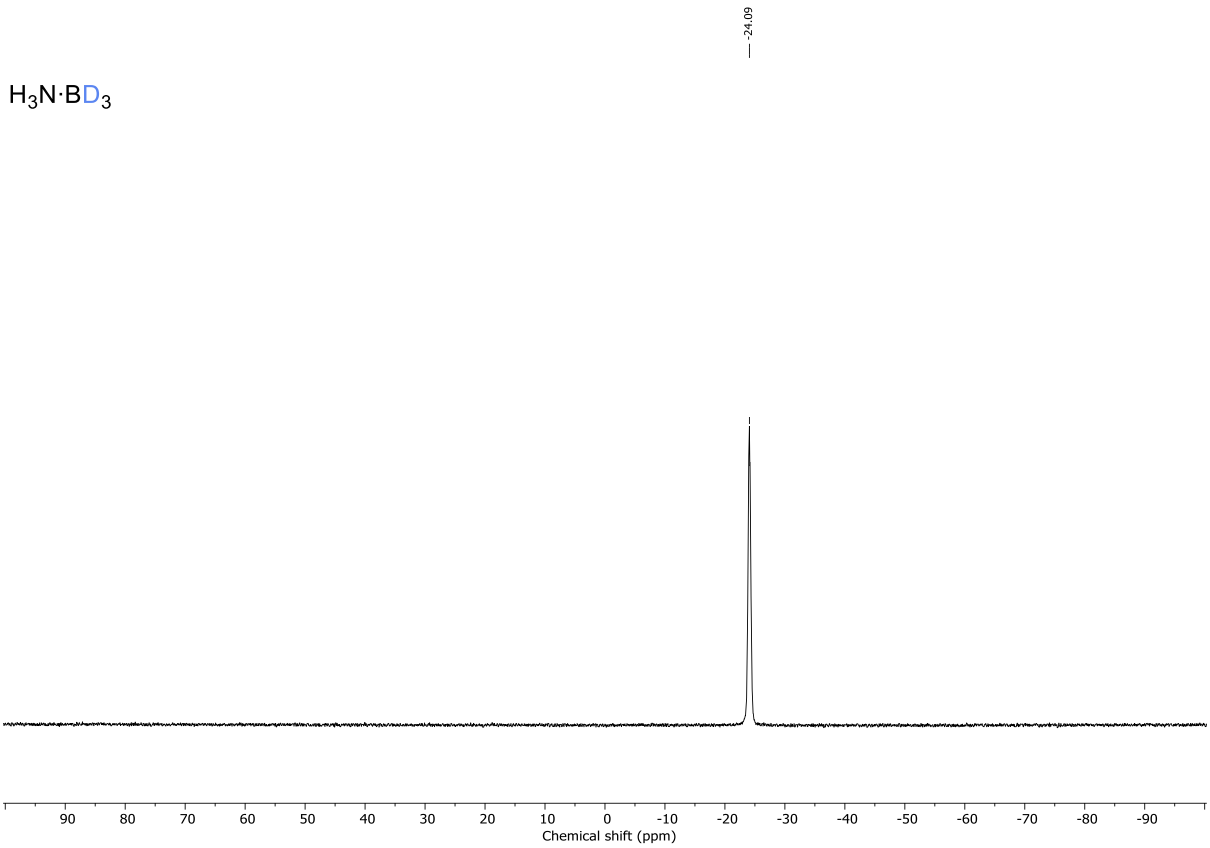

Supplement: Supplementary file 1 — Experimental protocols and characterization data, including Supplementary Figs. 1–49 and Supplementary Tables 1–21. [file 41586_2025_9291_MOESM1_ESM.pdf]
